# Optimized design and data analysis of tag-based cytosine methylation assays

**DOI:** 10.1186/gb-2010-11-4-r36

**Published:** 2010-04-01

**Authors:** Masako Suzuki, Qiang Jing, Daniel Lia, Marién Pascual, Andrew McLellan, John M Greally

**Affiliations:** 1 Department of Genetics (Computational Genetics), Center for Epigenomics, Albert Einstein College of Medicine, 1301 Morris Park Avenue, Bronx, NY 10461, USA

## Abstract

Using the type III restriction-modification enzyme EcoP15I, we isolated sequences flanking sites digested by the methylation-sensitive HpaII enzyme or its methylation-insensitive MspI isoschizomer for massively parallel sequencing. A novel data transformation allows us to normalise HpaII by MspI counts, resulting in more accurate quantification of methylation at >1.8 million loci in the human genome. This HELP-tagging assay is not sensitive to sequence polymorphism or base composition and allows exploration of both CG-rich and depleted genomic contexts.

## Abstract 2

Genome-wide, tag-based cytosine methylation analysis is optimized.

## Background

Epigenetic mechanisms of transcriptional regulation are increasingly being studied for their potential influences in human disease pathogenesis. Much of this interest is based on the paradigm of neoplastic transformation, in which epigenetic changes appear to be universal, widespread throughout the genome, causative of critical transcriptional changes and predictive of disease prognosis (reviewed in [1]). Furthermore, these epigenetic changes represent potential pharmacological targets for reversal and amelioration of the disease process [2].

Of the large number of regulatory processes referred to as epigenetic, there exist numerous assays to study chromatin component distribution, cytosine methylation and microRNA expression genome-wide. The chromatin components include a large number of post-translational modifications of histones, variant histones, DNA-binding proteins and associated complexes, all tested by chromatin immunoprecipitation (ChIP) approaches coupled with microarray hybridization or massively parallel sequencing (MPS). MicroRNAs can be identified and quantified by using microarrays and MPS, while cytosine methylation can be definitively studied by converting the DNA of the genome using sodium bisulfite, shotgun sequencing the product using MPS and mapping this back to the genome to count how frequently cytosines remain unconverted, indicating their methylation in the starting material, due to the resistance of methylcytosine to bisulfite conversion compared with unmethylated cytosines. This allows nucleotide resolution, strand-specific, quantitative assessment of cytosine methylation, with such studies performed in *Arabidopsis *[3-5] and human cells to date [6].

While this approach represents the ideal means of testing cytosine methylation, the amount of sequencing necessary (for the human genome, over 1 billion sequences of ~75 bp each [6]) to generate quantitative information genome-wide remains prohibitive in terms of cost, limiting these studies to the few referred to above. When studying human disease, the emphasis remains on cytosine methylation assays, as it is generally easier to collect clinical samples for DNA purification than for ChIP or even RNA assays. However, the cell populations harvested are rarely of high purity, and we generally do not know the degree of change in cytosine methylation in the disease of interest and thus the quantitative discrimination required for an assay, with some studies to date indicating that the changes may be quite subtle [7]. These concerns emphasize the need for cytosine methylation assays that can detect methylation levels intermediate in value and changes in disease that are relatively modest in magnitude. Certain microarray-based assays to study cytosine methylation have addressed this issue, with the methylated DNA immunoprecipitation (meDIP) assay amenable to such quantification when used for CpG islands [8] and possibly also for less CG dinucleotide-rich regions [9]. Restriction enzyme-based assays used with microarrays have also proven to be reasonably quantitative, whether based on methylation-sensitive (for example, the HELP assay [10]) or methylation-dependent (for example, MethylMapper [11]) enzymes. A promising new MPS-based assay is reduced representation bisulfite sequencing (RRBS), which is designed to study the CG-dense regions defined by short MspI fragments, and provides nucleotide resolution, quantitative data [12].

The use of MPS for what were previously microarray-based assays has been associated with improved performance [13], as we found when we modified our HELP (HpaII tiny fragment Enrichment by Ligation-mediated PCR) assay [10] for MPS, creating an assay similar to Methyl-Seq [14]. The strength of the HELP assay involves the comparison of the HpaII with the methylation-insensitive MspI representation, allowing a normalization step that makes the assay semi-quantitative [10]. The HELP representation approach was improved upon by Ball *et al. *[15], who developed the Methyl-Sensitive Cut Counting (MSCC) assay, which involves digesting DNA with HpaII, ligating an adapter to the cohesive end formed, using a restriction enzyme site within the adapter to digest at a flanking sequence and thus capturing the sequence immediate adjacent to the HpaII site. By adding a second MPS-compatible adapter, a library can be generated for MPS, allowing the counting of reads at these sites to represent the degree of methylation at the site. The authors demonstrated the assay to be reasonably quantitative, testing over 1.3 million sites in the human genome, representing not only HpaII sites clustered in CG-dense regions of the genome (approximately 12% of all HpaII sites are located in annotated CpG islands in the human genome [16]) but also the remaining majority of the genome in which CG dinucleotides are depleted, a genomic compartment not tested by RRBS as currently designed. A focus on the CG-dense minority of the genome will fail to observe changes such as those at CG-depleted promoters (such as *OCT4 *[17]) and CpG island shores [18], and within gene bodies where cytosine methylation has been found to be positively correlated with gene transcription [15]. It is likely, therefore, that an assay system that can study both CG-dense and CG-depleted regions will acquire substantially more information about epigenomic states than those directed at the CG-dense compartment alone.

In the current study, we tested whether the use of an MspI control would improve MSCC assay performance, as we had found for microarray-based HELP, and whether we could develop an analytical pipeline for routine use of this assay in epigenome-wide association studies. We also explored the use of longer tags than those employed in the MSCC, and added T7 RNA polymerase and reverse transcription steps to allow the generation of libraries without contaminating products, thus obviating the need for gel extraction. The influence of base composition and fragment length parameters as potential sources of bias were also tested, using the H1 (WA01) human embryonic stem (ES) cell line. The outcome is a modified assay that combines the strengths of MSCC and HELP-seq/Methyl-seq, and the supporting analytical workflow that maximizes the quantitative capabilities of the data generated.

## Results and discussion

### Library preparation and sequencing

**Figure 1:**
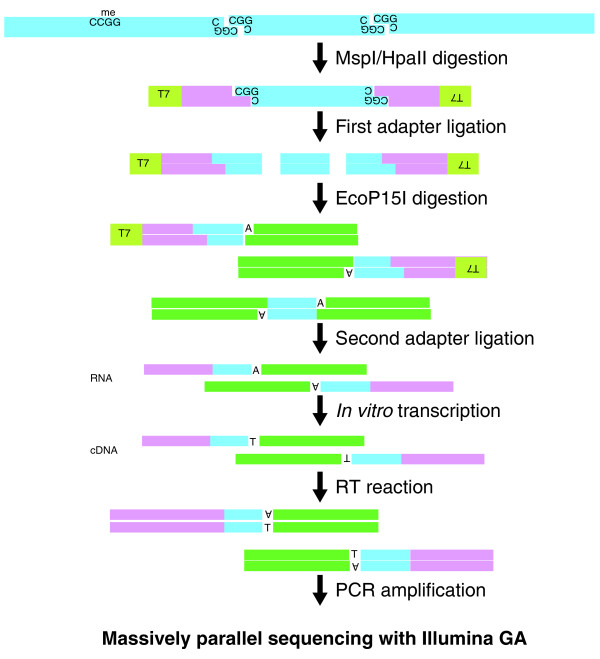
**HELP-tagging assay design and library preparation**. The genomic DNA is digested by HpaII or MspI, the former only cutting at CCGG sequences where the central CG dinucleotide is unmethylated. The first Illumina adapter (AE) is ligated to the compatible cohesive end created, juxtaposing an EcoP15I site beside the HpaII/MspI digestion site and allowing EcoP15I to digest within the flanking DNA sequence as shown. An A overhang is created, allowing the ligation of the second Illumina adapter (AS, green). This will create not only AE-insert-AS products but also AS-insert-AS molecules. By performing a T7 polymerase-mediated *in vitro *transcription from a promoter sequence located on the AE adapter, we can selectively enrich for the AE-insert-AS product, following which limited PCR amplification is performed to generate a single sized product for Illumina sequencing. RT, reverse transcription.

We generated HELP tagging libraries with HpaII- or MspI-digested DNA derived from human ES cells using the experimental approach shown in Figure 1. The assay differs from MSCC [15] by using EcoP15I instead of MmeI, generating longer flanking sequences (27 as opposed to 18 to 19 bp) and the addition of a T7 polymerase and reverse transcription step to allow the generation of the library without contaminating single-adapter products, while in addition obviating the need for gel extraction. After the library preparation, a single band of 125 bp in length was generated, as expected. Libraries were sequenced using an Illumina Genome Analyzer (36 bp single end reads) and the sequences were analyzed and aligned using Illumina pipeline software version 1.3 or 1.4. A summary of the Illumina analysis results for each replicate is shown in Table S1 in Additional file 1.

### Data quality and reproducibility

Based on our experimental design, successfully generated products would be expected to possess a 5'-CGCTGCTG sequence at the 3' end of the read, the first two nucleotides (CG) representing the cohesive end for ligation of HpaII/MspI digestion products, the remaining six nucleotides the EcoP15I restriction enzyme recognition site. In order to evaluate the yield of desired products, we counted the number of reads containing this sequence and plotted the starting positions of this sequence within the reads obtained. We observed that approximately two-thirds of the reads contained the expected sequence, and found that the majority was located at base positions 25 and 26, consistent with the known digestion properties of the restriction enzyme [19]. Removal of the approximately 30% of reads lacking the CG-EcoP15I sequence was performed to eliminate spurious sequences. In order to investigate sequence quality further, we also determined the number and relative position of Ns (ambiguous base calls) within the reads obtained. Overall, few reads were found to contain Ns, and where they were present, they were found to be evenly distributed by position within the sequence. To test data reproducibility, we compared the results of three experimental replicates against each other using the Pearson correlation coefficient metric. The results of this study showed that all replicates were highly correlated (all the *r *values exceed 0.9), which confirmed that the technical reproducibility of this assay was excellent (Table S2 in Additional file 1).

### Distribution of MspI/HpaII sequence tags

We merged three lanes of MspI data and observed that approximately 80% of the 2,292,198 annotated HpaII sites in the human genome (hg18) were represented by at least one read, for a total of over 1.8 million loci throughout the genome. The mean numbers of reads per locus for MspI and HpaII were 3.94 and 1.82, respectively, and MspI counts were distributed evenly across all genomic compartments examined (Table S3 in Additional file 1). We hypothesize that a combination of incomplete genomic coverage and polymorphisms within some CCGG sites (as we have previously observed [10]) accounts for the 20% of HpaII sites that were not represented by any reads.

### Normalization of HpaII by MspI counts and data transformation

**Figure 2:**
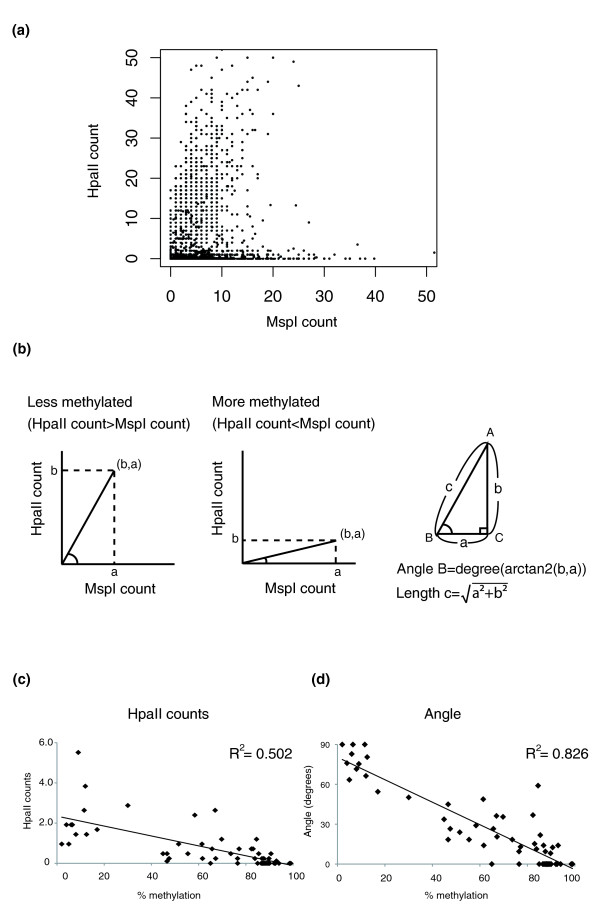
**Data transformation and bisulfite validation**. **(a) **Scatter plot showing the relationship between the number of HpaII and MspI reads at each locus. **(b) **The location of the data point on the scatter plot indicates whether it is likely to be less or more methylated with larger or smaller angles B subtended as shown, while the confidence of the measurement will be greater when more reads represent the data point, represented by the length of line c. **(c) **The HpaII count correlates negatively with the degree of methylation, with more counts occurring at loci with less methylation. **(d) **Transformation of the data to the B angle measure to normalize HpaII by MspI counts substantially improves the correlation with bisulfite MassArray validation data.

**Figure 3:**
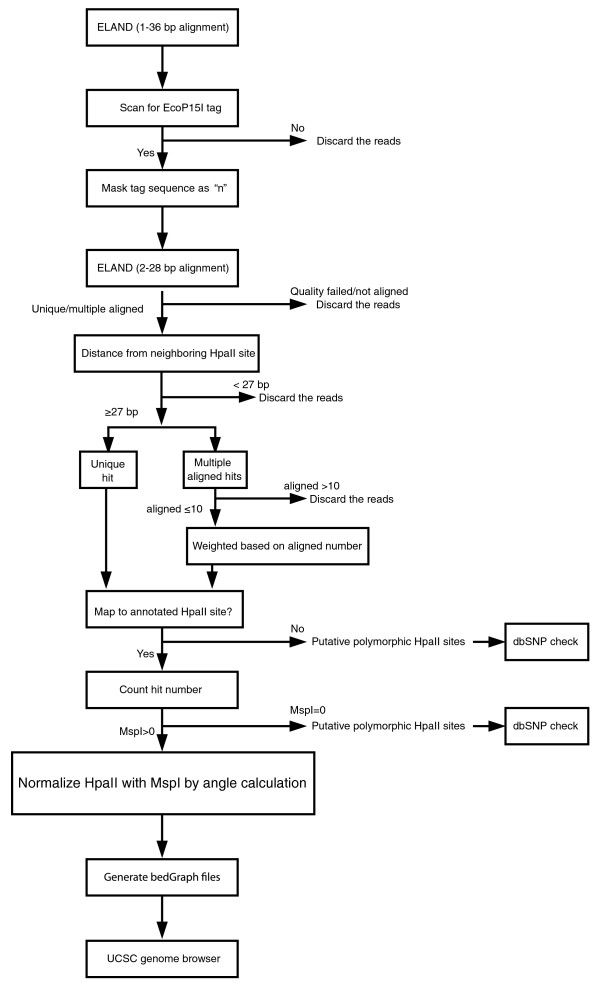
**HELP-tagging analysis workflow**. The analysis workflow for HELP-tagging data is illustrated. Only sequence reads that contain the adapter sequence and map to a single or ≤ 10 sites are retained, the latter repetitive sequences distributed by weighting among the matched loci. Potential polymorphic loci are annotated. Normalization of HpaII by MspI using the angle calculation described in the previous figure is performed and files are generated for genome browser visualization. UCSC, University of California Santa Cruz.

When we plot the MspI count on the x-axis and HpaII count on the y-axis for each HpaII site, we can see two major groups of values in the plot (Figure 2a), separated into loci with high or with minimal HpaII counts. This plot helped us to develop a new method for normalizing HpaII counts in terms of variability of the MspI representation. We recognize that hypomethylated loci are associated with relatively greater HpaII counts and a larger angle B (Figure 2b, left) whereas methylated loci will be defined by smaller angle values (Figure 2b, middle). Furthermore, some loci will tend to be sequenced more readily than others, and may have identical B values but differing distances from the origin (c distance), allowing a confidence score for identical methylation values (B) in terms of the c distance values (Figure 2b, right). To test this model, we used bisulfite MassArray to test quantitatively the cytosine methylation values for 61 HpaII sites (Tables S4, S5 and S6 in Additional file 1), choosing loci representing all components of the B angle spectrum of values. In Figure 2c, d we show the correlations between these gold standard cytosine methylation values and raw HpaII counts or B angle values. We find that there is the same negative correlation (R^2 ^= 0.502) between HpaII counts and cytosine methylation values as demonstrated in the MSCC technique [15], and that the angular transformation of the data incorporating the MspI normalization substantially improves this correlation (R^2 ^= 0.826), defining the optimal approach for processing of these data. We represent the data for University of California Santa Cruz (UCSC) genome browser visualization as wiggle tracks, with higher B angle values defining less methylated loci. Methylated loci with zero values that would be otherwise difficult to visualize as having been tested are represented as small negative values. We show the details of the analytical workflow in Figure 3 and an example of a UCSC genome browser representation of HELP-tagging data in Figure S1 in Additional file 1. All data are available through the Gene Expression Omnibus database (accession number [GEO:GSE19937]) and as UCSC genome browser tracks [20].

### Potential sources of bias: base composition and fragment length

**Figure 4:**
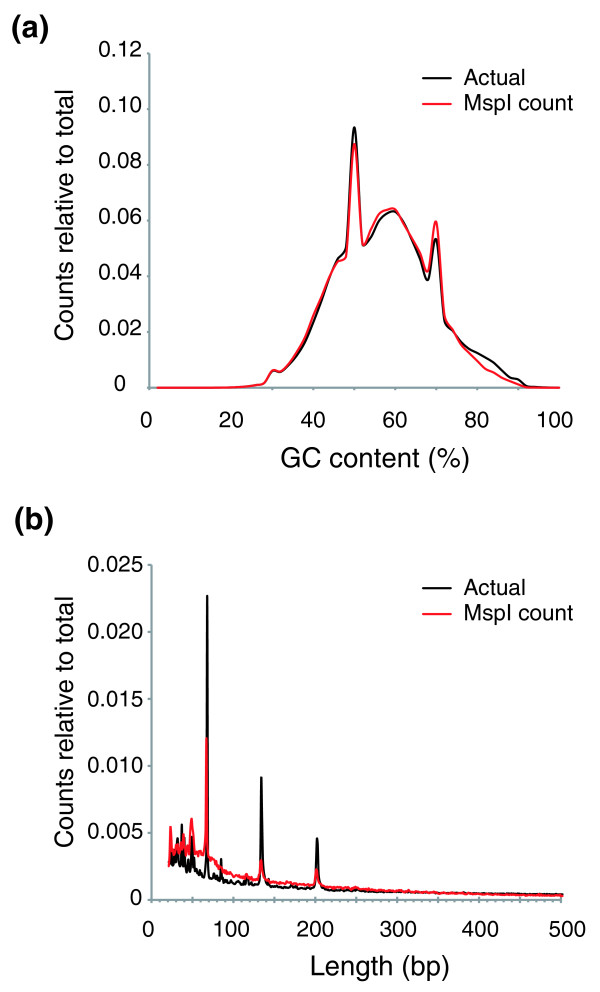
**Base composition and fragment length influences on sequence counts**. **(a) **The proportion of (G+C) nucleotides was calculated for the 50-bp sequence centered around each annotated CCGG in the reference human genome. The base composition of all of the MspI sequences generated from the human ES cell line studied was also calculated. The relative proportion for (G+C) content in 2% bins for each set of data was calculated and plotted as shown. The black line shows the proportions in the reference genome, while the red line illustrates the distribution we observed in our MspI experiment. Two peaks representing base composition in repetitive sequences are apparent. The MspI distribution closely matches the expected distribution except when the base composition exceeds approximately 80%, when it is slightly under-represented. **(b) **We calculated the relative frequencies of MspI digestion product sizes in the human reference genome. In this case we found that the shorter fragments are more likely to be sequenced than larger (≥300 bp) fragments. The three major peaks observed represent Alu short interspersed repetitive element (SINE) sequences.

As the number of reads at CCGG sites following MspI digestion should not be influenced by methylation, the representation obtained from MspI digestion allowed us to look for systematic sources of bias inherent to the assay. A major concern was that base composition could be a source of such bias, as it has been reported that Illumina sequencing can be influenced by GC composition [21], possibly because of the gel extraction step [22]. Our protocol does not require gel extraction and only begins to show an under-representation of sequences when the (G+C) content exceeds approximately 80% (Figure 4a). We also tested to see whether the sizes of the MspI fragments generated influenced the counts obtained, as the digestion by type III endonucleases like EcoP15I is most efficient when a pair of enzymes is present in convergent orientation on the same DNA molecule [19]. We find that there is indeed an over-representation for shorter (≤300 bp) and a corresponding modest under-representation for larger MspI fragments (Figure 4b).

### Identification of polymorphic CCGG sequences

**Figure 5:**
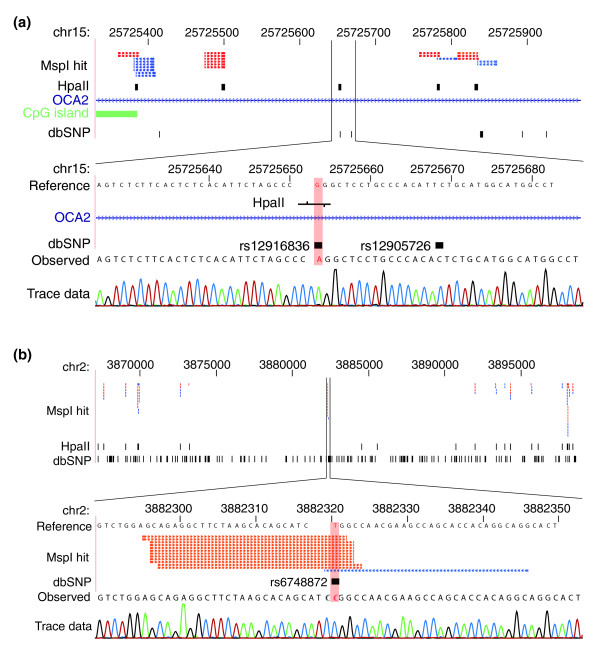
**Polymorphic HpaII sites identified by HELP-tagging**. Examples of HpaII sites **(a) **annotated in the reference genome sequence but not represented by MspI reads or **(b) **not annotated in the reference human genome and represented by at least four MspI reads are shown. In each case there is a SNP defined by dbSNP that indicates the C:T to G:A transversion that eliminates or restores the CCGG HpaII site.

Whereas MSCC used MmeI and generates an 18- to 19-bp sequence flanking the HpaII site [15], our use of EcoP15I generates a 27-bp flanking sequence. We asked whether this size difference influenced our ability to align sequences to the reference genome. We truncated our sequence reads to 19 bp to mimic the MSCC read length and found that this caused a profound loss of ability to align reads unambiguously (Table S7 in Additional file 1). To compensate for the low alignment rate, the MSCC report described an ingenious strategy of alignment to the sequences immediately flanking the annotated HpaII sites in the reference genome [15], an approach sufficiently powerful that it generated the well-validated data that they described. However, it does not offer the possibility of identifying polymorphic HpaII sites at the high frequencies that we previously observed for our HELP-seq assay [10]. We tested whether our longer sequences allowed the identification of loci at which an HpaII site is annotated in the reference genome but we obtain no sequence reads, and the opposite situation where we observed at least four MspI reads (the average number per annotated MspI/HpaII site) flanking a locus not annotated in the reference genome. In Table S8 in Additional file 1 we list approximately 6,600 candidate polymorphic HpaII sites, of which examples are shown in Figure 5, confirmed by targeted resequencing of those loci. The 6,600 loci were selected based on overlap with dbSNP entries, allowing us to evaluate the pattern of sequence variability at these loci. Approximately 80% of the SNPs are C:G to T:A transversions, consistent with deamination-mediated decay of methylcytosine being the cause of the polymorphism [23]. Polymorphic CG dinucleotides are major potential sources of error not only for microarrays, which are designed to a consensus genomic sequence, but also for both bisulfite sequencing, which would read the C to T transversion as unmethylated, and mass spectrometry-based assays, requiring the development of specific analytical approaches such as we have described [24].

### DNA methylation studies of human embryonic stem cells

**Figure 6:**
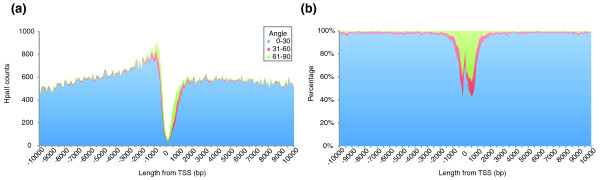
**Identification of a position effect on DNA methylation in transposable elements located close to gene promoters. **The distance from RefSeq gene transcription start sites and DNA methylation status are shown. The x-axis displays the distance from transcription start sites (TSSs). HpaII sites were categorized into three groups by angle, 0 to 30 (blue), 31 to 60 (red) and 61 to 90 (green). **(a)** Number of HpaII sites; **(b)** proportions of each angle category (%).

To test whether the HELP-tagging assay was generating data that are biologically plausible, we tested the methylation of different genomic sequence compartments as density plots of B angle values for the human ES cells used in these studies. In Figure S2a in Additional file 1 we show how promoters (defined as -2 kb to 2 kb from the transcription start site of RefSeq genes), gene bodies (the remaining region within the RefSeq gene) and intergenic (all other) sequences compare, finding the expected enrichment of hypomethylated loci with larger B angle values in promoter regions. When we compared unique with repetitive sequences, again we found the expected pattern of increased methylation of repetitive DNA compared with unique sequences (Figure S2b in Additional file 1). Combining these observations, we tested whether the transposable element component of annotated repetitive DNA sequences showed any tendency to unusual methylation near gene promoters. In Figure 6 we show that while transposable elements are generally methylated and are depleted near gene promoters, those that are proximal to promoters tend to be less methylated than those located more distally. While many types of transposable elements were represented in this promoter-proximal hypomethylated group, we found a subset to be the most markedly over-represented, as shown in Figure 6c.

The outcome of these studies was an improvement in the previously described MSCC [15] and HELP-seq [10] assays, not only by means of technical modifications such as the use of EcoP15I but also because of the concurrent use of MspI for normalization. The effect of these modifications was not only to increase the accuracy of the assay but also to enhance the ability to align sequences to the genome and thus identify polymorphic HpaII/MspI sites. The means of normalization of HpaII by MspI using an angular metric is an innovation that improved the data accuracy substantially and may have applications in other MPS assay normalization strategies. We were also able to discard reads that did not contain the expected adapter sequences, and created a straightforward data analytical pipeline that will facilitate processing of these HELP-tagging data by others.

The potential sources of systematic artifacts due to base composition or digestion product size were evaluated. Apart from a modest decrease in representation in regions above approximately 80% (G+C) content, base composition did not cause biases in representations, possibly in part due to our avoidance of a gel purification step in library preparation [22]. Fragment length does influence the outcome, most likely due to effects on EcoP15I digestion [19], although the effects should be similar for both HpaII and MspI and should, therefore, largely cancel each other out in the normalization step. It is possible that endogeneous EcoP15I sites could influence the representations, but to have an effect they would have to be located within the 27 bp adjacent to HpaII/MspI sites and would cause digestion of the ligated adapter, causing those loci to be under-represented in both HpaII and MspI datasets. The most likely effect of these endogeneous sites is that they contribute to the proportion of loci at which we could not obtain sequence reads.

Our exploration of the distribution of cytosine methylation in the same human ES cell line studied by Lister *et al. *[6] showed consistent results, with hypomethylation of transcription start sites and methylation of transposable elements, as expected from long-standing observations in the field. We furthermore discovered a limited subset of transposable elements that is hypomethylated when in close proximity to transcription start sites. When this subset was studied to determine whether certain types of transposable elements were disproportionately over-represented, we found two broad classes, one of transposable element fossils with no innate capacity to replicate themselves (the ancient DNA, long interspersed repetitive elements (LINEs) and short interspersed repetitive elements (SINEs) shown in Figure 6c) and younger ERV1 long terminal repeat retroelements. Loss of methylation of functionally inactive transposable elements is likely to be of no negative consequence to the host genome, consistent with the host defense hypothesis [25], while the young ERV long terminal repeats represent a group of transposons whose function has been harnessed as promoters of endogeneous genes [26,27]. This observation demonstrates the value of a high-resolution, genome-wide assay like HELP-tagging to define potential functional elements in an unbiased manner.

## Conclusions

We propose that MPS-based assays such as RRBS [12], MSCC [15] and HELP-tagging will prove to be the assays of choice for epigenome-wide association studies in human disease, with the latter two preferable as we begin to explore the CG-depleted majority of the genome. It should not be necessary to run MspI assays every time a HELP-tagging assay is performed, suggesting that a common MspI dataset can serve as a universal reference for a species, allowing a single lane of Illumina sequencing of the HpaII library to provide the methylation data for that sample. The development of analytical pipelines to support analysis of these datasets will be critical to the success of these projects, while the careful ongoing assessment of potential sources of bias will also be essential for improving assay performance.

## Materials and methods

### Cell preparation and DNA purification

H1 human ES cells (NIH code WA01 from Wicell Research Institute, Madison, WI, USA) were cultured on matrigel (BD Biosciences, San Diego, CA, USA), at 37°C, 5% O_2 _and 5% CO_2_. Amplified human ES cell pluripotency was assessed by flow cytometry with SSEA4, CD24 and Oct4 markers. To extract DNA, the cells were suspended in 10 ml of a solution of 10 mM Tris-HCl (pH 8.0), 0.1 M EDTA and 1 ml of 10% SDS to which 10 μl of RNase A (20 mg/ml) was added. After incubation for 1 hour at 37°C, 50 μl of proteinase K (20 mg/ml) was added and the solution was gently mixed and incubated in a 50°C water bath overnight. To purify the lysate, it was extracted three times using saturated phenol, then twice with chloroform, and dialyzed for 16 hours at 4°C against three changes of 0.2× SSC. Following dialysis, the DNA was concentrated by coating the dialysis bags in polyethylene glycol (molecular weight 20,000). The purity and final concentration of the purified DNA was checked by spectrometry (Nanodrop, Wilmington, DE, USA).

### Illumina library preparation

The sample preparation steps are illustrated in Figure 1. Two custom adapters were created for HELP-tagging, referred to as AE and AS. As well as an Illumina adapter sequence, adapter AE contains an EcoP15I recognition site and a T7 promoter sequence. Adapter AS contains an Illumina sequencing primer sequence. The adapter and primer sequences for library preparation are listed in Table S9 in Additional file 1. Genomic DNA (5 μg) was digested with HpaII and MspI in separate 200 μl reactions and purified by phenol/chloroform extraction followed by ethanol precipitation. The digested genomic DNA was ligated to adapter AE using a New England Biolabs Quick Ligation Kit (25 μl of 2× Quick ligase buffer, 3 μg of HpaII-digested DNA or 1 μg of MspI-digested DNA, 0.1 μl of Adapter AE (1 μM), 3 μl of Quick Ligase in a final volume of 50 μl). After AE ligation, the products were purified using Agencourt AMpure beads (Beckman Coulter, Brea CA, USA), then digested with EcoP15I (New England Biolabs). The restriction fragments were end-repaired to inhibit to dimerization of adapters, and tailed with a single dA, at the 3' end. After the dA tailing reaction, adapter AS was ligated to the dA-tailed fragments using a New England Biolabs Quick Ligation Kit (25 μl of 2× Quick ligase buffer, 2.5 μl of adapter AS (10 μM), 2.5 μl of Quick Ligase in a final volume 50 μl). After ligation, products were purified using the MinElute PCR purification kit (Qiagen, Hilden, Germany) and *in vitro*-transcribed using the Ambion MEGAshortscriptkit (Life Technologies, Carlsbad, CA, USA). Following *in vitro *transcription, products were purified with an RNeasy clean-up kit (Qiagen) before reverse transcription was performed using the Invitrogen SuperScript III kit (Life Technologies). The first strand cDNA produced was used as a template for PCR using the following conditions: 96°C for 2 minutes, then 18 cycles of 96°C for 15 seconds and 72°C for 15 seconds followed by 5 minutes at 72°C for the final extension. After PCR, the library was purified using a QIAQuick PCR clean-up kit (Qiagen).

### Single-locus quantitative validation assays

Bisulfite conversion and MassArray (Sequenom, San Diego, CA, USA) were performed using an aliquot of the same sample of DNA as was used for the high-throughput assays described above. Bisulfite conversion was performed with an EZ DNA Methylation kit (Zymo Research, Orange, CA, USA). Bisulfite primers were designed using MethPrimer [28], specifying the desired product length (250 to 450 bp), primer length (23 to 29 bp) and primer Tm (56 to 62°C). PCR was performed using FastStart High Fidelity Taq polymerase (Roche, Basel, Switzerland) with the following conditions: 95°C for 10 minutes, then 42 cycles of 95°C for 30 seconds, primer-specific Tm for 30 seconds and 72°C for 1 minute, followed by 72°C for 10 minutes for the final extension. Primer-specific Tm and sequence information are provided in Table S6 in Additional file 1. Bisulfite MassArray assays were performed by the institutional Genomics Core Facility. The data were analyzed using the analytical pipeline we have previously described [24].

### Bioinformatic analysis

Four lanes of sequencing were performed using an Illumina GA IIx Sequencer at the institutional Epigenomics Shared Facility. Three lanes were used for technical replicates of MspI, for the methylation-insensitive reference dataset. Images generated by the Illumina sequencer were analyzed by Illumina pipeline software (versions 1.3 to 1.4). Initial data processing was performed using the default read length of 36 bp, after which we isolated the sequences in which we found adapter sequences on the 3'-end, replaced the adapter sequence with a poly(N) sequence of the same length, and re-ran the Illumina ELAND pipeline again on these sequences with the sequence length set at 27 bp (the 2 to 28 bp subsequence). The data within the ELAND_extended.txt files were used for counting the number of aligned sequences adjacent to each CCGG (HpaII/MspI) site annotated in the hg18 freeze of the human genome at the UCSC genome browser. We permitted up to two mismatches in each sequence, and allowed a sequence to align to up to a maximum of 10 locations within the genome. For non-unique alignments, a sequence was assigned a partial count for each alignment location amounting to 1/n, where n represents the total number of aligned positions. To normalize the data between experiments, the number of sequences associated with each HpaII site was divided by the total number of sequences (including partial counts) aligning to all HpaII sites in the same sample. We refer to this figure as the fixed count below.

To examine an influence of (G+C) mononucleotide content on counts of sequences obtained, we extracted the (G+C) annotation from the hg18 freeze of the human genome at UCSC and examined the distribution of sequence counts according to (G+C) content. Annotated percentages of (G+C) content were available for adjacent 5-bp windows. For each annotated HpaII site, we calculated the mean percentage (G+C) for a 50-bp region centered at the restriction site. Counts of sequences associated with HpaII sites were obtained for 50 sequential non-overlapping windows of 2% (G+C) (the minimum possible in a sample of 50-bp regions). These data were then normalized as a proportion of the total number of fragments. Comparisons were made to the expected frequencies, which for each 2% (G+C) bin was represented by the counts of HpaII sites falling within a range relative to the total number of HpaII sites in the genome. This analysis was performed on both HpaII and MspI-digested DNA for comparison.

The potential effect of distance between HpaII sites on sequences counts obtained at each HpaII site was measured by summing the counts of sequences aligning within each restriction fragment, and normalizing the result with respect to total sequence count. As with the (G+C) analysis above, this was performed for both MspI and HpaII digested restriction fragments. The data were compared with the expected distribution determined by performing virtual restriction digestion using genomic HpaII site coordinates, and normalizing the number of virtual fragments of each size with respect to the total number of these virtual fragments.

## Abbreviations

bp: base pair; CG/CpG: cytosine-guanine dinucleotide; ChIP: chromatin immunoprecipitation; ES: embryonic stem; (G+C): guanine and cytosine mononucleotides; HELP: HpaII tiny fragment Enrichment by Ligation-mediated PCR; MPS: massively-parallel sequencing; MSCC: methyl-sensitive cut counting; RRBS: reduced representation bisulfite sequencing; UCSC: University of California Santa Cruz.

## Authors' contributions

MS and JMG designed the assays and strategies for its analysis, MS performed all library preparation and characterization, MS, DL and MP performed bisulfite validation studies, while QJ and AMcL performed computational analyses. JMG and MS prepared the manuscript.

## Supplementary Material

Additional file 1 Supplemental data containing two figures (Figures S1 and S2) and nine tables (Tables S1 to S9). Click here for file
