# Supplementary material for: Optimized design and data analysis of tag-based cytosine methylation assays
Source: Genome Biol. 2010 Apr 1;11(4):R36. doi: 10.1186/gb-2010-11-4-r36 (PMC2884539; doi:10.1186/gb-2010-11-4-r36)
Supplement: Additional file 1 — Supplemental data containing two figures (Figures S1 and S2) and nine tables (Tables S1 to S9). [file gb-2010-11-4-r36-S1.PDF]

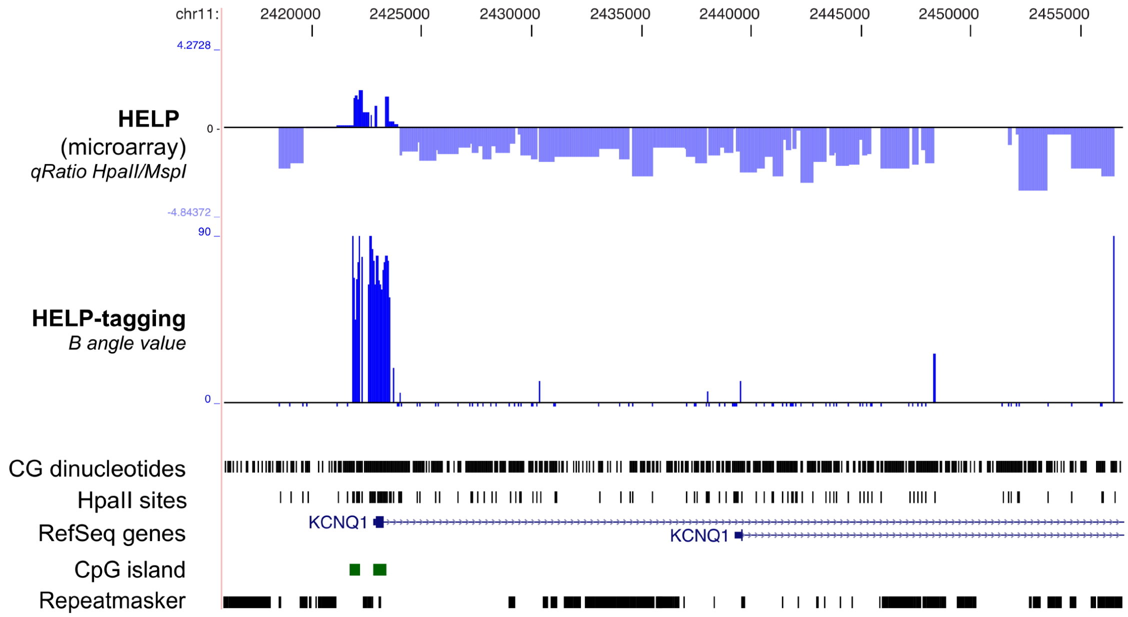


**Figure S1**

The UCSC genome browser representation of the B angle metric is shown for the promoters of the imprinted *KCNQ1* gene on human chromosome 11p15.5. Greater angle values indicating decresed methylation are shown as positive values. Loci at which MspI reads map to annotated HpaII/MspI sites without any HpaII representation (methylated sites) are shown as small negative values, as a zero value would fail to reveal that these loci were tested. The results of our microarray-based HELP assay on the same DNA sample are shown for comparison to illustrate the consistency between the assays.

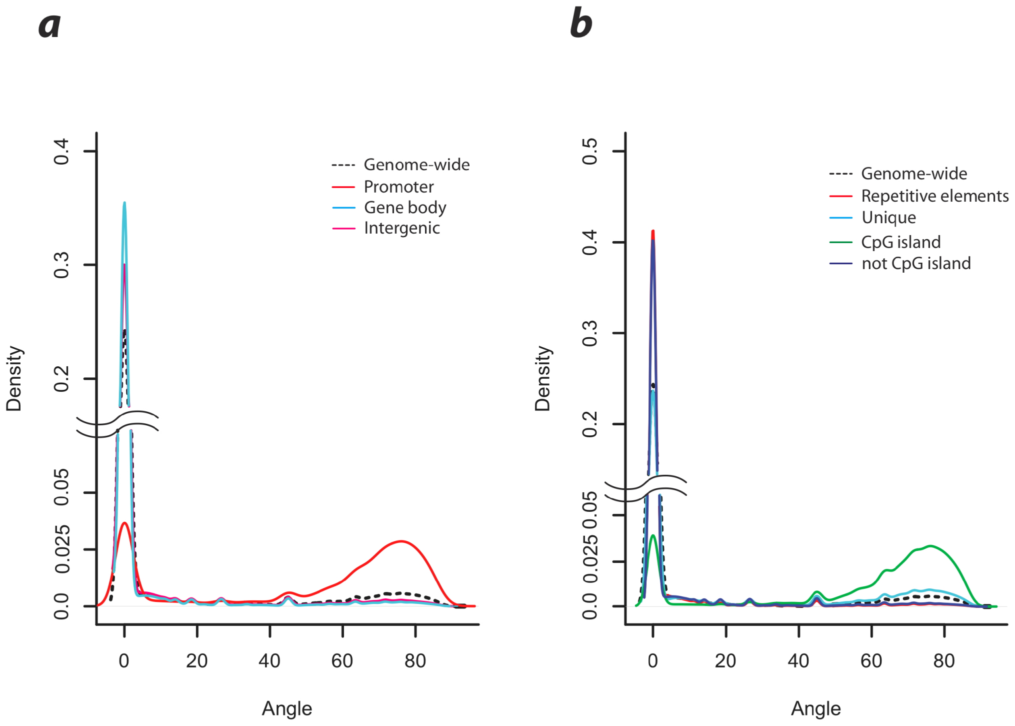


**Figure S2**

Angle value distributions are summarised as density plots for (a) promoters, gene bodies and intergenic regions, and (b) unique versus repetitive DNA, or CpG islands versus CG-depleted DNA, with the genome-wide average (dotted line) for reference. As expected, promoters and CpG islands are less methylated (a greater proportion of high angle values) than other sequences, while unique sequences are less methylated than repetitive.

## Table S1. HELP-tagging read number for each replicate and library

| Lane | Total # of reads | Aligned reads | | not matched | | Quality check failed | | Alignment>10 | |
|---|---|---|---|---|---|---|---|---|---|
| MspI_hES_1 1.4 | 5,987,967 | 4,302,009 | 71.84% | 294,967 | 4.93% | 12,783 | 0.21% | 1,378,208 | 23.02% |
| MspI_hES_2 1.3 | 7,153,197 | 3,870,062 | 54.10% | 2,434,289 | 34.03% | 76,470 | 1.07% | 772,376 | 10.80% |
| MspI_hES_3 1.3 | 7,552,369 | 4,010,827 | 53.11% | 2,618,175 | 34.67% | 150,307 | 1.99% | 773,060 | 10.24% |
| HpaII_hES 1.4 | 5,274,748 | 4,690,038 | 88.91% | 452,455 | 8.58% | 10,667 | 0.20% | 121,588 | 2.31% |
| MspI_hEP 1.4 | 7,292,523 | 5,341,135 | 73.24% | 367,585 | 5.04% | 13,400 | 0.18% | 1,570,403 | 21.53% |
| HpaII_hEP 1.4 | 5,917,385 | 5,147,885 | 87.00% | 476,864 | 8.06% | 11,056 | 0.19% | 281,580 | 4.76% |
| **Total** | 39,178,189 | 27,361,956 | 69.84% | 6,644,335 | 16.96% | 274,683 | 0.70% | 4,897,215 | 12.50% |

## Table S2. Correlation coefficiency between experimental replicates

| | MspI_hES_2 | MspI_hES_3 |
|---|---|---|
| **MspI_hES_1** | 0.9169 | 0.9149 |
| **MspI_hES_2** | | 0.9284 |
| **MspI_hES_3** | | |

**Table S3. Summary of read number with sequence features**

| | HpaII site | Analyzable* | | Hpa> 0 | | Average count | | | Angle > 60 | |
|---|---|---|---|---|---|---|---|---|---|---|
| | | site | percentage | site | percentage | Msp | HpaII | Angle | site | percentage |
| Total | 2,292,198 | 1,815,955 | 79.22 | 457,377 | 19.95 | 3.94 | 1.82 | 10.09 | 235,588 | 12.97 |
| In CpG island | 269,875 | 234,368 | 86.84 | 201,593 | 74.70 | 4.15 | 10.09 | 52.08 | 163,167 | 69.62 |
| Outside of CpG island | 2,022,319 | 1,600,419 | 79.14 | 389,880 | 19.28 | 3.94 | 1.72 | 9.58 | 195,467 | 12.21 |
| In repetitive element | 1,184,072 | 987,956 | 83.44 | 113,294 | 9.57 | 3.13 | 0.41 | 3.13 | 30,118 | 3.05 |
| Unique sequence | 1,108,126 | 827,999 | 74.72 | 344,083 | 31.05 | 4.81 | 3.33 | 17.54 | 205,470 | 24.82 |

*; Msp>0 or Msp=0 and Hpa>0

## Table S4. Bisulphite massarray validation assay information

| Assay name | Forward primer length | Reverse primer length | Sequence with conversion control |
|---|---|---|---|
| chr3.1 | 29 | 30 | TCACCTGTAATGTCAGCTTGAAAATGTCACGTTCTAGTTCCAGACAATATCCACAATATTAAAGCATTTTCGTTTAAAACCAAGAA<br>AAAAATATTTCAACCGGGTTTCAAGAGACTGATAAACCTAACCAGACTCACAATACAAAACAATTTCCTCCTATTTGACAATTCA<br>ATCCTTTTTTAAAGGAAATTTGGTGAGATGGCTGCAATTCTTTAGAAAGAAGTAGATTCAGAGATGTTTCAGTTTTGAAAATTAG<br>AATTTAGAGTTTAGGAGCTAAAACCAAGGAATATA |
| chr3.2 | 27 | 26 | AATTGTGACTTAAAGGCCAGGAGTGCCTCTTTTTACAAAGAAATGGTGCTAGTCAACCCCTTTACAAAATGCAAGCAATTTTGG<br>GGGAAGAGTCTTGAATCCGGAGATGAAAACGAGATCTTACTTTTGAAAGTGGTTTGGCCA |
| chr6.1 | 25 | 26 | TTAGGGCCTGGGTGCTGATTATTACCTTTACTGCTGTCTTTTTCAGGAGAAGCTGTTCACTTAGCTAGGGATTTTGGGTACATTTG<br>CGAAACGGAGTTTCCCGCCAAAGCCGTCTCTGAGTATTTGAACCGGCAGCACACAGACCCGAGTGACCTGCACTCCCGAAAGA<br>ATATGCTGTTGGCCACCAAGTGAGTTTATTAGACTCTGGGCCCTCATCTCACTCCCAGCTGGCTAGGCCACCCGATTTCGGAGTA<br>GGTGGTGGGGAGGGGCAGAGAAAGAAGCCAGAGGGTTGTC |
| chr6.2 | 27 | 26 | CTCATTTGAACTTTTACAGTGGGTTTTCTATTTATTTGATTTACTCTCGTCTTTGGCTTCGGTGTCATATTGTTGGTGTTTTATGGCC<br>GGCTCAGGCCTGCAGTGGGCGGCGCTGCGTGCGCCCGCACGTTTCTCTTGCAGGCGCGGCCCAAACAGCCGCGCTCATTTATT<br>TAGTGTTGGAGGACGCGGTTTGTCCCGTTGACAGCGTAATTTAAAGAAATATATGGAAGGCTGAAAAATCATTTGCTCAA |
| chr12.1 | 26 | 25 | CAGATGGTGATATCATTGATGGGTCCCGGAGAAGAACTATGCCAAAAGAGAGCAACAACAAAAAAATCAAGGTCAAGTGGCAT<br>AGAGGGCAAATCAGTATGGCATTTCAGTTTGCATTTAGAGACAGTCTGTGTATTTGCAAACAGGCCTGCCTTTAAGGGGAGTTA<br>ACCTTCTGTCTG |
| chr12.2 | 25 | 25 | AGAGGCCTAGGATGGCTGCCAGATACGGGCACTCATTCAGTTATTCACACACTCCTCGCGGTTGTAAATCCACAGAGCTGTACA<br>CTTTAGACTTGAGCAGCACATTGAGAGGGAGGGCTACTTACTGAAATAAAAGCCCCTGTCTCCACACACGAACTGAAGAGCAT<br>CCACCAGCTCAGCCCCGCAGAGCGTCTCCGGTCCAGCCGTGGCAGAGCTGGTGAAGGTGAGCAGGC |
| chr13.1 | 25 | 27 | TGACTCACTCAAGGGTTCTGATGATCGTTTCCCCTTCAGACATGCAGCCCAGATCTGCAATGGCTTCCTCACCCTTCAGACACAC<br>AGTCCAGATCTGCGATGGCTTCCTCACCCTTCAGACACACAGTCCAGGCCACTGAGATGCCTTCCTCACCCTTCAGACACACAG<br>TCCAGATCTGCGATACCTTCCACACCCTTCAGACACACAGTCCAGATCTGCGATACCTTCCACACCCTTCAGACACACAGTCCAG<br>GCCACCGAGATGCCTTCCTCACCCTTCAGACACACAGTCCAGATCTGCTATGCCTTCCTCACCCGGCAGACACACAGTCCAGAT<br>CTGCAATGCCTTCCTCATCCTTCAGACACACAGTCCAGACCTGTGATGCCTTCCTCACCCTTCAGACACACAGCCCAGGCCACT<br>GAGATGCCTTCCTCACTGATGAAATAA |
| chr13.2 | 26 | 27 | CCTGTGAATAGATGAGCAGCTTGAGGGAAGCGGTCCCACAGGACGGGCAAGCTTTCACGGAGGGGCTTTCCATTGACCATCCT<br>TTTCGCTTTGTCCTGTAGGTTGGGGACATTGTCATGGTTAAGGAGGACGAGACCTTTCCCTGCGACTTGATCTTCCTTTCCAGCA<br>ACCGGGGAGATGGGACGTGCCACGTCACCACCGCCAGCTTGGATGGAGAATCCAGCCATAAAGTAAGGGG |
| CBLC_PRO2 | 25 | 27 | cctgggcctCACCCCAGAGGACTTTGTTCTGGGGCGAAGGCTCACCTGAGTCTGGAGCCCGCCCGGAAGAGCTCGTCGTTGGCA<br>CTCCTTCGGCC(CCGG)GGAGGCAGCAGCGCGGCCACCTGCCTGCTCTTGGCCTCCAGATTGGCCAGGTAGATGAGTAGAAAGT<br>CC |
| CD14_PRO1 | 26 | 25 | AGACCTCTGGGGAAAGAACTATATGAAAGTCttttttttttttttttttttgagactgagtttggctctgttgcccaggctggagtgcagtggcgtgatctt<br>ggctcactgcaacctccgcctcccaagttcaagcaattctcttgcctcagattcctgagtagctgggattacaggagcccaccaccatgcccggctaatttttgtagtt<br>ttagtagagacagagtttcactatgttgaccaggcactcctgacctcaggtgatccgcccgccttggcctctcaaagtgctgggattacaggcgagagccaccaca<br>cccggccTGAAAGTCttttttacttttatttatttatttgagacggagttttgttcttgttgcccagg |
| CD14_PRO2 | 25 | 27 | GGAGAGAGCAGAGGTCTAGGAGGCCCCATCCAACCCCTGTGGCTCCCGAGTGGCACGCGTTCGACCCCAAGACCCTACACTCA<br>CCATGGTCGATAAGTCTTCCGAACCTCTGAGCTCCGGACAGGCTCTGGAAGTGCTTTAGCTTCTTTCCTACACAGCGGCACCCG<br>CCGGCTTCCAGGCTTCACACTTGTGAACTCTTCGGCTGCCTCTGACAGTTTATGTAATCCTGGGATG |
| RARA.2 | 27 | 27 | AATTATAGGATTCAGGGGCTTGACAGCTAGGGCAGGAGTCACCGCACTCACTTCCATATTACGCCGCCGCCTCACTTCTCAGATT<br>TAGGTGTGGGTGTGTGTGTGGTTGGGGGGAAAGGAGTGTAGGATACCACACGCTGCGGTCTTCTCCACCGAGCGCTATTTTCAT<br>TCTTTCCGCAGAACCTCACCCCGTTCTTGCTCTGAATCTTCGGTTCTGGGTCTGAGGGAGGGATTCTCCCGGATTCCCACGGTCC<br>AGTCTTCAACTAGGAGTGGCTCCTTTAAGA |
| SOCS.7 | 28 | 25 | CTTCTGCTGCAGCCCCCAGGCCCTGAATTACCTCCGGTGCCCTTCCCGCTGCAGGACTTGGTCCCTCTGGGGCGCCTGAGTAGA<br>GGGGagcagcagcagcagcagcagcagcaACCTCCCCCGCCCCCGCCTCCTCCCGGGCCCCTCCGGCCACTCGCGGGTCCTTCTCGG<br>AAGGGCTCCTTCAAAATCCGCCTCAGTCGCCTCTTTCGCACCAAGAGCTGCAACGGTGGCTCCGGCGGTGGGGATGGGACCGG<br>CAAGAGGCCTTCTGGAGAGCTGGCTGCTTCAGCTGCGAGCCTGACAGACATGGGAGGCTCT |
| HS1BP3.1 | 25 | 25 | GTGGGCAGGATGTGCCCTAGACCAAGCTTCTCCAACTGTAATGTGcgTGCAACCCCCTGGGGATCCTGTTAATATGCAGATTCTG<br>GTTCTGCAGGCCccggGGTGTGCCCTCACCATCTCTAGCAAATTCCCAAGTGATGTGGACACTGCTGTGTACTGCTCCAGAGACC<br>ATcgGAGAACATCCTAGGCTTcgCTGTCACATCTGCCCATCAGCCTGCAGCTGCTCCCTGATAGCTGCCCTCTCCTGGGACTGTAG<br>ACAAACTG |
| HS1BP3.2 | 26 | 27 | CCCAGCAGCTTAGGCTGAGAGACTTGCcgTTAGTCTTAACTTCTCTTCTTGTTCTCACACCCCATATCCAAGCATCAGCAAACCTG<br>AAAGCTCAATCTTCAAAATATATcgAGAACCCAAACATTTCTCACCCTCAcgGCcgCCATCACCCcgGGCCAAGCCACCACcgGCT<br>CTACATGGAAACCTACAAGCCTGTCCTGAATGCTCAGCTCGGCCACTGCCCATGCTCAGCATAGCAGCCAAAGGATCTCTATAAA<br>CCAGAAGTTGGGCCCTATTGCACTTGAAATTAAATCTGATGGCC |
| HS1BP3.3 | 25 | 25 | GGCTCCTGCTGTCCTGTCCTCCCAGTACACACTTTTCCAAGATTTCTTTTTCATGGCTCTCTCCCTCATGGAGCTTGTCcgGGATC<br>TGTCCAGGcgCCACTCCATCAGAAAGATGCCTCTTTACCACACCATAGAAAACAGCATGCATGCCTCCTACCACTCTGTTGCCCC<br>CACTGTTTTGTTTTCCTTCATAGCATGTATCATCCCTGACATGTcgTAGATTTGCCCACTGGGCCTGTGCCCTCATTAAAGCTCTAcg<br>AGAGGGACTTGTTTGTCTCCTAGAACTAGGGCCAGTACCcgGTACAGAGAAGGCTCTcgATAAACACCTTCTGGCTGAATTCAA<br>GCTTGAGTGCATGTGGCAATGACATGTGGGAGGAGATcgGGCTGGAAAGAGGAGCTGGGGTGcgACTGGGAAGGGCcgGCCT<br>TGCTGCTGGGCTAAGGcgTTTGGACTCACCCTGCAGGCAAGATCCTTGGGTGTGCTGGTGCCCTCAGCAAGAAATGAGCCTGA<br>CTCATGTGGCC |
| HS1BP3.4 | 25 | 25 | GGACCCCTCCAAGGCTTTTGCTGTAACTTGCGCTCTCCTGAGCTGCCTCGCAAGCAGCAACGACCTCTCCCTGGTCTCTCATCA<br>AACTCATCCTCCCAGAAACCGGGTTCCTGCCCTGTACTGTGTTCCCATGGCAACCTGAGCTTACTACCCGCTGCTGGCATCACCC<br>CCTCTCCGCTGCAGCCCCCTGCAGGGCGAGCTTGTCTGATTCGCCTCTGTCCCCAGTGCCCAGCACCGGGCCTGGGACTCAGG<br>GAGCTCTGAAAGGATGGGGAACTAGATTTTCGGGCTCAAAAGAGAAGAACCCAAGGCGGTAGGAAAGGAGAGCAGAGTCCC<br>C |
| DL1.1 | 26 | 26 | GGGAGCCCACTTAAGGCCTGTGTTACTGCCTTCCCCAGGGCCACGGAAACCTCTGCTCCAGGAATGGGGTAGGGAGGACAAG<br>GCCTCAGAGGGAACATCTCGCTGCAGGGAAGAGGGAGCGGCTGAGGGAAGCCCGGCCCCAGGTGTGGGAGAGACACTGGG<br>CAGCCACGTGGGGAAAGAGCAGAAAGTAGGTCAGTCACACAGGAGACGGAGCATGAAATTAATGAGCGTGCGGGAGCTGGA<br>GTAGTCACCTTCAGGGAA |
| DL1.2 | 25 | 25 | CCACAGTGGCAAACATGGGGAGAGAGGCACGTGTGTCTGCCTGATTGCAACAGGAACCGGGCCAGAGGCAGAAAGGCAGGG<br>AGAAGATGGACCAGTGAGGAGGAAAGATGAGGAGGTGCAGACACAGGTACTTGGTATGGGCACCATGTCTTGGCTCCCAGGT<br>TGG |

| | | | |
|---|---|---|---|
| DL1.3 | 26 | 26 | CCCAAGGGCTGAGCTTTGTCAACAAGGCCACGATACAGCTTAGACCAGAGAGAAGGAGCTGGAGGGACTGGGACTGGCTGTCCTCCCTCCTGGGGCCAGCCACATAAACAACCGGTTCCCTGGGACCGAGGCTGCTGCACAGGGAAAAGGGTAGGAGAGGGCAAAAAGTCACTCCC |
| DL1.4 | 26 | 26 | CAAAGTGTGGCAAAGCCCAAGGAGCCCGCGCCGCCTCTGCCCCTGCATGGCCCCAGACGGCTGGGGGTTTCCGGGCAGCCTGGAGTAGGATTTCAACATCTCAAGCTGGAGGTCACGTGCACAGAGCAGCCGGTGGCAAACGGGGAACTCCAGGAAGCCAGGCTGTTTCAGAGAAGTACTTGGAGCTGGGGGGCATTTAGGCAGGAGCCGAGTGAGGTCAGAGGGTCGGTGGCAGCCGTAGGCCTAACTTGCCAGAGAGGGAGGCCCAGT |
| DL1.5 | 26 | 28 | GCAGAGCCCAGAGCTGTATGGAGGAGGGACGAGGGTGGGCCTACCAGGACACGGCAGCTCCAGGCTCCTTTTAAGGAGGAATCCGTAAGTGGTTGTTAAGCTTGACTTCAGGCCTGGGGTGGGGGCAGGTTCTCATTGTCTTCAGCTCCTGTTTCTAGGCCCGGTCTTATGGCTTTTTAACCAAATAAGGCCAAGG |
| DL2.1 | 27 | 26 | CCAATTGGACCCAAAAGAAATGAATCTTCCTCAAAATGAATATCTGTTACTGAAGTGTACAATTAAAAAGACCTTACCCTAAACAGTCCCTCAGGAGTAACTGTACTACAGGAGAAAGAGAGCTGGGTTTCCAACTTAGAGGCCTGCATTCCGGTGTCAGAAATTAAGGATTTTGCTGTACAGCTTTGGACAGGTCATTCTTCATGTCTTGGC |
| DL2.2 | 26 | 27 | ATTACCAAGCATTGGCAAAAAGGTGGGGAAAGAGAGCCCTCACCCACTGCAGATGCAGCCGTTCTGAAGGACAATTGTGCACCTTAAATCCACCAAGCCCACTCCTGGGTATACACCCGGGGAAATTCTTCCCAGGGAACACAAGATG |
| DL2.3 | 26 | 26 | GCCTTCCTCAGCTGGGATAGGCCTCAGCTGGCGTGCTGAAGTTGGTAAAAGTCAGGCGTGTGTGAATCAGAGCCCGGGCCTCGCCACGGCGACGGCCAGGCTATTCATCACTGGGTGGTGGGTGACAGTGACTGATGGGCTAGGCCCGGGCCTGGCACTTCACCAGTGCAAGAATCATACGAGCCAGGGAGCAGAAAGACAGCTGGA |
| DL2.4 | 26 | 26 | AAAGGCAGAGCTGAAACAAGAACCTGACATCCAATCCGGAGTTCAGTTTTCACTAACATATGCACCTTGACTTCGCTTGAATTTCCTCCTTTTGATTGACAAAGAGTGAAAGTACTGGTGGCCTAATTTTATAAGAAACCACTGAAAATTCTTGAGAAAACTGGTTTACTGGTTCACAA |
| DL2.5 | 26 | 25 | AGCATAACTCAAGGGAGGGTCCAGAGGTCCTGCGGCTGGAGTGGAGTGAGCCGGAAAGAGGAAGGAAAGGTCACATGAAGAGCTGGGGCCAGGTCCTGATGGTAAGTGCTCTTTGGAACTCCTCCT |
| DL3.1 | 27 | 26 | GATTGTGGTGTTCATTTGTCTAGTCCAACTATTTTATAATAAACCGGGCTCAGGGATGTTCAATGAAATATTACATTCAAATTTACGTAACTGCATTTCTCTTGTAACAATAAACAATAATTTTCAAGGCATGAGAAAACTTCTGGACTTAC |
| DL3.2 | 26 | 26 | TCCAACTCCCAATGATTGGAGATGTTGATTTAGTACATCTGGAGTGGAACCGGGTAATATATTCTTTTTTTAAGCCTCTTATCTAATTCTGGTTAGAGATCAACTGTCTCAGACTGTCTCTCCCAGCTCTTGGTCATCTCCTCCATGATTTTGTCAGGGGCTAAACATTACCCAATTTAAGGTAAGCTAAAGAGTATTCCTGTTACAGATAGAAAAAAAAAAAAAGTTCATGGGTAGCAGTTCCCAGAAGGTGGGTATGAAAAGCCTAGC |
| DL3.4 | 28 | 26 | AGACACCAAGTATCTGAGAGGTAATGAGCGTGTGGGGTGAGAAGTGAGACTTTTTGAGTTGGATCCTCTGAACAGCTGGCTGGATTCGACAGGCACAGGGAATCCCAAGACCAGAGACTGGAATGCAGACATTCCTCCGGGTCTTAGGGCTTCTCTGGCAGAAAAACA |
| DL3.5 | 26 | 26 | GTACTGAATAAATACTGGGAGGGCCAGCGAGTCAGGGCCGGGGCTGACAGCACTCTTCTTAGAGTCTGTAAGTGGCCTGGACCTTCAAGCTGGAATGACAAGCATAATATCTGTGTCAGTGTACGTTATTCATCTGTCGTTAAGTCAGGGTCTGCAGGACAGTCCCCCACAGGTGGTG |
| DL3.6 | 28 | 26 | CCATTTTGAACTCAAAGAAGAAAATTGCTTGAATTTGCTTTTTTTTTTTTCTTTCTTTGTGAGACAGAGTCTCCGTCTGTTGCCTAGGCTGGAGTGCAGTGGCGACATCTCTGCTCACTGCAACCTCCGTCTCCTGGGTTCAAGCAGTTCTCATGCCTCAGCCCCACGTGTAGCTGGGATGACAGGTGTGCGCCACCATGCCCGGCTAATTTTTTGTATTTTTGGTAGAGACAGGGTTTCACCATGTTGGCCAGG |
| DL4.1 | 27 | 25 | CCATGCCTGGCTAATTTTTGTATTTTTTGTAGAGACAGGGTTTCGCCATGTAGCCCAGGCTGGTCTGGAACTCCTGGCCTCAAATGATCCACCCACCTCGGCTTCCCACAGAGCTGGGATTACAGACGTGAGCCACCACACCCAGCCACATTGTACCTTTTGAAAGAAGCAGCAGGGAGAGTAGCTGGGAAGCTAGACTAGGGCCAGATCCGGAGAGGCTTTGGATGCCAGGGGAGGCATCTGCATATATTTCAGTAGGCAAGAGGTGGAGAACGTGAGACACTTCCAAACAGGAAAGGGGATGCAAGCCCCA |
| DL4.2 | 26 | 26 | GGGGTGAGCAAGAAGCAGCAGGAGTACACCTCAGCTCCAAGAGAATATGCCACCTGAGCATACACTGTCCCCACACCGGCCAGCTCCTCCCCTTCCCCACCCCAAGCTGATGAATCGGGTTTGTTGACATTTGGGGCCTAGTCCCATGAACAAAGCTAAATTCCCTGAAGCGCGCTGTCTCCCAAGACCTTTCCCTTGAATGCTACAGCATCTGCTCCTGCCAAACCTCACTCCCATGTTCCACTTCCAAAAGGGGAA |
| DL4.3 | 25 | 25 | TGCTGTTTCTGCTGCCAGGAATGTCCTTCCCGTGGAGCCGGCCTGGGTGATCCTCTGCTGAGAAGCTTTTCACGATCAGCTCCTCCTCCCTGTAATCTCACTGCACCCAGCCCGCGTGTGTGTTTTAGTATTTATTGCACCTGAGGGCACTTTGTACACATTATATTGGGGTTTATTTCGTCTGTTTGCAACTAGACTAAGCTCTGTGAGGACTGGATCT |
| DL4.4 | 26 | 26 | GCTGGTTCAAATGGCCCTGCAGTGCATCAGCTGGCTAAGCCCATCCAGGATTTAAGCCATTTAAAAACCTCTTTGAAAAACACTTAACTAAATTCCCTTCACAGGAGCACAGTGCTGTCACAGAGCTCTGAGCAGCATTTAATATATTTTGTCTCACATCTTGGTTTTCCCACAAGCTGTCGCCCGGTTAGCCTTCTGTGCAGCGGGAGCAAGACTTTGCACAGTGGCGGGAGGACTCGGGAAGCCCTGAGCCAGCCACTAGAGGGGGATAGGATCTATT |
| DL4.5 | 26 | 27 | TGCCCTTGTGTCAGAATACTAAGGTGGGAGGCGGAGCGGAGGTGCCAGTCACTCTGGGCGAGGCCTTACTGCGCCCATGCATCCCGGGCCCATTTGTCAGCCCTTGCTCTTCAGTGGGAGCCCAGCTGCAAGCCCAGAGTCTG |
| DL5.1 | 26 | 27 | ATGTGATCTGTTCCCTCTGTCTCCAGGCGATGGCAGTGCCAAGAAGAGGTCCTGAGGGAGTTTTGTGTTTTGAGGAGAGTAGACTGCTTCCCTATCCAAAATACAAAGCTCTGTTTATTCATTTATTTACCCAGCACTGAGCACAGGGGCTGCCACAGTGGCCGGCTCAGACATGGCAGAGATCAGCAGAGAATGGG |
| DL5.2 | 26 | 26 | CCTCCTAAAGTGCTGGGATTACAGCTGTGAGCCACCCTGCCCGGCCACTTTTGTATGATTTCTAATGTATTTGTAATTTACCTAACAAATTGCCTAATCTGCTATGTTAATGTATTTATGAATTAAAATAAATACGACTGCATGTTTGTGGTTCATTTTTGTGGAGGTGGCTGTGGTGACATCAGCCAAGAATCTGAATGGTACTGTTGA |
| DL5.3 | 27 | 26 | ATGTGTGTTCTGCCCCTTGCAATAAAAGTTCTGCCTAAAACCAGCCTCAATCAAGGGTCAGAGTCAAACTCCAGAAGGGAAGCAAGACCTGGAAAGGAGAAGCGAAGCAGCAGAGCTGAGGTGCCGGGAATCCTGGGCAGGTGCGCAGGGCGAGGGTGGGCGGGGCCGTGGGCGGTGCTAGAGGCGGGCCCGACTATGCCAATCTGGGGCGCCGGCCAATGGGAGCCCCTGTGTGTCCTGTCTTAGGATGGACCAGGTATCTTCT |
| DL5.4 | 26 | 26 | GATTGAATGGGGATGTTAATTAATACCTTTGTTCTTAAGAATCAGCAGGGTTCCAGGAAGAAGGGCAAGACCCCGGAGCTGGGTCAGAGGTACAAGGTAGGAACCTAGTTCCTAGTTCCTAGAAATAAGAAATGTCTTAGGAATCAATGTGGATCCTCAGAGTGGATTCAGAAGCCAGAGATTCCTTAAGTTCTCTCTGGCTAGGGTCAG |
| DL5.5 | 25 | 28 | ATTAGCTGGGCATGGTGGCACATGCCTGTAATCCCAGCTACTCGATAGGCTGAGAATTGTTTGAACCCGGGAGGCGGAGGTTGTGGTGAGTCGAGATTACGCCATTGCACTCCAGCCTGGGCGCCAAGAGCAAAACTTCGTCTCAAAAAAAAAAAAACAAAGCCGACCCAAACTGTCCAAGATTCCCCTCAC |

## Table S5. Bisulphite massarray validation

| Assay name | Methylation | chr* | Position* | CGI** | RepMask*** | RefSeq | | | | Distance fix¶ | hES Msp fix¶ | hES Hpa fix¶ | Angle | HpaII/MspI |
|---|---|---|---|---|---|---|---|---|---|---|---|---|---|---|
| | | | | | | Promoter | RefSeqID | Genebody | RefSeqID | | | | | |
| DL4.1 | 0.6972642 | chr1 | 20536572 | No | No | No | | No | | 1.427 | 0.7745 | 1.1989 | 35.5377 | 1.5481 |
| DL5.2 | 0.6139191 | chr1 | 28708634 | No | Yes | No | | Yes | NM_001048198,NM_001048199 | 1.034 | 0.3872 | 0.9591 | 48.8141 | 2.4770 |
| DL5.3 | 0.8449592 | chr1 | 29555984 | No | No | No | | No | | 1.244 | 0.3319 | 1.1989 | 59.0362 | 3.6122 |
| DL5.1 | 0.4660534 | chr1 | 32477585 | No | No | No | | No | | 0.528 | 0.2213 | 0.4796 | 45.0000 | 2.1673 |
| DL3.6 | 0.9292852 | chr1 | 40518845 | No | Yes | No | | Yes | NM_005857 | 0.252 | 0.2213 | 0.1199 | 14.0362 | 0.5418 |
| DL1.3 | 0.90618035 | chr1 | 40900248 | No | No | No | | Yes | NM_014747 | 1.105 | 0.9957 | 0.4796 | 12.5288 | 0.4816 |
| DL2.1 | 0.868192225 | chr1 | 41486246 | No | Yes | No | | No | | 0.503 | 0.4425 | 0.2398 | 14.0362 | 0.5418 |
| DL1.1 | 0.87562555 | chr1 | 41516023 | No | No | No | | No | | 0.706 | 0.6638 | 0.2398 | 9.4623 | 0.3612 |
| DL1.2 | 0.89757185 | chr1 | 41529849 | No | No | No | | No | | 0.811 | 0.7745 | 0.2398 | 8.1301 | 0.3096 |
| DL1.4 | 0.7647495 | chr1 | 41977513 | No | No | No | | Yes | NM_024503,NM_001127714 | 0.706 | 0.6638 | 0.2398 | 9.4623 | 0.3612 |
| DL4.2 | 0.8233482 | chr1 | 207603205 | No | No | No | | No | | 0.845 | 0.4425 | 0.7194 | 36.8699 | 1.6255 |
| DL5.4 | 0.6697093 | chr1 | 207776917 | No | No | No | | No | | 3.116 | 1.6596 | 2.6377 | 36.2538 | 1.5894 |
| HS1BP3.1 | 0.65 | chr2 | 20711115 | No | Yes | No | | Yes | NM_022460 | 0.332 | 0.3319 | 0.0000 | 0.0000 | 0.0000 |
| HS1BP3.2 HpaII1 | 0.87 | chr2 | 20712258 | No | Yes | No | | Yes | NM_022460 | 0.332 | 0.3319 | 0.0000 | 0.0000 | 0.0000 |
| HS1BP3.2 HpaII2 | 0.885 | chr2 | 20712274 | No | Yes | No | | Yes | NM_022460 | 0.000 | 0.0000 | 0.0000 | 0.0000 | NA |
| HS1BP3.3 HpaII1 | 0.94 | chr2 | 20712606 | No | Yes | Yes | NM_022460 | No | | 0.221 | 0.2213 | 0.0000 | 0.0000 | 0.0000 |
| HS1BP3.3 HpaII2 | 0.9 | chr2 | 20712825 | No | Yes | Yes | NM_022460 | No | | 0.996 | 0.9957 | 0.0000 | 0.0000 | 0.0000 |
| HS1BP3.4 HpaII1 | 0.17 | chr2 | 20713766 | No | No | Yes | NM_022460 | No | | 1.767 | 0.5532 | 1.6785 | 54.4623 | 3.0343 |
| HS1BP3.4 HpaII2 | 0.125 | chr2 | 20713898 | No | Yes | Yes | NM_022460 | No | | 1.443 | 0.1106 | 1.4387 | 80.5377 | 13.0040 |
| DL1.5 | 0.61619005 | chr2 | 28683576 | No | No | No | | Yes | NM_153021 | 0.503 | 0.4425 | 0.2398 | 14.0362 | 0.5418 |
| DL2.2 | 0.55422885 | chr3 | 127151054 | Yes | Yes | No | | No | | 0.819 | 0.6638 | 0.4796 | 18.4349 | 0.7224 |
| chr3.1 | 0.04 | chr3 | 187135021 | Yes | No | No | | Yes | NM_004593 | 1.931 | 0.2213 | 1.9183 | 75.9638 | 8.6693 |
| chr3.2 | 0.06 | chr3 | 187135718 | No | No | No | | Yes | NM_004593 | 1.921 | 0.1106 | 1.9183 | 82.8750 | 17.3387 |
| DL2.5 | 0.73619675 | chr4 | 40369411 | No | Yes | No | | No | | 0.819 | 0.6638 | 0.4796 | 18.4349 | 0.7224 |
| DL2.3 | 0.8303393 | chr4 | 85465182 | No | No | No | | No | | 1.414 | 1.2170 | 0.7194 | 15.2551 | 0.5911 |
| DL2.4 | 0.771100675 | chr4 | 106107466 | No | No | No | | No | | 1.608 | 1.4383 | 0.7194 | 12.9946 | 0.5002 |
| DL3.4 | 0.6719508 | chr5 | 5447869 | No | No | No | | No | | 0.380 | 0.2950 | 0.2398 | 20.5560 | 0.8128 |
| DL3.5 | 0.5150264 | chr5 | 37122600 | No | Yes | No | | No | | 1.383 | 0.9957 | 0.9591 | 23.9625 | 0.9633 |
| CD14_GB1 | | chr5 | 139992172 | No | No | Yes | NM_000591,NM_001040021 | No | | 0.553 | 0.5532 | 0.0000 | 0.0000 | 0.0000 |
| CD14_PRO2 HpaII1 | 0.985 | chr5 | 139992872 | No | No | Yes | NM_000591,NM_001040021 | No | | 1.106 | 1.1064 | 0.0000 | 0.0000 | 0.0000 |
| CD14_PRO2 HpaII2 | 0.94 | chr5 | 139992923 | No | No | Yes | NM_000591,NM_001040021 | No | | 0.664 | 0.6638 | 0.0000 | 0.0000 | 0.0000 |
| CD14_PRO1 HpaII1 | 0.89 | chr5 | 139994416 | No | Yes | Yes | NM_000591,NM_001040021 | No | | 0.000 | 0.0000 | 0.0000 | 0.0000 | NA |
| CD14_PRO1 HpaII2 | 0.89 | chr5 | 139994542 | No | Yes | Yes | NM_000591,NM_001040021 | No | | 0.664 | 0.6638 | 0.0000 | 0.0000 | 0.0000 |
| chr6.1 | 0.05 | chr6 | 50915912 | No | No | No | | Yes | NM_003221 | 0.984 | 0.2213 | 0.9591 | 63.4349 | 4.3347 |
| chr6.2 | 0.08 | chr6 | 50916587 | No | No | No | | Yes | NM_003221 | 1.456 | 0.2213 | 1.4387 | 71.5651 | 6.5020 |
| DL4.3 | 0.44898065 | chr7 | 2627533 | Yes | No | No | | No | | 0.583 | 0.3319 | 0.4796 | 33.6901 | 1.4449 |
| DL4.5 | 0.583525325 | chr7 | 44070331 | No | No | Yes | NM_000290 | No | | 3.117 | 1.9915 | 2.3979 | 29.0546 | 1.2041 |
| DL3.1 | 0.47480925 | chr7 | 109831133 | No | No | No | | No | | 0.326 | 0.2213 | 0.2398 | 26.5651 | 1.0837 |
| DL5.5 | 0.4660534 | chr7 | 129365707 | No | Yes | No | | Yes | NM_003344,NM_182697 | 0.177 | 0.1438 | 0.1039 | 18.4349 | 0.7224 |
| DL4.4 | 0.6567495 | chr7 | 129705231 | Yes | No | No | | Yes | NM_001869 | 0.979 | 0.6638 | 0.7194 | 26.5651 | 1.0837 |
| DL3.2 | 0.854180875 | chr7 | 133432507 | No | No | No | | No | | 0.732 | 0.5532 | 0.4796 | 21.8014 | 0.8669 |
| KCNQ1_1 HpaII1 | 0.89 | chr11 | 2438800 | No | No | Yes | NM_181798 | Yes | NM_000218 | 0.221 | 0.2213 | 0.0000 | 0.0000 | 0.0000 |
| KCNQ1_1 HpaII2 | 0.895 | chr11 | 2438810 | No | No | Yes | NM_181798 | Yes | NM_000218 | 0.111 | 0.1106 | 0.0000 | 0.0000 | 0.0000 |
| KCNQ1_2 HpaII1 | 0.92 | chr11 | 2439171 | No | No | Yes | NM_181798 | Yes | NM_000218 | 0.885 | 0.8851 | 0.0000 | 0.0000 | 0.0000 |
| KCNQ1_2 HpaII2 | 0.925 | chr11 | 2439222 | No | No | Yes | NM_181798 | Yes | NM_000218 | 0.000 | 0.0000 | 0.0000 | 0.0000 | NA |
| KCNQ1_2 HpaII3 | 0.925 | chr11 | 2439226 | No | No | Yes | NM_181798 | Yes | NM_000218 | 0.221 | 0.2213 | 0.0000 | 0.0000 | 0.0000 |
| KCNQ1_2 HpaII4 | 0.925 | chr11 | 2439293 | No | No | Yes | NM_181798 | Yes | NM_000218 | 2.102 | 2.1021 | 0.0000 | 0.0000 | 0.0000 |
| KCNQ1_2 HpaII5 | 0.875 | chr11 | 2439356 | No | No | Yes | NM_181798 | Yes | NM_000218 | 1.328 | 1.3276 | 0.0000 | 0.0000 | 0.0000 |
| KCNQ1_3 | 0.8383 | chr11 | 2439532 | No | No | Yes | NM_181798 | Yes | NM_000218 | 0.603 | 0.5532 | 0.2398 | 11.3099 | 0.4335 |
| chr12.1 | 0.865 | chr12 | 101392898 | Yes | No | No | | Yes | NM_001111284,NM_001111283 | 0.332 | 0.3319 | 0.0000 | 0.0000 | 0.0000 |
| chr12.2 | 0.99 | chr12 | 101393622 | Yes | No | No | | Yes | NM_001111284,NM_001111283 | 0.000 | 0.0000 | 0.0000 | 0.0000 | NA |
| chr13.1 | 0.765 | chr13 | 112517735 | No | No | No | | Yes | NM_032189,NM_015205 | 0.000 | 0.0000 | 0.0000 | 0.0000 | NA |
| chr13.2 | 0.88 | chr13 | 112518468 | No | No | No | | Yes | NM_032189,NM_015205 | 0.553 | 0.5532 | 0.0000 | 0.0000 | 0.0000 |
| SOCS.7 HpaII1 | 0.115 | chr17 | 33762335 | No | No | Yes | NM_014598 | No | | 2.638 | 0.0000 | 2.6377 | 90.0000 | NA |
| SOCS.7 HpaII2 | 0.09 | chr17 | 33762353 | No | No | Yes | NM_014598 | No | | 5.555 | 0.6638 | 5.5151 | 75.3791 | 8.3081 |
| RARA.2 | 0.02 | chr17 | 35727828 | No | No | No | | Yes | NM_000964 | 0.959 | 0.0000 | 0.9591 | 90.0000 | NA |
| CBLC_PRO2 HpaII1 | 0.3 | chr19 | 49973330 | No | No | Yes | NM_012116 | No | | 3.083 | 1.1064 | 2.8774 | 50.1944 | 2.6008 |
| CBLC_PRO2 HpaII2 | 0.12 | chr19 | 49973363 | No | No | Yes | NM_012116 | No | | 3.914 | 0.7745 | 3.8366 | 66.3706 | 4.9539 |
| H19 | 0.8495846 | chr11 | 1975339 | No | No | No | | No | | 1.106 | 1.1064 | 0.0000 | 0.0000 | 0.0000 |
| DLK1 | 0.8803369 | chr14 | 100266902 | No | No | No | | Yes | NM_003836 | 0.332 | 0.3319 | 0.0000 | 0.0000 | 0.0000 |
| DLX5 | 0.06496035 | chr7 | 96493438 | No | No | Yes | NM_005221 | No | | 1.918 | 0.0000 | 1.9183 | 90.0000 | NA |

*; UCSC genome browser hg18 (NCBI assembly 36.1)
**; In CpG island or not
***; In repeatmasker-annotated regions (http://www.repeatmasker.org) or not
¶; number was normalised to total read number per lane
RefSeq; NCBI RNA reference sequences collection (http://www.ncbi.nlm.nih.gov/RefSeq/)

## Table S6. Bisulphite massarray validation primers

| Assay # | Forward PCR primer | Tm (GSP only) | Reverse PCR primer | Tm (GSP only) |
|---|---|---|---|---|
| CHR3.1 | TTATTTGTAATGTTAGTTTGAAAATGTTA | 56.28 | TATCTTTTTATATTCCTTAATTTTAACTCC | 56.22 |
| CHR3.2 | AATTGTGATTTAAAGGTTAGGAGTGTT | 58.98 | CTAACCAAACCACTTTCAAAAATAAA | 59.05 |
| CHR6.1 | TTAGGGTTTGGGTGTTGATTATTAT | 59.05 | AACAACCCTCTAACTTCTTTCTCTAC | 57.11 |
| CHR6.2 | TTTATTTGAATTTTTATAGTGGGTTTT | 57.17 | TTAAACAAATAATTTTTCAACCTTCC | 58.22 |
| CHR12.1 | TAGATGGTGATATTATTGATGGGTTT | 59.03 | CAAACAAAAAATTAACTCCCCTTAA | 59 |
| CHR12.2 | AGAGGTTTAGGATGGTTGTTAGATA | 56.73 | ACCTACTCACCTTCACCAACTCTAC | 59.2 |
| CHR13.1 | TGATTTATTTAAGGGTTTTGATGAT | 57.29 | TTATTTCATCAATAAAAAAAACATCTC | 56.47 |
| CHR13.2 | TTTGTGAATAGATGAGTAGTTTGAGG | 58.06 | CCCCTTACTTTATAACTAAATTCTCCA | 58.86 |
| DL1.1 | GGGAGTTTATTTAAGGTTTGTGTTAT | 57.41 | TTCCCTAAAAATAACTACTCCAACTC | 57.67 |
| DL1.2 | TTATAGTGGTAAATATGGGGAGAGA | 57.36 | CCAACCTAAAAACCAAAACATAATA | 57.73 |
| DL1.3 | TTTAAGGGTTGAGTTTTGTTAATAAG | 56.62 | AAAAATAACTTTTTACCCTCTCCTAC | 56.27 |
| DL1.4 | TAAAGTGTGGTAAAGTTTAAGGAGTT | 57.69 | ACTAAACCTCCCTCTCTAACAAATTA | 57.69 |
| DL1.5 | GTAGAGTTTAGAGTTGTATGGAGGAG | 56.22 | CCTTAACCTTATTTAATTAAAAAAACCAT | 57.27 |
| DL2.1 | TTAATTGGATTTAAAAGAAATGAATTT | 57.21 | ACCAAAACATAAAAAATAACCTATCC | 57.32 |
| DL2.2 | ATTATTAAGTATTGGTAAAAAGGTGG | 55.64 | CATCTTATATTCCCTAAAAAAAATTTC | 56.56 |
| DL2.3 | GTTTTTTTTAGTTGGGATAGGTTTTA | 57.58 | TCCAACTATCTTTCTACTCCCTAACT | 57.41 |
| DL2.4 | AAAGGTAGAGTTGAAATAAGAATTTG | 55.9 | TTATAAACCAATAAACCAATTTTCTC | 56.36 |
| DL2.5 | AGTATAATTTAAGGGAGGGTTTAGAG | 56.25 | AAAAAAAATTCCAAAAAACACTTAC | 56.45 |
| DL3.1 | GATTGTGGTGTTTATTTGTTTAGTTTA | 57.11 | ATAAATCCAAAAATTTTCTCATACCT | 57.07 |
| DL3.2 | TTTAATTTTTAATGATTGGAGATGTT | 56.7 | ACTAAACTTTTCATACCCACCTTCTA | 57.99 |
| DL3.3 | TTAAATTAAAAGAAATAAGTTTTGTGAT | 55.05 | AACCAAACTAATCTTAAACTCCTAAC | 55.13 |
| DL3.4 | AGATATTAAGTATTTGAGAGGTAATGAG | 54.55 | TATTTTTCTACCAAAAAAACCCTAAA | 57.98 |
| DL3.5 | GTATTGAATAAATATTGGGAGGGTTA | 57.95 | CACCACCTATAAAAAACTATCCTACA | 57.11 |
| DL3.6 | TTATTTTGAATTTAAAGAAGAAAATTGT | 56.75 | CCTAACCAACATAATAAAACCCTATC | 57.69 |
| DL4.1 | TTATGTTTGGTTAATTTTTGTATTTTT | 56.1 | TAAAACTTACATCCCCTTTCCTATT | 57.83 |
| DL4.2 | GGGGTGAGTAAGAAGTAGTAGGAGTA | 57.73 | TTCCCCTTTTAAAAATAAAACATAAA | 57.89 |
| DL4.3 | TGTTGTTTTTGTTGTTAGGAATGTT | 58.98 | AAATCCAATCCTCACAAAACTTAAT | 58.51 |
| DL4.4 | GTTGGTTTAAATGGTTTTGTAGTGTA | 57.77 | AATAAATCCTATCCCCCTCTAATAAC | 57.69 |
| DL4.5 | TGTTTTTGTGTTAGAATATTAAGGTG | 56.02 | CAAACTCTAAACTTACAACTAAACTCC | 55.71 |
| DL5.1 | ATGTGATTTGTTTTTTTTGTTTTTAG | 57.19 | CCCATTCTCTACTAATCTCTACCATAT | 56.82 |
| DL5.2 | TTTTTTAAAGTGTTGGGATTATAGTT | 56.11 | TCAACAATACCATTCAAATTCTTAAC | 57.77 |
| DL5.3 | ATGTGTGTTTTGTTTTTTGTAATAAAA | 57.46 | AAAAAATACCTAATCCATCCTAAAAC | 56.73 |
| DL5.4 | GATTGAATGGGGATGTTAATTAATAT | 57.79 | CTAACCCTAACCAAAAAAAACTTAAA | 57.73 |
| DL5.5 | ATTAGTTGGGTATGGTGGTATATGT | 56 | ATAAAAAAAATCTTAAACAATTTAAATC | 54.08 |

## Table S7. Alignment statistics for EcoP15I and simulated MmeI reads

| | 1-10 matches | >10 matches | No matches |
|---|---|---|---|
| EcoP15I 27 bp | 71.84% | 23.02% | 4.93% |
| Simulated MmeI 19 bp | 1.08% | 98.86% | 0.00% |

**Table S8. Putative polymorphic HpaII sites**

| Polymorphic HpaII site | | | | dbSNP¶ | | | |
|---|---|---|---|---|---|---|---|
| Chromosome | Position | Left weighted count* | Right weighted count* | Chromosome | ChromStart | dbSNPs | observed |
| chr1 | 886339 | 2 | 2 | chr1 | 886338 | rs28393498 | A/G |
| chr1 | 899418 | 10 | 4 | chr1 | 899417 | rs2340594 | C/T |
| chr1 | 904740 | 2 | 2 | chr1 | 904738 | rs13302983 | C/T |
| chr1 | 972859 | 8 | 1 | chr1 | 972856 | rs10267 | C/T |
| chr1 | 1050039 | 5 | 3 | chr1 | 1050036 | rs7548798 | C/T |
| chr1 | 1050039 | 5 | 3 | chr1 | 1050037 | rs7556082 | C/T |
| chr1 | 1052907 | 6 | 2 | chr1 | 1052906 | rs7545801 | A/G |
| chr1 | 1054400 | 2 | 2 | chr1 | 1054397 | rs6682475 | C/G |
| chr1 | 1066928 | 0 | 0 | chr1 | 1066926 | rs4970357 | A/C |
| chr1 | 1069055 | 6 | 1 | chr1 | 1069053 | rs11260602 | C/T |
| chr1 | 1076043 | 7 | 5 | chr1 | 1076041 | rs61766352 | C/T |
| chr1 | 1087151 | 4 | 2 | chr1 | 1087149 | rs9442384 | C/T |
| chr1 | 1229202 | 3 | 6 | chr1 | 1229201 | rs11260584 | G/T |
| chr1 | 1237358 | 6 | 2 | chr1 | 1237356 | rs12103 | C/T |
| chr1 | 1479937 | 2 | 2 | chr1 | 1479936 | rs7366635 | A/G |
| chr1 | 1605212 | 5 | 1 | chr1 | 1605210 | rs7520934 | A/C |
| chr1 | 1608538 | 7 | 3 | chr1 | 1608537 | rs28541555 | G/T |
| chr1 | 2028117 | 17 | 2 | chr1 | 2028115 | rs61775411 | C/T |
| chr1 | 2195409 | 3 | 4 | chr1 | 2195407 | rs12047069 | A/C |
| chr1 | 2237347 | 3 | 1 | chr1 | 2237346 | rs2645076 | A/G |
| chr1 | 2249910 | 0 | 0 | chr1 | 2249909 | rs2645063 | A/G |
| chr1 | 2401311 | 6 | 1 | chr1 | 2401310 | rs10797428 | A/G |
| chr1 | 2444570 | 3 | 1 | chr1 | 2444568 | rs2494625 | A/G |
| chr1 | 2711436 | 3 | 9 | chr1 | 2711435 | rs897623 | C/T |
| chr1 | 2731561 | 4 | 3 | chr1 | 2731559 | rs897622 | A/G |
| chr1 | 2986463 | 3 | 4 | chr1 | 2986461 | rs1569419 | A/G |
| chr1 | 3028483 | 1 | 5 | chr1 | 3028482 | rs2742672 | C/T |

| | | | | | | | |
|---|---|---|---|---|---|---|---|
| chr1 | 3200810 | 4 | 3 | chr1 | 3200809 | rs4648468 | A/G |
| chr1 | 3336852 | 0 | 0 | chr1 | 3336851 | rs10909944 | A/G |
| chr1 | 3455556 | 7 | 1 | chr1 | 3455555 | rs7539836 | A/G |
| chr1 | 3471210 | 3 | 1 | chr1 | 3471208 | rs12756207 | C/T |
| chr1 | 3486340 | 8 | 2 | chr1 | 3486338 | rs2794340 | A/G |
| chr1 | 3508805 | 3 | 3 | chr1 | 3508803 | rs7521546 | C/T |
| chr1 | 3517803 | 6 | 4 | chr1 | 3517803 | rs10797402 | C/G |
| chr1 | 3536898 | 3 | 2 | chr1 | 3536896 | rs34946124 | C/G |
| chr1 | 3759091 | 4 | 8 | chr1 | 3759090 | rs6657021 | A/G |
| chr1 | 3779675 | 6 | 7 | chr1 | 3779673 | rs66849805 | A/C |
| chr1 | 3830890 | 3 | 1 | chr1 | 3830887 | rs35314156 | C/G |
| chr1 | 4181671 | 1 | 5 | chr1 | 4181669 | rs4233265 | C/T |
| chr1 | 4627103 | 11 | 1 | chr1 | 4627102 | rs6663166 | A/G |
| chr1 | 4708830 | 1 | 14 | chr1 | 4708829 | rs433427 | C/T |
| chr1 | 4809669 | 1 | 3 | chr1 | 4809667 | rs7543326 | C/T |
| chr1 | 5240678 | 3 | 4 | chr1 | 5240677 | rs35263239 | A/G |
| chr1 | 5728407 | 4 | 2 | chr1 | 5728405 | rs9724874 | C/T |
| chr1 | 5793727 | 1 | 3 | chr1 | 5793727 | rs28411274 | A/G |
| chr1 | 5894042 | 6 | 1 | chr1 | 5894041 | rs6694744 | A/G |
| chr1 | 5910284 | 3 | 1 | chr1 | 5910282 | rs7520105 | C/T |
| chr1 | 6106678 | 5 | 2 | chr1 | 6106678 | rs2843493 | C/T |
| chr1 | 6109548 | 1 | 14 | chr1 | 6109547 | rs2746060 | C/T |
| chr1 | 6238804 | 9 | 8 | chr1 | 6238803 | rs6703384 | A/G |
| chr1 | 6506611 | 1 | 3 | chr1 | 6506610 | rs4908557 | A/G |
| chr1 | 7133139 | 2 | 6 | chr1 | 7133138 | rs6693832 | A/G |
| chr1 | 7442833 | 2 | 3 | chr1 | 7442833 | rs34328009 | C/G |
| chr1 | 7449730 | 1 | 4 | chr1 | 7449728 | rs1193234 | A/G |
| chr1 | 7488961 | 3 | 2 | chr1 | 7488960 | rs12059554 | A/G |
| chr1 | 7661838 | 7 | 4 | chr1 | 7661836 | rs4908678 | C/T |
| chr1 | 8299498 | 2 | 2 | chr1 | 8299497 | rs2083906 | C/T |
| chr1 | 9345565 | 4 | 2 | chr1 | 9345563 | rs553161 | A/G |
| chr1 | 9588951 | 1 | 11 | chr1 | 9588950 | rs7364899 | A/G |

| | | | | | | | |
|---|---|---|---|---|---|---|---|
| chr1 | 10726454 | 1 | 5 | chr1 | 10726453 | rs12026593 | C/G |
| chr1 | 10815668 | 3 | 1 | chr1 | 10815667 | rs1292657 | A/G |
| chr1 | 10886757 | 5 | 1 | chr1 | 10886756 | rs12732550 | A/G |
| chr1 | 10920185 | 3 | 1 | chr1 | 10920184 | rs1281033 | C/T |
| chr1 | 11412901 | 4 | 3 | chr1 | 11412899 | rs2379149 | C/T |
| chr1 | 11681107 | 0 | 0 | chr1 | 11681105 | rs6658124 | C/G |
| chr1 | 11749891 | 4 | 1 | chr1 | 11749890 | rs6540996 | A/G |
| chr1 | 11772034 | 2 | 4 | chr1 | 11772033 | rs868014 | C/T |
| chr1 | 11795608 | 2 | 2 | chr1 | 11795606 | rs72640251 | C/T |
| chr1 | 11890494 | 3 | 3 | chr1 | 11890491 | rs6662295 | C/G |
| chr1 | 12064048 | 6 | 2 | chr1 | 12064047 | rs2297875 | A/G |
| chr1 | 12547689 | 3 | 2 | chr1 | 12547687 | rs10864570 | C/T |
| chr1 | 12906896 | 0 | 0 | chr1 | 12906895 | rs1021406 | A/G |
| chr1 | 14944265 | 4 | 1 | chr1 | 14944263 | rs3795754 | C/T |
| chr1 | 14965328 | 2 | 6 | chr1 | 14965327 | rs12079599 | G/T |
| chr1 | 15231203 | 1 | 5 | chr1 | 15231202 | rs4661311 | A/G |
| chr1 | 15235129 | 1 | 12 | chr1 | 15235129 | rs35163828 | C/G |
| chr1 | 15316447 | 4 | 7 | chr1 | 15316445 | rs2235786 | A/G |
| chr1 | 15330989 | 1 | 4 | chr1 | 15330988 | rs4423000 | A/G |
| chr1 | 15375126 | 2 | 6 | chr1 | 15375125 | rs10754887 | A/G |
| chr1 | 15385556 | 3 | 1 | chr1 | 15385554 | rs761294 | C/G/T |
| chr1 | 16249081 | 4 | 1 | chr1 | 16249080 | rs72651918 | C/G |
| chr1 | 16249081 | 4 | 1 | chr1 | 16249080 | rs13306249 | C/G |
| chr1 | 16370635 | 1 | 4 | chr1 | 16370634 | rs11588341 | A/G |
| chr1 | 16382726 | 3 | 6 | chr1 | 16382725 | rs10907282 | A/G |
| chr1 | 16608720 | 1 | 10 | chr1 | 16608718 | rs4661746 | C/T |
| chr1 | 16735986 | 6 | 2 | chr1 | 16735984 | rs61769545 | A/C |
| chr1 | 16735986 | 6 | 2 | chr1 | 16735984 | rs71644008 | G/T |
| chr1 | 16798220 | 1 | 4 | chr1 | 16798218 | rs9728293 | C/G |
| chr1 | 16798220 | 1 | 4 | chr1 | 16798219 | rs610386 | A/G |
| chr1 | 16896492 | 7 | 10 | chr1 | 16896491 | rs601094 | A/G |
| chr1 | 16932355 | 3 | 2 | chr1 | 16932353 | rs1759230 | A/G |

| | | | | | | | |
|---|---|---|---|---|---|---|---|
| chr1 | 16932355 | 3 | 2 | chr1 | 16932354 | rs2779478 | C/G |
| chr1 | 17243741 | 4 | 1 | chr1 | 17243738 | rs12073028 | C/T |
| chr1 | 17267201 | 8 | 3 | chr1 | 17267199 | rs10157828 | C/T |
| chr1 | 17269464 | 0 | 0 | chr1 | 17269462 | rs2076594 | G/T |
| chr1 | 17429721 | 4 | 1 | chr1 | 17429719 | rs3003406 | A/C |
| chr1 | 17466851 | 1 | 3 | chr1 | 17466849 | rs4262593 | C/T |
| chr1 | 17489032 | 3 | 2 | chr1 | 17489031 | rs11588418 | A/G |
| chr1 | 17532401 | 6 | 5 | chr1 | 17532399 | rs3795214 | A/G |
| chr1 | 17590711 | 4 | 2 | chr1 | 17590710 | rs12127405 | A/G |
| chr1 | 17863640 | 5 | 2 | chr1 | 17863638 | rs6695710 | C/T |
| chr1 | 18147397 | 1 | 3 | chr1 | 18147396 | rs61049395 | A/G |
| chr1 | 18340681 | 4 | 3 | chr1 | 18340679 | rs876032 | A/G |
| chr1 | 18348210 | 4 | 1 | chr1 | 18348208 | rs72942402 | C/T |
| chr1 | 18352934 | 2 | 2 | chr1 | 18352933 | rs9662824 | A/G |
| chr1 | 18415658 | 3 | 1 | chr1 | 18415657 | rs6676376 | A/G |
| chr1 | 18705407 | 9 | 1 | chr1 | 18705406 | rs2356242 | A/G |
| chr1 | 19581165 | 3 | 8 | chr1 | 19581163 | rs10753560 | C/T |
| chr1 | 19899059 | 3 | 14 | chr1 | 19899058 | rs3934192 | C/T |
| chr1 | 20840598 | 2 | 3 | chr1 | 20840597 | rs3102071 | C/T |
| chr1 | 20927313 | 1 | 6 | chr1 | 20927312 | rs646786 | A/G |
| chr1 | 21560729 | 5 | 2 | chr1 | 21560727 | rs2682362 | A/G |
| chr1 | 21688365 | 2 | 6 | chr1 | 21688362 | rs904928 | C/G |
| chr1 | 21757096 | 2 | 3 | chr1 | 21757094 | rs1697416 | C/T |
| chr1 | 21849532 | 2 | 24 | chr1 | 21849531 | rs3767119 | A/G |
| chr1 | 22106172 | 2 | 3 | chr1 | 22106170 | rs66850327 | C/T |
| chr1 | 22449785 | 1 | 4 | chr1 | 22449783 | rs2744756 | C/T |
| chr1 | 22980676 | 1 | 5 | chr1 | 22980675 | rs2817904 | C/T |
| chr1 | 23171995 | 1 | 11 | chr1 | 23171993 | rs6692248 | C/T |
| chr1 | 23543141 | 6 | 4 | chr1 | 23543138 | rs12408922 | C/G/T |
| chr1 | 23682949 | 5 | 5 | chr1 | 23682946 | rs6686497 | C/T |
| chr1 | 23696386 | 16 | 2 | chr1 | 23696384 | rs1009590 | C/G |
| chr1 | 23837702 | 4 | 2 | chr1 | 23837701 | rs60379440 | A/G |

| | | | | | | | |
|---|---|---|---|---|---|---|---|
| chr1 | 23851510 | 2 | 2 | chr1 | 23851509 | rs7548320 | A/G |
| chr1 | 24092304 | 2 | 2 | chr1 | 24092303 | rs2502967 | A/G |
| chr1 | 24261267 | 1 | 4 | chr1 | 24261265 | rs3795303 | A/G |
| chr1 | 24679148 | 6 | 2 | chr1 | 24679146 | rs195738 | C/T |
| chr1 | 25113703 | 2 | 3 | chr1 | 25113702 | rs9438876 | A/G |
| chr1 | 25145876 | 3 | 6 | chr1 | 25145874 | rs876109 | C/T |
| chr1 | 25276676 | 6 | 1 | chr1 | 25276674 | rs371551 | A/G |
| chr1 | 25281053 | 9 | 2 | chr1 | 25281051 | rs311482 | A/G |
| chr1 | 25993624 | 2 | 3 | chr1 | 25993622 | rs807246 | A/G |
| chr1 | 25998962 | 2 | 2 | chr1 | 25998961 | rs7524771 | A/G |
| chr1 | 26220027 | 5 | 3 | chr1 | 26220026 | rs11247845 | C/G |
| chr1 | 26355226 | 8 | 3 | chr1 | 26355224 | rs2783716 | A/C |
| chr1 | 26381068 | 1 | 3 | chr1 | 26381066 | rs2275945 | A/G |
| chr1 | 27240107 | 4 | 7 | chr1 | 27240106 | rs502928 | A/G |
| chr1 | 27552384 | 4 | 7 | chr1 | 27552383 | rs3813795 | A/G |
| chr1 | 28122271 | 2 | 3 | chr1 | 28122269 | rs4908373 | C/T |
| chr1 | 28334249 | 2 | 2 | chr1 | 28334247 | rs10751727 | C/T |
| chr1 | 29430868 | 2 | 11 | chr1 | 29430867 | rs34921636 | A/G |
| chr1 | 30178769 | 2 | 2 | chr1 | 30178767 | rs12756043 | C/G |
| chr1 | 30961488 | 1 | 3 | chr1 | 30961487 | rs20566 | A/G |
| chr1 | 31134812 | 5 | 2 | chr1 | 31134810 | rs10753239 | C/T |
| chr1 | 31679469 | 11 | 1 | chr1 | 31679468 | rs11587589 | A/G |
| chr1 | 31745344 | 1 | 4 | chr1 | 31745342 | rs1316509 | C/T |
| chr1 | 31894751 | 5 | 3 | chr1 | 31894750 | rs7545321 | A/G |
| chr1 | 32057091 | 6 | 7 | chr1 | 32057090 | rs61513120 | A/G |
| chr1 | 32086987 | 2 | 6 | chr1 | 32086986 | rs1536131 | C/T |
| chr1 | 32194699 | 1 | 6 | chr1 | 32194698 | rs67415556 | A/G |
| chr1 | 32933466 | 0 | 0 | chr1 | 32933464 | rs360042 | C/T |
| chr1 | 33389998 | 0 | 0 | chr1 | 33389996 | rs673894 | C/T |
| chr1 | 33645181 | 2 | 3 | chr1 | 33645179 | rs6661037 | C/T |
| chr1 | 34098076 | 3 | 1 | chr1 | 34098074 | rs1321625 | C/T |
| chr1 | 34185402 | 4 | 11 | chr1 | 34185401 | rs556392 | C/T |

| | | | | | | | |
|---|---|---|---|---|---|---|---|
| chr1 | 34212904 | 3 | 3 | chr1 | 34212902 | rs536766 | C/T |
| chr1 | 34366829 | 7 | 5 | chr1 | 34366826 | rs9425997 | C/T |
| chr1 | 35003253 | 2 | 2 | chr1 | 35003252 | rs1123973 | C/T |
| chr1 | 37537556 | 2 | 9 | chr1 | 37537555 | rs10737167 | A/G |
| chr1 | 37601310 | 5 | 1 | chr1 | 37601309 | rs3920509 | C/T |
| chr1 | 37732038 | 6 | 1 | chr1 | 37732036 | rs215210 | A/G |
| chr1 | 37973940 | 2 | 4 | chr1 | 37973939 | rs631453 | C/T |
| chr1 | 38374741 | 4 | 4 | chr1 | 38374739 | rs12074713 | A/C |
| chr1 | 38551984 | 5 | 4 | chr1 | 38551982 | rs2480698 | C/T |
| chr1 | 38660651 | 2 | 4 | chr1 | 38660649 | rs72667590 | C/T |
| chr1 | 38986949 | 2 | 2 | chr1 | 38986947 | rs6701519 | C/T |
| chr1 | 39233382 | 2 | 7 | chr1 | 39233380 | rs6662758 | C/T |
| chr1 | 39707300 | 1 | 3 | chr1 | 39707299 | rs6698781 | A/G |
| chr1 | 39766020 | 5 | 2 | chr1 | 39766018 | rs1180343 | C/T |
| chr1 | 39991868 | 4 | 4 | chr1 | 39991866 | rs14528 | C/T |
| chr1 | 40579621 | 2 | 2 | chr1 | 40579619 | rs442418 | A/G |
| chr1 | 40880737 | 4 | 1 | chr1 | 40880735 | rs11208590 | C/T |
| chr1 | 41038495 | 3 | 2 | chr1 | 41038494 | rs11806761 | A/G |
| chr1 | 41099473 | 2 | 3 | chr1 | 41099472 | rs12044255 | C/G |
| chr1 | 41161409 | 6 | 2 | chr1 | 41161407 | rs7513904 | C/T |
| chr1 | 41193572 | 7 | 1 | chr1 | 41193571 | rs6671627 | A/G |
| chr1 | 41727677 | 3 | 3 | chr1 | 41727676 | rs71648550 | A/G |
| chr1 | 41748917 | 7 | 4 | chr1 | 41748914 | rs9439043 | C/T |
| chr1 | 41748917 | 7 | 4 | chr1 | 41748916 | rs58930436 | A/G |
| chr1 | 41988291 | 8 | 1 | chr1 | 41988289 | rs907640 | A/G |
| chr1 | 42034615 | 4 | 3 | chr1 | 42034613 | rs7538636 | C/T |
| chr1 | 42701719 | 2 | 7 | chr1 | 42701716 | rs2275117 | A/C |
| chr1 | 43493581 | 2 | 3 | chr1 | 43493580 | rs3862227 | A/G |
| chr1 | 44151686 | 8 | 3 | chr1 | 44151685 | rs946237 | A/C |
| chr1 | 44238007 | 4 | 8 | chr1 | 44238005 | rs803619 | C/T |
| chr1 | 44540836 | 2 | 3 | chr1 | 44540835 | rs11808967 | G/T |
| chr1 | 44883947 | 9 | 2 | chr1 | 44883946 | rs12728857 | A/G |

| | | | | | | | |
|---|---|---|---|---|---|---|---|
| chr1 | 46631321 | 7 | 4 | chr1 | 46631320 | rs1984490 | C/T |
| chr1 | 47445884 | 4 | 2 | chr1 | 47445882 | rs13373881 | C/T |
| chr1 | 47677498 | 2 | 10 | chr1 | 47677495 | rs2405913 | C/G |
| chr1 | 47757279 | 3 | 3 | chr1 | 47757278 | rs471608 | A/C |
| chr1 | 47947161 | 5 | 3 | chr1 | 47947159 | rs4926971 | C/T |
| chr1 | 48454622 | 2 | 12 | chr1 | 48454620 | rs10788883 | A/C |
| chr1 | 48951338 | 5 | 1 | chr1 | 48951336 | rs320060 | C/T |
| chr1 | 49112190 | 2 | 4 | chr1 | 49112188 | rs12747763 | C/T |
| chr1 | 50523337 | 3 | 6 | chr1 | 50523336 | rs2356866 | A/G |
| chr1 | 50571020 | 2 | 6 | chr1 | 50571018 | rs6692513 | C/T |
| chr1 | 52056607 | 2 | 2 | chr1 | 52056605 | rs2747525 | G/T |
| chr1 | 52545213 | 2 | 3 | chr1 | 52545212 | rs2762830 | C/T |
| chr1 | 53300536 | 2 | 2 | chr1 | 53300535 | rs7513730 | G/T |
| chr1 | 53352746 | 5 | 1 | chr1 | 53352744 | rs1288377 | A/C |
| chr1 | 53433582 | 9 | 2 | chr1 | 53433580 | rs7554022 | A/C |
| chr1 | 53454287 | 4 | 2 | chr1 | 53454286 | rs1134688 | A/C |
| chr1 | 53768346 | 3 | 5 | chr1 | 53768344 | rs12025611 | C/T |
| chr1 | 53780453 | 3 | 5 | chr1 | 53780450 | rs12403311 | C/T |
| chr1 | 53929213 | 1 | 8 | chr1 | 53929211 | rs12082358 | A/C |
| chr1 | 53976913 | 4 | 2 | chr1 | 53976911 | rs702488 | A/G |
| chr1 | 54090286 | 4 | 3 | chr1 | 54090284 | rs1127922 | A/G |
| chr1 | 54496836 | 3 | 7 | chr1 | 54496833 | rs3766442 | A/G |
| chr1 | 54718606 | 1 | 4 | chr1 | 54718604 | rs4927129 | C/T |
| chr1 | 54758421 | 5 | 3 | chr1 | 54758419 | rs449141 | A/G |
| chr1 | 54836820 | 2 | 2 | chr1 | 54836818 | rs2664061 | A/G |
| chr1 | 54862510 | 4 | 6 | chr1 | 54862508 | rs1702005 | C/T |
| chr1 | 54893058 | 1 | 3 | chr1 | 54893056 | rs2570770 | A/G |
| chr1 | 55036140 | 1 | 3 | chr1 | 55036138 | rs619476 | A/G |
| chr1 | 55475860 | 1 | 5 | chr1 | 55475859 | rs3897715 | C/T |
| chr1 | 56173710 | 8 | 1 | chr1 | 56173708 | rs11206687 | C/T |
| chr1 | 56559139 | 19 | 2 | chr1 | 56559138 | rs4534430 | A/G |
| chr1 | 56733943 | 5 | 4 | chr1 | 56733941 | rs10888976 | C/T |

| | | | | | | | |
|---|---|---|---|---|---|---|---|
| chr1 | 56754407 | 4 | 1 | chr1 | 56754406 | rs7525693 | A/G |
| chr1 | 56766005 | 3 | 4 | chr1 | 56766002 | rs1759752 | C/G |
| chr1 | 57091408 | 1 | 3 | chr1 | 57091407 | rs658444 | C/T |
| chr1 | 57418477 | 6 | 3 | chr1 | 57418476 | rs4912431 | G/T |
| chr1 | 57424081 | 5 | 6 | chr1 | 57424079 | rs23705 | G/T |
| chr1 | 57538644 | 1 | 3 | chr1 | 57538643 | rs499397 | A/C |
| chr1 | 57864167 | 6 | 6 | chr1 | 57864165 | rs10732834 | C/T |
| chr1 | 58229115 | 11 | 2 | chr1 | 58229113 | rs1188000 | A/G |
| chr1 | 61855312 | 2 | 3 | chr1 | 61855310 | rs57796953 | A/C |
| chr1 | 63175762 | 1 | 3 | chr1 | 63175761 | rs2803235 | A/G |
| chr1 | 63242032 | 2 | 6 | chr1 | 63242030 | rs72671747 | C/T |
| chr1 | 63255340 | 1 | 3 | chr1 | 63255338 | rs17124332 | C/T |
| chr1 | 63846505 | 6 | 1 | chr1 | 63846503 | rs855302 | A/G |
| chr1 | 63962020 | 3 | 1 | chr1 | 63962019 | rs2819167 | A/G |
| chr1 | 64176848 | 5 | 3 | chr1 | 64176845 | rs855943 | A/G |
| chr1 | 64233517 | 8 | 1 | chr1 | 64233515 | rs285366 | C/T |
| chr1 | 64614526 | 1 | 3 | chr1 | 64614525 | rs7541786 | A/G |
| chr1 | 64618287 | 2 | 3 | chr1 | 64618285 | rs10889481 | C/T |
| chr1 | 64799206 | 1 | 5 | chr1 | 64799205 | rs10789160 | A/G |
| chr1 | 65661443 | 3 | 1 | chr1 | 65661441 | rs9436297 | C/T |
| chr1 | 65762700 | 7 | 1 | chr1 | 65762698 | rs1171281 | A/G |
| chr1 | 67311945 | 4 | 2 | chr1 | 67311943 | rs983111 | C/T |
| chr1 | 67706547 | 1 | 3 | chr1 | 67706546 | rs2105263 | A/G |
| chr1 | 68179751 | 1 | 9 | chr1 | 68179750 | rs1938428 | C/T |
| chr1 | 68470969 | 6 | 2 | chr1 | 68470967 | rs2820474 | C/T |
| chr1 | 69496681 | 12 | 2 | chr1 | 69496680 | rs2791210 | C/T |
| chr1 | 71292382 | 0 | 0 | chr1 | 71292380 | rs2744907 | C/G |
| chr1 | 71446327 | 6 | 3 | chr1 | 71446325 | rs12406316 | C/T |
| chr1 | 72671947 | 1 | 3 | chr1 | 72671946 | rs1445581 | C/T |
| chr1 | 73516461 | 1 | 8 | chr1 | 73516460 | rs11210193 | A/G |
| chr1 | 75962333 | 4 | 2 | chr1 | 75962332 | rs1251077 | C/G |
| chr1 | 76738207 | 3 | 2 | chr1 | 76738205 | rs1436085 | C/T |

| | | | | | | | |
|---|---|---|---|---|---|---|---|
| chr1 | 76896899 | 6 | 1 | chr1 | 76896898 | rs4949737 | A/G |
| chr1 | 76898777 | 5 | 2 | chr1 | 76898776 | rs681033 | A/G |
| chr1 | 77533473 | 3 | 2 | chr1 | 77533472 | rs1167209 | C/T |
| chr1 | 78222304 | 3 | 3 | chr1 | 78222302 | rs61778901 | C/T |
| chr1 | 80362547 | 1 | 4 | chr1 | 80362545 | rs12119851 | A/C |
| chr1 | 80704647 | 4 | 1 | chr1 | 80704645 | rs10735761 | C/T |
| chr1 | 81449986 | 5 | 2 | chr1 | 81449985 | rs7553222 | A/G |
| chr1 | 81479816 | 5 | 1 | chr1 | 81479815 | rs1536571 | A/G |
| chr1 | 82879127 | 6 | 6 | chr1 | 82879126 | rs11163533 | A/G |
| chr1 | 83004557 | 13 | 11 | chr1 | 83004556 | rs320905 | A/C |
| chr1 | 85235253 | 1 | 3 | chr1 | 85235252 | rs11161505 | G/T |
| chr1 | 85891035 | 3 | 7 | chr1 | 85891034 | rs2970548 | A/G |
| chr1 | 86817172 | 1 | 3 | chr1 | 86817170 | rs772607 | A/G |
| chr1 | 87250982 | 4 | 1 | chr1 | 87250980 | rs6576859 | C/T |
| chr1 | 87812599 | 6 | 1 | chr1 | 87812598 | rs6658600 | A/G |
| chr1 | 89624998 | 2 | 2 | chr1 | 89624997 | rs6669782 | A/G |
| chr1 | 89753965 | 5 | 2 | chr1 | 89753963 | rs11580069 | C/T |
| chr1 | 90000467 | 3 | 3 | chr1 | 90000465 | rs551180 | A/G |
| chr1 | 90082213 | 5 | 8 | chr1 | 90082210 | rs10922734 | C/T |
| chr1 | 90968765 | 3 | 1 | chr1 | 90968763 | rs12042107 | C/T |
| chr1 | 91097911 | 2 | 4 | chr1 | 91097910 | rs12046172 | G/T |
| chr1 | 91309012 | 3 | 4 | chr1 | 91309011 | rs2748708 | A/C |
| chr1 | 91399559 | 4 | 3 | chr1 | 91399558 | rs631502 | C/T |
| chr1 | 91950251 | 3 | 2 | chr1 | 91950250 | rs11165300 | G/T |
| chr1 | 94007002 | 3 | 1 | chr1 | 94007000 | rs12409459 | C/T |
| chr1 | 94046386 | 5 | 1 | chr1 | 94046385 | rs74101573 | A/G |
| chr1 | 94246434 | 1 | 4 | chr1 | 94246432 | rs2275029 | C/T |
| chr1 | 94246434 | 1 | 4 | chr1 | 94246433 | rs56142141 | A/G |
| chr1 | 94334076 | 4 | 5 | chr1 | 94334076 | rs4147822 | C/T |
| chr1 | 94774189 | 4 | 8 | chr1 | 94774187 | rs698946 | A/C |
| chr1 | 94876738 | 5 | 3 | chr1 | 94876737 | rs2996285 | C/T |
| chr1 | 94925305 | 3 | 1 | chr1 | 94925303 | rs1344170 | C/T |

| | | | | | | | |
|---|---|---|---|---|---|---|---|
| chr1 | 94925305 | 3 | 1 | chr1 | 94925304 | rs3905416 | G/T |
| chr1 | 94925305 | 3 | 1 | chr1 | 94925305 | rs34449174 | -/G |
| chr1 | 96763080 | 3 | 1 | chr1 | 96763079 | rs11165659 | C/G |
| chr1 | 98159544 | 2 | 5 | chr1 | 98159542 | rs61787828 | A/C |
| chr1 | 99465747 | 3 | 4 | chr1 | 99465746 | rs997484 | A/C |
| chr1 | 99922506 | 2 | 4 | chr1 | 99922505 | rs6698880 | A/G |
| chr1 | 100207198 | 3 | 3 | chr1 | 100207197 | rs6577152 | A/G |
| chr1 | 101552536 | 3 | 1 | chr1 | 101552535 | rs1122392 | C/T |
| chr1 | 101788681 | 1 | 4 | chr1 | 101788681 | rs1146717 | C/T |
| chr1 | 102359245 | 10 | 1 | chr1 | 102359243 | rs6577305 | C/T |
| chr1 | 107781201 | 5 | 2 | chr1 | 107781201 | rs483206 | G/T |
| chr1 | 108241607 | 6 | 1 | chr1 | 108241606 | rs11185203 | A/G |
| chr1 | 109091329 | 1 | 3 | chr1 | 109091326 | rs7553437 | C/T |
| chr1 | 109165640 | 8 | 1 | chr1 | 109165639 | rs2788437 | A/G |
| chr1 | 110122552 | 2 | 2 | chr1 | 110122551 | rs56145123 | A/G |
| chr1 | 110543609 | 4 | 1 | chr1 | 110543607 | rs2603579 | A/G |
| chr1 | 110858338 | 2 | 3 | chr1 | 110858336 | rs1281178 | A/G |
| chr1 | 110951735 | 4 | 1 | chr1 | 110951735 | rs7538552 | A/G |
| chr1 | 112156142 | 3 | 1 | chr1 | 112156141 | rs2813869 | A/G |
| chr1 | 112374789 | 6 | 2 | chr1 | 112374787 | rs12749667 | C/T |
| chr1 | 112625346 | 1 | 3 | chr1 | 112625344 | rs4073808 | C/T |
| chr1 | 112625346 | 1 | 3 | chr1 | 112625345 | rs3121986 | A/G |
| chr1 | 112700969 | 8 | 1 | chr1 | 112700968 | rs1936050 | A/G |
| chr1 | 114636261 | 1 | 4 | chr1 | 114636259 | rs12031081 | C/T |
| chr1 | 114636261 | 1 | 4 | chr1 | 114636260 | rs61812921 | A/G |
| chr1 | 115356338 | 1 | 5 | chr1 | 115356336 | rs6673805 | C/T |
| chr1 | 116231434 | 3 | 8 | chr1 | 116231432 | rs1617980 | A/G |
| chr1 | 117395987 | 1 | 3 | chr1 | 117395986 | rs10801929 | A/G |
| chr1 | 117559420 | 6 | 4 | chr1 | 117559419 | rs7549280 | A/G |
| chr1 | 117999685 | 3 | 1 | chr1 | 117999684 | rs68178 | A/G |
| chr1 | 118401066 | 5 | 5 | chr1 | 118401065 | rs12124362 | A/G |
| chr1 | 118703766 | 7 | 1 | chr1 | 118703765 | rs2474938 | A/C/T |

| | | | | | | | |
|---|---|---|---|---|---|---|---|
| chr1 | 118948408 | 1 | 7 | chr1 | 118948406 | rs12137431 | C/T |
| chr1 | 119460448 | 1 | 4 | chr1 | 119460447 | rs10923752 | A/G |
| chr1 | 121186281 | 18 | 5 | chr1 | 121186278 | rs56127708 | C/T |
| chr1 | 121186281 | 18 | 5 | chr1 | 121186279 | rs28848264 | A/C |
| chr1 | 121186281 | 18 | 5 | chr1 | 121186280 | rs4115080 | A/G |
| chr1 | 121186281 | 18 | 5 | chr1 | 121186281 | rs66910629 | C/G |
| chr1 | 121186729 | 26 | 34 | chr1 | 121186726 | rs28808143 | A/C/T |
| chr1 | 121186729 | 26 | 34 | chr1 | 121186727 | rs4788400 | A/C/T |
| chr1 | 121186729 | 26 | 34 | chr1 | 121186728 | rs4127587 | A/G/T |
| chr1 | 121186729 | 26 | 34 | chr1 | 121186729 | rs1138158 | A/C/T |
| chr1 | 121186924 | 1 | 92 | chr1 | 121186921 | rs55662648 | C/T |
| chr1 | 121186924 | 1 | 92 | chr1 | 121186922 | rs7412645 | G/T |
| chr1 | 121186924 | 1 | 92 | chr1 | 121186924 | rs7412646 | C/G |
| chr1 | 142044473 | 2 | 20 | chr1 | 142044471 | rs61785362 | C/T |
| chr1 | 142308909 | 3 | 2 | chr1 | 142308909 | rs1712140 | C/T |
| chr1 | 143852726 | 3 | 2 | chr1 | 143852725 | rs1378193 | C/T |
| chr1 | 144341830 | 1 | 3 | chr1 | 144341829 | rs10752828 | A/G |
| chr1 | 145142814 | 4 | 1 | chr1 | 145142813 | rs6593745 | A/G |
| chr1 | 145175209 | 3 | 6 | chr1 | 145175207 | rs12140294 | C/T |
| chr1 | 145533942 | 6 | 2 | chr1 | 145533941 | rs10465881 | A/G |
| chr1 | 145688063 | 1 | 4 | chr1 | 145688061 | rs7513213 | C/T |
| chr1 | 146193147 | 3 | 2 | chr1 | 146193146 | rs2999687 | A/G |
| chr1 | 147173975 | 2 | 4 | chr1 | 147173974 | rs150998 | C/T |
| chr1 | 147173975 | 2 | 4 | chr1 | 147173974 | rs71221119 | A/G |
| chr1 | 147561390 | 2 | 3 | chr1 | 147561387 | rs7514503 | C/T |
| chr1 | 147561390 | 2 | 3 | chr1 | 147561388 | rs679647 | A/G |
| chr1 | 147561390 | 2 | 3 | chr1 | 147561389 | rs71255886 | CC/TT |
| chr1 | 147580610 | 3 | 2 | chr1 | 147580609 | rs1376166 | A/G |
| chr1 | 147580610 | 3 | 2 | chr1 | 147580609 | rs61786382 | A/G |
| chr1 | 147966141 | 3 | 2 | chr1 | 147966140 | rs1376166 | A/G |
| chr1 | 147966141 | 3 | 2 | chr1 | 147966140 | rs55935832 | A/G |
| chr1 | 148522212 | 2 | 3 | chr1 | 148522210 | rs2147324 | A/G |

| | | | | | | | |
|---|---|---|---|---|---|---|---|
| chr1 | 149115306 | 3 | 1 | chr1 | 149115304 | rs3820541 | C/G |
| chr1 | 149779872 | 4 | 2 | chr1 | 149779870 | rs3790507 | A/G |
| chr1 | 150655100 | 9 | 2 | chr1 | 150655098 | rs3753446 | A/C |
| chr1 | 151316542 | 3 | 4 | chr1 | 151316540 | rs399550 | C/T |
| chr1 | 151561278 | 3 | 1 | chr1 | 151561276 | rs2916229 | A/G |
| chr1 | 151569041 | 3 | 5 | chr1 | 151569040 | rs2771120 | A/G |
| chr1 | 151588037 | 3 | 3 | chr1 | 151588036 | rs10888555 | C/G |
| chr1 | 151707043 | 6 | 2 | chr1 | 151707042 | rs2986206 | A/G |
| chr1 | 152264428 | 5 | 1 | chr1 | 152264427 | rs7552737 | A/G |
| chr1 | 152584043 | 3 | 5 | chr1 | 152584041 | rs1194586 | A/G |
| chr1 | 152760707 | 9 | 3 | chr1 | 152760705 | rs4269769 | C/G |
| chr1 | 152948574 | 5 | 1 | chr1 | 152948573 | rs6695232 | A/G |
| chr1 | 153009199 | 3 | 7 | chr1 | 153009198 | rs4845392 | A/G |
| chr1 | 153041582 | 3 | 4 | chr1 | 153041581 | rs2135695 | C/T |
| chr1 | 153299942 | 3 | 3 | chr1 | 153299940 | rs11264304 | C/T |
| chr1 | 153434410 | 3 | 2 | chr1 | 153434409 | rs41264915 | A/G |
| chr1 | 154452904 | 881 | 171 | chr1 | 154452901 | rs58984276 | -/CT |
| chr1 | 154452904 | 881 | 171 | chr1 | 154452902 | rs61813337 | C/T |
| chr1 | 154452974 | 832 | 522 | chr1 | 154452972 | rs1062299 | C/T |
| chr1 | 154452974 | 832 | 522 | chr1 | 154452974 | rs3899924 | C/G |
| chr1 | 154805779 | 4 | 9 | chr1 | 154805777 | rs6427319 | C/T |
| chr1 | 154829555 | 10 | 2 | chr1 | 154829553 | rs942960 | C/T |
| chr1 | 155581863 | 1 | 3 | chr1 | 155581861 | rs4661127 | C/T |
| chr1 | 155652736 | 1 | 4 | chr1 | 155652735 | rs6690419 | G/T |
| chr1 | 156253604 | 5 | 2 | chr1 | 156253602 | rs6661931 | C/T |
| chr1 | 156314225 | 2 | 4 | chr1 | 156314224 | rs11264901 | A/G |
| chr1 | 156321003 | 7 | 5 | chr1 | 156321002 | rs927662 | A/G |
| chr1 | 156371271 | 1 | 8 | chr1 | 156371270 | rs28689939 | A/G |
| chr1 | 156809084 | 4 | 1 | chr1 | 156809083 | rs863354 | A/G |
| chr1 | 157383426 | 1 | 8 | chr1 | 157383424 | rs703154 | C/T |
| chr1 | 158357299 | 5 | 2 | chr1 | 158357297 | rs2854246 | C/T |
| chr1 | 158731173 | 10 | 3 | chr1 | 158731171 | rs678456 | A/G |

| | | | | | | | |
|---|---|---|---|---|---|---|---|
| chr1 | 158732980 | 2 | 3 | chr1 | 158732979 | rs3795326 | C/T |
| chr1 | 159162494 | 3 | 1 | chr1 | 159162493 | rs11265526 | A/G |
| chr1 | 159848825 | 11 | 3 | chr1 | 159848823 | rs74127049 | C/G |
| chr1 | 159966856 | 8 | 1 | chr1 | 159966854 | rs1503809 | C/T |
| chr1 | 160127137 | 9 | 11 | chr1 | 160127135 | rs6683830 | A/C |
| chr1 | 160238288 | 2 | 3 | chr1 | 160238287 | rs2499833 | C/T |
| chr1 | 160428959 | 4 | 2 | chr1 | 160428956 | rs59656361 | C/T |
| chr1 | 160428959 | 4 | 2 | chr1 | 160428957 | rs12047527 | C/T |
| chr1 | 160802131 | 3 | 9 | chr1 | 160802129 | rs6695928 | C/T |
| chr1 | 161103387 | 1 | 3 | chr1 | 161103385 | rs7525831 | C/T |
| chr1 | 161496051 | 2 | 2 | chr1 | 161496049 | rs3413 | C/T |
| chr1 | 161636329 | 2 | 9 | chr1 | 161636327 | rs3010355 | C/T |
| chr1 | 162083625 | 4 | 2 | chr1 | 162083624 | rs781940 | C/T |
| chr1 | 162767660 | 10 | 4 | chr1 | 162767659 | rs1417294 | G/T |
| chr1 | 163197548 | 2 | 7 | chr1 | 163197546 | rs74118271 | C/T |
| chr1 | 163197548 | 2 | 7 | chr1 | 163197547 | rs1936087 | C/T |
| chr1 | 163619835 | 3 | 1 | chr1 | 163619833 | rs1501493 | C/T |
| chr1 | 163961522 | 3 | 4 | chr1 | 163961520 | rs7524755 | C/T |
| chr1 | 164847266 | 7 | 2 | chr1 | 164847264 | rs473746 | A/G |
| chr1 | 164874183 | 2 | 3 | chr1 | 164874181 | rs513797 | C/T |
| chr1 | 165674311 | 4 | 2 | chr1 | 165674309 | rs2462553 | A/G |
| chr1 | 166027431 | 1 | 3 | chr1 | 166027430 | rs10800323 | A/G |
| chr1 | 166506369 | 1 | 3 | chr1 | 166506368 | rs10800348 | A/G |
| chr1 | 166632759 | 2 | 3 | chr1 | 166632757 | rs1407946 | G/T |
| chr1 | 166819880 | 3 | 1 | chr1 | 166819878 | rs10918928 | C/T |
| chr1 | 167403458 | 2 | 4 | chr1 | 167403456 | rs10800408 | C/T |
| chr1 | 168495377 | 1 | 3 | chr1 | 168495375 | rs35313496 | A/C |
| chr1 | 173605696 | 6 | 1 | chr1 | 173605695 | rs6702574 | A/G |
| chr1 | 176754897 | 3 | 1 | chr1 | 176754896 | rs10913564 | A/G |
| chr1 | 177190100 | 5 | 3 | chr1 | 177190099 | rs2039533 | A/G |
| chr1 | 177601541 | 3 | 2 | chr1 | 177601539 | rs16853998 | C/G |
| chr1 | 177684404 | 4 | 4 | chr1 | 177684402 | rs11580135 | C/T |

| | | | | | | | |
|---|---|---|---|---|---|---|---|
| chr1 | 178231502 | 2 | 3 | chr1 | 178231501 | rs2501615 | C/T |
| chr1 | 178779941 | 10 | 11 | chr1 | 178779940 | rs6425647 | A/G |
| chr1 | 178782842 | 4 | 8 | chr1 | 178782841 | rs4652509 | A/G |
| chr1 | 179154001 | 4 | 3 | chr1 | 179153999 | rs13374657 | C/T |
| chr1 | 179375631 | 4 | 5 | chr1 | 179375630 | rs4652565 | C/G |
| chr1 | 179448890 | 2 | 2 | chr1 | 179448889 | rs10732971 | A/G |
| chr1 | 179449181 | 3 | 7 | chr1 | 179449179 | rs10752842 | C/T |
| chr1 | 179458435 | 2 | 3 | chr1 | 179458433 | rs874470 | C/T |
| chr1 | 179659323 | 10 | 1 | chr1 | 179659321 | rs631583 | A/G |
| chr1 | 179848330 | 4 | 1 | chr1 | 179848328 | rs6671804 | C/T |
| chr1 | 180534411 | 1 | 5 | chr1 | 180534410 | rs2477186 | A/G |
| chr1 | 180626791 | 2 | 3 | chr1 | 180626791 | rs4075940 | G/T |
| chr1 | 180824761 | 3 | 2 | chr1 | 180824759 | rs10911102 | C/T |
| chr1 | 180824761 | 3 | 2 | chr1 | 180824761 | rs12743617 | G/T |
| chr1 | 181524102 | 1 | 5 | chr1 | 181524100 | rs638066 | A/C |
| chr1 | 181779464 | 1 | 4 | chr1 | 181779462 | rs789176 | C/T |
| chr1 | 183087670 | 2 | 3 | chr1 | 183087668 | rs487685 | C/T |
| chr1 | 184886475 | 5 | 8 | chr1 | 184886474 | rs718665 | A/G |
| chr1 | 185720251 | 4 | 2 | chr1 | 185720249 | rs1938508 | C/T |
| chr1 | 185865497 | 1 | 3 | chr1 | 185865495 | rs17605276 | C/T |
| chr1 | 185874250 | 3 | 2 | chr1 | 185874249 | rs1591829 | A/G |
| chr1 | 185961301 | 1 | 6 | chr1 | 185961299 | rs6674535 | C/T |
| chr1 | 186824649 | 1 | 4 | chr1 | 186824647 | rs28698181 | C/T |
| chr1 | 188226827 | 5 | 1 | chr1 | 188226826 | rs1320748 | A/G |
| chr1 | 189108611 | 1 | 3 | chr1 | 189108609 | rs2118652 | A/G |
| chr1 | 190311997 | 7 | 8 | chr1 | 190311995 | rs2999593 | C/T |
| chr1 | 190841995 | 6 | 4 | chr1 | 190841992 | rs1977259 | G/T |
| chr1 | 192089274 | 1 | 4 | chr1 | 192089272 | rs594431 | C/T |
| chr1 | 192116280 | 4 | 1 | chr1 | 192116278 | rs517206 | C/T |
| chr1 | 192143882 | 2 | 4 | chr1 | 192143881 | rs480580 | A/G |
| chr1 | 192224014 | 4 | 2 | chr1 | 192224013 | rs629872 | C/T |
| chr1 | 192248460 | 2 | 2 | chr1 | 192248458 | rs655426 | A/G |

| | | | | | | | |
|---|---|---|---|---|---|---|---|
| chr1 | 193207960 | 4 | 3 | chr1 | 193207959 | rs339572 | A/G |
| chr1 | 194333393 | 2 | 2 | chr1 | 194333392 | rs7520620 | A/G |
| chr1 | 196021009 | 4 | 6 | chr1 | 196021008 | rs2488399 | A/G |
| chr1 | 196334660 | 2 | 2 | chr1 | 196334658 | rs1233812 | A/G |
| chr1 | 197542818 | 3 | 1 | chr1 | 197542816 | rs10753848 | C/T |
| chr1 | 198180816 | 1 | 3 | chr1 | 198180815 | rs10919777 | C/G |
| chr1 | 198535759 | 4 | 1 | chr1 | 198535756 | rs940400 | G/T |
| chr1 | 198590752 | 2 | 2 | chr1 | 198590750 | rs10919909 | C/T |
| chr1 | 199130891 | 3 | 1 | chr1 | 199130889 | rs184164 | A/G |
| chr1 | 199290604 | 1 | 4 | chr1 | 199290602 | rs10800755 | C/G |
| chr1 | 199441897 | 4 | 2 | chr1 | 199441896 | rs9651057 | A/G |
| chr1 | 199458280 | 2 | 2 | chr1 | 199458279 | rs6427886 | C/G |
| chr1 | 199460797 | 2 | 3 | chr1 | 199460796 | rs12134361 | A/G |
| chr1 | 199484822 | 4 | 15 | chr1 | 199484820 | rs7536174 | C/T |
| chr1 | 199528426 | 3 | 2 | chr1 | 199528424 | rs864402 | A/G |
| chr1 | 200392521 | 4 | 12 | chr1 | 200392521 | rs10753910 | A/G |
| chr1 | 200405102 | 2 | 2 | chr1 | 200405100 | rs3935009 | C/T |
| chr1 | 200452602 | 3 | 2 | chr1 | 200452601 | rs930735 | A/G |
| chr1 | 201422704 | 2 | 3 | chr1 | 201422702 | rs10399931 | C/T |
| chr1 | 201527124 | 5 | 3 | chr1 | 201527122 | rs4543864 | C/T |
| chr1 | 201632979 | 3 | 1 | chr1 | 201632979 | rs12738866 | G/T |
| chr1 | 201850328 | 4 | 1 | chr1 | 201850327 | rs3900967 | C/T |
| chr1 | 201875495 | 5 | 8 | chr1 | 201875494 | rs10793748 | A/G |
| chr1 | 202612360 | 1 | 3 | chr1 | 202612358 | rs2942146 | A/G |
| chr1 | 203682683 | 4 | 4 | chr1 | 203682682 | rs913725 | C/G |
| chr1 | 203768092 | 2 | 8 | chr1 | 203768091 | rs1042832 | A/G |
| chr1 | 203768144 | 3 | 3 | chr1 | 203768143 | rs14028 | A/G |
| chr1 | 203816183 | 1 | 4 | chr1 | 203816181 | rs4523578 | C/T |
| chr1 | 203826743 | 10 | 1 | chr1 | 203826741 | rs7539751 | C/T |
| chr1 | 204590878 | 2 | 9 | chr1 | 204590877 | rs12136762 | C/T |
| chr1 | 204978189 | 2 | 4 | chr1 | 204978188 | rs4311892 | A/G |
| chr1 | 206191118 | 3 | 2 | chr1 | 206191117 | rs11578744 | A/G |

| | | | | | | | |
|---|---|---|---|---|---|---|---|
| chr1 | 206301674 | 6 | 3 | chr1 | 206301673 | rs2478822 | C/T |
| chr1 | 206345124 | 6 | 1 | chr1 | 206345122 | rs4844405 | C/T |
| chr1 | 206449532 | 3 | 18 | chr1 | 206449530 | rs2801210 | C/T |
| chr1 | 206482711 | 1 | 6 | chr1 | 206482709 | rs12132513 | A/C |
| chr1 | 207396168 | 10 | 1 | chr1 | 207396167 | rs6672764 | A/G |
| chr1 | 207999954 | 1 | 4 | chr1 | 207999953 | rs6685077 | A/G |
| chr1 | 208922474 | 5 | 2 | chr1 | 208922473 | rs1890841 | A/G |
| chr1 | 210184988 | 1 | 5 | chr1 | 210184988 | rs2993540 | C/T |
| chr1 | 212315288 | 2 | 3 | chr1 | 212315286 | rs72755405 | C/T |
| chr1 | 212630965 | 3 | 1 | chr1 | 212630962 | rs7550799 | C/T |
| chr1 | 212634001 | 2 | 3 | chr1 | 212634000 | rs6657368 | A/G |
| chr1 | 214484299 | 6 | 1 | chr1 | 214484297 | rs682319 | C/T |
| chr1 | 214861049 | 5 | 1 | chr1 | 214861048 | rs17670802 | A/G |
| chr1 | 215587316 | 9 | 6 | chr1 | 215587316 | rs61826665 | G/T |
| chr1 | 216129615 | 3 | 1 | chr1 | 216129613 | rs12753990 | C/G |
| chr1 | 217289941 | 2 | 8 | chr1 | 217289939 | rs871107 | C/T |
| chr1 | 217779405 | 3 | 6 | chr1 | 217779403 | rs2820450 | G/T |
| chr1 | 218046615 | 10 | 5 | chr1 | 218046613 | rs4357535 | A/C |
| chr1 | 218623211 | 4 | 4 | chr1 | 218623210 | rs1999935 | G/T |
| chr1 | 219982932 | 3 | 1 | chr1 | 219982929 | rs4317817 | A/C |
| chr1 | 220039638 | 1 | 3 | chr1 | 220039637 | rs11118863 | A/G |
| chr1 | 220809058 | 2 | 4 | chr1 | 220809056 | rs3008642 | A/C |
| chr1 | 220869426 | 4 | 4 | chr1 | 220869425 | rs3748626 | G/T |
| chr1 | 221307796 | 8 | 2 | chr1 | 221307794 | rs28459924 | C/G |
| chr1 | 221882970 | 2 | 5 | chr1 | 221882969 | rs61825210 | A/G |
| chr1 | 222381400 | 3 | 1 | chr1 | 222381398 | rs4653563 | A/C |
| chr1 | 222381400 | 3 | 1 | chr1 | 222381399 | rs4528084 | A/G |
| chr1 | 222687993 | 3 | 11 | chr1 | 222687990 | rs2405033 | C/G |
| chr1 | 222783389 | 2 | 5 | chr1 | 222783387 | rs985300 | A/G |
| chr1 | 224065367 | 0 | 0 | chr1 | 224065366 | rs10799325 | C/G |
| chr1 | 224116271 | 3 | 3 | chr1 | 224116270 | rs360080 | A/G |
| chr1 | 224440424 | 3 | 5 | chr1 | 224440423 | rs1219675 | C/T |

| | | | | | | | |
|---|---|---|---|---|---|---|---|
| chr1 | 224793731 | 6 | 3 | chr1 | 224793730 | rs530360 | A/G |
| chr1 | 224794528 | 12 | 8 | chr1 | 224794526 | rs6668015 | C/T |
| chr1 | 224810764 | 2 | 5 | chr1 | 224810761 | rs588782 | C/T |
| chr1 | 225249198 | 4 | 3 | chr1 | 225249197 | rs1045247 | C/T |
| chr1 | 225250058 | 3 | 1 | chr1 | 225250056 | rs6664955 | C/T |
| chr1 | 225399678 | 2 | 4 | chr1 | 225399677 | rs6675599 | A/G |
| chr1 | 225776247 | 4 | 3 | chr1 | 225776246 | rs2492633 | C/T |
| chr1 | 226178904 | 7 | 1 | chr1 | 226178902 | rs10916245 | C/T |
| chr1 | 226291447 | 4 | 1 | chr1 | 226291446 | rs3121310 | A/G |
| chr1 | 226417711 | 3 | 4 | chr1 | 226417709 | rs6682260 | A/C |
| chr1 | 226501091 | 1 | 3 | chr1 | 226501089 | rs7517108 | C/T |
| chr1 | 226533374 | 2 | 2 | chr1 | 226533372 | rs3795782 | C/T |
| chr1 | 226698343 | 1 | 4 | chr1 | 226698341 | rs4653960 | C/T |
| chr1 | 227189262 | 2 | 2 | chr1 | 227189260 | rs10916402 | A/C |
| chr1 | 227228374 | 1 | 4 | chr1 | 227228372 | rs649656 | A/G |
| chr1 | 228270575 | 1 | 8 | chr1 | 228270573 | rs3124555 | C/T |
| chr1 | 228311824 | 3 | 3 | chr1 | 228311822 | rs1022663 | A/G |
| chr1 | 228826936 | 2 | 3 | chr1 | 228826935 | rs853470 | A/G |
| chr1 | 228826936 | 2 | 3 | chr1 | 228826936 | rs74142971 | C/G |
| chr1 | 228969974 | 11 | 2 | chr1 | 228969972 | rs3828126 | C/T |
| chr1 | 229503830 | 1 | 3 | chr1 | 229503829 | rs203750 | C/T |
| chr1 | 229598015 | 2 | 9 | chr1 | 229598014 | rs2486734 | C/T |
| chr1 | 229819565 | 4 | 1 | chr1 | 229819564 | rs6541275 | A/G |
| chr1 | 230133493 | 1 | 3 | chr1 | 230133491 | rs928101 | A/G |
| chr1 | 230794195 | 1 | 8 | chr1 | 230794194 | rs4649390 | A/G |
| chr1 | 230797787 | 7 | 2 | chr1 | 230797786 | rs870743 | C/T |
| chr1 | 232934274 | 2 | 7 | chr1 | 232934273 | rs675630 | C/T |
| chr1 | 232981818 | 2 | 2 | chr1 | 232981816 | rs2152769 | G/T |
| chr1 | 233120982 | 2 | 3 | chr1 | 233120979 | rs2459154 | A/G |
| chr1 | 233599147 | 2 | 6 | chr1 | 233599145 | rs6674229 | C/T |
| chr1 | 233821142 | 8 | 2 | chr1 | 233821140 | rs2841893 | C/T |
| chr1 | 233821142 | 8 | 2 | chr1 | 233821140 | rs67364353 | CA/TG |

| | | | | | | | |
|---|---|---|---|---|---|---|---|
| chr1 | 233821142 | 8 | 2 | chr1 | 233821141 | rs2774318 | A/G |
| chr1 | 233831701 | 5 | 1 | chr1 | 233831699 | rs74148718 | C/T |
| chr1 | 234321869 | 1 | 3 | chr1 | 234321868 | rs2753444 | A/G |
| chr1 | 234652927 | 2 | 2 | chr1 | 234652925 | rs668490 | A/G |
| chr1 | 234753199 | 4 | 2 | chr1 | 234753197 | rs2799419 | A/G |
| chr1 | 234798677 | 6 | 2 | chr1 | 234798676 | rs2799430 | C/T |
| chr1 | 234935356 | 1 | 5 | chr1 | 234935355 | rs12025491 | A/G |
| chr1 | 234969490 | 4 | 1 | chr1 | 234969487 | rs2288602 | A/C |
| chr1 | 235191340 | 9 | 2 | chr1 | 235191339 | rs4659759 | A/G |
| chr1 | 235732916 | 1 | 3 | chr1 | 235732915 | rs2618700 | C/G |
| chr1 | 235850937 | 6 | 4 | chr1 | 235850935 | rs489088 | A/G |
| chr1 | 237469178 | 1 | 4 | chr1 | 237469177 | rs1004788 | C/T |
| chr1 | 238449957 | 1 | 5 | chr1 | 238449956 | rs10495461 | G/T |
| chr1 | 238959979 | 4 | 1 | chr1 | 238959977 | rs12116483 | C/T |
| chr1 | 239192162 | 6 | 9 | chr1 | 239192160 | rs520831 | A/G |
| chr1 | 240119244 | 7 | 3 | chr1 | 240119243 | rs4150018 | G/T |
| chr1 | 240473337 | 3 | 2 | chr1 | 240473335 | rs35910815 | C/T |
| chr1 | 240753670 | 1 | 3 | chr1 | 240753669 | rs2246486 | A/G |
| chr1 | 240886842 | 3 | 1 | chr1 | 240886841 | rs1333694 | C/T |
| chr1 | 240942408 | 2 | 3 | chr1 | 240942406 | rs59844102 | C/T |
| chr1 | 241118043 | 2 | 3 | chr1 | 241118042 | rs7531247 | A/G |
| chr1 | 241141917 | 2 | 3 | chr1 | 241141916 | rs2502358 | A/G |
| chr1 | 241568206 | 26 | 1 | chr1 | 241568205 | rs2783963 | C/T |
| chr1 | 241950500 | 3 | 1 | chr1 | 241950499 | rs12144546 | G/T |
| chr1 | 242289135 | 3 | 1 | chr1 | 242289133 | rs12561872 | C/T |
| chr1 | 243601692 | 6 | 1 | chr1 | 243601691 | rs1173843 | C/T |
| chr1 | 243632302 | 6 | 6 | chr1 | 243632300 | rs482212 | C/T |
| chr1 | 243641364 | 4 | 2 | chr1 | 243641363 | rs1069246 | A/G |
| chr1 | 243719018 | 7 | 3 | chr1 | 243719016 | rs1538468 | A/G |
| chr1 | 243921303 | 7 | 1 | chr1 | 243921302 | rs4658796 | A/G |
| chr1 | 244177808 | 1 | 3 | chr1 | 244177807 | rs1538300 | A/G |
| chr1 | 244508443 | 4 | 2 | chr1 | 244508441 | rs10924670 | C/T |

| | | | | | | | |
|---|---|---|---|---|---|---|---|
| chr1 | 244508443 | 4 | 2 | chr1 | 244508443 | rs10924671 | A/G |
| chr1 | 244919822 | 2 | 3 | chr1 | 244919822 | rs6413860 | A/G |
| chr1 | 245525354 | 2 | 4 | chr1 | 245525353 | rs1771945 | A/G |
| chr1 | 246155491 | 8 | 3 | chr1 | 246155489 | rs4925781 | C/T |
| chr1 | 247147772 | 7 | 2 | chr1 | 247147771 | rs4489602 | A/G |
| chr2 | 108385 | 3 | 1 | chr2 | 108382 | rs1829220 | G/T |
| chr2 | 108385 | 3 | 1 | chr2 | 108384 | rs2015419 | C/T |
| chr2 | 305215 | 3 | 3 | chr2 | 305214 | rs4643574 | A/G |
| chr2 | 452798 | 3 | 1 | chr2 | 452798 | rs867926 | A/G |
| chr2 | 457148 | 1 | 5 | chr2 | 457147 | rs2724860 | A/G |
| chr2 | 585488 | 6 | 1 | chr2 | 585487 | rs2685243 | A/G |
| chr2 | 635371 | 4 | 1 | chr2 | 635371 | rs7567710 | G/T |
| chr2 | 726084 | 9 | 2 | chr2 | 726083 | rs6753840 | A/G |
| chr2 | 736036 | 1 | 8 | chr2 | 736034 | rs12617893 | C/T |
| chr2 | 786870 | 1 | 3 | chr2 | 786870 | rs4444569 | C/G |
| chr2 | 847282 | 1 | 6 | chr2 | 847280 | rs66467553 | C/T |
| chr2 | 1071348 | 1 | 3 | chr2 | 1071347 | rs4290689 | A/G |
| chr2 | 1408153 | 2 | 2 | chr2 | 1408150 | rs28461932 | C/T |
| chr2 | 1458573 | 4 | 1 | chr2 | 1458572 | rs11681539 | G/T |
| chr2 | 1505064 | 0 | 0 | chr2 | 1505063 | rs56316296 | G/T |
| chr2 | 1829849 | 3 | 3 | chr2 | 1829847 | rs4473413 | A/G |
| chr2 | 2502360 | 3 | 2 | chr2 | 2502359 | rs73181319 | A/G |
| chr2 | 2716560 | 4 | 1 | chr2 | 2716558 | rs57729419 | C/T |
| chr2 | 2723200 | 1 | 4 | chr2 | 2723199 | rs6759696 | C/T |
| chr2 | 2735759 | 8 | 2 | chr2 | 2735759 | rs2385314 | A/G |
| chr2 | 2927010 | 1 | 4 | chr2 | 2927009 | rs1868352 | C/T |
| chr2 | 3020040 | 5 | 7 | chr2 | 3020039 | rs6726453 | A/G |
| chr2 | 3058624 | 1 | 3 | chr2 | 3058623 | rs11127394 | A/G |
| chr2 | 3088635 | 5 | 3 | chr2 | 3088634 | rs7564506 | G/T |
| chr2 | 3171150 | 8 | 3 | chr2 | 3171147 | rs921666 | C/G |
| chr2 | 3221567 | 3 | 1 | chr2 | 3221565 | rs6548141 | C/T |
| chr2 | 3575850 | 9 | 1 | chr2 | 3575848 | rs10186193 | C/T |

| | | | | | | | |
|---|---|---|---|---|---|---|---|
| chr2 | 3771759 | 2 | 4 | chr2 | 3771757 | rs357978 | A/G |
| chr2 | 3799383 | 3 | 3 | chr2 | 3799382 | rs881852 | A/G |
| chr2 | 3869924 | 3 | 4 | chr2 | 3869923 | rs897065 | C/T |
| chr2 | 3882322 | 12 | 1 | chr2 | 3882320 | rs6748872 | C/T |
| chr2 | 4131979 | 3 | 2 | chr2 | 4131978 | rs68028952 | A/G |
| chr2 | 4683218 | 2 | 8 | chr2 | 4683216 | rs699550 | C/T |
| chr2 | 4905935 | 2 | 2 | chr2 | 4905933 | rs2580867 | A/G |
| chr2 | 4928241 | 2 | 7 | chr2 | 4928240 | rs12711961 | A/G |
| chr2 | 5387285 | 1 | 3 | chr2 | 5387283 | rs6734151 | C/T |
| chr2 | 5909982 | 3 | 1 | chr2 | 5909980 | rs2564012 | A/C |
| chr2 | 6053671 | 1 | 4 | chr2 | 6053669 | rs2693854 | A/G |
| chr2 | 6330338 | 3 | 1 | chr2 | 6330337 | rs1020457 | A/G |
| chr2 | 6792545 | 5 | 4 | chr2 | 6792543 | rs308029 | C/T |
| chr2 | 7046700 | 2 | 3 | chr2 | 7046698 | rs13019196 | C/T |
| chr2 | 7092170 | 2 | 2 | chr2 | 7092168 | rs441241 | C/T |
| chr2 | 7258864 | 6 | 4 | chr2 | 7258862 | rs4669152 | C/T |
| chr2 | 7548265 | 3 | 6 | chr2 | 7548262 | rs12614348 | A/C |
| chr2 | 7716281 | 1 | 4 | chr2 | 7716280 | rs6741099 | A/G |
| chr2 | 7770819 | 2 | 3 | chr2 | 7770817 | rs772452 | C/T |
| chr2 | 7794189 | 1 | 3 | chr2 | 7794188 | rs1720995 | A/C |
| chr2 | 7851128 | 2 | 3 | chr2 | 7851127 | rs885135 | C/T |
| chr2 | 8437339 | 4 | 2 | chr2 | 8437338 | rs7593528 | A/G |
| chr2 | 8644127 | 3 | 5 | chr2 | 8644126 | rs3845747 | C/T |
| chr2 | 9162982 | 2 | 4 | chr2 | 9162981 | rs2666204 | C/T |
| chr2 | 9316567 | 1 | 4 | chr2 | 9316566 | rs2709589 | C/T |
| chr2 | 9733389 | 6 | 2 | chr2 | 9733389 | rs9287712 | A/G |
| chr2 | 9820577 | 4 | 4 | chr2 | 9820575 | rs869159 | A/G |
| chr2 | 9851696 | 2 | 4 | chr2 | 9851694 | rs2357268 | C/T |
| chr2 | 9873987 | 1 | 4 | chr2 | 9873986 | rs11892895 | G/T |
| chr2 | 9907214 | 2 | 2 | chr2 | 9907212 | rs287989 | C/T |
| chr2 | 9907214 | 2 | 2 | chr2 | 9907212 | rs34886162 | -/C |
| chr2 | 9907214 | 2 | 2 | chr2 | 9907213 | rs4143793 | G/T |

| | | | | | | | |
|---|---|---|---|---|---|---|---|
| chr2 | 9907214 | 2 | 2 | chr2 | 9907214 | rs35934083 | -/G |
| chr2 | 10152027 | 2 | 2 | chr2 | 10152027 | rs7587529 | A/G |
| chr2 | 10342145 | 2 | 3 | chr2 | 10342143 | rs4669565 | C/T |
| chr2 | 10525148 | 1 | 7 | chr2 | 10525147 | rs818159 | C/G |
| chr2 | 10566309 | 2 | 4 | chr2 | 10566307 | rs818185 | C/T |
| chr2 | 10831558 | 2 | 6 | chr2 | 10831557 | rs2046793 | A/G |
| chr2 | 10889014 | 2 | 20 | chr2 | 10889012 | rs1734437 | A/G |
| chr2 | 11549430 | 1 | 6 | chr2 | 11549428 | rs4233893 | C/T |
| chr2 | 12370011 | 10 | 1 | chr2 | 12370010 | rs10929800 | A/G |
| chr2 | 12673360 | 1 | 6 | chr2 | 12673358 | rs883804 | C/T |
| chr2 | 13040439 | 2 | 2 | chr2 | 13040437 | rs10495602 | A/G |
| chr2 | 15465842 | 7 | 2 | chr2 | 15465840 | rs2031011 | C/T |
| chr2 | 15759306 | 6 | 1 | chr2 | 15759305 | rs6431727 | A/G |
| chr2 | 15900953 | 7 | 3 | chr2 | 15900951 | rs7604985 | C/T |
| chr2 | 16145238 | 8 | 2 | chr2 | 16145237 | rs28716566 | A/G |
| chr2 | 16158533 | 2 | 5 | chr2 | 16158531 | rs28458202 | C/T |
| chr2 | 16782600 | 4 | 4 | chr2 | 16782599 | rs72777989 | G/T |
| chr2 | 19335492 | 8 | 1 | chr2 | 19335491 | rs851361 | C/T |
| chr2 | 19781915 | 1 | 3 | chr2 | 19781914 | rs2881742 | C/G |
| chr2 | 20126098 | 7 | 8 | chr2 | 20126097 | rs987619 | A/G |
| chr2 | 20194820 | 4 | 5 | chr2 | 20194818 | rs975951 | C/T |
| chr2 | 20201985 | 0 | 0 | chr2 | 20201984 | rs2348315 | A/G |
| chr2 | 20601514 | 7 | 3 | chr2 | 20601512 | rs1377353 | C/T |
| chr2 | 20641237 | 2 | 3 | chr2 | 20641236 | rs2882035 | A/G |
| chr2 | 20690906 | 3 | 1 | chr2 | 20690905 | rs6531250 | A/G |
| chr2 | 20816942 | 3 | 8 | chr2 | 20816939 | rs660069 | A/C |
| chr2 | 20920253 | 12 | 1 | chr2 | 20920252 | rs13002483 | G/T |
| chr2 | 20922521 | 2 | 8 | chr2 | 20922520 | rs11675569 | A/G |
| chr2 | 21338260 | 0 | 0 | chr2 | 21338258 | rs870638 | A/C |
| chr2 | 22592014 | 3 | 1 | chr2 | 22592013 | rs12712504 | A/G |
| chr2 | 22890973 | 2 | 2 | chr2 | 22890971 | rs6711378 | C/T |
| chr2 | 23614370 | 6 | 4 | chr2 | 23614369 | rs12472268 | A/G |

| | | | | | | | |
|---|---|---|---|---|---|---|---|
| chr2 | 23632648 | 3 | 1 | chr2 | 23632647 | rs2339788 | A/G |
| chr2 | 23637626 | 4 | 4 | chr2 | 23637625 | rs1560591 | A/G |
| chr2 | 23700888 | 1 | 5 | chr2 | 23700887 | rs2339849 | G/T |
| chr2 | 24251785 | 2 | 4 | chr2 | 24251784 | rs2042467 | G/T |
| chr2 | 24820595 | 2 | 2 | chr2 | 24820594 | rs56088501 | A/G |
| chr2 | 24902351 | 3 | 2 | chr2 | 24902350 | rs2289090 | A/G |
| chr2 | 25158561 | 6 | 2 | chr2 | 25158559 | rs482546 | C/T |
| chr2 | 25292605 | 2 | 4 | chr2 | 25292604 | rs10185041 | G/T |
| chr2 | 25726806 | 11 | 1 | chr2 | 25726803 | rs10865366 | A/C |
| chr2 | 25726806 | 11 | 1 | chr2 | 25726804 | rs858664 | A/G |
| chr2 | 26057183 | 5 | 5 | chr2 | 26057181 | rs1465878 | A/G |
| chr2 | 26110345 | 3 | 5 | chr2 | 26110343 | rs4665299 | A/C |
| chr2 | 26259489 | 1 | 9 | chr2 | 26259488 | rs10779956 | A/G |
| chr2 | 27116627 | 2 | 4 | chr2 | 27116625 | rs1866654 | A/G |
| chr2 | 27157259 | 1 | 3 | chr2 | 27157258 | rs2736976 | A/G |
| chr2 | 27584445 | 2 | 3 | chr2 | 27584443 | rs1260326 | C/T |
| chr2 | 28244832 | 5 | 2 | chr2 | 28244831 | rs72816414 | A/G |
| chr2 | 28244832 | 5 | 2 | chr2 | 28244832 | rs6725130 | A/G |
| chr2 | 28429466 | 1 | 3 | chr2 | 28429464 | rs6547846 | C/T |
| chr2 | 28445438 | 1 | 3 | chr2 | 28445437 | rs1876772 | A/G |
| chr2 | 28619759 | 3 | 3 | chr2 | 28619757 | rs11127164 | C/T |
| chr2 | 28668188 | 4 | 1 | chr2 | 28668186 | rs11127176 | C/T |
| chr2 | 29613932 | 4 | 2 | chr2 | 29613930 | rs6547940 | C/T |
| chr2 | 31031898 | 3 | 2 | chr2 | 31031896 | rs73923310 | C/T |
| chr2 | 31278390 | 0 | 0 | chr2 | 31278388 | rs11692905 | C/T |
| chr2 | 31640297 | 7 | 4 | chr2 | 31640296 | rs2300698 | A/G |
| chr2 | 33214929 | 1 | 3 | chr2 | 33214928 | rs6714546 | A/G |
| chr2 | 33618213 | 1 | 4 | chr2 | 33618212 | rs11687777 | C/G |
| chr2 | 35070721 | 1 | 8 | chr2 | 35070719 | rs10167480 | C/T |
| chr2 | 35359199 | 8 | 2 | chr2 | 35359197 | rs10189794 | C/T |
| chr2 | 35707210 | 9 | 2 | chr2 | 35707208 | rs6713533 | C/T |
| chr2 | 36065736 | 4 | 1 | chr2 | 36065735 | rs1705047 | C/T |

| | | | | | | | |
|---|---|---|---|---|---|---|---|
| chr2 | 36276108 | 2 | 8 | chr2 | 36276107 | rs1167436 | C/T |
| chr2 | 37720998 | 1 | 3 | chr2 | 37720997 | rs1468664 | C/T |
| chr2 | 37724011 | 6 | 2 | chr2 | 37724011 | rs3731854 | C/G |
| chr2 | 37753525 | 2 | 4 | chr2 | 37753523 | rs2041506 | A/G |
| chr2 | 37954943 | 1 | 4 | chr2 | 37954942 | rs2565640 | A/G |
| chr2 | 37955259 | 9 | 2 | chr2 | 37955258 | rs2565641 | A/G |
| chr2 | 38119483 | 1 | 8 | chr2 | 38119481 | rs100461 | C/T |
| chr2 | 38845461 | 3 | 2 | chr2 | 38845459 | rs10865143 | C/T |
| chr2 | 38993420 | 2 | 3 | chr2 | 38993419 | rs1868867 | C/G |
| chr2 | 40294734 | 2 | 4 | chr2 | 40294732 | rs419125 | A/G |
| chr2 | 40708281 | 4 | 1 | chr2 | 40708280 | rs7576279 | A/G |
| chr2 | 41053098 | 1 | 20 | chr2 | 41053096 | rs17405921 | C/T |
| chr2 | 42311683 | 6 | 5 | chr2 | 42311681 | rs7605663 | C/T |
| chr2 | 42762456 | 3 | 1 | chr2 | 42762454 | rs13023683 | C/T |
| chr2 | 43107556 | 3 | 5 | chr2 | 43107554 | rs10187008 | C/T |
| chr2 | 45316973 | 1 | 5 | chr2 | 45316972 | rs1559479 | A/G |
| chr2 | 45338738 | 4 | 6 | chr2 | 45338736 | rs6712469 | C/T |
| chr2 | 45724114 | 3 | 1 | chr2 | 45724113 | rs475247 | A/G |
| chr2 | 45894297 | 3 | 1 | chr2 | 45894296 | rs4953262 | A/G |
| chr2 | 46123473 | 7 | 1 | chr2 | 46123472 | rs6544870 | A/G |
| chr2 | 47043147 | 15 | 10 | chr2 | 47043145 | rs12712981 | C/T |
| chr2 | 47151935 | 2 | 5 | chr2 | 47151933 | rs7569495 | C/T |
| chr2 | 47175622 | 1 | 7 | chr2 | 47175620 | rs7566404 | C/T |
| chr2 | 47348224 | 2 | 3 | chr2 | 47348223 | rs7564801 | A/G |
| chr2 | 48840584 | 4 | 3 | chr2 | 48840582 | rs2956359 | A/G |
| chr2 | 49131965 | 1 | 3 | chr2 | 49131964 | rs72827293 | A/G |
| chr2 | 52749548 | 2 | 6 | chr2 | 52749546 | rs13018204 | C/T |
| chr2 | 53289625 | 2 | 3 | chr2 | 53289624 | rs7561602 | A/G |
| chr2 | 54500543 | 2 | 2 | chr2 | 54500542 | rs60708897 | A/G |
| chr2 | 54507554 | 1 | 6 | chr2 | 54507552 | rs4510259 | C/G/T |
| chr2 | 54941044 | 6 | 1 | chr2 | 54941043 | rs67025912 | A/G |
| chr2 | 57156918 | 3 | 1 | chr2 | 57156917 | rs12988904 | A/G |

| | | | | | | | |
|---|---|---|---|---|---|---|---|
| chr2 | 60304497 | 1 | 16 | chr2 | 60304495 | rs10188396 | C/T |
| chr2 | 60419770 | 10 | 1 | chr2 | 60419769 | rs48314 | C/T |
| chr2 | 60663699 | 7 | 1 | chr2 | 60663698 | rs357002 | A/G |
| chr2 | 61670281 | 2 | 2 | chr2 | 61670280 | rs2427885 | A/G |
| chr2 | 65533992 | 1 | 6 | chr2 | 65533991 | rs1437459 | C/T |
| chr2 | 65586223 | 7 | 6 | chr2 | 65586221 | rs840979 | C/T |
| chr2 | 65628036 | 2 | 8 | chr2 | 65628035 | rs702921 | A/G |
| chr2 | 65702998 | 9 | 2 | chr2 | 65702997 | rs971410 | A/G |
| chr2 | 65809655 | 11 | 3 | chr2 | 65809653 | rs2111464 | C/T |
| chr2 | 65845051 | 4 | 4 | chr2 | 65845049 | rs2193469 | C/T |
| chr2 | 67690885 | 3 | 2 | chr2 | 67690883 | rs2861684 | C/T |
| chr2 | 68454302 | 4 | 2 | chr2 | 68454301 | rs4671208 | A/G |
| chr2 | 68872776 | 3 | 3 | chr2 | 68872775 | rs4854517 | A/G |
| chr2 | 70661842 | 3 | 4 | chr2 | 70661841 | rs12619641 | A/G |
| chr2 | 70722475 | 2 | 7 | chr2 | 70722473 | rs12616862 | C/T |
| chr2 | 70725802 | 0 | 0 | chr2 | 70725800 | rs880848 | A/G |
| chr2 | 71018142 | 0 | 0 | chr2 | 71018141 | rs7588170 | A/G |
| chr2 | 71056057 | 1 | 5 | chr2 | 71056056 | rs17664519 | A/G |
| chr2 | 71145335 | 1 | 6 | chr2 | 71145334 | rs13407019 | A/G |
| chr2 | 71412953 | 8 | 4 | chr2 | 71412952 | rs3771382 | C/G |
| chr2 | 71913013 | 1 | 5 | chr2 | 71913011 | rs11679946 | C/T |
| chr2 | 72943508 | 3 | 5 | chr2 | 72943508 | rs4852902 | A/G |
| chr2 | 72948846 | 7 | 2 | chr2 | 72948845 | rs7570103 | A/G |
| chr2 | 73192938 | 1 | 5 | chr2 | 73192937 | rs4143146 | G/T |
| chr2 | 73243225 | 2 | 2 | chr2 | 73243223 | rs6546810 | C/T |
| chr2 | 73494586 | 1 | 8 | chr2 | 73494584 | rs2421546 | A/C |
| chr2 | 73860646 | 4 | 1 | chr2 | 73860643 | rs2272051 | A/G |
| chr2 | 73860853 | 4 | 1 | chr2 | 73860852 | rs3813228 | A/G |
| chr2 | 74005689 | 7 | 3 | chr2 | 74005686 | rs831524 | C/G |
| chr2 | 76194339 | 3 | 7 | chr2 | 76194338 | rs11886371 | C/G/T |
| chr2 | 76199748 | 4 | 4 | chr2 | 76199746 | rs6759874 | C/T |
| chr2 | 76395846 | 3 | 1 | chr2 | 76395845 | rs6718886 | A/G |

| | | | | | | | |
|---|---|---|---|---|---|---|---|
| chr2 | 76770070 | 4 | 1 | chr2 | 76770069 | rs17404655 | G/T |
| chr2 | 76871837 | 5 | 2 | chr2 | 76871836 | rs4853273 | A/G |
| chr2 | 79096111 | 5 | 2 | chr2 | 79096109 | rs425048 | A/G |
| chr2 | 80369162 | 3 | 3 | chr2 | 80369160 | rs56903490 | C/T |
| chr2 | 80607648 | 2 | 2 | chr2 | 80607646 | rs1434071 | G/T |
| chr2 | 81084618 | 2 | 2 | chr2 | 81084616 | rs3979265 | C/T |
| chr2 | 82296048 | 10 | 3 | chr2 | 82296046 | rs878818 | C/G |
| chr2 | 83628318 | 5 | 1 | chr2 | 83628317 | rs62153548 | A/G |
| chr2 | 84295848 | 1 | 7 | chr2 | 84295847 | rs6718550 | A/G |
| chr2 | 84611107 | 13 | 3 | chr2 | 84611105 | rs1460695 | A/G |
| chr2 | 84687575 | 15 | 3 | chr2 | 84687573 | rs4276039 | C/T |
| chr2 | 85537721 | 5 | 1 | chr2 | 85537720 | rs6716108 | A/G |
| chr2 | 85829748 | 2 | 3 | chr2 | 85829746 | rs13395555 | C/T |
| chr2 | 87486384 | 4 | 2 | chr2 | 87486383 | rs57283050 | A/G |
| chr2 | 88396799 | 1 | 5 | chr2 | 88396798 | rs1258415 | C/T |
| chr2 | 90999541 | 27 | 15 | chr2 | 90999539 | rs578305 | A/G |
| chr2 | 90999618 | 11 | 33 | chr2 | 90999617 | rs635550 | C/T |
| chr2 | 91140910 | 4 | 4 | chr2 | 91140907 | rs2248711 | C/G |
| chr2 | 91140910 | 4 | 4 | chr2 | 91140907 | rs71262893 | CC/GG |
| chr2 | 91140910 | 4 | 4 | chr2 | 91140908 | rs2386107 | C/G |
| chr2 | 91141002 | 26 | 6 | chr2 | 91141000 | rs1809097 | C/T |
| chr2 | 91166312 | 2 | 4 | chr2 | 91166311 | rs4927492 | C/T |
| chr2 | 91166312 | 2 | 4 | chr2 | 91166311 | rs67992558 | ATGAGAATTCCG/CTGAGAATTCCA |
| chr2 | 91168636 | 3 | 1 | chr2 | 91168634 | rs2531394 | C/G |
| chr2 | 91232627 | 4 | 2 | chr2 | 91232625 | rs71221119 | A/G |
| chr2 | 96145714 | 1 | 3 | chr2 | 96145712 | rs3111873 | C/G |
| chr2 | 98356133 | 5 | 1 | chr2 | 98356131 | rs3769754 | G/T |
| chr2 | 100612278 | 8 | 3 | chr2 | 100612277 | rs13025521 | A/G |
| chr2 | 100652455 | 7 | 3 | chr2 | 100652454 | rs10201136 | A/G |
| chr2 | 100791825 | 4 | 1 | chr2 | 100791823 | rs72627409 | C/T |
| chr2 | 100925337 | 2 | 4 | chr2 | 100925335 | rs7605570 | C/T |
| chr2 | 100966107 | 5 | 4 | chr2 | 100966105 | rs3768990 | C/T |

| | | | | | | | |
|---|---|---|---|---|---|---|---|
| chr2 | 101296199 | 1 | 4 | chr2 | 101296197 | rs12712095 | C/T |
| chr2 | 101296199 | 1 | 4 | chr2 | 101296197 | rs36114027 | -/G |
| chr2 | 101900315 | 4 | 2 | chr2 | 101900313 | rs7603475 | C/T |
| chr2 | 101957725 | 2 | 8 | chr2 | 101957724 | rs10179894 | A/G |
| chr2 | 102359108 | 1 | 5 | chr2 | 102359106 | rs2270297 | A/G |
| chr2 | 102469666 | 1 | 3 | chr2 | 102469663 | rs10175045 | C/T |
| chr2 | 102505257 | 13 | 3 | chr2 | 102505256 | rs6750971 | A/G |
| chr2 | 102571607 | 3 | 4 | chr2 | 102571606 | rs17027517 | A/G |
| chr2 | 102924989 | 11 | 1 | chr2 | 102924987 | rs6726414 | A/C |
| chr2 | 104423286 | 4 | 5 | chr2 | 104423285 | rs6543255 | A/G |
| chr2 | 104728793 | 7 | 12 | chr2 | 104728792 | rs4851040 | A/G |
| chr2 | 104922301 | 1 | 5 | chr2 | 104922300 | rs7595767 | G/T |
| chr2 | 105291514 | 1 | 3 | chr2 | 105291512 | rs873738 | C/T |
| chr2 | 105345939 | 3 | 2 | chr2 | 105345937 | rs2278501 | C/T |
| chr2 | 105837826 | 3 | 9 | chr2 | 105837824 | rs6543345 | C/T |
| chr2 | 106009939 | 7 | 1 | chr2 | 106009937 | rs72832974 | C/T |
| chr2 | 106341052 | 2 | 3 | chr2 | 106341050 | rs4234103 | C/T |
| chr2 | 106467292 | 4 | 3 | chr2 | 106467290 | rs71251056 | A/C |
| chr2 | 107242931 | 1 | 4 | chr2 | 107242929 | rs73951056 | C/T |
| chr2 | 107242931 | 1 | 4 | chr2 | 107242930 | rs995720 | A/G |
| chr2 | 107735979 | 6 | 1 | chr2 | 107735979 | rs700859 | A/G |
| chr2 | 108271481 | 3 | 2 | chr2 | 108271479 | rs2305485 | C/T |
| chr2 | 108559939 | 1 | 6 | chr2 | 108559938 | rs10205370 | A/G |
| chr2 | 108595155 | 1 | 3 | chr2 | 108595154 | rs2577601 | C/T |
| chr2 | 109001237 | 5 | 3 | chr2 | 109001235 | rs7568610 | C/T |
| chr2 | 109107989 | 2 | 3 | chr2 | 109107987 | rs12712029 | C/T |
| chr2 | 109156454 | 2 | 2 | chr2 | 109156452 | rs2163259 | A/G |
| chr2 | 109167518 | 2 | 2 | chr2 | 109167517 | rs4676074 | A/G |
| chr2 | 111217925 | 3 | 1 | chr2 | 111217923 | rs55927063 | C/T |
| chr2 | 111285880 | 1 | 3 | chr2 | 111285879 | rs72832841 | A/G |
| chr2 | 111311960 | 2 | 3 | chr2 | 111311959 | rs12463442 | A/G |
| chr2 | 111548192 | 2 | 8 | chr2 | 111548189 | rs1439288 | A/G |

| | | | | | | | |
|---|---|---|---|---|---|---|---|
| chr2 | 111548192 | 2 | 8 | chr2 | 111548189 | rs71405705 | CCG/TCA |
| chr2 | 111548192 | 2 | 8 | chr2 | 111548191 | rs35644645 | A/G |
| chr2 | 111548192 | 2 | 8 | chr2 | 111548191 | rs34791521 | -/A |
| chr2 | 111652930 | 3 | 1 | chr2 | 111652929 | rs10439381 | A/G |
| chr2 | 113016866 | 4 | 2 | chr2 | 113016864 | rs4849072 | A/C |
| chr2 | 113115616 | 1 | 6 | chr2 | 113115614 | rs1568113 | C/T |
| chr2 | 113602855 | 5 | 9 | chr2 | 113602854 | rs1794067 | A/G |
| chr2 | 115636146 | 3 | 1 | chr2 | 115636146 | rs7557011 | C/G |
| chr2 | 115636146 | 3 | 1 | chr2 | 115636146 | rs67216957 | CT/GC |
| chr2 | 117768360 | 5 | 5 | chr2 | 117768359 | rs1349741 | C/T |
| chr2 | 118121603 | 2 | 2 | chr2 | 118121601 | rs62192727 | C/T |
| chr2 | 118644190 | 3 | 4 | chr2 | 118644189 | rs11123476 | A/G |
| chr2 | 119322057 | 5 | 1 | chr2 | 119322055 | rs11123496 | C/G |
| chr2 | 119322232 | 4 | 1 | chr2 | 119322229 | rs3731613 | G/T |
| chr2 | 119327497 | 2 | 2 | chr2 | 119327495 | rs62159867 | C/T |
| chr2 | 120211861 | 6 | 2 | chr2 | 120211859 | rs13419800 | C/T |
| chr2 | 120851066 | 5 | 7 | chr2 | 120851064 | rs7602467 | C/T |
| chr2 | 121160732 | 1 | 3 | chr2 | 121160731 | rs11122807 | G/T |
| chr2 | 121293828 | 3 | 1 | chr2 | 121293827 | rs6541740 | A/G |
| chr2 | 121296336 | 4 | 2 | chr2 | 121296335 | rs11122822 | A/G |
| chr2 | 121346329 | 1 | 3 | chr2 | 121346328 | rs2871873 | A/G |
| chr2 | 121403004 | 3 | 5 | chr2 | 121403002 | rs4848649 | C/T |
| chr2 | 121445793 | 3 | 5 | chr2 | 121445792 | rs2677507 | C/T |
| chr2 | 121508944 | 6 | 1 | chr2 | 121508943 | rs2592608 | C/T |
| chr2 | 121522155 | 1 | 6 | chr2 | 121522153 | rs2677530 | G/T |
| chr2 | 121541369 | 2 | 5 | chr2 | 121541367 | rs2677510 | A/G |
| chr2 | 121626238 | 3 | 1 | chr2 | 121626237 | rs36079691 | A/G |
| chr2 | 122292555 | 2 | 6 | chr2 | 122292554 | rs11679195 | A/G |
| chr2 | 122444857 | 3 | 1 | chr2 | 122444855 | rs11894285 | C/T |
| chr2 | 123298693 | 3 | 1 | chr2 | 123298692 | rs159395 | C/T |
| chr2 | 124112615 | 6 | 2 | chr2 | 124112613 | rs2670609 | A/G |
| chr2 | 124385671 | 2 | 2 | chr2 | 124385670 | rs1214070 | C/T |

| | | | | | | | |
|---|---|---|---|---|---|---|---|
| chr2 | 126138788 | 4 | 6 | chr2 | 126138787 | rs1593682 | C/G |
| chr2 | 126432299 | 1 | 4 | chr2 | 126432298 | rs56119302 | G/T |
| chr2 | 127059451 | 4 | 1 | chr2 | 127059450 | rs6707983 | A/G |
| chr2 | 127271441 | 5 | 4 | chr2 | 127271438 | rs13410315 | C/T |
| chr2 | 127271441 | 5 | 4 | chr2 | 127271438 | rs34704270 | CC/TT |
| chr2 | 127271441 | 5 | 4 | chr2 | 127271439 | rs13410316 | C/T |
| chr2 | 127440454 | 5 | 1 | chr2 | 127440453 | rs6431207 | A/G |
| chr2 | 128172946 | 2 | 4 | chr2 | 128172944 | rs11891672 | C/T |
| chr2 | 128251492 | 1 | 4 | chr2 | 128251491 | rs6756562 | A/G |
| chr2 | 128442852 | 4 | 2 | chr2 | 128442851 | rs2917657 | A/G |
| chr2 | 128683214 | 4 | 2 | chr2 | 128683212 | rs7563710 | C/T |
| chr2 | 129265372 | 1 | 3 | chr2 | 129265372 | rs2406263 | A/G |
| chr2 | 129490073 | 1 | 3 | chr2 | 129490072 | rs11883895 | A/G |
| chr2 | 129714914 | 1 | 4 | chr2 | 129714912 | rs2952621 | C/T |
| chr2 | 129932399 | 6 | 1 | chr2 | 129932398 | rs4662943 | A/G |
| chr2 | 130033474 | 7 | 1 | chr2 | 130033473 | rs4662960 | A/G |
| chr2 | 130632885 | 1 | 5 | chr2 | 130632884 | rs2084784 | A/G |
| chr2 | 131388742 | 3 | 5 | chr2 | 131388740 | rs6752724 | C/T |
| chr2 | 131401808 | 1 | 6 | chr2 | 131401806 | rs12162378 | C/T |
| chr2 | 132728738 | 198 | 3 | chr2 | 132728736 | rs62164990 | C/T |
| chr2 | 132728780 | 2 | 4 | chr2 | 132728778 | rs62164992 | A/G |
| chr2 | 132729084 | 214 | 12 | chr2 | 132729081 | rs11883978 | C/T |
| chr2 | 132730895 | 36 | 24 | chr2 | 132730892 | rs72869458 | C/G |
| chr2 | 132731148 | 3 | 5 | chr2 | 132731147 | rs72869462 | A/G |
| chr2 | 132742203 | 527 | 101 | chr2 | 132742201 | rs4381828 | C/T |
| chr2 | 132747214 | 9 | 26 | chr2 | 132747212 | rs62167520 | C/T |
| chr2 | 132747214 | 9 | 26 | chr2 | 132747213 | rs1064883 | A/G |
| chr2 | 132770159 | 2 | 2 | chr2 | 132770158 | rs13023440 | A/G |
| chr2 | 132983144 | 1 | 3 | chr2 | 132983142 | rs12691821 | C/T |
| chr2 | 136642693 | 11 | 1 | chr2 | 136642691 | rs10928558 | C/T |
| chr2 | 136956774 | 4 | 6 | chr2 | 136956773 | rs2033172 | A/G |
| chr2 | 139424701 | 7 | 1 | chr2 | 139424699 | rs1351924 | A/G |

| | | | | | | | |
|---|---|---|---|---|---|---|---|
| chr2 | 140252146 | 2 | 3 | chr2 | 140252144 | rs12692014 | C/T |
| chr2 | 141070816 | 2 | 3 | chr2 | 141070815 | rs540441 | C/G |
| chr2 | 141525772 | 2 | 2 | chr2 | 141525771 | rs1922710 | A/G |
| chr2 | 141526715 | 2 | 6 | chr2 | 141526713 | rs2380922 | C/T |
| chr2 | 141526715 | 2 | 6 | chr2 | 141526713 | rs34619188 | -/C |
| chr2 | 141560913 | 6 | 1 | chr2 | 141560912 | rs7575215 | A/G |
| chr2 | 142284381 | 3 | 1 | chr2 | 142284379 | rs12990449 | C/T |
| chr2 | 143995705 | 3 | 2 | chr2 | 143995704 | rs5022663 | A/G |
| chr2 | 144040743 | 3 | 1 | chr2 | 144040741 | rs7601537 | C/T |
| chr2 | 144283142 | 10 | 1 | chr2 | 144283140 | rs1882623 | C/T |
| chr2 | 144503743 | 3 | 3 | chr2 | 144503742 | rs72852992 | A/G |
| chr2 | 148046277 | 2 | 2 | chr2 | 148046276 | rs12691759 | A/G |
| chr2 | 149032718 | 4 | 4 | chr2 | 149032716 | rs12691798 | C/T |
| chr2 | 150393437 | 2 | 3 | chr2 | 150393436 | rs72862730 | A/G |
| chr2 | 150393437 | 2 | 3 | chr2 | 150393437 | rs35540461 | -/G |
| chr2 | 150826891 | 13 | 1 | chr2 | 150826889 | rs7598384 | A/C |
| chr2 | 152604833 | 1 | 8 | chr2 | 152604832 | rs2709766 | A/G |
| chr2 | 152799329 | 3 | 3 | chr2 | 152799327 | rs2345901 | C/T |
| chr2 | 153369269 | 4 | 3 | chr2 | 153369266 | rs1896291 | G/T |
| chr2 | 153532030 | 2 | 4 | chr2 | 153532029 | rs10931408 | A/G |
| chr2 | 153541956 | 2 | 2 | chr2 | 153541955 | rs12995185 | A/G |
| chr2 | 153839291 | 3 | 2 | chr2 | 153839290 | rs34988460 | A/G |
| chr2 | 154383519 | 3 | 1 | chr2 | 154383518 | rs1881205 | A/G |
| chr2 | 155197116 | 3 | 3 | chr2 | 155197114 | rs13421974 | C/T |
| chr2 | 155974508 | 4 | 1 | chr2 | 155974506 | rs11888169 | C/T |
| chr2 | 156419324 | 5 | 2 | chr2 | 156419322 | rs11885215 | C/T |
| chr2 | 158960060 | 1 | 3 | chr2 | 158960059 | rs2042081 | A/G |
| chr2 | 159310921 | 7 | 7 | chr2 | 159310919 | rs1515925 | C/G |
| chr2 | 159741420 | 2 | 9 | chr2 | 159741419 | rs759034 | A/G |
| chr2 | 159792509 | 3 | 1 | chr2 | 159792508 | rs2042495 | A/G |
| chr2 | 159836151 | 3 | 1 | chr2 | 159836150 | rs174267 | A/G |
| chr2 | 160572228 | 1 | 4 | chr2 | 160572226 | rs2667007 | C/T |

| | | | | | | | |
|---|---|---|---|---|---|---|---|
| chr2 | 161813983 | 5 | 11 | chr2 | 161813982 | rs10168014 | A/G |
| chr2 | 164120726 | 2 | 3 | chr2 | 164120725 | rs4668418 | A/G |
| chr2 | 165851447 | 4 | 2 | chr2 | 165851444 | rs60606324 | A/C |
| chr2 | 165851447 | 4 | 2 | chr2 | 165851445 | rs353136 | A/C |
| chr2 | 165883575 | 1 | 3 | chr2 | 165883574 | rs13023748 | C/G |
| chr2 | 169709556 | 6 | 1 | chr2 | 169709555 | rs4668122 | A/G |
| chr2 | 169927063 | 5 | 2 | chr2 | 169927061 | rs1559013 | C/T |
| chr2 | 170137462 | 2 | 3 | chr2 | 170137460 | rs2683457 | C/T |
| chr2 | 171065220 | 2 | 4 | chr2 | 171065218 | rs6433208 | C/T |
| chr2 | 171078083 | 2 | 2 | chr2 | 171078081 | rs2010922 | C/T |
| chr2 | 172462801 | 3 | 4 | chr2 | 172462799 | rs6753859 | C/T |
| chr2 | 174189128 | 1 | 3 | chr2 | 174189126 | rs755816 | A/G |
| chr2 | 174583058 | 2 | 3 | chr2 | 174583056 | rs13028699 | C/T |
| chr2 | 175256010 | 4 | 6 | chr2 | 175256009 | rs74173160 | A/G |
| chr2 | 175816727 | 4 | 2 | chr2 | 175816726 | rs268210 | C/T |
| chr2 | 176937307 | 1 | 3 | chr2 | 176937305 | rs2203960 | A/G |
| chr2 | 177391879 | 3 | 1 | chr2 | 177391878 | rs840693 | A/G |
| chr2 | 177640484 | 2 | 2 | chr2 | 177640482 | rs1534682 | C/T |
| chr2 | 177658696 | 4 | 2 | chr2 | 177658693 | rs4145329 | C/T |
| chr2 | 178126821 | 2 | 6 | chr2 | 178126820 | rs1348850 | A/G |
| chr2 | 179608754 | 3 | 2 | chr2 | 179608752 | rs7598203 | C/T |
| chr2 | 180248610 | 2 | 2 | chr2 | 180248608 | rs13419649 | A/C |
| chr2 | 182108231 | 8 | 1 | chr2 | 182108230 | rs4666787 | A/G |
| chr2 | 184089467 | 6 | 1 | chr2 | 184089465 | rs12996552 | C/T |
| chr2 | 184620894 | 3 | 1 | chr2 | 184620892 | rs35066125 | C/T |
| chr2 | 185911642 | 3 | 3 | chr2 | 185911641 | rs850875 | A/G |
| chr2 | 186934835 | 3 | 4 | chr2 | 186934834 | rs1448854 | C/T |
| chr2 | 186996786 | 1 | 5 | chr2 | 186996784 | rs4667091 | C/T |
| chr2 | 187068685 | 6 | 3 | chr2 | 187068684 | rs6434182 | A/G |
| chr2 | 189317903 | 2 | 2 | chr2 | 189317902 | rs781245 | A/G |
| chr2 | 189923772 | 2 | 2 | chr2 | 189923771 | rs10177513 | A/G |
| chr2 | 189963220 | 2 | 2 | chr2 | 189963217 | rs1520864 | C/T |

| | | | | | | | |
|---|---|---|---|---|---|---|---|
| chr2 | 189963220 | 2 | 2 | chr2 | 189963219 | rs58926658 | A/G |
| chr2 | 190338379 | 4 | 3 | chr2 | 190338377 | rs1225120 | C/T |
| chr2 | 190889889 | 2 | 3 | chr2 | 190889888 | rs291461 | G/T |
| chr2 | 191729562 | 5 | 2 | chr2 | 191729561 | rs7575463 | A/G |
| chr2 | 191764883 | 3 | 1 | chr2 | 191764881 | rs10804038 | C/T |
| chr2 | 192125992 | 3 | 1 | chr2 | 192125991 | rs4586589 | A/G |
| chr2 | 195709700 | 2 | 2 | chr2 | 195709698 | rs938066 | C/T |
| chr2 | 195737644 | 8 | 2 | chr2 | 195737643 | rs6434740 | A/G |
| chr2 | 195888397 | 5 | 1 | chr2 | 195888396 | rs1464711 | C/T |
| chr2 | 197777595 | 2 | 7 | chr2 | 197777594 | rs1881405 | C/T |
| chr2 | 199351367 | 3 | 2 | chr2 | 199351366 | rs938856 | A/G |
| chr2 | 199850881 | 4 | 1 | chr2 | 199850879 | rs12105045 | C/T |
| chr2 | 200408856 | 2 | 2 | chr2 | 200408854 | rs2949005 | C/T |
| chr2 | 201172541 | 5 | 2 | chr2 | 201172539 | rs10179964 | C/T |
| chr2 | 202102306 | 5 | 2 | chr2 | 202102304 | rs4675147 | C/T |
| chr2 | 202677932 | 5 | 5 | chr2 | 202677931 | rs6746162 | A/G |
| chr2 | 203060984 | 4 | 4 | chr2 | 203060983 | rs6750950 | A/G |
| chr2 | 203162256 | 4 | 2 | chr2 | 203162255 | rs11679006 | C/G |
| chr2 | 204979115 | 4 | 7 | chr2 | 204979114 | rs4347813 | A/G |
| chr2 | 204979115 | 4 | 7 | chr2 | 204979115 | rs73982231 | A/G |
| chr2 | 206301876 | 3 | 2 | chr2 | 206301874 | rs849524 | A/G |
| chr2 | 206380533 | 5 | 4 | chr2 | 206380531 | rs4675544 | C/T |
| chr2 | 206479787 | 2 | 2 | chr2 | 206479785 | rs6435317 | C/T |
| chr2 | 207780481 | 1 | 16 | chr2 | 207780479 | rs1263677 | A/G |
| chr2 | 209172927 | 3 | 1 | chr2 | 209172926 | rs1594272 | A/G |
| chr2 | 209856175 | 5 | 2 | chr2 | 209856175 | rs12470898 | A/G |
| chr2 | 211448433 | 1 | 8 | chr2 | 211448431 | rs6741683 | C/T |
| chr2 | 212147107 | 5 | 1 | chr2 | 212147105 | rs12694242 | C/T |
| chr2 | 212147107 | 5 | 1 | chr2 | 212147106 | rs58679637 | A/G |
| chr2 | 212431705 | 1 | 3 | chr2 | 212431703 | rs1025752 | A/G |
| chr2 | 212897041 | 2 | 3 | chr2 | 212897039 | rs12694277 | C/T |
| chr2 | 215280757 | 6 | 1 | chr2 | 215280755 | rs11674080 | C/T |

| | | | | | | | |
|---|---|---|---|---|---|---|---|
| chr2 | 215382183 | 0 | 0 | chr2 | 215382181 | rs13021937 | C/T |
| chr2 | 215484387 | 7 | 2 | chr2 | 215484385 | rs12614543 | C/T |
| chr2 | 215774291 | 4 | 2 | chr2 | 215774290 | rs10932596 | A/G |
| chr2 | 216293283 | 4 | 2 | chr2 | 216293281 | rs1011146 | C/T |
| chr2 | 216572480 | 4 | 2 | chr2 | 216572479 | rs1344697 | A/G |
| chr2 | 217433396 | 7 | 2 | chr2 | 217433395 | rs1179734 | C/T |
| chr2 | 218218315 | 2 | 7 | chr2 | 218218313 | rs12987800 | C/T |
| chr2 | 218326820 | 2 | 11 | chr2 | 218326818 | rs12466441 | C/T |
| chr2 | 218391399 | 2 | 3 | chr2 | 218391398 | rs2571445 | C/T |
| chr2 | 218512922 | 4 | 2 | chr2 | 218512920 | rs3791905 | A/G |
| chr2 | 218767664 | 7 | 1 | chr2 | 218767663 | rs7574838 | A/G |
| chr2 | 218859463 | 6 | 4 | chr2 | 218859461 | rs2382817 | A/C |
| chr2 | 219634413 | 4 | 1 | chr2 | 219634411 | rs437512 | A/G |
| chr2 | 219954464 | 8 | 4 | chr2 | 219954461 | rs6436148 | C/T |
| chr2 | 220069680 | 0 | 0 | chr2 | 220069678 | rs1567489 | C/G |
| chr2 | 220131018 | 5 | 2 | chr2 | 220131017 | rs2278201 | A/G |
| chr2 | 220136241 | 4 | 7 | chr2 | 220136239 | rs11904583 | C/T |
| chr2 | 220189676 | 4 | 5 | chr2 | 220189674 | rs652509 | A/G |
| chr2 | 220190073 | 3 | 1 | chr2 | 220190072 | rs672893 | A/G |
| chr2 | 222698259 | 3 | 2 | chr2 | 222698258 | rs16863422 | A/G |
| chr2 | 222828532 | 4 | 4 | chr2 | 222828531 | rs6724843 | A/G |
| chr2 | 223528241 | 2 | 3 | chr2 | 223528240 | rs35263043 | A/G |
| chr2 | 223715267 | 3 | 1 | chr2 | 223715267 | rs1448305 | C/T |
| chr2 | 224501191 | 6 | 2 | chr2 | 224501189 | rs12991916 | C/T |
| chr2 | 224668315 | 1 | 5 | chr2 | 224668314 | rs998708 | G/T |
| chr2 | 224709893 | 4 | 2 | chr2 | 224709891 | rs11883466 | C/T |
| chr2 | 224709893 | 4 | 2 | chr2 | 224709892 | rs10933043 | G/T |
| chr2 | 224716714 | 2 | 2 | chr2 | 224716713 | rs13430129 | A/G |
| chr2 | 225504237 | 1 | 5 | chr2 | 225504236 | rs9808432 | A/G |
| chr2 | 225507351 | 4 | 4 | chr2 | 225507350 | rs13033698 | A/G |
| chr2 | 227865833 | 1 | 4 | chr2 | 227865831 | rs4261710 | C/T |
| chr2 | 228174384 | 3 | 1 | chr2 | 228174383 | rs6710402 | C/G |

| | | | | | | | |
|---|---|---|---|---|---|---|---|
| chr2 | 228231481 | 1 | 5 | chr2 | 228231480 | rs10933201 | A/G |
| chr2 | 229432248 | 2 | 2 | chr2 | 229432246 | rs4303709 | C/T |
| chr2 | 229434957 | 2 | 3 | chr2 | 229434956 | rs13002075 | G/T |
| chr2 | 229786153 | 4 | 3 | chr2 | 229786151 | rs10804351 | C/T |
| chr2 | 231093192 | 3 | 1 | chr2 | 231093190 | rs890670 | A/G |
| chr2 | 231477315 | 1 | 4 | chr2 | 231477314 | rs11889992 | A/G |
| chr2 | 231558865 | 1 | 4 | chr2 | 231558864 | rs2369000 | C/T |
| chr2 | 231752929 | 2 | 3 | chr2 | 231752927 | rs6755467 | A/C |
| chr2 | 231763143 | 4 | 2 | chr2 | 231763140 | rs55748426 | C/G |
| chr2 | 232206287 | 3 | 1 | chr2 | 232206286 | rs12694899 | A/G |
| chr2 | 232247161 | 5 | 9 | chr2 | 232247159 | rs12616152 | C/T |
| chr2 | 233475281 | 2 | 7 | chr2 | 233475280 | rs4973569 | A/G |
| chr2 | 233501456 | 1 | 4 | chr2 | 233501454 | rs73102769 | C/T |
| chr2 | 233663968 | 3 | 2 | chr2 | 233663966 | rs4335931 | C/T |
| chr2 | 234343407 | 2 | 2 | chr2 | 234343406 | rs12474441 | G/T |
| chr2 | 234382408 | 5 | 5 | chr2 | 234382407 | rs10803663 | A/G |
| chr2 | 234548684 | 7 | 2 | chr2 | 234548682 | rs13414162 | C/T |
| chr2 | 234873944 | 6 | 1 | chr2 | 234873942 | rs2363118 | A/G |
| chr2 | 235765697 | 7 | 2 | chr2 | 235765695 | rs10929104 | C/T |
| chr2 | 235821134 | 4 | 1 | chr2 | 235821132 | rs4547480 | A/C |
| chr2 | 235834415 | 2 | 2 | chr2 | 235834414 | rs7568030 | A/G |
| chr2 | 235845411 | 4 | 2 | chr2 | 235845410 | rs2043194 | A/G |
| chr2 | 235877187 | 2 | 3 | chr2 | 235877186 | rs2316019 | A/G |
| chr2 | 236200135 | 1 | 3 | chr2 | 236200133 | rs4233622 | C/T |
| chr2 | 236218099 | 3 | 2 | chr2 | 236218098 | rs13018025 | A/G |
| chr2 | 236430474 | 3 | 1 | chr2 | 236430473 | rs10202398 | A/G |
| chr2 | 236477361 | 5 | 7 | chr2 | 236477359 | rs7601516 | C/G |
| chr2 | 236497312 | 4 | 2 | chr2 | 236497311 | rs7571708 | A/G |
| chr2 | 236601117 | 2 | 2 | chr2 | 236601115 | rs11692136 | C/T |
| chr2 | 237022132 | 5 | 2 | chr2 | 237022130 | rs6743304 | C/T |
| chr2 | 237295034 | 1 | 5 | chr2 | 237295033 | rs10196267 | A/G |
| chr2 | 237472637 | 3 | 10 | chr2 | 237472636 | rs13391650 | A/G |

| | | | | | | | | |
|---|---|---|---|---|---|---|---|---|
| chr2 | 238459443 | 8 | 6 | chr2 | 238459442 | rs302670 | | C/T |
| chr2 | 238586143 | 4 | 1 | chr2 | 238586141 | rs2028929 | | A/G |
| chr2 | 238703653 | 3 | 5 | chr2 | 238703650 | rs57813708 | | C/T |
| chr2 | 239169712 | 1 | 3 | chr2 | 239169711 | rs4663317 | | A/G |
| chr2 | 239525276 | 4 | 1 | chr2 | 239525274 | rs11895857 | | C/T |
| chr2 | 239554541 | 4 | 4 | chr2 | 239554539 | rs11124202 | | C/T |
| chr2 | 240364930 | 2 | 3 | chr2 | 240364928 | rs6730007 | | C/T |
| chr2 | 240365550 | 1 | 4 | chr2 | 240365548 | rs7580603 | | C/T |
| chr2 | 240376757 | 1 | 5 | chr2 | 240376755 | rs10865084 | | C/T |
| chr2 | 240603588 | 2 | 4 | chr2 | 240603588 | rs3792082 | | A/C |
| chr2 | 240725728 | 1 | 5 | chr2 | 240725726 | rs12473823 | | A/C |
| chr2 | 240825092 | 5 | 1 | chr2 | 240825090 | rs4854081 | | A/G |
| chr2 | 241000429 | 1 | 4 | chr2 | 241000428 | rs1316369 | | C/T |
| chr2 | 241271745 | 3 | 4 | chr2 | 241271743 | rs1968423 | | A/G |
| chr2 | 241275334 | 4 | 2 | chr2 | 241275333 | rs6437366 | | C/G |
| chr2 | 241279186 | 4 | 3 | chr2 | 241279185 | rs1968423 | | A/G |
| chr2 | 241279186 | 4 | 3 | chr2 | 241279185 | rs71363916 | | A/G |
| chr2 | 241364978 | 6 | 1 | chr2 | 241364977 | rs4676369 | | C/T |
| chr2 | 241462455 | 0 | 0 | chr2 | 241462454 | rs10933640 | | A/G |
| chr2 | 241476292 | 4 | 2 | chr2 | 241476290 | rs6707763 | | C/T |
| chr2 | 241476707 | 8 | 4 | chr2 | 241476706 | rs6707568 | | A/G |
| chr2 | 241481237 | 1 | 3 | chr2 | 241481236 | rs12105664 | | C/G |
| chr2 | 241492425 | 4 | 4 | chr2 | 241492424 | rs10185785 | | A/G |
| chr2 | 241515601 | 3 | 3 | chr2 | 241515600 | rs6745171 | | A/G |
| chr2 | 241792415 | 14 | 1 | chr2 | 241792413 | rs11683405 | | C/T |
| chr2 | 242138591 | 1 | 3 | chr2 | 242138589 | rs4675935 | | A/G |
| chr2 | 242262435 | 3 | 2 | chr2 | 242262432 | rs7559229 | | C/T |
| chr2 | 242333618 | 2 | 4 | chr2 | 242333617 | rs35532480 | | A/G |
| chr2 | 242363895 | 2 | 3 | chr2 | 242363893 | rs11899246 | | C/T |
| chr2 | 242399134 | 1 | 4 | chr2 | 242399132 | rs34748986 | | C/T |
| chr2 | 242471423 | 6 | 1 | chr2 | 242471422 | rs10427182 | | G/T |
| chr2 | 242543613 | 1 | 3 | chr2 | 242543610 | rs36120366 | | C/G |

| | | | | | | | |
|---|---|---|---|---|---|---|---|
| chr2 | 242564650 | 5 | 2 | chr2 | 242564648 | rs34993180 | C/T |
| chr2 | 242565241 | 2 | 2 | chr2 | 242565239 | rs34334574 | C/T |
| chr2 | 242643272 | 1 | 3 | chr2 | 242643270 | rs55798363 | C/T |
| chr3 | 480030 | 2 | 6 | chr3 | 480029 | rs1078673 | A/G |
| chr3 | 1471905 | 1 | 3 | chr3 | 1471904 | rs12489560 | A/G |
| chr3 | 1829321 | 1 | 3 | chr3 | 1829319 | rs4234543 | C/T |
| chr3 | 1878274 | 6 | 3 | chr3 | 1878273 | rs12632630 | A/G |
| chr3 | 3247958 | 2 | 2 | chr3 | 3247956 | rs7633036 | A/C |
| chr3 | 3560330 | 1 | 4 | chr3 | 3560328 | rs62247883 | C/T |
| chr3 | 3782630 | 2 | 5 | chr3 | 3782629 | rs1352407 | A/G |
| chr3 | 3817821 | 2 | 10 | chr3 | 3817820 | rs2584051 | C/T |
| chr3 | 4725809 | 5 | 1 | chr3 | 4725807 | rs3804994 | C/T |
| chr3 | 4810033 | 3 | 10 | chr3 | 4810032 | rs7645608 | A/G |
| chr3 | 4811240 | 1 | 4 | chr3 | 4811239 | rs2633715 | A/G |
| chr3 | 5043646 | 2 | 3 | chr3 | 5043644 | rs11130242 | C/T |
| chr3 | 5798044 | 2 | 3 | chr3 | 5798043 | rs2437204 | C/T |
| chr3 | 5940178 | 7 | 2 | chr3 | 5940177 | rs925855 | C/T |
| chr3 | 6370871 | 2 | 2 | chr3 | 6370869 | rs10510332 | C/T |
| chr3 | 7757372 | 2 | 2 | chr3 | 7757370 | rs9826579 | C/T |
| chr3 | 8045407 | 5 | 2 | chr3 | 8045406 | rs547596 | C/T |
| chr3 | 8667449 | 4 | 10 | chr3 | 8667448 | rs370508 | A/G |
| chr3 | 8816763 | 0 | 0 | chr3 | 8816761 | rs237836 | C/T |
| chr3 | 9041854 | 5 | 4 | chr3 | 9041852 | rs421041 | A/G |
| chr3 | 9151074 | 1 | 4 | chr3 | 9151073 | rs2675188 | C/T |
| chr3 | 9169283 | 2 | 3 | chr3 | 9169281 | rs2675185 | A/G |
| chr3 | 9359715 | 1 | 3 | chr3 | 9359713 | rs2600197 | A/G |
| chr3 | 9883525 | 2 | 7 | chr3 | 9883524 | rs2479 | A/G |
| chr3 | 10413326 | 5 | 1 | chr3 | 10413324 | rs17032817 | C/T |
| chr3 | 10949051 | 1 | 3 | chr3 | 10949050 | rs1881367 | A/G |
| chr3 | 11101823 | 1 | 4 | chr3 | 11101823 | rs2343789 | C/G |
| chr3 | 11105167 | 1 | 3 | chr3 | 11105166 | rs349716 | A/G |
| chr3 | 12778163 | 7 | 3 | chr3 | 12778161 | rs9985266 | C/T |

| | | | | | | | |
|---|---|---|---|---|---|---|---|
| chr3 | 13136473 | 2 | 4 | chr3 | 13136471 | rs2648704 | A/G |
| chr3 | 13161109 | 2 | 9 | chr3 | 13161108 | rs358374 | C/T |
| chr3 | 13194338 | 1 | 4 | chr3 | 13194336 | rs357139 | C/T |
| chr3 | 13236850 | 2 | 3 | chr3 | 13236849 | rs12632026 | A/G |
| chr3 | 13275500 | 2 | 2 | chr3 | 13275498 | rs56073575 | C/T |
| chr3 | 13349732 | 4 | 11 | chr3 | 13349731 | rs6769425 | A/G |
| chr3 | 13412276 | 2 | 3 | chr3 | 13412274 | rs6767830 | C/T |
| chr3 | 13567322 | 8 | 2 | chr3 | 13567320 | rs2122499 | A/G |
| chr3 | 13701400 | 1 | 3 | chr3 | 13701399 | rs9812250 | C/G |
| chr3 | 13701400 | 1 | 3 | chr3 | 13701399 | rs35185053 | CA/GG |
| chr3 | 13701400 | 1 | 3 | chr3 | 13701400 | rs9849765 | A/G |
| chr3 | 13889827 | 6 | 6 | chr3 | 13889826 | rs9868948 | G/T |
| chr3 | 13893958 | 2 | 3 | chr3 | 13893956 | rs9832711 | A/C |
| chr3 | 14439436 | 2 | 2 | chr3 | 14439435 | rs12494428 | A/G |
| chr3 | 14451315 | 1 | 7 | chr3 | 14451314 | rs11720548 | G/T |
| chr3 | 14494107 | 1 | 4 | chr3 | 14494105 | rs9845444 | C/T |
| chr3 | 14669082 | 1 | 9 | chr3 | 14669080 | rs6442464 | C/T |
| chr3 | 14864761 | 6 | 2 | chr3 | 14864759 | rs1687295 | C/T |
| chr3 | 14915616 | 1 | 4 | chr3 | 14915614 | rs1687335 | A/G |
| chr3 | 15080909 | 1 | 4 | chr3 | 15080907 | rs62240400 | C/G |
| chr3 | 15470658 | 7 | 1 | chr3 | 15470657 | rs3773458 | C/T |
| chr3 | 15923467 | 9 | 5 | chr3 | 15923465 | rs4685271 | C/T |
| chr3 | 16047075 | 5 | 2 | chr3 | 16047074 | rs1993332 | C/T |
| chr3 | 16167148 | 2 | 3 | chr3 | 16167146 | rs6771632 | C/T |
| chr3 | 16184336 | 5 | 1 | chr3 | 16184335 | rs2730349 | A/G |
| chr3 | 16423595 | 2 | 3 | chr3 | 16423593 | rs4685334 | C/G |
| chr3 | 16448111 | 5 | 1 | chr3 | 16448110 | rs1464170 | A/G |
| chr3 | 19373246 | 1 | 6 | chr3 | 19373244 | rs73182758 | A/C |
| chr3 | 20482625 | 3 | 14 | chr3 | 20482624 | rs6550532 | A/G |
| chr3 | 21349778 | 3 | 2 | chr3 | 21349777 | rs1395996 | A/G |
| chr3 | 21772648 | 2 | 2 | chr3 | 21772646 | rs7616111 | C/G |
| chr3 | 21899195 | 3 | 1 | chr3 | 21899194 | rs259554 | C/G |

| | | | | | | | |
|---|---|---|---|---|---|---|---|
| chr3 | 23254628 | 7 | 2 | chr3 | 23254626 | rs12330479 | C/T |
| chr3 | 24323201 | 1 | 6 | chr3 | 24323200 | rs4295122 | A/G |
| chr3 | 24700507 | 3 | 5 | chr3 | 24700506 | rs62230488 | A/G |
| chr3 | 24822085 | 1 | 3 | chr3 | 24822083 | rs6781264 | C/T |
| chr3 | 25146630 | 2 | 3 | chr3 | 25146628 | rs2062483 | A/C |
| chr3 | 25313966 | 2 | 4 | chr3 | 25313964 | rs17016150 | C/T |
| chr3 | 25397780 | 2 | 2 | chr3 | 25397779 | rs7618590 | A/G |
| chr3 | 25677049 | 2 | 3 | chr3 | 25677048 | rs73054065 | A/G |
| chr3 | 27336885 | 14 | 2 | chr3 | 27336883 | rs583298 | C/T |
| chr3 | 27336885 | 14 | 2 | chr3 | 27336884 | rs537613 | A/G |
| chr3 | 27988908 | 1 | 4 | chr3 | 27988906 | rs9825611 | C/T |
| chr3 | 28056686 | 5 | 1 | chr3 | 28056685 | rs11926203 | A/G |
| chr3 | 28844953 | 3 | 2 | chr3 | 28844951 | rs1353926 | A/G |
| chr3 | 28844953 | 3 | 2 | chr3 | 28844952 | rs1353925 | C/T |
| chr3 | 30131817 | 12 | 5 | chr3 | 30131814 | rs1946640 | G/T |
| chr3 | 30156702 | 4 | 4 | chr3 | 30156701 | rs11708467 | A/G |
| chr3 | 30262463 | 5 | 4 | chr3 | 30262463 | rs9834473 | G/T |
| chr3 | 30278390 | 1 | 3 | chr3 | 30278389 | rs62242244 | A/G |
| chr3 | 32396084 | 3 | 4 | chr3 | 32396082 | rs4955287 | C/T |
| chr3 | 32396084 | 3 | 4 | chr3 | 32396083 | rs28377724 | A/G |
| chr3 | 32483503 | 7 | 27 | chr3 | 32483501 | rs619913 | A/G |
| chr3 | 33074885 | 5 | 2 | chr3 | 33074883 | rs7637435 | C/T |
| chr3 | 35176054 | 6 | 5 | chr3 | 35176052 | rs4340651 | A/C |
| chr3 | 36676511 | 13 | 7 | chr3 | 36676510 | rs1396026 | A/G |
| chr3 | 37574865 | 2 | 3 | chr3 | 37574863 | rs730172 | C/T |
| chr3 | 38381653 | 2 | 5 | chr3 | 38381651 | rs704942 | A/G |
| chr3 | 38533834 | 2 | 2 | chr3 | 38533833 | rs6599211 | A/G |
| chr3 | 38774614 | 1 | 3 | chr3 | 38774612 | rs62244071 | C/T |
| chr3 | 39768887 | 1 | 8 | chr3 | 39768885 | rs4676668 | C/T |
| chr3 | 41265420 | 5 | 6 | chr3 | 41265418 | rs2371485 | C/T |
| chr3 | 41463724 | 2 | 3 | chr3 | 41463721 | rs62259335 | C/T |
| chr3 | 41514644 | 1 | 8 | chr3 | 41514643 | rs1795326 | C/T |

| | | | | | | | |
|---|---|---|---|---|---|---|---|
| chr3 | 41523546 | 1 | 4 | chr3 | 41523544 | rs2055187 | C/T |
| chr3 | 42751606 | 1 | 5 | chr3 | 42751604 | rs121993 | A/G |
| chr3 | 43004611 | 1 | 5 | chr3 | 43004610 | rs834180 | C/T |
| chr3 | 43079707 | 6 | 4 | chr3 | 43079705 | rs834176 | G/T |
| chr3 | 43103093 | 15 | 3 | chr3 | 43103091 | rs674118 | G/T |
| chr3 | 44303113 | 2 | 2 | chr3 | 44303112 | rs6801972 | A/G |
| chr3 | 45163761 | 11 | 3 | chr3 | 45163760 | rs13066191 | G/T |
| chr3 | 45574423 | 3 | 2 | chr3 | 45574422 | rs4575933 | C/G |
| chr3 | 45574423 | 3 | 2 | chr3 | 45574423 | rs34945992 | A/G |
| chr3 | 45715563 | 1 | 6 | chr3 | 45715561 | rs9829631 | C/T |
| chr3 | 46684504 | 4 | 4 | chr3 | 46684503 | rs939411 | A/G |
| chr3 | 46692936 | 11 | 3 | chr3 | 46692934 | rs6808525 | C/T |
| chr3 | 46710102 | 7 | 2 | chr3 | 46710101 | rs11719061 | C/G |
| chr3 | 46767143 | 4 | 2 | chr3 | 46767141 | rs28584393 | C/T |
| chr3 | 46918033 | 1 | 3 | chr3 | 46918031 | rs1531137 | A/G |
| chr3 | 46943232 | 5 | 3 | chr3 | 46943230 | rs7635480 | C/T |
| chr3 | 47312706 | 1 | 5 | chr3 | 47312704 | rs295443 | C/T |
| chr3 | 47424585 | 4 | 2 | chr3 | 47424583 | rs1531874 | C/T |
| chr3 | 47493494 | 3 | 3 | chr3 | 47493493 | rs12631697 | A/G |
| chr3 | 48464402 | 2 | 5 | chr3 | 48464401 | rs922075 | A/G |
| chr3 | 49728007 | 2 | 9 | chr3 | 49728006 | rs34614773 | G/T |
| chr3 | 52228010 | 1 | 5 | chr3 | 52228008 | rs352162 | C/T |
| chr3 | 52561030 | 2 | 7 | chr3 | 52561029 | rs1961958 | C/T |
| chr3 | 52777443 | 1 | 6 | chr3 | 52777441 | rs2230534 | G/T |
| chr3 | 52812896 | 2 | 2 | chr3 | 52812894 | rs2710331 | A/G |
| chr3 | 53055189 | 2 | 5 | chr3 | 53055187 | rs2564941 | A/C |
| chr3 | 53059329 | 9 | 2 | chr3 | 53059328 | rs2581814 | A/C |
| chr3 | 53174874 | 1 | 3 | chr3 | 53174872 | rs6445567 | C/T |
| chr3 | 53534947 | 4 | 3 | chr3 | 53534946 | rs62251864 | A/G |
| chr3 | 53644945 | 2 | 7 | chr3 | 53644943 | rs11709171 | A/C |
| chr3 | 53748478 | 5 | 1 | chr3 | 53748476 | rs2612030 | A/G |
| chr3 | 53759543 | 2 | 2 | chr3 | 53759542 | rs877257 | C/T |

| | | | | | | | |
|---|---|---|---|---|---|---|---|
| chr3 | 54350396 | 0 | 0 | chr3 | 54350395 | rs62256098 | A/G |
| chr3 | 54697189 | 4 | 2 | chr3 | 54697187 | rs17054291 | C/T |
| chr3 | 55099522 | 0 | 0 | chr3 | 55099520 | rs3868873 | C/T |
| chr3 | 55282443 | 6 | 1 | chr3 | 55282441 | rs465970 | C/T |
| chr3 | 56851637 | 3 | 9 | chr3 | 56851635 | rs1500708 | A/G |
| chr3 | 57471129 | 2 | 2 | chr3 | 57471127 | rs9869463 | C/T |
| chr3 | 57740685 | 3 | 1 | chr3 | 57740683 | rs9811191 | C/T |
| chr3 | 57804407 | 3 | 2 | chr3 | 57804405 | rs17666667 | C/T |
| chr3 | 57941486 | 5 | 2 | chr3 | 57941484 | rs1658371 | C/T |
| chr3 | 58147605 | 25 | 7 | chr3 | 58147604 | rs34899072 | A/G |
| chr3 | 58346786 | 3 | 2 | chr3 | 58346784 | rs62258093 | C/T |
| chr3 | 58590711 | 3 | 1 | chr3 | 58590709 | rs11130660 | C/T |
| chr3 | 58786097 | 2 | 2 | chr3 | 58786096 | rs6782432 | A/G |
| chr3 | 59214182 | 3 | 1 | chr3 | 59214181 | rs6782094 | C/G |
| chr3 | 59378688 | 3 | 2 | chr3 | 59378686 | rs7623549 | C/T |
| chr3 | 59786677 | 1 | 4 | chr3 | 59786676 | rs1872495 | A/G |
| chr3 | 60572879 | 2 | 2 | chr3 | 60572878 | rs59194055 | G/T |
| chr3 | 60605839 | 5 | 3 | chr3 | 60605838 | rs2856068 | A/G |
| chr3 | 60729248 | 4 | 2 | chr3 | 60729245 | rs57203484 | A/C |
| chr3 | 61524311 | 3 | 2 | chr3 | 61524311 | rs9827952 | G/T |
| chr3 | 62642143 | 2 | 5 | chr3 | 62642142 | rs62242518 | G/T |
| chr3 | 62667321 | 3 | 3 | chr3 | 62667319 | rs4688319 | C/T |
| chr3 | 62769603 | 4 | 2 | chr3 | 62769601 | rs304223 | C/T |
| chr3 | 62909432 | 2 | 5 | chr3 | 62909431 | rs4688344 | A/G |
| chr3 | 63004556 | 8 | 2 | chr3 | 63004555 | rs11720948 | A/G |
| chr3 | 63545699 | 3 | 2 | chr3 | 63545698 | rs6809554 | A/G |
| chr3 | 64751508 | 1 | 8 | chr3 | 64751507 | rs318767 | A/G |
| chr3 | 65350832 | 10 | 3 | chr3 | 65350831 | rs9862463 | A/G |
| chr3 | 65399823 | 5 | 1 | chr3 | 65399822 | rs9880755 | A/G |
| chr3 | 66747553 | 3 | 1 | chr3 | 66747550 | rs2365473 | A/G |
| chr3 | 67131454 | 4 | 3 | chr3 | 67131451 | rs4856833 | C/T |
| chr3 | 67469520 | 3 | 1 | chr3 | 67469519 | rs1387865 | A/G |

| | | | | | | | |
|---|---|---|---|---|---|---|---|
| chr3 | 67485532 | 2 | 2 | chr3 | 67485530 | rs9828283 | C/T |
| chr3 | 67507714 | 1 | 3 | chr3 | 67507712 | rs4615095 | C/T |
| chr3 | 67852263 | 1 | 3 | chr3 | 67852262 | rs4856882 | A/G |
| chr3 | 69123292 | 3 | 1 | chr3 | 69123290 | rs7644667 | C/T |
| chr3 | 69520271 | 10 | 1 | chr3 | 69520269 | rs986203 | A/G |
| chr3 | 69958105 | 5 | 2 | chr3 | 69958104 | rs9872200 | G/T |
| chr3 | 70486630 | 1 | 6 | chr3 | 70486627 | rs805490 | C/T |
| chr3 | 71454425 | 8 | 2 | chr3 | 71454423 | rs4677035 | A/C |
| chr3 | 71708814 | 3 | 2 | chr3 | 71708812 | rs9828619 | C/T |
| chr3 | 71724895 | 3 | 2 | chr3 | 71724894 | rs7618503 | G/T |
| chr3 | 72078127 | 3 | 1 | chr3 | 72078125 | rs11128229 | C/T |
| chr3 | 73820568 | 3 | 6 | chr3 | 73820567 | rs1567576 | A/G |
| chr3 | 75361696 | 7 | 3 | chr3 | 75361695 | rs72897080 | A/G |
| chr3 | 75801419 | 6 | 1 | chr3 | 75801418 | rs36165888 | C/T |
| chr3 | 75819930 | 2 | 4 | chr3 | 75819928 | rs4071885 | C/G |
| chr3 | 75848751 | 4 | 1 | chr3 | 75848750 | rs7530959 | A/G |
| chr3 | 76198745 | 4 | 5 | chr3 | 76198743 | rs4624567 | C/T |
| chr3 | 76895518 | 1 | 5 | chr3 | 76895516 | rs2608160 | C/T |
| chr3 | 77480761 | 4 | 1 | chr3 | 77480759 | rs6548500 | C/T |
| chr3 | 77649263 | 3 | 1 | chr3 | 77649262 | rs9839344 | A/G |
| chr3 | 78783861 | 2 | 11 | chr3 | 78783859 | rs60505291 | C/T |
| chr3 | 79525741 | 9 | 1 | chr3 | 79525740 | rs17396958 | A/G |
| chr3 | 81963947 | 4 | 3 | chr3 | 81963945 | rs2691073 | G/T |
| chr3 | 82627186 | 4 | 1 | chr3 | 82627184 | rs6548820 | C/T |
| chr3 | 84421936 | 3 | 2 | chr3 | 84421935 | rs4536842 | C/T |
| chr3 | 87487115 | 3 | 2 | chr3 | 87487114 | rs4859062 | A/G |
| chr3 | 88258290 | 5 | 2 | chr3 | 88258288 | rs4858933 | C/T |
| chr3 | 96661336 | 2 | 11 | chr3 | 96661335 | rs4340704 | A/G |
| chr3 | 97523022 | 4 | 2 | chr3 | 97523021 | rs58471761 | A/G |
| chr3 | 99983818 | 2 | 2 | chr3 | 99983817 | rs3772101 | A/G |
| chr3 | 100042030 | 8 | 5 | chr3 | 100042028 | rs2439227 | C/G |
| chr3 | 101237136 | 2 | 3 | chr3 | 101237135 | rs7432913 | A/G |

| | | | | | | | |
|---|---|---|---|---|---|---|---|
| chr3 | 101250244 | 1 | 3 | chr3 | 101250242 | rs4928151 | A/C |
| chr3 | 102768110 | 8 | 6 | chr3 | 102768109 | rs1976944 | A/G |
| chr3 | 104947505 | 1 | 4 | chr3 | 104947503 | rs2100653 | A/G |
| chr3 | 106031647 | 2 | 11 | chr3 | 106031646 | rs1946723 | A/G |
| chr3 | 107178390 | 2 | 5 | chr3 | 107178389 | rs2655032 | C/T |
| chr3 | 107178476 | 3 | 1 | chr3 | 107178474 | rs4894970 | C/T |
| chr3 | 107555879 | 1 | 3 | chr3 | 107555877 | rs4481183 | C/T |
| chr3 | 108004252 | 4 | 8 | chr3 | 108004250 | rs1607795 | C/T |
| chr3 | 108090009 | 6 | 3 | chr3 | 108090007 | rs812830 | A/C |
| chr3 | 108326582 | 4 | 1 | chr3 | 108326581 | rs1528307 | C/T |
| chr3 | 108862527 | 5 | 4 | chr3 | 108862526 | rs167924 | A/G |
| chr3 | 109622838 | 1 | 5 | chr3 | 109622837 | rs4593038 | A/G |
| chr3 | 109740407 | 2 | 5 | chr3 | 109740406 | rs2603130 | C/G |
| chr3 | 109981389 | 2 | 2 | chr3 | 109981388 | rs16844883 | G/T |
| chr3 | 110216778 | 6 | 1 | chr3 | 110216777 | rs6437813 | A/G |
| chr3 | 110344094 | 2 | 4 | chr3 | 110344093 | rs10049362 | A/G |
| chr3 | 110471968 | 1 | 6 | chr3 | 110471966 | rs4855586 | A/C |
| chr3 | 110520368 | 2 | 6 | chr3 | 110520367 | rs7648265 | A/G |
| chr3 | 110916031 | 5 | 3 | chr3 | 110916031 | rs1733268 | A/G |
| chr3 | 111469789 | 1 | 4 | chr3 | 111469787 | rs6790010 | C/T |
| chr3 | 112081509 | 3 | 6 | chr3 | 112081507 | rs2169565 | C/T |
| chr3 | 112963368 | 4 | 1 | chr3 | 112963366 | rs6791845 | C/T |
| chr3 | 114192518 | 5 | 4 | chr3 | 114192517 | rs2272393 | C/G |
| chr3 | 115580196 | 5 | 2 | chr3 | 115580194 | rs2722006 | A/G |
| chr3 | 115595358 | 1 | 4 | chr3 | 115595357 | rs2718432 | C/T |
| chr3 | 116619010 | 10 | 1 | chr3 | 116619009 | rs6773235 | A/G |
| chr3 | 117230863 | 8 | 1 | chr3 | 117230861 | rs9863087 | C/T |
| chr3 | 118067972 | 4 | 4 | chr3 | 118067970 | rs7643014 | C/T |
| chr3 | 120857891 | 3 | 1 | chr3 | 120857891 | rs1872432 | C/T |
| chr3 | 121604101 | 2 | 2 | chr3 | 121604100 | rs1147699 | C/T |
| chr3 | 123690984 | 1 | 6 | chr3 | 123690982 | rs10934601 | C/T |
| chr3 | 123996655 | 8 | 7 | chr3 | 123996653 | rs891406 | G/T |

| | | | | | | | |
|---|---|---|---|---|---|---|---|
| chr3 | 124113038 | 2 | 3 | chr3 | 124113035 | rs2303983 | C/T |
| chr3 | 124148738 | 3 | 3 | chr3 | 124148737 | rs9831044 | A/G |
| chr3 | 124347509 | 1 | 5 | chr3 | 124347507 | rs1567991 | A/G |
| chr3 | 124572618 | 13 | 1 | chr3 | 124572617 | rs6438790 | A/G |
| chr3 | 124627569 | 5 | 1 | chr3 | 124627568 | rs1869861 | C/T |
| chr3 | 124650269 | 7 | 5 | chr3 | 124650268 | rs62262433 | C/G |
| chr3 | 125933975 | 1 | 3 | chr3 | 125933973 | rs16835925 | C/T |
| chr3 | 126060362 | 3 | 3 | chr3 | 126060360 | rs848797 | C/T |
| chr3 | 126363067 | 2 | 11 | chr3 | 126363066 | rs567432 | A/G |
| chr3 | 126557990 | 2 | 4 | chr3 | 126557989 | rs7609964 | A/G |
| chr3 | 126803821 | 12 | 1 | chr3 | 126803819 | rs11717632 | C/G |
| chr3 | 127339605 | 3 | 2 | chr3 | 127339605 | rs6799991 | A/G |
| chr3 | 127348716 | 1 | 4 | chr3 | 127348715 | rs1965848 | C/T |
| chr3 | 127639350 | 1 | 6 | chr3 | 127639349 | rs7131 | C/G |
| chr3 | 128116109 | 8 | 7 | chr3 | 128116108 | rs2303994 | C/G |
| chr3 | 128624532 | 1 | 5 | chr3 | 128624529 | rs34887230 | C/G |
| chr3 | 128665788 | 2 | 4 | chr3 | 128665787 | rs4974437 | A/G |
| chr3 | 128915249 | 3 | 6 | chr3 | 128915248 | rs597956 | C/T |
| chr3 | 128924504 | 1 | 4 | chr3 | 128924502 | rs7652988 | C/T |
| chr3 | 128952086 | 4 | 3 | chr3 | 128952084 | rs497897 | C/T |
| chr3 | 128956215 | 1 | 3 | chr3 | 128956213 | rs6803366 | C/G |
| chr3 | 129484058 | 3 | 1 | chr3 | 129484056 | rs7374952 | C/T |
| chr3 | 129631054 | 2 | 6 | chr3 | 129631052 | rs7630605 | C/G |
| chr3 | 129639982 | 3 | 4 | chr3 | 129639981 | rs2713583 | G/T |
| chr3 | 129646013 | 1 | 4 | chr3 | 129646012 | rs7622080 | A/G |
| chr3 | 130271468 | 3 | 2 | chr3 | 130271467 | rs9850562 | A/G |
| chr3 | 130794703 | 4 | 2 | chr3 | 130794701 | rs2626000 | C/T |
| chr3 | 131029420 | 7 | 2 | chr3 | 131029418 | rs784689 | C/T |
| chr3 | 131716761 | 14 | 12 | chr3 | 131716760 | rs2661949 | C/T |
| chr3 | 133206721 | 1 | 3 | chr3 | 133206720 | rs9839165 | A/G |
| chr3 | 134165585 | 5 | 1 | chr3 | 134165583 | rs7614291 | C/T |
| chr3 | 134562860 | 2 | 4 | chr3 | 134562859 | rs11919147 | A/G |

| | | | | | | | |
|---|---|---|---|---|---|---|---|
| chr3 | 134629965 | 3 | 3 | chr3 | 134629964 | rs1871357 | A/G |
| chr3 | 135186522 | 3 | 1 | chr3 | 135186522 | rs11709321 | G/T |
| chr3 | 135654066 | 5 | 1 | chr3 | 135654065 | rs1391695 | A/C |
| chr3 | 137225057 | 10 | 6 | chr3 | 137225056 | rs1523594 | A/G |
| chr3 | 139270652 | 3 | 5 | chr3 | 139270652 | rs445025 | A/C |
| chr3 | 140308231 | 0 | 0 | chr3 | 140308230 | rs676734 | C/T |
| chr3 | 140963860 | 1 | 4 | chr3 | 140963859 | rs9847705 | G/T |
| chr3 | 141327008 | 3 | 1 | chr3 | 141327007 | rs4308236 | A/G |
| chr3 | 141477561 | 1 | 8 | chr3 | 141477560 | rs4234485 | G/T |
| chr3 | 141526330 | 4 | 1 | chr3 | 141526329 | rs1479875 | A/G |
| chr3 | 141767806 | 3 | 2 | chr3 | 141767804 | rs4683510 | C/T |
| chr3 | 141931030 | 3 | 1 | chr3 | 141931028 | rs869393 | G/T |
| chr3 | 142041712 | 4 | 1 | chr3 | 142041711 | rs2030295 | A/G |
| chr3 | 142053411 | 5 | 2 | chr3 | 142053410 | rs9814403 | A/G |
| chr3 | 142857689 | 1 | 5 | chr3 | 142857688 | rs295298 | A/G |
| chr3 | 143083988 | 1 | 3 | chr3 | 143083986 | rs4234470 | C/T |
| chr3 | 144360966 | 6 | 1 | chr3 | 144360964 | rs4564940 | C/T |
| chr3 | 146051289 | 4 | 3 | chr3 | 146051287 | rs10935517 | C/T |
| chr3 | 146213251 | 1 | 4 | chr3 | 146213250 | rs9289690 | A/G |
| chr3 | 148107204 | 2 | 2 | chr3 | 148107202 | rs7649578 | A/C |
| chr3 | 149899732 | 2 | 2 | chr3 | 149899731 | rs2640539 | C/G |
| chr3 | 150096076 | 3 | 4 | chr3 | 150096075 | rs957790 | A/G |
| chr3 | 150490041 | 1 | 8 | chr3 | 150490040 | rs62276205 | A/G |
| chr3 | 150553602 | 2 | 2 | chr3 | 150553600 | rs6440599 | C/T |
| chr3 | 150710708 | 4 | 1 | chr3 | 150710706 | rs13072859 | C/T |
| chr3 | 153336805 | 2 | 2 | chr3 | 153336804 | rs188981 | A/G |
| chr3 | 153342055 | 3 | 1 | chr3 | 153342054 | rs7629219 | A/G |
| chr3 | 153804779 | 2 | 2 | chr3 | 153804777 | rs11927708 | C/T |
| chr3 | 153883646 | 1 | 3 | chr3 | 153883644 | rs1949384 | C/T |
| chr3 | 154157612 | 3 | 1 | chr3 | 154157610 | rs1403501 | A/C |
| chr3 | 154514229 | 1 | 4 | chr3 | 154514228 | rs6785362 | A/G |
| chr3 | 154859663 | 3 | 1 | chr3 | 154859662 | rs17738623 | A/G |

| | | | | | | | |
|---|---|---|---|---|---|---|---|
| chr3 | 155091665 | 11 | 2 | chr3 | 155091664 | rs787954 | A/G |
| chr3 | 158570737 | 1 | 3 | chr3 | 158570735 | rs1500916 | C/G |
| chr3 | 158941619 | 5 | 2 | chr3 | 158941618 | rs4287882 | A/G |
| chr3 | 159307820 | 5 | 1 | chr3 | 159307819 | rs4234321 | A/G |
| chr3 | 160884874 | 2 | 9 | chr3 | 160884873 | rs9845025 | A/G |
| chr3 | 161061236 | 1 | 3 | chr3 | 161061235 | rs16847223 | A/G |
| chr3 | 162919782 | 1 | 3 | chr3 | 162919780 | rs1515732 | A/G |
| chr3 | 163141680 | 2 | 2 | chr3 | 163141679 | rs6797849 | G/T |
| chr3 | 163965426 | 1 | 4 | chr3 | 163965425 | rs9290133 | A/G |
| chr3 | 164137289 | 4 | 1 | chr3 | 164137287 | rs9283620 | C/T |
| chr3 | 165225363 | 2 | 2 | chr3 | 165225361 | rs11714940 | C/T |
| chr3 | 166395414 | 1 | 3 | chr3 | 166395413 | rs6443475 | A/G |
| chr3 | 166949730 | 1 | 3 | chr3 | 166949730 | rs10513616 | A/G |
| chr3 | 166972156 | 6 | 2 | chr3 | 166972155 | rs2686402 | A/G |
| chr3 | 167624258 | 2 | 2 | chr3 | 167624255 | rs6443551 | C/T |
| chr3 | 167624258 | 2 | 2 | chr3 | 167624256 | rs10936491 | C/T |
| chr3 | 169228397 | 2 | 7 | chr3 | 169228396 | rs9861339 | A/G |
| chr3 | 170051663 | 6 | 1 | chr3 | 170051662 | rs55825701 | A/G |
| chr3 | 170588731 | 1 | 5 | chr3 | 170588729 | rs1273879 | C/T |
| chr3 | 170805020 | 3 | 2 | chr3 | 170805018 | rs9814415 | C/T |
| chr3 | 171257078 | 3 | 4 | chr3 | 171257076 | rs16854812 | C/T |
| chr3 | 171422741 | 5 | 5 | chr3 | 171422738 | rs481781 | C/T |
| chr3 | 171580536 | 3 | 1 | chr3 | 171580535 | rs16855034 | C/G |
| chr3 | 171684335 | 2 | 4 | chr3 | 171684333 | rs6789637 | C/T |
| chr3 | 172796406 | 2 | 2 | chr3 | 172796404 | rs58549358 | C/T |
| chr3 | 173308777 | 3 | 2 | chr3 | 173308776 | rs6768163 | G/T |
| chr3 | 173645532 | 1 | 19 | chr3 | 173645530 | rs477251 | C/T |
| chr3 | 174799303 | 3 | 1 | chr3 | 174799302 | rs3853387 | A/C |
| chr3 | 175622294 | 2 | 6 | chr3 | 175622292 | rs11717847 | C/T |
| chr3 | 176289634 | 3 | 1 | chr3 | 176289633 | rs9880351 | G/T |
| chr3 | 177750383 | 4 | 7 | chr3 | 177750382 | rs62285666 | A/G |
| chr3 | 178939346 | 3 | 1 | chr3 | 178939344 | rs6443490 | C/T |

| | | | | | | | |
|---|---|---|---|---|---|---|---|
| chr3 | 178944761 | 3 | 1 | chr3 | 178944760 | rs9852700 | A/G |
| chr3 | 178947323 | 3 | 1 | chr3 | 178947322 | rs9864169 | A/G |
| chr3 | 180901870 | 2 | 2 | chr3 | 180901868 | rs9833074 | C/T |
| chr3 | 181174392 | 1 | 3 | chr3 | 181174390 | rs12488363 | C/T |
| chr3 | 181241044 | 4 | 2 | chr3 | 181241042 | rs56397887 | C/T |
| chr3 | 183956444 | 7 | 3 | chr3 | 183956443 | rs2700852 | A/G |
| chr3 | 183979016 | 10 | 1 | chr3 | 183979015 | rs9290746 | A/G |
| chr3 | 184138553 | 2 | 5 | chr3 | 184138551 | rs1002767 | A/G |
| chr3 | 184331810 | 2 | 2 | chr3 | 184331808 | rs7630494 | C/T |
| chr3 | 185492280 | 2 | 2 | chr3 | 185492279 | rs35637422 | A/G |
| chr3 | 185709134 | 2 | 10 | chr3 | 185709132 | rs4912496 | C/T |
| chr3 | 185710213 | 3 | 1 | chr3 | 185710211 | rs62286032 | A/C |
| chr3 | 185951327 | 1 | 26 | chr3 | 185951325 | rs4476482 | C/T |
| chr3 | 186353857 | 3 | 2 | chr3 | 186353855 | rs7640955 | C/T |
| chr3 | 186531009 | 1 | 12 | chr3 | 186531007 | rs7432658 | C/T |
| chr3 | 186614558 | 7 | 1 | chr3 | 186614556 | rs9837797 | C/T |
| chr3 | 186661257 | 3 | 5 | chr3 | 186661255 | rs57425527 | C/T |
| chr3 | 187449294 | 2 | 4 | chr3 | 187449293 | rs2268838 | C/T |
| chr3 | 188211848 | 14 | 2 | chr3 | 188211847 | rs4686834 | A/G |
| chr3 | 188214443 | 0 | 0 | chr3 | 188214442 | rs7619989 | C/G |
| chr3 | 188303894 | 16 | 3 | chr3 | 188303892 | rs849755 | A/G |
| chr3 | 188409041 | 2 | 3 | chr3 | 188409040 | rs61690667 | A/G |
| chr3 | 188691367 | 3 | 1 | chr3 | 188691365 | rs1579367 | C/T |
| chr3 | 188814184 | 2 | 2 | chr3 | 188814182 | rs6444245 | C/T |
| chr3 | 188935604 | 2 | 5 | chr3 | 188935603 | rs9869180 | A/G |
| chr3 | 189075594 | 2 | 5 | chr3 | 189075592 | rs2713722 | C/T |
| chr3 | 189676022 | 3 | 3 | chr3 | 189676020 | rs1346161 | G/T |
| chr3 | 189955016 | 5 | 3 | chr3 | 189955015 | rs34631447 | A/G |
| chr3 | 190755615 | 3 | 1 | chr3 | 190755613 | rs1920260 | C/T |
| chr3 | 190818538 | 4 | 2 | chr3 | 190818537 | rs36108040 | A/G |
| chr3 | 190830471 | 3 | 6 | chr3 | 190830469 | rs2178413 | C/T |
| chr3 | 190980310 | 1 | 4 | chr3 | 190980309 | rs4686529 | A/G |

| | | | | | | | | |
|---|---|---|---|---|---|---|---|---|
| chr3 | 191429462 | 2 | 7 | chr3 | 191429460 | rs512265 | C/T |
| chr3 | 191516277 | 2 | 3 | chr3 | 191516276 | rs9809943 | A/G |
| chr3 | 194529279 | 2 | 2 | chr3 | 194529278 | rs12639145 | A/G |
| chr3 | 195188377 | 3 | 1 | chr3 | 195188376 | rs1388102 | C/T |
| chr3 | 195269425 | 3 | 1 | chr3 | 195269424 | rs73201434 | A/G |
| chr3 | 195472418 | 3 | 1 | chr3 | 195472418 | rs60219871 | A/G |
| chr3 | 195541748 | 1 | 7 | chr3 | 195541748 | rs6772300 | C/G |
| chr3 | 195819191 | 1 | 3 | chr3 | 195819189 | rs922282 | A/G |
| chr3 | 195888258 | 3 | 1 | chr3 | 195888255 | rs1773220 | G/GC/T/TT |
| chr3 | 195888258 | 3 | 1 | chr3 | 195888255 | rs63360263 | G/T |
| chr3 | 195981646 | 2 | 4 | chr3 | 195981645 | rs6437415 | A/G |
| chr3 | 195981716 | 7 | 2 | chr3 | 195981715 | rs6437417 | A/G |
| chr3 | 196221444 | 2 | 2 | chr3 | 196221443 | rs2676923 | A/G |
| chr3 | 196233047 | 1 | 6 | chr3 | 196233045 | rs34111367 | -/C |
| chr3 | 196233047 | 1 | 6 | chr3 | 196233047 | rs2676875 | A/G |
| chr3 | 196281253 | 2 | 3 | chr3 | 196281251 | rs10933695 | C/G |
| chr3 | 196795192 | 1 | 3 | chr3 | 196795190 | rs34625309 | C/T |
| chr3 | 196803213 | 1 | 3 | chr3 | 196803211 | rs9330981 | C/T |
| chr3 | 196910859 | 3 | 4 | chr3 | 196910857 | rs3107776 | A/G |
| chr3 | 196935714 | 5 | 12 | chr3 | 196935713 | rs28517569 | A/G |
| chr3 | 196935714 | 5 | 12 | chr3 | 196935713 | rs72611174 | A/G |
| chr3 | 197231795 | 1 | 4 | chr3 | 197231794 | rs7625570 | A/G |
| chr3 | 197538836 | 9 | 3 | chr3 | 197538835 | rs6785339 | G/T |
| chr3 | 197740105 | 1 | 6 | chr3 | 197740105 | rs58400165 | C/G |
| chr3 | 198699160 | 5 | 8 | chr3 | 198699159 | rs7623973 | A/G |
| chr4 | 732573 | 7 | 6 | chr4 | 732571 | rs4690301 | C/T |
| chr4 | 1088222 | 4 | 2 | chr4 | 1088221 | rs10032349 | A/G |
| chr4 | 1097479 | 3 | 3 | chr4 | 1097477 | rs62296477 | C/T |
| chr4 | 1182243 | 3 | 1 | chr4 | 1182242 | rs1250125 | C/T |
| chr4 | 1381641 | 0 | 0 | chr4 | 1381639 | rs72643406 | A/C |
| chr4 | 1468880 | 2 | 7 | chr4 | 1468878 | rs11941361 | C/T |
| chr4 | 1495802 | 0 | 0 | chr4 | 1495800 | rs12505155 | C/T |

| | | | | | | | |
|---|---|---|---|---|---|---|---|
| chr4 | 1496825 | 0 | 0 | chr4 | 1496823 | rs34268770 | C/T |
| chr4 | 1496918 | 0 | 0 | chr4 | 1496916 | rs34571779 | C/T |
| chr4 | 1519271 | 1 | 7 | chr4 | 1519268 | rs60607502 | C/G |
| chr4 | 1575848 | 0 | 0 | chr4 | 1575846 | rs6844513 | C/T |
| chr4 | 1580832 | 1 | 7 | chr4 | 1580830 | rs6858080 | C/T |
| chr4 | 1616758 | 5 | 1 | chr4 | 1616754 | rs33913493 | -/CCCA |
| chr4 | 1616758 | 5 | 1 | chr4 | 1616757 | rs56172344 | A/G |
| chr4 | 1690622 | 5 | 7 | chr4 | 1690621 | rs798755 | A/G |
| chr4 | 1723222 | 4 | 2 | chr4 | 1723221 | rs11724531 | A/G |
| chr4 | 1785682 | 4 | 4 | chr4 | 1785681 | rs4242021 | A/G |
| chr4 | 2018369 | 6 | 4 | chr4 | 2018367 | rs518984 | C/G |
| chr4 | 2035171 | 2 | 3 | chr4 | 2035170 | rs454226 | A/C |
| chr4 | 2275850 | 2 | 16 | chr4 | 2275850 | rs661301 | C/T |
| chr4 | 2304497 | 3 | 8 | chr4 | 2304495 | rs28562182 | C/T |
| chr4 | 2361430 | 2 | 4 | chr4 | 2361429 | rs3135089 | C/T |
| chr4 | 2911872 | 1 | 3 | chr4 | 2911870 | rs1475974 | A/G |
| chr4 | 2941296 | 6 | 9 | chr4 | 2941294 | rs2185886 | C/T |
| chr4 | 3449935 | 9 | 3 | chr4 | 3449933 | rs2699429 | C/T |
| chr4 | 3458789 | 1 | 3 | chr4 | 3458789 | rs1881801 | C/T |
| chr4 | 3476731 | 3 | 4 | chr4 | 3476730 | rs2858027 | A/G |
| chr4 | 3488246 | 3 | 4 | chr4 | 3488244 | rs1730778 | A/G |
| chr4 | 3553118 | 1 | 5 | chr4 | 3553117 | rs12508663 | A/G |
| chr4 | 3553289 | 1 | 3 | chr4 | 3553288 | rs2880808 | G/T |
| chr4 | 3572132 | 3 | 5 | chr4 | 3572131 | rs13130523 | A/G |
| chr4 | 3579905 | 3 | 2 | chr4 | 3579904 | rs73792541 | A/G |
| chr4 | 3604393 | 2 | 5 | chr4 | 3604392 | rs12511586 | A/G |
| chr4 | 3604691 | 2 | 4 | chr4 | 3604690 | rs73794094 | A/G |
| chr4 | 3621656 | 5 | 2 | chr4 | 3621654 | rs177787 | A/G |
| chr4 | 3772763 | 3 | 5 | chr4 | 3772761 | rs59854817 | C/T |
| chr4 | 3783728 | 4 | 2 | chr4 | 3783726 | rs28612860 | C/T |
| chr4 | 3828368 | 3 | 1 | chr4 | 3828366 | rs28461257 | C/T |
| chr4 | 3841031 | 1 | 4 | chr4 | 3841030 | rs28544923 | A/G |

| | | | | | | | |
|---|---|---|---|---|---|---|---|
| chr4 | 4988663 | 1 | 5 | chr4 | 4988662 | rs6446716 | A/G |
| chr4 | 5066316 | 6 | 5 | chr4 | 5066315 | rs4546187 | A/G |
| chr4 | 5179061 | 3 | 1 | chr4 | 5179059 | rs10937625 | C/T |
| chr4 | 5567279 | 4 | 5 | chr4 | 5567278 | rs4586871 | A/G |
| chr4 | 5992149 | 7 | 1 | chr4 | 5992148 | rs28690642 | A/G |
| chr4 | 6076667 | 1 | 3 | chr4 | 6076666 | rs9884435 | A/G |
| chr4 | 6344739 | 1 | 3 | chr4 | 6344738 | rs11729672 | A/G |
| chr4 | 6516276 | 12 | 1 | chr4 | 6516274 | rs4540118 | C/T |
| chr4 | 6583239 | 5 | 1 | chr4 | 6583237 | rs4234756 | A/G |
| chr4 | 6583712 | 1 | 4 | chr4 | 6583711 | rs10937740 | A/G |
| chr4 | 6787137 | 2 | 3 | chr4 | 6787136 | rs12646189 | A/G |
| chr4 | 6798944 | 3 | 5 | chr4 | 6798943 | rs9683440 | A/G |
| chr4 | 6799011 | 3 | 1 | chr4 | 6799009 | rs10804986 | C/G |
| chr4 | 7065791 | 5 | 3 | chr4 | 7065789 | rs4689574 | C/T |
| chr4 | 7323090 | 4 | 7 | chr4 | 7323088 | rs6856530 | C/T |
| chr4 | 7356536 | 1 | 5 | chr4 | 7356534 | rs4025901 | A/C/G |
| chr4 | 7356536 | 1 | 5 | chr4 | 7356535 | rs55816146 | G/T |
| chr4 | 7541777 | 2 | 2 | chr4 | 7541775 | rs10805000 | C/T |
| chr4 | 7587578 | 5 | 6 | chr4 | 7587578 | rs6446608 | A/G |
| chr4 | 7741857 | 2 | 4 | chr4 | 7741856 | rs929265 | C/T |
| chr4 | 7757746 | 2 | 2 | chr4 | 7757744 | rs6446614 | C/T |
| chr4 | 7757746 | 2 | 2 | chr4 | 7757746 | rs6827478 | A/G |
| chr4 | 7763707 | 4 | 4 | chr4 | 7763705 | rs757243 | A/G |
| chr4 | 7808950 | 3 | 1 | chr4 | 7808949 | rs17466895 | A/G |
| chr4 | 7814661 | 4 | 1 | chr4 | 7814660 | rs2269850 | C/T |
| chr4 | 7873087 | 2 | 7 | chr4 | 7873085 | rs4234831 | C/T |
| chr4 | 8279632 | 2 | 4 | chr4 | 8279630 | rs1281145 | C/T |
| chr4 | 8279849 | 2 | 5 | chr4 | 8279847 | rs55642964 | C/T |
| chr4 | 8303932 | 10 | 11 | chr4 | 8303930 | rs1712295 | A/G |
| chr4 | 8344455 | 2 | 4 | chr4 | 8344455 | rs13129283 | C/G |
| chr4 | 8369101 | 1 | 4 | chr4 | 8369098 | rs4696809 | A/C |
| chr4 | 8379932 | 2 | 3 | chr4 | 8379931 | rs1474314 | C/T |

| | | | | | | | |
|---|---|---|---|---|---|---|---|
| chr4 | 8425260 | 4 | 1 | chr4 | 8425258 | rs827011 | C/T |
| chr4 | 8669761 | 3 | 5 | chr4 | 8669761 | rs4696867 | C/G |
| chr4 | 8706276 | 3 | 3 | chr4 | 8706274 | rs10010630 | C/T |
| chr4 | 8756660 | 7 | 1 | chr4 | 8756658 | rs10938737 | A/C |
| chr4 | 8756792 | 1 | 3 | chr4 | 8756790 | rs4696717 | C/T |
| chr4 | 9218028 | 4 | 2 | chr4 | 9218028 | rs4974855 | A/C |
| chr4 | 9531030 | 1 | 4 | chr4 | 9531028 | rs6823877 | C/T |
| chr4 | 9645605 | 4 | 3 | chr4 | 9645603 | rs3733584 | A/G |
| chr4 | 9997411 | 2 | 2 | chr4 | 9997410 | rs9990427 | A/G |
| chr4 | 10193126 | 2 | 4 | chr4 | 10193125 | rs6819450 | G/T |
| chr4 | 11050243 | 3 | 1 | chr4 | 11050241 | rs10939305 | C/T |
| chr4 | 13266598 | 4 | 2 | chr4 | 13266597 | rs10805319 | G/T |
| chr4 | 14497786 | 2 | 2 | chr4 | 14497784 | rs35984733 | C/G |
| chr4 | 14761951 | 10 | 2 | chr4 | 14761949 | rs1455204 | A/G |
| chr4 | 16875397 | 3 | 1 | chr4 | 16875395 | rs472953 | A/G |
| chr4 | 19917435 | 2 | 5 | chr4 | 19917433 | rs12641426 | C/T |
| chr4 | 22049514 | 8 | 2 | chr4 | 22049513 | rs6824750 | A/G |
| chr4 | 23676082 | 2 | 2 | chr4 | 23676080 | rs16874921 | C/G |
| chr4 | 23898914 | 2 | 3 | chr4 | 23898912 | rs7667379 | C/T |
| chr4 | 24469433 | 6 | 2 | chr4 | 24469431 | rs800477 | C/T |
| chr4 | 24909493 | 11 | 1 | chr4 | 24909492 | rs316791 | A/G |
| chr4 | 25151165 | 2 | 8 | chr4 | 25151164 | rs55877462 | A/G |
| chr4 | 25582993 | 1 | 3 | chr4 | 25582993 | rs7375697 | A/G |
| chr4 | 26825600 | 2 | 3 | chr4 | 26825598 | rs4639059 | C/T |
| chr4 | 30361286 | 4 | 1 | chr4 | 30361284 | rs10003467 | C/T |
| chr4 | 30709598 | 1 | 5 | chr4 | 30709596 | rs2201945 | A/C |
| chr4 | 31493122 | 2 | 7 | chr4 | 31493120 | rs7658061 | C/T |
| chr4 | 35739387 | 1 | 3 | chr4 | 35739386 | rs61797472 | A/G |
| chr4 | 36007495 | 6 | 10 | chr4 | 36007494 | rs9306932 | A/G |
| chr4 | 36117145 | 1 | 12 | chr4 | 36117143 | rs6828747 | C/T |
| chr4 | 36117145 | 1 | 12 | chr4 | 36117143 | rs55752340 | A/G |
| chr4 | 36137377 | 4 | 3 | chr4 | 36137375 | rs4131542 | G/T |

| | | | | | | | |
|---|---|---|---|---|---|---|---|
| chr4 | 36501610 | 6 | 1 | chr4 | 36501608 | rs2118356 | C/T |
| chr4 | 36657203 | 1 | 4 | chr4 | 36657201 | rs6419096 | C/T |
| chr4 | 37330335 | 2 | 18 | chr4 | 37330334 | rs16993769 | A/G |
| chr4 | 37588033 | 6 | 5 | chr4 | 37588032 | rs2925952 | A/G |
| chr4 | 37846055 | 3 | 7 | chr4 | 37846054 | rs4832749 | A/G |
| chr4 | 37885777 | 1 | 5 | chr4 | 37885776 | rs7670226 | G/T |
| chr4 | 37947260 | 3 | 1 | chr4 | 37947258 | rs34398477 | A/C |
| chr4 | 37996600 | 11 | 1 | chr4 | 37996599 | rs4282192 | G/T |
| chr4 | 38593309 | 3 | 1 | chr4 | 38593308 | rs4240248 | A/C |
| chr4 | 38676282 | 6 | 1 | chr4 | 38676281 | rs73236695 | A/G |
| chr4 | 39214134 | 3 | 2 | chr4 | 39214132 | rs13101951 | C/T |
| chr4 | 39698436 | 1 | 3 | chr4 | 39698435 | rs6838749 | A/G |
| chr4 | 39863991 | 5 | 4 | chr4 | 39863990 | rs1397934 | A/G |
| chr4 | 39888343 | 0 | 0 | chr4 | 39888342 | rs4974973 | C/G |
| chr4 | 40074096 | 9 | 1 | chr4 | 40074095 | rs4861372 | G/T |
| chr4 | 40531587 | 1 | 3 | chr4 | 40531586 | rs7694048 | A/G |
| chr4 | 40913534 | 2 | 9 | chr4 | 40913532 | rs17589402 | A/C |
| chr4 | 41057990 | 3 | 1 | chr4 | 41057988 | rs60137031 | C/T |
| chr4 | 41213350 | 2 | 3 | chr4 | 41213349 | rs6447080 | A/G |
| chr4 | 43248092 | 2 | 5 | chr4 | 43248091 | rs4330382 | A/G |
| chr4 | 47268417 | 4 | 1 | chr4 | 47268416 | rs10004038 | A/G |
| chr4 | 48037802 | 1 | 7 | chr4 | 48037799 | rs66671150 | C/T |
| chr4 | 48973008 | 3 | 1 | chr4 | 48973008 | rs71269488 | C/G |
| chr4 | 48994198 | 15 | 9 | chr4 | 48994195 | rs9700569 | G/T |
| chr4 | 48994198 | 15 | 9 | chr4 | 48994195 | rs61797928 | G/T |
| chr4 | 49000334 | 15 | 9 | chr4 | 49000331 | rs61797928 | G/T |
| chr4 | 49014740 | 9 | 15 | chr4 | 49014740 | rs61797928 | G/T |
| chr4 | 49030802 | 1 | 3 | chr4 | 49030799 | rs71269488 | C/G |
| chr4 | 49354618 | 3 | 63 | chr4 | 49354615 | rs62636966 | A/C |
| chr4 | 52642465 | 2 | 3 | chr4 | 52642463 | rs6827593 | C/T |
| chr4 | 53643822 | 5 | 1 | chr4 | 53643821 | rs4623079 | A/G |
| chr4 | 54460544 | 3 | 1 | chr4 | 54460542 | rs13123862 | C/T |

| | | | | | | | |
|---|---|---|---|---|---|---|---|
| chr4 | 55518396 | 5 | 1 | chr4 | 55518394 | rs7684211 | C/T |
| chr4 | 55957131 | 3 | 1 | chr4 | 55957130 | rs1128141 | C/T |
| chr4 | 56265745 | 4 | 1 | chr4 | 56265744 | rs4496649 | C/T |
| chr4 | 57842472 | 2 | 4 | chr4 | 57842470 | rs17498319 | C/T |
| chr4 | 58109048 | 4 | 1 | chr4 | 58109046 | rs6822252 | C/T |
| chr4 | 58109048 | 4 | 1 | chr4 | 58109047 | rs1372095 | C/T |
| chr4 | 58639329 | 14 | 5 | chr4 | 58639326 | rs2660928 | G/T |
| chr4 | 58748844 | 3 | 2 | chr4 | 58748843 | rs2196055 | A/G |
| chr4 | 59827776 | 3 | 1 | chr4 | 59827773 | rs2879866 | C/T |
| chr4 | 59827776 | 3 | 1 | chr4 | 59827775 | rs72631023 | A/G |
| chr4 | 61049640 | 2 | 2 | chr4 | 61049639 | rs10024983 | A/G |
| chr4 | 63036301 | 9 | 2 | chr4 | 63036299 | rs34133572 | A/C |
| chr4 | 64513525 | 4 | 1 | chr4 | 64513523 | rs13143880 | C/T |
| chr4 | 64960462 | 2 | 2 | chr4 | 64960461 | rs7694849 | A/G |
| chr4 | 65584936 | 2 | 8 | chr4 | 65584934 | rs4642296 | C/T |
| chr4 | 65584936 | 2 | 8 | chr4 | 65584936 | rs1440930 | C/G |
| chr4 | 65790197 | 3 | 1 | chr4 | 65790197 | rs162572 | A/C |
| chr4 | 66775390 | 5 | 2 | chr4 | 66775388 | rs4860206 | A/C |
| chr4 | 67391223 | 4 | 1 | chr4 | 67391222 | rs10471145 | A/G |
| chr4 | 67601986 | 1 | 5 | chr4 | 67601984 | rs13121878 | C/T |
| chr4 | 68467370 | 2 | 3 | chr4 | 68467368 | rs353163 | A/G |
| chr4 | 68591363 | 3 | 5 | chr4 | 68591361 | rs6842364 | C/T |
| chr4 | 72655099 | 3 | 3 | chr4 | 72655098 | rs1043218 | A/G |
| chr4 | 73145765 | 2 | 4 | chr4 | 73145763 | rs4435712 | C/T |
| chr4 | 73541786 | 4 | 1 | chr4 | 73541786 | rs1383935 | G/T |
| chr4 | 74081679 | 7 | 1 | chr4 | 74081677 | rs7676278 | C/T |
| chr4 | 74661695 | 3 | 1 | chr4 | 74661693 | rs1247664 | C/T |
| chr4 | 74806870 | 1 | 4 | chr4 | 74806869 | rs10938091 | G/T |
| chr4 | 75128045 | 1 | 4 | chr4 | 75128043 | rs352042 | A/G |
| chr4 | 75849600 | 3 | 1 | chr4 | 75849599 | rs9996945 | A/G |
| chr4 | 76847957 | 11 | 2 | chr4 | 76847955 | rs7659408 | A/C |
| chr4 | 77029805 | 2 | 3 | chr4 | 77029804 | rs11725766 | A/G |

| | | | | | | | |
|---|---|---|---|---|---|---|---|
| chr4 | 77060772 | 2 | 3 | chr4 | 77060771 | rs2280101 | C/T |
| chr4 | 77080162 | 4 | 2 | chr4 | 77080160 | rs6847716 | C/G |
| chr4 | 79686090 | 3 | 1 | chr4 | 79686088 | rs67325389 | C/T |
| chr4 | 82886807 | 4 | 2 | chr4 | 82886806 | rs17561568 | A/G |
| chr4 | 83767812 | 2 | 3 | chr4 | 83767811 | rs13134864 | A/G |
| chr4 | 83804687 | 3 | 1 | chr4 | 83804685 | rs6830953 | C/T |
| chr4 | 84119284 | 3 | 2 | chr4 | 84119283 | rs7674484 | A/G |
| chr4 | 84160860 | 5 | 1 | chr4 | 84160858 | rs6847981 | C/T |
| chr4 | 84357866 | 1 | 3 | chr4 | 84357863 | rs6535444 | C/G |
| chr4 | 85296897 | 2 | 3 | chr4 | 85296895 | rs28481766 | C/T |
| chr4 | 85710291 | 1 | 3 | chr4 | 85710290 | rs10776519 | A/G |
| chr4 | 88134319 | 1 | 4 | chr4 | 88134318 | rs4693799 | A/G |
| chr4 | 89132145 | 1 | 4 | chr4 | 89132144 | rs4345141 | A/G |
| chr4 | 91096985 | 5 | 2 | chr4 | 91096984 | rs6812004 | A/G |
| chr4 | 92042933 | 4 | 2 | chr4 | 92042932 | rs1377917 | C/T |
| chr4 | 92452790 | 4 | 2 | chr4 | 92452788 | rs6532287 | C/T |
| chr4 | 92845444 | 6 | 1 | chr4 | 92845443 | rs13105299 | A/G |
| chr4 | 93624874 | 10 | 3 | chr4 | 93624872 | rs35400056 | C/T |
| chr4 | 95994124 | 1 | 9 | chr4 | 95994123 | rs1544392 | A/G |
| chr4 | 96327586 | 3 | 3 | chr4 | 96327585 | rs3755880 | C/T |
| chr4 | 99789834 | 6 | 2 | chr4 | 99789832 | rs9307228 | C/T |
| chr4 | 100890148 | 9 | 2 | chr4 | 100890146 | rs1426730 | A/G |
| chr4 | 100991627 | 4 | 1 | chr4 | 100991625 | rs4699767 | C/T |
| chr4 | 104675877 | 3 | 2 | chr4 | 104675875 | rs368218 | A/G |
| chr4 | 105549582 | 1 | 4 | chr4 | 105549581 | rs11097861 | A/G |
| chr4 | 106484131 | 7 | 3 | chr4 | 106484129 | rs2726486 | C/T |
| chr4 | 107131247 | 7 | 1 | chr4 | 107131245 | rs17036424 | C/T |
| chr4 | 109195159 | 11 | 2 | chr4 | 109195157 | rs4386676 | C/T |
| chr4 | 110046425 | 1 | 3 | chr4 | 110046424 | rs1405145 | A/G |
| chr4 | 111307487 | 6 | 3 | chr4 | 111307486 | rs62326607 | A/G |
| chr4 | 111607715 | 7 | 1 | chr4 | 111607713 | rs6814283 | C/T |
| chr4 | 113311990 | 6 | 3 | chr4 | 113311988 | rs4541590 | C/T |

| | | | | | | | |
|---|---|---|---|---|---|---|---|
| chr4 | 115754265 | 1 | 4 | chr4 | 115754264 | rs4585328 | G/T |
| chr4 | 115764163 | 9 | 2 | chr4 | 115764161 | rs4148254 | C/T |
| chr4 | 115764163 | 9 | 2 | chr4 | 115764162 | rs6851610 | A/G |
| chr4 | 116893060 | 2 | 4 | chr4 | 116893059 | rs1382596 | A/G |
| chr4 | 117128781 | 9 | 5 | chr4 | 117128779 | rs35338277 | C/T |
| chr4 | 118066207 | 2 | 3 | chr4 | 118066205 | rs6849680 | C/T |
| chr4 | 118066207 | 2 | 3 | chr4 | 118066206 | rs28532270 | C/G |
| chr4 | 119188807 | 6 | 1 | chr4 | 119188805 | rs13123909 | C/T |
| chr4 | 119873271 | 3 | 1 | chr4 | 119873269 | rs2046779 | C/G |
| chr4 | 120426020 | 5 | 1 | chr4 | 120426019 | rs10010174 | A/G |
| chr4 | 120971015 | 16 | 1 | chr4 | 120971014 | rs7439012 | A/G |
| chr4 | 122211840 | 5 | 1 | chr4 | 122211840 | rs6825650 | G/T |
| chr4 | 122736407 | 7 | 1 | chr4 | 122736405 | rs1531662 | C/T |
| chr4 | 126274193 | 2 | 7 | chr4 | 126274192 | rs2390784 | C/T |
| chr4 | 129643506 | 2 | 2 | chr4 | 129643505 | rs62318206 | A/G |
| chr4 | 130110241 | 1 | 4 | chr4 | 130110239 | rs7655841 | C/T |
| chr4 | 130867902 | 1 | 13 | chr4 | 130867901 | rs2728750 | C/T |
| chr4 | 130911244 | 2 | 3 | chr4 | 130911243 | rs10518556 | C/T |
| chr4 | 132530060 | 5 | 1 | chr4 | 132530059 | rs6534838 | A/G |
| chr4 | 133339421 | 1 | 4 | chr4 | 133339419 | rs13110367 | C/T |
| chr4 | 136156566 | 7 | 1 | chr4 | 136156565 | rs12505849 | A/G |
| chr4 | 137019501 | 10 | 2 | chr4 | 137019499 | rs34930228 | C/T |
| chr4 | 137591113 | 3 | 4 | chr4 | 137591112 | rs62309714 | A/G |
| chr4 | 139557593 | 2 | 3 | chr4 | 139557592 | rs2912460 | A/G |
| chr4 | 139616144 | 1 | 6 | chr4 | 139616142 | rs13148881 | C/T |
| chr4 | 140062639 | 2 | 2 | chr4 | 140062638 | rs6829538 | G/T |
| chr4 | 141007837 | 2 | 3 | chr4 | 141007836 | rs795984 | A/G |
| chr4 | 141043269 | 5 | 8 | chr4 | 141043267 | rs736352 | A/G |
| chr4 | 141043269 | 5 | 8 | chr4 | 141043269 | rs736351 | C/T |
| chr4 | 142026900 | 3 | 1 | chr4 | 142026899 | rs6826361 | A/G |
| chr4 | 144382393 | 2 | 3 | chr4 | 144382392 | rs1440418 | A/G |
| chr4 | 150055264 | 3 | 3 | chr4 | 150055263 | rs7665428 | A/G |

| | | | | | | | | |
|---|---|---|---|---|---|---|---|---|
| chr4 | 150205645 | 2 | 2 | | chr4 | 150205644 | rs6851497 | A/G |
| chr4 | 152603413 | 3 | 2 | | chr4 | 152603411 | rs10454251 | C/T |
| chr4 | 152812382 | 2 | 3 | | chr4 | 152812382 | rs6535809 | A/G |
| chr4 | 153024149 | 2 | 2 | | chr4 | 153024148 | rs1385780 | C/T |
| chr4 | 153142393 | 3 | 1 | | chr4 | 153142392 | rs7684777 | A/G |
| chr4 | 153847612 | 6 | 1 | | chr4 | 153847610 | rs13142918 | C/T |
| chr4 | 154895390 | 3 | 1 | | chr4 | 154895389 | rs6535948 | A/G |
| chr4 | 155412147 | 3 | 3 | | chr4 | 155412146 | rs4461508 | A/G |
| chr4 | 155558901 | 3 | 1 | | chr4 | 155558901 | rs902454 | C/G |
| chr4 | 155700814 | 3 | 3 | | chr4 | 155700812 | rs1025154 | A/G |
| chr4 | 156328384 | 4 | 1 | | chr4 | 156328382 | rs4402990 | A/C |
| chr4 | 158112620 | 4 | 1 | | chr4 | 158112620 | rs1002091 | C/G |
| chr4 | 158553097 | 6 | 1 | | chr4 | 158553095 | rs4234911 | C/T |
| chr4 | 164630523 | 10 | 6 | | chr4 | 164630522 | rs10034495 | A/G |
| chr4 | 165329985 | 1 | 6 | | chr4 | 165329983 | rs2018210 | A/G |
| chr4 | 166219645 | 1 | 5 | | chr4 | 166219644 | rs9999037 | A/G |
| chr4 | 168069936 | 2 | 2 | | chr4 | 168069934 | rs4406047 | C/T |
| chr4 | 168809327 | 2 | 2 | | chr4 | 168809325 | rs1554292 | A/G |
| chr4 | 168819835 | 2 | 3 | | chr4 | 168819833 | rs12649190 | C/T |
| chr4 | 169552109 | 10 | 2 | | chr4 | 169552107 | rs3828493 | C/T |
| chr4 | 170008918 | 6 | 5 | | chr4 | 170008916 | rs2062588 | C/T |
| chr4 | 170113949 | 4 | 2 | | chr4 | 170113947 | rs11132668 | C/T |
| chr4 | 170312246 | 3 | 5 | | chr4 | 170312245 | rs10050030 | A/G |
| chr4 | 171066733 | 5 | 3 | | chr4 | 171066731 | rs7690888 | A/C |
| chr4 | 172129710 | 3 | 2 | | chr4 | 172129708 | rs2661918 | A/G |
| chr4 | 173193591 | 2 | 2 | | chr4 | 173193589 | rs4146318 | C/T |
| chr4 | 173705709 | 4 | 1 | | chr4 | 173705709 | rs1874505 | A/G |
| chr4 | 174189744 | 7 | 2 | | chr4 | 174189742 | rs17059107 | C/T |
| chr4 | 174325814 | 2 | 2 | | chr4 | 174325812 | rs6835098 | C/T |
| chr4 | 174362987 | 5 | 4 | | chr4 | 174362985 | rs9991863 | C/T |
| chr4 | 174709428 | 6 | 1 | | chr4 | 174709427 | rs4695732 | A/G |
| chr4 | 175174154 | 1 | 4 | | chr4 | 175174152 | rs2196377 | A/G |

| | | | | | | | |
|---|---|---|---|---|---|---|---|
| chr4 | 175980878 | 2 | 9 | chr4 | 175980876 | rs11932588 | C/T |
| chr4 | 176032563 | 1 | 6 | chr4 | 176032561 | rs13106865 | A/C |
| chr4 | 176982039 | 4 | 4 | chr4 | 176982037 | rs2877903 | C/T |
| chr4 | 177075685 | 2 | 4 | chr4 | 177075683 | rs7438439 | C/T |
| chr4 | 177569196 | 2 | 5 | chr4 | 177569194 | rs6829504 | C/G |
| chr4 | 177666268 | 2 | 3 | chr4 | 177666266 | rs58696622 | C/T |
| chr4 | 177666268 | 2 | 3 | chr4 | 177666267 | rs309702 | C/T |
| chr4 | 177674568 | 2 | 3 | chr4 | 177674567 | rs373324 | A/G |
| chr4 | 177778196 | 1 | 3 | chr4 | 177778195 | rs1349243 | A/G |
| chr4 | 178089063 | 12 | 4 | chr4 | 178089062 | rs1816542 | C/T |
| chr4 | 178613632 | 3 | 1 | chr4 | 178613631 | rs2333649 | C/T |
| chr4 | 178898115 | 3 | 11 | chr4 | 178898114 | rs11936847 | A/G |
| chr4 | 178939915 | 2 | 3 | chr4 | 178939914 | rs12645758 | A/G |
| chr4 | 181030192 | 7 | 1 | chr4 | 181030189 | rs11131948 | A/C |
| chr4 | 181567031 | 4 | 6 | chr4 | 181567030 | rs2309247 | A/G |
| chr4 | 182451051 | 3 | 3 | chr4 | 182451049 | rs4861473 | A/C |
| chr4 | 182659379 | 5 | 1 | chr4 | 182659377 | rs6837981 | C/T |
| chr4 | 182776394 | 13 | 1 | chr4 | 182776393 | rs2118903 | A/G |
| chr4 | 183261731 | 8 | 1 | chr4 | 183261730 | rs34688675 | G/T |
| chr4 | 183299024 | 2 | 2 | chr4 | 183299021 | rs12650536 | C/G |
| chr4 | 183360845 | 1 | 8 | chr4 | 183360843 | rs7349674 | C/T |
| chr4 | 184314981 | 4 | 5 | chr4 | 184314980 | rs12504402 | A/G |
| chr4 | 184534776 | 0 | 0 | chr4 | 184534775 | rs55961833 | G/T |
| chr4 | 184535232 | 2 | 4 | chr4 | 184535231 | rs62358773 | C/G |
| chr4 | 184546254 | 1 | 3 | chr4 | 184546252 | rs2221830 | -/A/G |
| chr4 | 184557023 | 2 | 3 | chr4 | 184557023 | rs7691819 | C/G |
| chr4 | 184567914 | 1 | 4 | chr4 | 184567912 | rs6840609 | C/G |
| chr4 | 185211313 | 2 | 2 | chr4 | 185211311 | rs4391081 | C/T |
| chr4 | 185374248 | 1 | 3 | chr4 | 185374246 | rs1882328 | C/T |
| chr4 | 185471031 | 1 | 3 | chr4 | 185471030 | rs4862359 | G/T |
| chr4 | 186234077 | 2 | 2 | chr4 | 186234076 | rs6857565 | A/G |
| chr4 | 186739273 | 2 | 6 | chr4 | 186739271 | rs7665363 | C/T |

| | | | | | | | |
|---|---|---|---|---|---|---|---|
| chr4 | 186801551 | 5 | 1 | chr4 | 186801550 | rs28455134 | A/G |
| chr4 | 186879643 | 10 | 14 | chr4 | 186879641 | rs13119888 | C/T |
| chr4 | 186933167 | 1 | 4 | chr4 | 186933165 | rs11728719 | A/C |
| chr4 | 187045154 | 7 | 7 | chr4 | 187045153 | rs6552935 | A/G |
| chr4 | 187278597 | 1 | 5 | chr4 | 187278596 | rs1715050 | C/T |
| chr4 | 187309994 | 3 | 8 | chr4 | 187309993 | rs2276913 | C/T |
| chr4 | 187311031 | 4 | 4 | chr4 | 187311029 | rs62350484 | C/T |
| chr4 | 187312952 | 1 | 4 | chr4 | 187312951 | rs35459248 | A/G |
| chr4 | 187500670 | 3 | 1 | chr4 | 187500668 | rs4861710 | C/T |
| chr4 | 187570904 | 2 | 2 | chr4 | 187570903 | rs7681112 | C/G |
| chr4 | 188244413 | 1 | 3 | chr4 | 188244411 | rs1038598 | G/T |
| chr4 | 188529010 | 3 | 1 | chr4 | 188529008 | rs11132443 | A/C |
| chr4 | 189042946 | 1 | 5 | chr4 | 189042944 | rs4618368 | C/T |
| chr4 | 189155267 | 5 | 1 | chr4 | 189155267 | rs12504373 | G/T |
| chr4 | 189533774 | 1 | 3 | chr4 | 189533772 | rs62351564 | C/T |
| chr4 | 189577133 | 6 | 1 | chr4 | 189577131 | rs7679869 | C/G |
| chr4 | 189616515 | 3 | 1 | chr4 | 189616513 | rs372298 | A/G |
| chr4 | 190807622 | 5 | 2 | chr4 | 190807622 | rs9312409 | C/G |
| chr4 | 190808565 | 3 | 1 | chr4 | 190808564 | rs2598647 | A/C |
| chr4 | 190811483 | 7 | 3 | chr4 | 190811481 | rs6841879 | C/T |
| chr4 | 190811483 | 7 | 3 | chr4 | 190811481 | rs72188018 | -/CTTCCCAT |
| chr4 | 190811483 | 7 | 3 | chr4 | 190811482 | rs2598625 | C/T |
| chr4 | 190837645 | 4 | 7 | chr4 | 190837644 | rs4043302 | -/A/AGCAGCCCAGAGGG/G |
| chr4 | 190871513 | 4 | 11 | chr4 | 190871511 | rs2979457 | C/T |
| chr4 | 191150965 | 5 | 7 | chr4 | 191150964 | rs73027075 | A/G |
| chr4 | 191178254 | 4 | 2 | chr4 | 191178254 | rs71234089 | A/G |
| chr5 | 222362 | 0 | 0 | chr5 | 222360 | rs13159868 | C/T |
| chr5 | 222904 | 2 | 17 | chr5 | 222901 | rs1108867 | A/G |
| chr5 | 222904 | 2 | 17 | chr5 | 222903 | rs66818203 | A/G |
| chr5 | 234761 | 3 | 1 | chr5 | 234761 | rs11133847 | A/G |
| chr5 | 236516 | 2 | 6 | chr5 | 236515 | rs4956991 | A/G |
| chr5 | 353255 | 2 | 3 | chr5 | 353253 | rs2672728 | C/T |

| | | | | | | | |
|---|---|---|---|---|---|---|---|
| chr5 | 353255 | 2 | 3 | chr5 | 353254 | rs2721006 | C/T |
| chr5 | 518511 | 2 | 4 | chr5 | 518509 | rs1035908 | C/T |
| chr5 | 554545 | 2 | 3 | chr5 | 554543 | rs13177896 | C/T |
| chr5 | 731062 | 3 | 2 | chr5 | 731059 | rs61731452 | C/G |
| chr5 | 731062 | 3 | 2 | chr5 | 731061 | rs450628 | A/G |
| chr5 | 738111 | 0 | 0 | chr5 | 738110 | rs413295 | A/G |
| chr5 | 815674 | 2 | 4 | chr5 | 815673 | rs432044 | C/T |
| chr5 | 1092002 | 6 | 1 | chr5 | 1092002 | rs60241368 | C/G |
| chr5 | 1110615 | 3 | 3 | chr5 | 1110614 | rs2241606 | A/G |
| chr5 | 1111558 | 5 | 15 | chr5 | 1111556 | rs10057983 | C/T |
| chr5 | 1111719 | 2 | 5 | chr5 | 1111717 | rs10058105 | C/T |
| chr5 | 1163798 | 3 | 1 | chr5 | 1163796 | rs6864465 | C/T |
| chr5 | 1222112 | 4 | 2 | chr5 | 1222111 | rs7702150 | A/G |
| chr5 | 1278395 | 3 | 5 | chr5 | 1278393 | rs10061504 | C/T |
| chr5 | 1386742 | 2 | 9 | chr5 | 1386741 | rs73024265 | A/G |
| chr5 | 1499389 | 11 | 4 | chr5 | 1499388 | rs2652511 | C/T |
| chr5 | 1543019 | 6 | 1 | chr5 | 1543018 | rs72717531 | C/G |
| chr5 | 1543019 | 6 | 1 | chr5 | 1543019 | rs73031885 | A/G |
| chr5 | 1574893 | 7 | 1 | chr5 | 1574892 | rs11960645 | A/G |
| chr5 | 1776446 | 3 | 5 | chr5 | 1776444 | rs11949447 | C/T |
| chr5 | 1790686 | 5 | 1 | chr5 | 1790686 | rs10051983 | C/G |
| chr5 | 1811806 | 6 | 2 | chr5 | 1811805 | rs11738620 | A/G |
| chr5 | 1904600 | 2 | 3 | chr5 | 1904598 | rs263216 | A/G |
| chr5 | 1941359 | 9 | 2 | chr5 | 1941358 | rs260407 | C/G/T |
| chr5 | 1969531 | 2 | 3 | chr5 | 1969529 | rs6879297 | A/C |
| chr5 | 1983864 | 3 | 1 | chr5 | 1983863 | rs7444647 | A/G |
| chr5 | 2250448 | 3 | 4 | chr5 | 2250447 | rs11747063 | A/G |
| chr5 | 2253304 | 3 | 4 | chr5 | 2253302 | rs11133936 | C/G |
| chr5 | 2444454 | 4 | 12 | chr5 | 2444452 | rs383748 | A/G |
| chr5 | 2464900 | 2 | 6 | chr5 | 2464898 | rs315890 | C/T |
| chr5 | 2609981 | 4 | 10 | chr5 | 2609979 | rs7711137 | C/T |
| chr5 | 2693123 | 1 | 4 | chr5 | 2693122 | rs2897047 | C/T |

| | | | | | | | |
|---|---|---|---|---|---|---|---|
| chr5 | 2710193 | 0 | 0 | chr5 | 2710192 | rs6883186 | A/G |
| chr5 | 2829720 | 3 | 1 | chr5 | 2829718 | rs4866493 | C/G |
| chr5 | 2948097 | 6 | 1 | chr5 | 2948096 | rs2934565 | C/T |
| chr5 | 3288697 | 1 | 3 | chr5 | 3288695 | rs251816 | A/G |
| chr5 | 3619192 | 1 | 5 | chr5 | 3619190 | rs828310 | A/G |
| chr5 | 3784378 | 1 | 4 | chr5 | 3784376 | rs10075006 | C/T |
| chr5 | 4095790 | 2 | 7 | chr5 | 4095789 | rs2452882 | C/T |
| chr5 | 4309548 | 3 | 4 | chr5 | 4309546 | rs263733 | C/T |
| chr5 | 4318682 | 3 | 3 | chr5 | 4318680 | rs463734 | C/T |
| chr5 | 4584372 | 3 | 1 | chr5 | 4584370 | rs12514688 | C/T |
| chr5 | 4931025 | 3 | 4 | chr5 | 4931023 | rs56128967 | C/T |
| chr5 | 4995503 | 3 | 1 | chr5 | 4995501 | rs12519737 | C/G |
| chr5 | 5005192 | 3 | 2 | chr5 | 5005191 | rs25799 | C/T |
| chr5 | 5300582 | 1 | 6 | chr5 | 5300581 | rs2913637 | A/G |
| chr5 | 5413015 | 6 | 1 | chr5 | 5413014 | rs2964460 | C/T |
| chr5 | 5581231 | 2 | 3 | chr5 | 5581229 | rs2591708 | A/G |
| chr5 | 5599894 | 3 | 1 | chr5 | 5599892 | rs1871474 | C/T |
| chr5 | 5648935 | 3 | 1 | chr5 | 5648933 | rs2448436 | A/G |
| chr5 | 5745980 | 2 | 2 | chr5 | 5745978 | rs1501824 | C/T |
| chr5 | 7939105 | 2 | 2 | chr5 | 7939103 | rs162037 | C/T |
| chr5 | 8230920 | 8 | 1 | chr5 | 8230919 | rs336174 | C/T |
| chr5 | 8575021 | 2 | 3 | chr5 | 8575020 | rs415234 | A/G |
| chr5 | 8615480 | 2 | 6 | chr5 | 8615478 | rs756758 | A/G |
| chr5 | 9761887 | 3 | 1 | chr5 | 9761886 | rs3105161 | G/T |
| chr5 | 10026935 | 2 | 5 | chr5 | 10026934 | rs10072891 | A/G |
| chr5 | 10085708 | 4 | 2 | chr5 | 10085707 | rs7735551 | A/G |
| chr5 | 10193664 | 2 | 2 | chr5 | 10193662 | rs2136195 | A/G |
| chr5 | 10512710 | 2 | 6 | chr5 | 10512708 | rs2088625 | A/G |
| chr5 | 10620512 | 10 | 4 | chr5 | 10620510 | rs814578 | C/T |
| chr5 | 10620512 | 10 | 4 | chr5 | 10620511 | rs71599536 | C/G |
| chr5 | 10691181 | 3 | 2 | chr5 | 10691179 | rs11745612 | C/T |
| chr5 | 10737943 | 1 | 3 | chr5 | 10737941 | rs3822411 | C/T |

| | | | | | | | |
|---|---|---|---|---|---|---|---|
| chr5 | 12269225 | 1 | 7 | chr5 | 12269223 | rs4557410 | C/T |
| chr5 | 13903750 | 1 | 3 | chr5 | 13903749 | rs6554820 | A/G |
| chr5 | 14476837 | 1 | 3 | chr5 | 14476835 | rs890937 | C/T |
| chr5 | 15012725 | 2 | 4 | chr5 | 15012724 | rs856529 | A/C |
| chr5 | 17116444 | 2 | 3 | chr5 | 17116443 | rs2086608 | A/G |
| chr5 | 17346762 | 1 | 3 | chr5 | 17346760 | rs298568 | G/T |
| chr5 | 17827601 | 4 | 3 | chr5 | 17827600 | rs1822399 | A/C |
| chr5 | 17831218 | 2 | 5 | chr5 | 17831217 | rs7707669 | A/G |
| chr5 | 17841931 | 2 | 2 | chr5 | 17841929 | rs1443398 | A/G |
| chr5 | 18343436 | 1 | 6 | chr5 | 18343434 | rs1504206 | C/T |
| chr5 | 18635767 | 1 | 3 | chr5 | 18635765 | rs1496286 | C/T |
| chr5 | 19105736 | 1 | 3 | chr5 | 19105735 | rs62350862 | G/T |
| chr5 | 24700473 | 4 | 1 | chr5 | 24700471 | rs9293149 | C/T |
| chr5 | 25890558 | 1 | 3 | chr5 | 25890556 | rs6878799 | C/T |
| chr5 | 26803705 | 1 | 3 | chr5 | 26803703 | rs10038002 | C/T |
| chr5 | 28376921 | 1 | 3 | chr5 | 28376919 | rs10039943 | C/T |
| chr5 | 28987094 | 3 | 2 | chr5 | 28987093 | rs314813 | A/G |
| chr5 | 29191764 | 3 | 6 | chr5 | 29191762 | rs309669 | A/G |
| chr5 | 31573993 | 5 | 1 | chr5 | 31573991 | rs7720494 | A/C |
| chr5 | 31954743 | 1 | 3 | chr5 | 31954741 | rs4867088 | C/T |
| chr5 | 32123559 | 3 | 1 | chr5 | 32123558 | rs157497 | A/G |
| chr5 | 32281360 | 1 | 3 | chr5 | 32281358 | rs59953683 | C/T |
| chr5 | 33668475 | 1 | 9 | chr5 | 33668473 | rs6897038 | C/T |
| chr5 | 34458363 | 6 | 5 | chr5 | 34458361 | rs12519686 | A/C |
| chr5 | 34521650 | 2 | 5 | chr5 | 34521648 | rs6451132 | C/T |
| chr5 | 35014753 | 2 | 10 | chr5 | 35014751 | rs163590 | A/G |
| chr5 | 35066004 | 3 | 4 | chr5 | 35066002 | rs425572 | A/G |
| chr5 | 35071423 | 7 | 7 | chr5 | 35071422 | rs189362 | G/T |
| chr5 | 35589152 | 4 | 1 | chr5 | 35589151 | rs286421 | A/G |
| chr5 | 35983288 | 1 | 5 | chr5 | 35983287 | rs13172042 | C/G |
| chr5 | 36089341 | 3 | 3 | chr5 | 36089340 | rs583595 | A/C |
| chr5 | 36507717 | 4 | 3 | chr5 | 36507714 | rs4645350 | C/T |

| | | | | | | | |
|---|---|---|---|---|---|---|---|
| chr5 | 36507717 | 4 | 3 | chr5 | 36507715 | rs2937554 | A/G |
| chr5 | 36646365 | 5 | 1 | chr5 | 36646364 | rs72732525 | A/G |
| chr5 | 36842946 | 1 | 3 | chr5 | 36842945 | rs292168 | A/G |
| chr5 | 37723767 | 3 | 3 | chr5 | 37723766 | rs4371784 | A/G |
| chr5 | 37892985 | 2 | 2 | chr5 | 37892983 | rs2973114 | A/G |
| chr5 | 38052542 | 4 | 2 | chr5 | 38052539 | rs10036158 | C/T |
| chr5 | 38052542 | 4 | 2 | chr5 | 38052540 | rs6888603 | C/G |
| chr5 | 39293642 | 24 | 1 | chr5 | 39293640 | rs2897020 | A/G |
| chr5 | 40108499 | 2 | 2 | chr5 | 40108498 | rs6896408 | A/G |
| chr5 | 40130620 | 2 | 2 | chr5 | 40130619 | rs10038202 | A/G |
| chr5 | 41080374 | 3 | 2 | chr5 | 41080372 | rs325857 | C/G |
| chr5 | 41097473 | 1 | 6 | chr5 | 41097471 | rs1023840 | C/T |
| chr5 | 41097473 | 1 | 6 | chr5 | 41097472 | rs865093 | A/G |
| chr5 | 43099910 | 1 | 7 | chr5 | 43099908 | rs12521684 | C/T |
| chr5 | 43415549 | 3 | 1 | chr5 | 43415548 | rs11741797 | A/G |
| chr5 | 44750088 | 2 | 5 | chr5 | 44750086 | rs2218080 | A/G |
| chr5 | 45850034 | 4 | 2 | chr5 | 45850031 | rs13176359 | C/T |
| chr5 | 52120123 | 2 | 2 | chr5 | 52120121 | rs10067659 | C/G |
| chr5 | 52320875 | 5 | 2 | chr5 | 52320873 | rs28095 | C/T |
| chr5 | 52378255 | 10 | 3 | chr5 | 52378254 | rs1421934 | C/T |
| chr5 | 52723399 | 4 | 2 | chr5 | 52723397 | rs34268497 | C/T |
| chr5 | 53421286 | 5 | 5 | chr5 | 53421285 | rs695922 | C/T |
| chr5 | 54228424 | 1 | 3 | chr5 | 54228422 | rs67500686 | A/C |
| chr5 | 54565388 | 1 | 6 | chr5 | 54565386 | rs163104 | A/C |
| chr5 | 55037135 | 6 | 1 | chr5 | 55037133 | rs11745701 | A/C |
| chr5 | 55261085 | 3 | 3 | chr5 | 55261084 | rs324995 | G/T |
| chr5 | 55858837 | 3 | 1 | chr5 | 55858835 | rs29997 | A/G |
| chr5 | 55953714 | 1 | 4 | chr5 | 55953712 | rs2448425 | A/G |
| chr5 | 56964132 | 1 | 11 | chr5 | 56964131 | rs110873 | C/T |
| chr5 | 57093717 | 2 | 2 | chr5 | 57093716 | rs4508990 | G/T |
| chr5 | 57190292 | 2 | 3 | chr5 | 57190291 | rs2408854 | A/G |
| chr5 | 57287724 | 1 | 4 | chr5 | 57287723 | rs1027164 | A/C |

| | | | | | | | |
|---|---|---|---|---|---|---|---|
| chr5 | 58669349 | 3 | 1 | chr5 | 58669347 | rs35259 | C/T |
| chr5 | 60276742 | 5 | 2 | chr5 | 60276742 | rs158922 | C/T |
| chr5 | 61004232 | 3 | 3 | chr5 | 61004231 | rs36049 | C/T |
| chr5 | 61048648 | 3 | 1 | chr5 | 61048646 | rs166142 | G/T |
| chr5 | 61468616 | 3 | 3 | chr5 | 61468615 | rs2961836 | A/G |
| chr5 | 63784130 | 4 | 2 | chr5 | 63784128 | rs271718 | C/T |
| chr5 | 65143971 | 4 | 9 | chr5 | 65143970 | rs2254485 | A/G |
| chr5 | 67315137 | 3 | 3 | chr5 | 67315135 | rs28654894 | C/T |
| chr5 | 69232092 | 0 | 0 | chr5 | 69232090 | rs9772189 | C/T |
| chr5 | 69233712 | 0 | 0 | chr5 | 69233710 | rs9801909 | C/T |
| chr5 | 69233712 | 0 | 0 | chr5 | 69233710 | rs71206057 | A/G |
| chr5 | 70107158 | 0 | 0 | chr5 | 70107156 | rs9772189 | C/T |
| chr5 | 70108778 | 0 | 0 | chr5 | 70108776 | rs9801909 | C/T |
| chr5 | 70108778 | 0 | 0 | chr5 | 70108776 | rs71206057 | A/G |
| chr5 | 73690703 | 3 | 2 | chr5 | 73690701 | rs376398 | C/G |
| chr5 | 75443992 | 4 | 11 | chr5 | 75443990 | rs30249 | C/T |
| chr5 | 75965589 | 2 | 5 | chr5 | 75965588 | rs1697844 | A/G |
| chr5 | 76002988 | 3 | 2 | chr5 | 76002987 | rs7734335 | A/G |
| chr5 | 76271915 | 2 | 2 | chr5 | 76271913 | rs6888438 | C/T |
| chr5 | 76271915 | 2 | 2 | chr5 | 76271914 | rs6862185 | A/G |
| chr5 | 76387729 | 3 | 1 | chr5 | 76387728 | rs72765112 | A/G |
| chr5 | 77941742 | 1 | 3 | chr5 | 77941741 | rs10223112 | A/G |
| chr5 | 79227147 | 2 | 2 | chr5 | 79227145 | rs6883826 | C/T |
| chr5 | 79668680 | 4 | 4 | chr5 | 79668679 | rs10037935 | A/G |
| chr5 | 80330357 | 5 | 1 | chr5 | 80330356 | rs569262 | A/C |
| chr5 | 81267214 | 3 | 1 | chr5 | 81267213 | rs6452438 | A/G |
| chr5 | 81754345 | 2 | 2 | chr5 | 81754343 | rs224934 | A/G |
| chr5 | 82509401 | 3 | 4 | chr5 | 82509400 | rs10040363 | A/G |
| chr5 | 83422735 | 3 | 5 | chr5 | 83422733 | rs6452563 | C/T |
| chr5 | 84497336 | 1 | 4 | chr5 | 84497334 | rs7446529 | C/T |
| chr5 | 84497336 | 1 | 4 | chr5 | 84497334 | rs66555462 | CA/TG |
| chr5 | 84497336 | 1 | 4 | chr5 | 84497335 | rs10474177 | A/G |

| | | | | | | | |
|---|---|---|---|---|---|---|---|
| chr5 | 86393702 | 3 | 1 | chr5 | 86393700 | rs4339389 | A/C |
| chr5 | 87071884 | 5 | 1 | chr5 | 87071883 | rs1458200 | C/T |
| chr5 | 94417331 | 3 | 2 | chr5 | 94417329 | rs1426098 | A/G |
| chr5 | 96146016 | 0 | 0 | chr5 | 96146014 | rs30376 | C/T |
| chr5 | 96221036 | 8 | 5 | chr5 | 96221035 | rs1230366 | A/G |
| chr5 | 96701467 | 1 | 3 | chr5 | 96701466 | rs12652541 | G/T |
| chr5 | 97221448 | 1 | 3 | chr5 | 97221446 | rs4533913 | C/T |
| chr5 | 97535701 | 4 | 3 | chr5 | 97535699 | rs4269306 | C/T |
| chr5 | 97878373 | 2 | 8 | chr5 | 97878372 | rs17658337 | A/G |
| chr5 | 97899518 | 11 | 3 | chr5 | 97899516 | rs10478759 | C/T |
| chr5 | 99186588 | 2 | 6 | chr5 | 99186586 | rs7446518 | C/T |
| chr5 | 100960880 | 5 | 2 | chr5 | 100960879 | rs3907457 | A/G |
| chr5 | 101123152 | 2 | 3 | chr5 | 101123151 | rs1600276 | A/G |
| chr5 | 103246170 | 2 | 2 | chr5 | 103246169 | rs6890911 | A/G |
| chr5 | 103479624 | 1 | 3 | chr5 | 103479623 | rs10515364 | A/G |
| chr5 | 104233891 | 2 | 3 | chr5 | 104233890 | rs1708598 | A/G |
| chr5 | 104388553 | 1 | 3 | chr5 | 104388551 | rs6872255 | C/T |
| chr5 | 107208811 | 1 | 4 | chr5 | 107208811 | rs67994848 | G/T |
| chr5 | 107243530 | 1 | 4 | chr5 | 107243528 | rs10067015 | C/T |
| chr5 | 108099555 | 2 | 4 | chr5 | 108099553 | rs9285863 | C/T |
| chr5 | 109089483 | 1 | 6 | chr5 | 109089482 | rs952826 | A/G |
| chr5 | 112285490 | 3 | 1 | chr5 | 112285489 | rs151980 | C/G |
| chr5 | 113735740 | 2 | 2 | chr5 | 113735739 | rs181949 | C/T |
| chr5 | 114456187 | 1 | 18 | chr5 | 114456186 | rs2416399 | C/G |
| chr5 | 115875946 | 1 | 5 | chr5 | 115875944 | rs6897947 | C/T |
| chr5 | 115919689 | 8 | 1 | chr5 | 115919687 | rs153641 | A/G |
| chr5 | 116107929 | 2 | 2 | chr5 | 116107927 | rs26674 | A/C |
| chr5 | 116578977 | 3 | 3 | chr5 | 116578975 | rs7702997 | C/T |
| chr5 | 116719232 | 8 | 2 | chr5 | 116719231 | rs7716581 | A/G |
| chr5 | 118323626 | 10 | 1 | chr5 | 118323624 | rs6894429 | C/T |
| chr5 | 118996990 | 3 | 3 | chr5 | 118996989 | rs328696 | C/T |
| chr5 | 119317577 | 7 | 6 | chr5 | 119317576 | rs4360068 | A/G |

| | | | | | | | | |
|---|---|---|---|---|---|---|---|---|
| chr5 | 120778285 | 1 | 3 | chr5 | 120778284 | rs13174895 | A/G |
| chr5 | 121926733 | 6 | 3 | chr5 | 121926732 | rs7720605 | G/T |
| chr5 | 123021555 | 3 | 2 | chr5 | 123021554 | rs1862264 | A/G |
| chr5 | 123254352 | 8 | 3 | chr5 | 123254350 | rs330429 | C/T |
| chr5 | 123856467 | 7 | 6 | chr5 | 123856466 | rs4331893 | A/G |
| chr5 | 126545250 | 3 | 1 | chr5 | 126545248 | rs248100 | C/T |
| chr5 | 127580488 | 0 | 0 | chr5 | 127580486 | rs6866881 | A/C |
| chr5 | 127580488 | 0 | 0 | chr5 | 127580487 | rs2617613 | A/G |
| chr5 | 127580488 | 0 | 0 | chr5 | 127580488 | rs2546147 | A/G |
| chr5 | 129815960 | 3 | 3 | chr5 | 129815958 | rs7731636 | C/T |
| chr5 | 131625291 | 1 | 4 | chr5 | 131625290 | rs10463891 | A/G |
| chr5 | 131733356 | 1 | 3 | chr5 | 131733356 | rs2631367 | C/G |
| chr5 | 132922561 | 1 | 3 | chr5 | 132922560 | rs30488 | A/G |
| chr5 | 133031682 | 1 | 3 | chr5 | 133031681 | rs7706100 | A/G |
| chr5 | 133414474 | 3 | 4 | chr5 | 133414473 | rs41564532 | A/G |
| chr5 | 133450715 | 2 | 4 | chr5 | 133450714 | rs244689 | C/T |
| chr5 | 133478395 | 3 | 1 | chr5 | 133478395 | rs173424 | C/T |
| chr5 | 133856255 | 8 | 1 | chr5 | 133856254 | rs7701346 | A/G |
| chr5 | 135059140 | 2 | 2 | chr5 | 135059137 | rs11746356 | C/T |
| chr5 | 135059140 | 2 | 2 | chr5 | 135059137 | rs35106113 | CA/TC |
| chr5 | 135059140 | 2 | 2 | chr5 | 135059138 | rs11739844 | A/C |
| chr5 | 135151806 | 2 | 2 | chr5 | 135151806 | rs72667122 | C/G |
| chr5 | 135176555 | 9 | 4 | chr5 | 135176553 | rs3930698 | A/G |
| chr5 | 135468263 | 0 | 0 | chr5 | 135468262 | rs12521857 | A/G |
| chr5 | 135892976 | 2 | 2 | chr5 | 135892975 | rs11952460 | A/G |
| chr5 | 136259768 | 5 | 2 | chr5 | 136259767 | rs55869761 | A/G |
| chr5 | 136965778 | 3 | 6 | chr5 | 136965777 | rs6596399 | A/G |
| chr5 | 137391073 | 2 | 3 | chr5 | 137391071 | rs9790993 | C/T |
| chr5 | 137397068 | 12 | 1 | chr5 | 137397065 | rs1462570 | A/G |
| chr5 | 137701067 | 2 | 3 | chr5 | 137701064 | rs17171800 | C/G |
| chr5 | 137701067 | 2 | 3 | chr5 | 137701065 | rs3756766 | A/C |
| chr5 | 138545134 | 27 | 5 | chr5 | 138545133 | rs13160445 | A/G |

| | | | | | | | |
|---|---|---|---|---|---|---|---|
| chr5 | 139025188 | 2 | 3 | chr5 | 139025186 | rs6580190 | C/T |
| chr5 | 139033951 | 5 | 8 | chr5 | 139033950 | rs6580192 | A/G |
| chr5 | 139147863 | 6 | 4 | chr5 | 139147863 | rs735795 | C/G |
| chr5 | 139219105 | 7 | 1 | chr5 | 139219104 | rs3822741 | C/T |
| chr5 | 140537964 | 4 | 2 | chr5 | 140537962 | rs17844481 | C/T |
| chr5 | 140551711 | 1 | 10 | chr5 | 140551709 | rs4912742 | C/T |
| chr5 | 140574044 | 4 | 2 | chr5 | 140574042 | rs17844481 | C/T |
| chr5 | 141052229 | 3 | 1 | chr5 | 141052227 | rs17287030 | C/T |
| chr5 | 141072889 | 1 | 3 | chr5 | 141072888 | rs248618 | G/T |
| chr5 | 141130625 | 7 | 1 | chr5 | 141130623 | rs152356 | C/T |
| chr5 | 141362769 | 5 | 6 | chr5 | 141362767 | rs252114 | A/G |
| chr5 | 141559388 | 2 | 2 | chr5 | 141559387 | rs2906071 | C/T |
| chr5 | 141617242 | 4 | 1 | chr5 | 141617241 | rs4912636 | G/T |
| chr5 | 144170367 | 3 | 2 | chr5 | 144170365 | rs6580331 | C/T |
| chr5 | 145392033 | 6 | 1 | chr5 | 145392032 | rs6580403 | A/G |
| chr5 | 145419841 | 10 | 1 | chr5 | 145419839 | rs2962525 | C/T |
| chr5 | 147188237 | 3 | 1 | chr5 | 147188235 | rs6580502 | C/T |
| chr5 | 147546317 | 3 | 2 | chr5 | 147546316 | rs12717962 | -/A/G |
| chr5 | 147546317 | 3 | 2 | chr5 | 147546316 | rs62950160 | A/G |
| chr5 | 148120890 | 5 | 2 | chr5 | 148120889 | rs919723 | G/T |
| chr5 | 149390713 | 3 | 1 | chr5 | 149390711 | rs2278394 | A/C |
| chr5 | 149655015 | 9 | 1 | chr5 | 149655013 | rs891940 | A/G |
| chr5 | 149891180 | 2 | 2 | chr5 | 149891178 | rs12517252 | C/T |
| chr5 | 150458488 | 2 | 2 | chr5 | 150458486 | rs7707871 | C/T |
| chr5 | 152087301 | 3 | 2 | chr5 | 152087300 | rs72799148 | A/G |
| chr5 | 153979888 | 2 | 3 | chr5 | 153979887 | rs13168119 | A/G |
| chr5 | 154116211 | 5 | 2 | chr5 | 154116208 | rs960499 | C/T |
| chr5 | 155763202 | 3 | 12 | chr5 | 155763201 | rs12054876 | C/G |
| chr5 | 156060198 | 5 | 3 | chr5 | 156060197 | rs39928 | A/G |
| chr5 | 157382327 | 6 | 9 | chr5 | 157382326 | rs4704910 | A/G |
| chr5 | 157388769 | 1 | 5 | chr5 | 157388768 | rs17229224 | A/G |
| chr5 | 158465838 | 1 | 4 | chr5 | 158465837 | rs6862454 | A/G |

| | | | | | | | |
|---|---|---|---|---|---|---|---|
| chr5 | 158920166 | 2 | 5 | chr5 | 158920165 | rs6895787 | A/G |
| chr5 | 159238271 | 2 | 4 | chr5 | 159238270 | rs11739251 | G/T |
| chr5 | 159598176 | 0 | 0 | chr5 | 159598176 | rs2277954 | C/T |
| chr5 | 160215867 | 1 | 3 | chr5 | 160215866 | rs62392648 | G/T |
| chr5 | 161026138 | 2 | 2 | chr5 | 161026137 | rs13163878 | A/G |
| chr5 | 162589498 | 1 | 3 | chr5 | 162589497 | rs1001987 | C/T |
| chr5 | 163239625 | 5 | 1 | chr5 | 163239623 | rs9313987 | C/T |
| chr5 | 163354167 | 3 | 3 | chr5 | 163354165 | rs6898276 | C/T |
| chr5 | 164526134 | 4 | 2 | chr5 | 164526132 | rs2861139 | A/G |
| chr5 | 164694818 | 3 | 1 | chr5 | 164694817 | rs35245192 | A/G |
| chr5 | 164834095 | 1 | 3 | chr5 | 164834093 | rs72813333 | C/T |
| chr5 | 166370994 | 2 | 2 | chr5 | 166370992 | rs10044459 | C/T |
| chr5 | 166760039 | 2 | 3 | chr5 | 166760037 | rs1158821 | A/C |
| chr5 | 167072563 | 4 | 2 | chr5 | 167072562 | rs1459072 | A/G |
| chr5 | 167143050 | 4 | 5 | chr5 | 167143049 | rs2973662 | C/T |
| chr5 | 167529501 | 5 | 3 | chr5 | 167529500 | rs7711412 | G/T |
| chr5 | 167776027 | 3 | 1 | chr5 | 167776026 | rs55919710 | G/T |
| chr5 | 168209425 | 4 | 1 | chr5 | 168209424 | rs2974425 | A/G |
| chr5 | 169146053 | 7 | 7 | chr5 | 169146053 | rs1863994 | A/G |
| chr5 | 170131892 | 1 | 4 | chr5 | 170131890 | rs12173179 | C/T |
| chr5 | 170781587 | 1 | 5 | chr5 | 170781585 | rs4597987 | C/T |
| chr5 | 170909975 | 1 | 3 | chr5 | 170909974 | rs1432965 | C/T |
| chr5 | 171900382 | 2 | 2 | chr5 | 171900380 | rs17705749 | C/T |
| chr5 | 172093103 | 12 | 7 | chr5 | 172093102 | rs324340 | A/G |
| chr5 | 172137607 | 2 | 2 | chr5 | 172137605 | rs2249619 | C/T |
| chr5 | 172258128 | 1 | 3 | chr5 | 172258126 | rs59184418 | C/T |
| chr5 | 172651627 | 7 | 1 | chr5 | 172651625 | rs4242165 | C/T |
| chr5 | 172657723 | 1 | 3 | chr5 | 172657721 | rs11744625 | C/G |
| chr5 | 172737516 | 3 | 5 | chr5 | 172737515 | rs11134791 | G/T |
| chr5 | 172802201 | 3 | 2 | chr5 | 172802200 | rs11743405 | G/T |
| chr5 | 173209851 | 1 | 3 | chr5 | 173209850 | rs10055764 | A/G |
| chr5 | 173696845 | 3 | 2 | chr5 | 173696844 | rs2913487 | A/G |

| | | | | | | | |
|---|---|---|---|---|---|---|---|
| chr5 | 174179278 | 6 | 1 | chr5 | 174179276 | rs6874836 | C/T |
| chr5 | 174830619 | 3 | 3 | chr5 | 174830617 | rs2644648 | C/T |
| chr5 | 175262887 | 8 | 4 | chr5 | 175262886 | rs62397792 | C/T |
| chr5 | 175492615 | 1 | 4 | chr5 | 175492614 | rs6556238 | A/G |
| chr5 | 175928542 | 0 | 0 | chr5 | 175928540 | rs10056130 | C/T |
| chr5 | 176031443 | 3 | 1 | chr5 | 176031442 | rs11749406 | A/G |
| chr5 | 176221057 | 3 | 6 | chr5 | 176221056 | rs10866703 | A/G |
| chr5 | 176469009 | 7 | 1 | chr5 | 176469007 | rs244725 | A/G |
| chr5 | 176705697 | 3 | 2 | chr5 | 176705696 | rs9313758 | G/T |
| chr5 | 177295540 | 4 | 8 | chr5 | 177295538 | rs11249782 | C/T |
| chr5 | 177295540 | 4 | 8 | chr5 | 177295538 | rs62397792 | C/T |
| chr5 | 177324584 | 4 | 16 | chr5 | 177324582 | rs10072261 | C/T |
| chr5 | 177426765 | 3 | 4 | chr5 | 177426764 | rs7443165 | A/G |
| chr5 | 177487339 | 1 | 3 | chr5 | 177487337 | rs10903277 | C/T |
| chr5 | 177636649 | 1 | 9 | chr5 | 177636647 | rs2913839 | A/G |
| chr5 | 177652227 | 1 | 5 | chr5 | 177652225 | rs2913821 | A/G |
| chr5 | 177815154 | 3 | 3 | chr5 | 177815152 | rs2647688 | C/T |
| chr5 | 177826488 | 10 | 4 | chr5 | 177826486 | rs2647700 | A/G |
| chr5 | 177848395 | 3 | 1 | chr5 | 177848393 | rs35346263 | C/T |
| chr5 | 178158733 | 3 | 2 | chr5 | 178158731 | rs34040448 | C/T |
| chr5 | 178190465 | 3 | 9 | chr5 | 178190464 | rs4073864 | A/G |
| chr5 | 178346423 | 7 | 6 | chr5 | 178346421 | rs2256966 | A/G |
| chr5 | 178358926 | 3 | 8 | chr5 | 178358924 | rs11249608 | C/T |
| chr5 | 178492018 | 0 | 0 | chr5 | 178492016 | rs3822594 | C/G |
| chr5 | 178623382 | 4 | 2 | chr5 | 178623381 | rs42875 | C/T |
| chr5 | 178743224 | 2 | 8 | chr5 | 178743222 | rs624162 | A/C |
| chr5 | 178764931 | 2 | 13 | chr5 | 178764930 | rs637910 | C/T |
| chr5 | 178811314 | 4 | 4 | chr5 | 178811313 | rs1814610 | A/G |
| chr5 | 178983873 | 6 | 5 | chr5 | 178983873 | rs4590149 | G/T |
| chr5 | 179019439 | 5 | 2 | chr5 | 179019438 | rs28833246 | A/G |
| chr5 | 179316422 | 2 | 4 | chr5 | 179316420 | rs155576 | C/T |
| chr5 | 179370407 | 2 | 2 | chr5 | 179370405 | rs10068039 | C/T |

| | | | | | | | |
|---|---|---|---|---|---|---|---|
| chr5 | 179489124 | 4 | 1 | chr5 | 179489123 | rs1867455 | C/T |
| chr5 | 179498315 | 3 | 2 | chr5 | 179498313 | rs7707107 | C/T |
| chr5 | 179605432 | 4 | 1 | chr5 | 179605430 | rs4362908 | C/T |
| chr5 | 179670844 | 4 | 4 | chr5 | 179670842 | rs3805705 | A/G |
| chr5 | 179707621 | 4 | 1 | chr5 | 179707620 | rs4700736 | A/G |
| chr5 | 179750814 | 1 | 4 | chr5 | 179750813 | rs4700949 | A/G |
| chr5 | 179820518 | 2 | 2 | chr5 | 179820516 | rs10434807 | C/T |
| chr5 | 180521665 | 2 | 5 | chr5 | 180521664 | rs6865622 | A/G |
| chr6 | 229613 | 1 | 5 | chr6 | 229612 | rs1150764 | C/T |
| chr6 | 259182 | 6 | 1 | chr6 | 259180 | rs2797319 | C/T |
| chr6 | 294167 | 3 | 9 | chr6 | 294165 | rs2666939 | C/T |
| chr6 | 980144 | 3 | 12 | chr6 | 980143 | rs1033484 | C/T |
| chr6 | 993309 | 7 | 5 | chr6 | 993307 | rs10793859 | C/T |
| chr6 | 999721 | 1 | 5 | chr6 | 999719 | rs9406246 | C/T |
| chr6 | 1045777 | 2 | 5 | chr6 | 1045776 | rs2050158 | C/T |
| chr6 | 1263551 | 3 | 2 | chr6 | 1263549 | rs951319 | C/G |
| chr6 | 1280970 | 3 | 2 | chr6 | 1280968 | rs9689020 | C/T |
| chr6 | 1500379 | 3 | 1 | chr6 | 1500377 | rs9391924 | C/T |
| chr6 | 1525958 | 3 | 2 | chr6 | 1525955 | rs2816222 | C/T |
| chr6 | 1825107 | 1 | 3 | chr6 | 1825106 | rs10484694 | A/C |
| chr6 | 1936493 | 1 | 3 | chr6 | 1936492 | rs12198576 | C/G |
| chr6 | 2627824 | 2 | 4 | chr6 | 2627822 | rs942491 | C/T |
| chr6 | 2708726 | 7 | 2 | chr6 | 2708724 | rs13196532 | C/T |
| chr6 | 3195323 | 3 | 2 | chr6 | 3195322 | rs6596957 | A/G |
| chr6 | 3317487 | 2 | 2 | chr6 | 3317484 | rs1079286 | A/G |
| chr6 | 3317487 | 2 | 2 | chr6 | 3317484 | rs71530349 | CT/TC |
| chr6 | 3317487 | 2 | 2 | chr6 | 3317485 | rs1079285 | A/G |
| chr6 | 3373215 | 0 | 0 | chr6 | 3373214 | rs6914698 | A/G |
| chr6 | 3718118 | 4 | 1 | chr6 | 3718117 | rs12206351 | A/G |
| chr6 | 3728467 | 4 | 1 | chr6 | 3728466 | rs12154197 | A/G |
| chr6 | 3894454 | 2 | 2 | chr6 | 3894453 | rs9378390 | A/G |
| chr6 | 3998889 | 3 | 2 | chr6 | 3998887 | rs853399 | C/T |

| | | | | | | | |
|---|---|---|---|---|---|---|---|
| chr6 | 4903481 | 3 | 1 | chr6 | 4903480 | rs6907963 | A/G |
| chr6 | 5063826 | 7 | 1 | chr6 | 5063824 | rs460440 | C/T |
| chr6 | 5432629 | 3 | 2 | chr6 | 5432628 | rs386501 | C/T |
| chr6 | 6537983 | 2 | 4 | chr6 | 6537982 | rs1073897 | C/T |
| chr6 | 6802182 | 4 | 4 | chr6 | 6802181 | rs12200128 | G/T |
| chr6 | 6856799 | 3 | 2 | chr6 | 6856798 | rs2249359 | A/G |
| chr6 | 7643384 | 2 | 2 | chr6 | 7643382 | rs927415 | C/T |
| chr6 | 10973369 | 3 | 3 | chr6 | 10973368 | rs3843518 | A/G |
| chr6 | 11368316 | 5 | 1 | chr6 | 11368313 | rs57562398 | A/C |
| chr6 | 11503812 | 5 | 2 | chr6 | 11503811 | rs4363034 | A/G |
| chr6 | 11718229 | 10 | 3 | chr6 | 11718227 | rs577824 | C/T |
| chr6 | 12454737 | 4 | 13 | chr6 | 12454736 | rs2095344 | A/G |
| chr6 | 14242572 | 4 | 3 | chr6 | 14242570 | rs853360 | C/T |
| chr6 | 14283383 | 4 | 1 | chr6 | 14283382 | rs853377 | A/G |
| chr6 | 14379653 | 0 | 0 | chr6 | 14379652 | rs9370736 | A/G |
| chr6 | 14524743 | 4 | 4 | chr6 | 14524741 | rs9349940 | A/C |
| chr6 | 15959092 | 1 | 3 | chr6 | 15959091 | rs220926 | A/G |
| chr6 | 15978758 | 5 | 2 | chr6 | 15978756 | rs12209943 | C/T |
| chr6 | 16269097 | 2 | 5 | chr6 | 16269095 | rs3886540 | C/T |
| chr6 | 16313039 | 3 | 2 | chr6 | 16313038 | rs742067 | C/T |
| chr6 | 16493484 | 2 | 7 | chr6 | 16493482 | rs9477104 | A/C |
| chr6 | 17147793 | 7 | 1 | chr6 | 17147791 | rs1322034 | A/G |
| chr6 | 17572666 | 3 | 1 | chr6 | 17572665 | rs9383287 | A/G |
| chr6 | 17589351 | 2 | 3 | chr6 | 17589350 | rs16879756 | A/G |
| chr6 | 20453095 | 3 | 2 | chr6 | 20453094 | rs9356730 | A/G |
| chr6 | 22939626 | 3 | 1 | chr6 | 22939625 | rs1205495 | C/T |
| chr6 | 25261946 | 1 | 3 | chr6 | 25261944 | rs4712889 | C/T |
| chr6 | 25344326 | 1 | 3 | chr6 | 25344325 | rs4626418 | G/T |
| chr6 | 25424481 | 8 | 1 | chr6 | 25424479 | rs2143022 | A/C |
| chr6 | 25504517 | 3 | 1 | chr6 | 25504516 | rs9467485 | A/G |
| chr6 | 25608688 | 2 | 2 | chr6 | 25608687 | rs301393 | G/T |
| chr6 | 27488080 | 3 | 2 | chr6 | 27488079 | rs4711151 | A/G |

| | | | | | | | |
|---|---|---|---|---|---|---|---|
| chr6 | 32635823 | 1 | 4 | chr6 | 32635822 | rs28846812 | A/G |
| chr6 | 33661656 | 6 | 2 | chr6 | 33661654 | rs395671 | C/G |
| chr6 | 33897381 | 4 | 7 | chr6 | 33897379 | rs4713687 | C/T |
| chr6 | 33919426 | 0 | 0 | chr6 | 33919424 | rs13193499 | C/T |
| chr6 | 34071587 | 3 | 2 | chr6 | 34071586 | rs2499770 | A/G |
| chr6 | 34253339 | 2 | 2 | chr6 | 34253338 | rs4713758 | A/G |
| chr6 | 34965432 | 3 | 2 | chr6 | 34965429 | rs2273007 | C/T |
| chr6 | 35226368 | 2 | 6 | chr6 | 35226367 | rs17539666 | A/G |
| chr6 | 35585282 | 1 | 6 | chr6 | 35585280 | rs9296155 | C/T |
| chr6 | 35936240 | 6 | 2 | chr6 | 35936239 | rs17654270 | A/G |
| chr6 | 36206312 | 11 | 1 | chr6 | 36206310 | rs12191295 | C/T |
| chr6 | 36733194 | 3 | 2 | chr6 | 36733193 | rs6907801 | A/G |
| chr6 | 37088618 | 5 | 1 | chr6 | 37088617 | rs1753291 | A/G |
| chr6 | 37286844 | 3 | 2 | chr6 | 37286842 | rs1224328 | C/T |
| chr6 | 39066550 | 5 | 2 | chr6 | 39066549 | rs6921666 | A/G |
| chr6 | 39967864 | 5 | 2 | chr6 | 39967862 | rs3004060 | C/T |
| chr6 | 40206078 | 1 | 4 | chr6 | 40206076 | rs1721413 | A/G |
| chr6 | 40600030 | 3 | 2 | chr6 | 40600029 | rs392641 | G/T |
| chr6 | 41123146 | 1 | 6 | chr6 | 41123144 | rs728477 | C/T |
| chr6 | 41472089 | 4 | 6 | chr6 | 41472087 | rs1969767 | C/T |
| chr6 | 41595280 | 3 | 4 | chr6 | 41595279 | rs2496648 | A/G |
| chr6 | 41602037 | 1 | 3 | chr6 | 41602035 | rs2477845 | C/T |
| chr6 | 42480380 | 1 | 7 | chr6 | 42480379 | rs260285 | A/G |
| chr6 | 42766509 | 1 | 7 | chr6 | 42766507 | rs374036 | C/T |
| chr6 | 43219424 | 3 | 4 | chr6 | 43219422 | rs3737185 | A/G |
| chr6 | 43857142 | 3 | 3 | chr6 | 43857140 | rs3025021 | C/T |
| chr6 | 43901369 | 1 | 6 | chr6 | 43901368 | rs6940798 | A/G |
| chr6 | 43940008 | 11 | 1 | chr6 | 43940007 | rs4714705 | A/G |
| chr6 | 44044453 | 5 | 1 | chr6 | 44044452 | rs9394975 | A/G |
| chr6 | 44127497 | 7 | 1 | chr6 | 44127495 | rs6937694 | C/T |
| chr6 | 44227654 | 7 | 3 | chr6 | 44227653 | rs3734697 | A/G |
| chr6 | 45905834 | 1 | 4 | chr6 | 45905833 | rs10948259 | A/G |

| | | | | | | | |
|---|---|---|---|---|---|---|---|
| chr6 | 47874659 | 1 | 5 | chr6 | 47874656 | rs9473187 | A/C |
| chr6 | 48049223 | 2 | 2 | chr6 | 48049222 | rs2814478 | C/T |
| chr6 | 50163126 | 5 | 1 | chr6 | 50163124 | rs586485 | C/T |
| chr6 | 50800310 | 5 | 1 | chr6 | 50800309 | rs10948573 | C/G |
| chr6 | 51062030 | 1 | 6 | chr6 | 51062029 | rs283579 | A/G |
| chr6 | 51706365 | 3 | 1 | chr6 | 51706364 | rs1256000 | C/T |
| chr6 | 51857109 | 3 | 5 | chr6 | 51857108 | rs4715256 | A/G |
| chr6 | 54712476 | 2 | 10 | chr6 | 54712475 | rs10948847 | A/G |
| chr6 | 56240797 | 4 | 3 | chr6 | 56240795 | rs6940268 | C/T |
| chr6 | 57327678 | 1 | 3 | chr6 | 57327676 | rs9296868 | C/T |
| chr6 | 57436879 | 1 | 3 | chr6 | 57436878 | rs35233178 | C/T |
| chr6 | 57487789 | 6 | 6 | chr6 | 57487787 | rs7747396 | C/T |
| chr6 | 57548778 | 1 | 8 | chr6 | 57548777 | rs477006 | A/G |
| chr6 | 57550717 | 5 | 2 | chr6 | 57550716 | rs536923 | A/G |
| chr6 | 58884696 | 6 | 1 | chr6 | 58884693 | rs9717243 | C/T |
| chr6 | 58884696 | 6 | 1 | chr6 | 58884694 | rs4928539 | A/G |
| chr6 | 58884696 | 6 | 1 | chr6 | 58884695 | rs57096886 | C/G |
| chr6 | 58884696 | 6 | 1 | chr6 | 58884696 | rs9404798 | G/T |
| chr6 | 58884696 | 6 | 1 | chr6 | 58884696 | rs59506742 | -/G |
| chr6 | 62369628 | 2 | 5 | chr6 | 62369626 | rs7740039 | C/T |
| chr6 | 62369628 | 2 | 5 | chr6 | 62369626 | rs71539291 | CA/TG |
| chr6 | 62369628 | 2 | 5 | chr6 | 62369627 | rs6453781 | A/G |
| chr6 | 63459005 | 1 | 5 | chr6 | 63459003 | rs6939538 | C/T |
| chr6 | 66653536 | 3 | 4 | chr6 | 66653535 | rs2211636 | A/G |
| chr6 | 67114876 | 4 | 1 | chr6 | 67114874 | rs851857 | A/G |
| chr6 | 67698021 | 3 | 2 | chr6 | 67698020 | rs6912709 | A/G |
| chr6 | 67986125 | 2 | 2 | chr6 | 67986123 | rs1074916 | G/T |
| chr6 | 68517188 | 6 | 5 | chr6 | 68517186 | rs4280950 | C/T |
| chr6 | 70429710 | 4 | 2 | chr6 | 70429708 | rs7752102 | C/T |
| chr6 | 70802971 | 1 | 5 | chr6 | 70802969 | rs10945192 | C/T |
| chr6 | 71384012 | 2 | 4 | chr6 | 71384011 | rs2691476 | A/G |
| chr6 | 71966932 | 4 | 5 | chr6 | 71966931 | rs4502887 | C/G |

| | | | | | | | |
|---|---|---|---|---|---|---|---|
| chr6 | 73083199 | 1 | 3 | chr6 | 73083198 | rs2807503 | C/T |
| chr6 | 73483064 | 3 | 1 | chr6 | 73483062 | rs73537781 | A/C |
| chr6 | 73918396 | 1 | 5 | chr6 | 73918393 | rs72951081 | A/C |
| chr6 | 73918396 | 1 | 5 | chr6 | 73918394 | rs4235874 | C/T |
| chr6 | 74295329 | 4 | 1 | chr6 | 74295328 | rs9352005 | A/G |
| chr6 | 75580697 | 1 | 4 | chr6 | 75580695 | rs7760092 | C/T |
| chr6 | 76635386 | 3 | 2 | chr6 | 76635385 | rs3798435 | A/C |
| chr6 | 77149329 | 5 | 4 | chr6 | 77149328 | rs2451543 | A/G |
| chr6 | 77708859 | 3 | 1 | chr6 | 77708858 | rs4472308 | A/G |
| chr6 | 79346824 | 1 | 5 | chr6 | 79346823 | rs484582 | G/T |
| chr6 | 80036468 | 4 | 1 | chr6 | 80036467 | rs4706765 | A/G |
| chr6 | 80738942 | 1 | 4 | chr6 | 80738942 | rs151652 | C/G |
| chr6 | 80770933 | 1 | 3 | chr6 | 80770931 | rs465480 | C/G |
| chr6 | 83323218 | 6 | 1 | chr6 | 83323217 | rs3011875 | A/G |
| chr6 | 84577190 | 2 | 10 | chr6 | 84577189 | rs1570842 | C/G |
| chr6 | 85145982 | 4 | 6 | chr6 | 85145981 | rs9449886 | A/G |
| chr6 | 85533019 | 10 | 13 | chr6 | 85533018 | rs215931 | A/G |
| chr6 | 86243194 | 5 | 4 | chr6 | 86243193 | rs6454469 | A/G |
| chr6 | 88522525 | 2 | 3 | chr6 | 88522524 | rs4707400 | C/G |
| chr6 | 88522525 | 2 | 3 | chr6 | 88522524 | rs4707401 | CT/G/GG/T |
| chr6 | 88522525 | 2 | 3 | chr6 | 88522525 | rs72906564 | G/T |
| chr6 | 90030783 | 1 | 3 | chr6 | 90030781 | rs723041 | C/T |
| chr6 | 91165240 | 5 | 2 | chr6 | 91165239 | rs9444762 | A/G |
| chr6 | 91918507 | 7 | 2 | chr6 | 91918506 | rs4269348 | A/G |
| chr6 | 93900309 | 3 | 1 | chr6 | 93900308 | rs9353987 | A/G |
| chr6 | 94738860 | 4 | 1 | chr6 | 94738858 | rs1488313 | C/T |
| chr6 | 96207520 | 2 | 3 | chr6 | 96207518 | rs9375156 | C/T |
| chr6 | 96707570 | 13 | 3 | chr6 | 96707568 | rs12191957 | C/T |
| chr6 | 96707570 | 13 | 3 | chr6 | 96707569 | rs4240598 | A/G |
| chr6 | 96742826 | 2 | 3 | chr6 | 96742824 | rs4240600 | C/T |
| chr6 | 96760211 | 2 | 3 | chr6 | 96760209 | rs4132773 | A/G |
| chr6 | 96794016 | 7 | 4 | chr6 | 96794014 | rs6919901 | C/T |

| | | | | | | | |
|---|---|---|---|---|---|---|---|
| chr6 | 98329815 | 3 | 1 | chr6 | 98329813 | rs72926948 | C/T |
| chr6 | 98446490 | 1 | 6 | chr6 | 98446489 | rs6569153 | A/G |
| chr6 | 98446703 | 4 | 2 | chr6 | 98446701 | rs6902595 | C/T |
| chr6 | 98447658 | 6 | 2 | chr6 | 98447656 | rs7756014 | C/T |
| chr6 | 99309944 | 6 | 2 | chr6 | 99309942 | rs4406241 | C/T |
| chr6 | 101997113 | 2 | 3 | chr6 | 101997112 | rs2788274 | A/G |
| chr6 | 102431866 | 2 | 2 | chr6 | 102431865 | rs9390791 | A/G |
| chr6 | 102539562 | 2 | 2 | chr6 | 102539561 | rs1360790 | A/G |
| chr6 | 103464854 | 4 | 1 | chr6 | 103464852 | rs9390917 | C/T |
| chr6 | 104940718 | 4 | 3 | chr6 | 104940716 | rs13220571 | C/T |
| chr6 | 104940718 | 4 | 3 | chr6 | 104940717 | rs13205781 | A/CA/G/TG |
| chr6 | 104940718 | 4 | 3 | chr6 | 104940717 | rs67142071 | CA/TG |
| chr6 | 105086206 | 3 | 2 | chr6 | 105086205 | rs1934061 | A/G |
| chr6 | 105656719 | 0 | 0 | chr6 | 105656717 | rs221658 | G/T |
| chr6 | 105659443 | 2 | 7 | chr6 | 105659442 | rs221662 | A/G |
| chr6 | 105660604 | 4 | 2 | chr6 | 105660601 | rs455114 | C/G |
| chr6 | 105660604 | 4 | 2 | chr6 | 105660601 | rs71549429 | CC/GT |
| chr6 | 105660604 | 4 | 2 | chr6 | 105660602 | rs414074 | A/G |
| chr6 | 106698344 | 2 | 2 | chr6 | 106698342 | rs11152967 | C/T |
| chr6 | 106986623 | 2 | 3 | chr6 | 106986622 | rs1884461 | C/T |
| chr6 | 107003028 | 1 | 4 | chr6 | 107003026 | rs4492224 | C/T |
| chr6 | 107087549 | 5 | 3 | chr6 | 107087547 | rs6908538 | C/T |
| chr6 | 107087549 | 5 | 3 | chr6 | 107087548 | rs6928874 | A/G |
| chr6 | 107783660 | 2 | 2 | chr6 | 107783658 | rs72939877 | C/T |
| chr6 | 108062787 | 2 | 8 | chr6 | 108062787 | rs9486659 | A/G |
| chr6 | 109133041 | 2 | 5 | chr6 | 109133039 | rs1268155 | A/G |
| chr6 | 110990456 | 1 | 11 | chr6 | 110990455 | rs2462483 | A/G |
| chr6 | 112683648 | 3 | 1 | chr6 | 112683647 | rs4571602 | A/G |
| chr6 | 112770442 | 2 | 4 | chr6 | 112770441 | rs6900370 | A/G |
| chr6 | 113286022 | 2 | 4 | chr6 | 113286020 | rs58149916 | C/T |
| chr6 | 114193233 | 19 | 4 | chr6 | 114193231 | rs9400669 | C/T |
| chr6 | 114301216 | 3 | 1 | chr6 | 114301215 | rs11153451 | A/G |

| | | | | | | | |
|---|---|---|---|---|---|---|---|
| chr6 | 116831070 | 2 | 2 | chr6 | 116831069 | rs1029313 | C/G |
| chr6 | 117379993 | 3 | 4 | chr6 | 117379991 | rs2353358 | C/T |
| chr6 | 117660991 | 11 | 1 | chr6 | 117660990 | rs7770292 | A/G |
| chr6 | 117660991 | 11 | 1 | chr6 | 117660991 | rs2781137 | G/T |
| chr6 | 118554019 | 2 | 2 | chr6 | 118554018 | rs4512262 | G/T |
| chr6 | 122801048 | 4 | 1 | chr6 | 122801047 | rs577049 | A/G |
| chr6 | 123248031 | 16 | 1 | chr6 | 123248029 | rs7758880 | C/T |
| chr6 | 124514490 | 2 | 5 | chr6 | 124514488 | rs802248 | C/T |
| chr6 | 124625631 | 5 | 1 | chr6 | 124625630 | rs2753141 | A/G |
| chr6 | 131295666 | 3 | 1 | chr6 | 131295665 | rs7766004 | A/G |
| chr6 | 133006529 | 7 | 1 | chr6 | 133006527 | rs7764614 | C/T |
| chr6 | 133006529 | 7 | 1 | chr6 | 133006528 | rs7764501 | A/G |
| chr6 | 133583857 | 1 | 4 | chr6 | 133583856 | rs4895957 | A/G |
| chr6 | 136720242 | 3 | 1 | chr6 | 136720241 | rs4640907 | A/G |
| chr6 | 136720242 | 3 | 1 | chr6 | 136720242 | rs71576394 | A/G |
| chr6 | 137475898 | 1 | 5 | chr6 | 137475897 | rs276574 | A/G |
| chr6 | 139455457 | 4 | 1 | chr6 | 139455454 | rs72976606 | C/T |
| chr6 | 139458433 | 3 | 4 | chr6 | 139458432 | rs62439984 | A/G |
| chr6 | 143058384 | 8 | 4 | chr6 | 143058383 | rs571637 | G/T |
| chr6 | 147529278 | 1 | 5 | chr6 | 147529276 | rs1221645 | C/T |
| chr6 | 147938147 | 1 | 3 | chr6 | 147938145 | rs55647502 | C/T |
| chr6 | 148059422 | 1 | 4 | chr6 | 148059421 | rs9399617 | A/C/G |
| chr6 | 148059422 | 1 | 4 | chr6 | 148059423 | rs35111633 | -/G |
| chr6 | 148350473 | 1 | 3 | chr6 | 148350471 | rs36056323 | A/C |
| chr6 | 148456342 | 2 | 2 | chr6 | 148456341 | rs2328851 | A/G |
| chr6 | 148748900 | 2 | 7 | chr6 | 148748898 | rs9498024 | C/T |
| chr6 | 149341000 | 1 | 3 | chr6 | 149340999 | rs6924147 | A/G |
| chr6 | 149377777 | 2 | 5 | chr6 | 149377775 | rs2500547 | C/T |
| chr6 | 149377777 | 2 | 5 | chr6 | 149377776 | rs57955571 | G/T |
| chr6 | 149377777 | 2 | 5 | chr6 | 149377778 | rs34512795 | -/G |
| chr6 | 149425572 | 4 | 5 | chr6 | 149425571 | rs13190879 | A/G |
| chr6 | 150678341 | 4 | 9 | chr6 | 150678339 | rs4870464 | C/T |

| | | | | | | | |
|---|---|---|---|---|---|---|---|
| chr6 | 150936332 | 1 | 3 | chr6 | 150936330 | rs9322266 | C/T |
| chr6 | 150967915 | 1 | 3 | chr6 | 150967915 | rs2029645 | G/T |
| chr6 | 151704852 | 7 | 3 | chr6 | 151704851 | rs6901885 | C/G |
| chr6 | 152020740 | 1 | 4 | chr6 | 152020739 | rs10484920 | A/G |
| chr6 | 152843991 | 4 | 1 | chr6 | 152843989 | rs7763880 | C/T |
| chr6 | 154262907 | 2 | 5 | chr6 | 154262906 | rs581564 | A/G |
| chr6 | 155357248 | 2 | 2 | chr6 | 155357247 | rs13199643 | A/G |
| chr6 | 155534245 | 11 | 6 | chr6 | 155534244 | rs2151959 | A/G |
| chr6 | 155602186 | 8 | 4 | chr6 | 155602185 | rs3935936 | A/G |
| chr6 | 155638594 | 2 | 4 | chr6 | 155638592 | rs927718 | C/T |
| chr6 | 156069412 | 5 | 2 | chr6 | 156069411 | rs7764540 | A/G |
| chr6 | 156122070 | 2 | 4 | chr6 | 156122069 | rs2647710 | A/G |
| chr6 | 156178381 | 2 | 2 | chr6 | 156178379 | rs13213139 | C/T |
| chr6 | 156417541 | 3 | 3 | chr6 | 156417541 | rs9384419 | A/G |
| chr6 | 156530247 | 2 | 5 | chr6 | 156530245 | rs62434812 | C/T |
| chr6 | 156915017 | 1 | 4 | chr6 | 156915015 | rs577148 | A/G |
| chr6 | 156915017 | 1 | 4 | chr6 | 156915016 | rs672626 | A/G |
| chr6 | 158363141 | 1 | 3 | chr6 | 158363139 | rs12206632 | C/G |
| chr6 | 158401982 | 6 | 13 | chr6 | 158401981 | rs189482 | A/G |
| chr6 | 159093840 | 2 | 2 | chr6 | 159093839 | rs3123095 | A/G |
| chr6 | 159254667 | 1 | 3 | chr6 | 159254666 | rs73015544 | C/G |
| chr6 | 159434242 | 2 | 5 | chr6 | 159434240 | rs1994564 | C/T |
| chr6 | 159478986 | 1 | 4 | chr6 | 159478984 | rs2451265 | A/G |
| chr6 | 160274792 | 2 | 2 | chr6 | 160274791 | rs6939644 | A/G |
| chr6 | 160414399 | 8 | 3 | chr6 | 160414398 | rs629849 | A/G |
| chr6 | 161647174 | 2 | 2 | chr6 | 161647172 | rs9347491 | C/T |
| chr6 | 163127716 | 4 | 7 | chr6 | 163127714 | rs9458645 | C/T |
| chr6 | 163311399 | 2 | 5 | chr6 | 163311397 | rs9355416 | C/T |
| chr6 | 163540400 | 4 | 2 | chr6 | 163540398 | rs6917118 | A/C |
| chr6 | 163688771 | 4 | 5 | chr6 | 163688771 | rs878423 | C/G |
| chr6 | 163696180 | 3 | 3 | chr6 | 163696179 | rs9365560 | A/G |
| chr6 | 163944094 | 5 | 7 | chr6 | 163944092 | rs10945902 | -/C/G |

| | | | | | | | |
|---|---|---|---|---|---|---|---|
| chr6 | 163944094 | 5 | 7 | chr6 | 163944092 | rs63601760 | C/G |
| chr6 | 163944094 | 5 | 7 | chr6 | 163944093 | rs10945903 | C/G |
| chr6 | 164628779 | 4 | 8 | chr6 | 164628777 | rs794122 | A/G |
| chr6 | 165263206 | 5 | 1 | chr6 | 165263205 | rs1322188 | C/T |
| chr6 | 165911574 | 6 | 1 | chr6 | 165911574 | rs1410480 | A/G |
| chr6 | 166615185 | 3 | 1 | chr6 | 166615183 | rs2064879 | C/T |
| chr6 | 166695668 | 3 | 1 | chr6 | 166695666 | rs7752716 | C/T |
| chr6 | 166742786 | 3 | 1 | chr6 | 166742785 | rs9459664 | A/G |
| chr6 | 166794070 | 3 | 2 | chr6 | 166794069 | rs9355582 | G/T |
| chr6 | 166899037 | 2 | 3 | chr6 | 166899035 | rs3778401 | A/G |
| chr6 | 166906757 | 1 | 5 | chr6 | 166906756 | rs11755586 | A/G |
| chr6 | 167436360 | 4 | 2 | chr6 | 167436358 | rs2039320 | A/G |
| chr6 | 167504638 | 3 | 3 | chr6 | 167504636 | rs9457322 | G/T |
| chr6 | 167504638 | 3 | 3 | chr6 | 167504636 | rs62436851 | A/C |
| chr6 | 167662337 | 4 | 3 | chr6 | 167662336 | rs4709170 | A/G |
| chr6 | 167670168 | 7 | 2 | chr6 | 167670166 | rs3010556 | C/G |
| chr6 | 167717422 | 3 | 3 | chr6 | 167717421 | rs9457322 | G/T |
| chr6 | 167947572 | 4 | 2 | chr6 | 167947571 | rs550392 | C/T |
| chr6 | 168209041 | 1 | 3 | chr6 | 168209040 | rs9295040 | A/G |
| chr6 | 168258524 | 12 | 4 | chr6 | 168258522 | rs4708435 | C/T |
| chr6 | 168484410 | 1 | 3 | chr6 | 168484408 | rs9456064 | C/T |
| chr6 | 168508743 | 1 | 4 | chr6 | 168508742 | rs942543 | C/T |
| chr6 | 168509437 | 1 | 3 | chr6 | 168509435 | rs73038303 | C/G |
| chr6 | 168721340 | 4 | 2 | chr6 | 168721338 | rs7751540 | C/T |
| chr6 | 168721340 | 4 | 2 | chr6 | 168721339 | rs7762332 | A/G |
| chr6 | 169033226 | 2 | 4 | chr6 | 169033225 | rs7751281 | A/G |
| chr6 | 169056380 | 3 | 9 | chr6 | 169056378 | rs4708553 | A/C |
| chr6 | 169085008 | 4 | 6 | chr6 | 169085006 | rs4708560 | C/T |
| chr6 | 169165706 | 5 | 6 | chr6 | 169165704 | rs9346501 | C/T |
| chr6 | 169362173 | 3 | 3 | chr6 | 169362171 | rs9393165 | A/G |
| chr6 | 169991814 | 1 | 3 | chr6 | 169991813 | rs6912144 | A/G |
| chr6 | 170192950 | 5 | 1 | chr6 | 170192949 | rs6932737 | A/G |

| | | | | | | | |
|---|---|---|---|---|---|---|---|
| chr6 | 170206226 | 2 | 4 | chr6 | 170206224 | rs9348317 | C/T |
| chr6 | 170235198 | 5 | 4 | chr6 | 170235196 | rs6456233 | C/T |
| chr6 | 170305940 | 2 | 4 | chr6 | 170305939 | rs3012374 | A/G |
| chr6 | 170314116 | 7 | 3 | chr6 | 170314114 | rs2935094 | A/G |
| chr6 | 170319135 | 0 | 0 | chr6 | 170319134 | rs3013290 | G/T |
| chr6 | 170319135 | 0 | 0 | chr6 | 170319136 | rs2935053 | C/T |
| chr6 | 170372011 | 3 | 2 | chr6 | 170372008 | rs62424301 | C/G |
| chr6 | 170372011 | 3 | 2 | chr6 | 170372008 | rs67846384 | -/G |
| chr6 | 170591167 | 3 | 2 | chr6 | 170591166 | rs4710827 | A/G |
| chr7 | 158577 | 2 | 4 | chr7 | 158576 | rs6964622 | A/G |
| chr7 | 179784 | 1 | 3 | chr7 | 179782 | rs12718068 | C/T |
| chr7 | 186866 | 4 | 2 | chr7 | 186864 | rs9642262 | C/T |
| chr7 | 256025 | 5 | 1 | chr7 | 256023 | rs13309533 | A/C |
| chr7 | 636938 | 6 | 1 | chr7 | 636937 | rs9690480 | A/G |
| chr7 | 681433 | 2 | 7 | chr7 | 681431 | rs6970378 | C/T |
| chr7 | 860043 | 2 | 5 | chr7 | 860042 | rs4419711 | A/G |
| chr7 | 863178 | 5 | 2 | chr7 | 863176 | rs9655181 | A/C |
| chr7 | 925127 | 3 | 3 | chr7 | 925126 | rs12668016 | A/G |
| chr7 | 1087445 | 1 | 4 | chr7 | 1087443 | rs4724104 | C/T |
| chr7 | 1185824 | 4 | 1 | chr7 | 1185821 | rs6950714 | A/C |
| chr7 | 1275734 | 3 | 2 | chr7 | 1275733 | rs2056838 | A/G |
| chr7 | 1352429 | 2 | 2 | chr7 | 1352426 | rs10155805 | C/T |
| chr7 | 1474716 | 0 | 0 | chr7 | 1474716 | rs11972070 | G/T |
| chr7 | 1490086 | 8 | 4 | chr7 | 1490084 | rs10235180 | C/T |
| chr7 | 1495210 | 1 | 10 | chr7 | 1495209 | rs10230463 | A/G |
| chr7 | 1509341 | 7 | 4 | chr7 | 1509339 | rs3752715 | C/T |
| chr7 | 1519899 | 4 | 2 | chr7 | 1519898 | rs10952143 | A/G |
| chr7 | 1815799 | 1 | 5 | chr7 | 1815797 | rs4351332 | C/T |
| chr7 | 1829691 | 2 | 6 | chr7 | 1829689 | rs9770241 | C/T |
| chr7 | 1852859 | 1 | 4 | chr7 | 1852858 | rs10234557 | A/G |
| chr7 | 1856126 | 1 | 3 | chr7 | 1856124 | rs10250550 | C/T |
| chr7 | 1875679 | 1 | 10 | chr7 | 1875678 | rs10950411 | A/G |

| | | | | | | | |
|---|---|---|---|---|---|---|---|
| chr7 | 1914098 | 1 | 3 | chr7 | 1914096 | rs6461004 | C/T |
| chr7 | 1954950 | 2 | 8 | chr7 | 1954949 | rs10807766 | A/G |
| chr7 | 1958322 | 3 | 8 | chr7 | 1958321 | rs6961018 | A/G |
| chr7 | 1970252 | 10 | 2 | chr7 | 1970250 | rs4721253 | -/C/T |
| chr7 | 1970252 | 10 | 2 | chr7 | 1970250 | rs56198132 | C/T |
| chr7 | 2022540 | 6 | 5 | chr7 | 2022539 | rs939945 | A/G |
| chr7 | 2022999 | 3 | 19 | chr7 | 2022997 | rs939947 | A/C |
| chr7 | 2156912 | 2 | 3 | chr7 | 2156910 | rs1637770 | A/G |
| chr7 | 2280134 | 2 | 2 | chr7 | 2280132 | rs7456643 | C/T |
| chr7 | 2656000 | 4 | 1 | chr7 | 2655997 | rs3801077 | G/T |
| chr7 | 2715285 | 4 | 1 | chr7 | 2715283 | rs59438885 | C/G |
| chr7 | 2746318 | 3 | 4 | chr7 | 2746317 | rs798515 | C/T |
| chr7 | 3023422 | 4 | 3 | chr7 | 3023421 | rs62439326 | A/G |
| chr7 | 3095316 | 2 | 4 | chr7 | 3095314 | rs4722404 | C/T |
| chr7 | 3286769 | 5 | 3 | chr7 | 3286767 | rs10240575 | C/T |
| chr7 | 3286769 | 5 | 3 | chr7 | 3286768 | rs73045613 | C/G |
| chr7 | 3325258 | 4 | 4 | chr7 | 3325256 | rs4298408 | C/T |
| chr7 | 4146279 | 1 | 3 | chr7 | 4146277 | rs660802 | A/G |
| chr7 | 4178244 | 1 | 7 | chr7 | 4178243 | rs631405 | C/T |
| chr7 | 4274599 | 1 | 3 | chr7 | 4274598 | rs1562539 | C/T |
| chr7 | 4275724 | 2 | 4 | chr7 | 4275722 | rs10242102 | C/G |
| chr7 | 4337296 | 8 | 5 | chr7 | 4337294 | rs6462754 | C/T |
| chr7 | 4719166 | 2 | 5 | chr7 | 4719165 | rs4132809 | C/T |
| chr7 | 4728669 | 7 | 2 | chr7 | 4728668 | rs7781456 | A/G |
| chr7 | 4807997 | 4 | 3 | chr7 | 4807995 | rs414035 | A/G |
| chr7 | 5525862 | 3 | 1 | chr7 | 5525861 | rs2537619 | C/T |
| chr7 | 5843843 | 9 | 5 | chr7 | 5843842 | rs7784978 | A/G |
| chr7 | 6032988 | 7 | 2 | chr7 | 6032986 | rs2639 | C/T |
| chr7 | 6552895 | 7 | 4 | chr7 | 6552893 | rs4720679 | C/T |
| chr7 | 7821066 | 2 | 2 | chr7 | 7821065 | rs4725035 | A/G |
| chr7 | 8450727 | 2 | 2 | chr7 | 8450727 | rs12702751 | A/G |
| chr7 | 9071977 | 3 | 2 | chr7 | 9071975 | rs34522817 | C/T |

| | | | | | | | |
|---|---|---|---|---|---|---|---|
| chr7 | 9585906 | 7 | 1 | chr7 | 9585904 | rs2259306 | C/T |
| chr7 | 9601132 | 1 | 4 | chr7 | 9601131 | rs4549691 | A/G |
| chr7 | 9857812 | 5 | 1 | chr7 | 9857811 | rs1514880 | A/G |
| chr7 | 10483925 | 4 | 5 | chr7 | 10483923 | rs12673009 | A/C |
| chr7 | 11500844 | 3 | 2 | chr7 | 11500843 | rs2189311 | A/G |
| chr7 | 12048450 | 4 | 3 | chr7 | 12048449 | rs13238747 | A/G |
| chr7 | 12205800 | 1 | 4 | chr7 | 12205798 | rs17165701 | C/T |
| chr7 | 12837009 | 1 | 4 | chr7 | 12837008 | rs10236747 | A/G |
| chr7 | 13445410 | 5 | 6 | chr7 | 13445409 | rs969197 | C/G |
| chr7 | 15606103 | 6 | 1 | chr7 | 15606101 | rs6461193 | C/T |
| chr7 | 16730606 | 3 | 1 | chr7 | 16730604 | rs6959312 | C/T |
| chr7 | 16760029 | 4 | 1 | chr7 | 16760028 | rs6975847 | A/G |
| chr7 | 17173283 | 1 | 7 | chr7 | 17173280 | rs10950648 | C/T |
| chr7 | 18331473 | 4 | 3 | chr7 | 18331472 | rs584781 | A/G |
| chr7 | 18955757 | 2 | 6 | chr7 | 18955756 | rs2051920 | C/T |
| chr7 | 19495547 | 1 | 3 | chr7 | 19495546 | rs2080223 | C/T |
| chr7 | 19767068 | 3 | 5 | chr7 | 19767066 | rs57916046 | C/T |
| chr7 | 21525566 | 2 | 2 | chr7 | 21525564 | rs6461572 | C/T |
| chr7 | 22844629 | 5 | 1 | chr7 | 22844628 | rs12535331 | G/T |
| chr7 | 22968179 | 4 | 6 | chr7 | 22968177 | rs73082381 | C/T |
| chr7 | 23577155 | 4 | 1 | chr7 | 23577153 | rs227951 | C/T |
| chr7 | 24758420 | 4 | 2 | chr7 | 24758418 | rs2248149 | C/T |
| chr7 | 25411938 | 1 | 4 | chr7 | 25411936 | rs6461865 | C/T |
| chr7 | 25581109 | 5 | 4 | chr7 | 25581108 | rs73085920 | A/G |
| chr7 | 25581109 | 5 | 4 | chr7 | 25581109 | rs2813890 | A/G |
| chr7 | 25585686 | 5 | 1 | chr7 | 25585684 | rs208544 | C/G |
| chr7 | 26678940 | 1 | 5 | chr7 | 26678939 | rs17290728 | G/T |
| chr7 | 27171806 | 4 | 2 | chr7 | 27171806 | rs3801776 | A/G |
| chr7 | 27529764 | 5 | 1 | chr7 | 27529762 | rs6462027 | C/T |
| chr7 | 27529764 | 5 | 1 | chr7 | 27529763 | rs73686421 | A/G |
| chr7 | 27862601 | 12 | 1 | chr7 | 27862598 | rs6462056 | C/G |
| chr7 | 28115558 | 4 | 5 | chr7 | 28115557 | rs10281935 | A/G |

| | | | | | | | |
|---|---|---|---|---|---|---|---|
| chr7 | 28183332 | 1 | 3 | chr7 | 28183331 | rs10272119 | A/G |
| chr7 | 28227063 | 3 | 6 | chr7 | 28227062 | rs10249019 | A/G |
| chr7 | 28394917 | 3 | 3 | chr7 | 28394915 | rs7794304 | C/T |
| chr7 | 28518621 | 6 | 2 | chr7 | 28518619 | rs2391669 | C/T |
| chr7 | 28564166 | 4 | 5 | chr7 | 28564164 | rs216737 | C/T |
| chr7 | 29199970 | 1 | 7 | chr7 | 29199969 | rs39050 | A/G |
| chr7 | 30154457 | 6 | 1 | chr7 | 30154456 | rs10268447 | A/G |
| chr7 | 30706216 | 3 | 1 | chr7 | 30706215 | rs107540 | A/G |
| chr7 | 32786738 | 1 | 5 | chr7 | 32786736 | rs1811486 | C/T |
| chr7 | 33107829 | 2 | 2 | chr7 | 33107828 | rs6964595 | A/G |
| chr7 | 33728596 | 2 | 3 | chr7 | 33728595 | rs2392256 | A/G |
| chr7 | 33900564 | 4 | 1 | chr7 | 33900563 | rs7809969 | A/G |
| chr7 | 34106159 | 4 | 2 | chr7 | 34106158 | rs1362459 | A/G |
| chr7 | 34119659 | 6 | 2 | chr7 | 34119658 | rs2058678 | A/G |
| chr7 | 34122338 | 1 | 6 | chr7 | 34122337 | rs4723351 | G/T |
| chr7 | 34874312 | 4 | 1 | chr7 | 34874310 | rs1833090 | A/C |
| chr7 | 35338565 | 3 | 1 | chr7 | 35338564 | rs235400 | C/T |
| chr7 | 37528892 | 5 | 1 | chr7 | 37528892 | rs1425132 | A/G |
| chr7 | 38077152 | 4 | 3 | chr7 | 38077150 | rs1524068 | A/G |
| chr7 | 38761451 | 8 | 3 | chr7 | 38761450 | rs4720300 | A/G |
| chr7 | 38910076 | 4 | 2 | chr7 | 38910074 | rs6976302 | A/C |
| chr7 | 39323524 | 4 | 2 | chr7 | 39323522 | rs4723857 | C/T |
| chr7 | 39498514 | 4 | 1 | chr7 | 39498512 | rs17620176 | C/T |
| chr7 | 39712992 | 4 | 1 | chr7 | 39712990 | rs4723892 | C/T |
| chr7 | 39927634 | 5 | 1 | chr7 | 39927632 | rs1533943 | A/G |
| chr7 | 41041913 | 2 | 2 | chr7 | 41041911 | rs10280625 | C/T |
| chr7 | 41769976 | 1 | 7 | chr7 | 41769974 | rs6975680 | C/T |
| chr7 | 42065501 | 4 | 5 | chr7 | 42065500 | rs917229 | C/T |
| chr7 | 42199132 | 3 | 3 | chr7 | 42199131 | rs62443811 | A/G |
| chr7 | 42468110 | 2 | 3 | chr7 | 42468109 | rs2877149 | A/G |
| chr7 | 42756850 | 2 | 5 | chr7 | 42756849 | rs2583872 | G/T |
| chr7 | 42771406 | 1 | 3 | chr7 | 42771405 | rs2583889 | C/T |

| | | | | | | | | |
|---|---|---|---|---|---|---|---|---|
| chr7 | 43233013 | 2 | 3 | chr7 | 43233012 | rs12702026 | | A/G |
| chr7 | 44066190 | 3 | 1 | chr7 | 44066188 | rs11552797 | | C/T |
| chr7 | 44068563 | 6 | 4 | chr7 | 44068561 | rs11767165 | | C/T |
| chr7 | 44102100 | 1 | 3 | chr7 | 44102099 | rs11768607 | | A/G |
| chr7 | 44247473 | 0 | 0 | chr7 | 44247472 | rs2075074 | | C/T |
| chr7 | 44301777 | 9 | 6 | chr7 | 44301775 | rs62459119 | | C/T |
| chr7 | 45161678 | 3 | 1 | chr7 | 45161677 | rs56178166 | | A/G |
| chr7 | 46333353 | 9 | 6 | chr7 | 46333351 | rs10278621 | | C/T |
| chr7 | 47454139 | 8 | 2 | chr7 | 47454137 | rs940857 | | C/T |
| chr7 | 47487376 | 2 | 2 | chr7 | 47487374 | rs1543035 | | C/T |
| chr7 | 47505072 | 11 | 2 | chr7 | 47505070 | rs2271311 | | C/T |
| chr7 | 47505072 | 11 | 2 | chr7 | 47505071 | rs2271312 | | A/G |
| chr7 | 47577670 | 5 | 5 | chr7 | 47577669 | rs4551248 | | A/G |
| chr7 | 48038297 | 4 | 2 | chr7 | 48038296 | rs12537979 | | A/G |
| chr7 | 49427613 | 1 | 5 | chr7 | 49427612 | rs13228770 | | A/G |
| chr7 | 49938864 | 1 | 5 | chr7 | 49938863 | rs11978548 | | A/G |
| chr7 | 50111615 | 2 | 4 | chr7 | 50111613 | rs4917116 | | C/T |
| chr7 | 50129435 | 5 | 3 | chr7 | 50129434 | rs1379173 | | A/G |
| chr7 | 50395528 | 7 | 15 | chr7 | 50395527 | rs6592967 | | A/G |
| chr7 | 50574700 | 4 | 3 | chr7 | 50574699 | rs1466163 | | C/T |
| chr7 | 50680621 | 5 | 1 | chr7 | 50680619 | rs10464788 | | C/T |
| chr7 | 51168411 | 4 | 1 | chr7 | 51168410 | rs1370403 | | A/G |
| chr7 | 51490852 | 6 | 5 | chr7 | 51490850 | rs1320414 | | A/G |
| chr7 | 53164055 | 1 | 4 | chr7 | 53164054 | rs28469327 | | A/G |
| chr7 | 55475894 | 2 | 2 | chr7 | 55475892 | rs1723875 | | A/G |
| chr7 | 55521650 | 2 | 4 | chr7 | 55521648 | rs13241056 | | C/G |
| chr7 | 55605381 | 2 | 3 | chr7 | 55605380 | rs6976995 | | A/G |
| chr7 | 55719306 | 1 | 3 | chr7 | 55719304 | rs62455748 | | C/T |
| chr7 | 56209938 | 3 | 1 | chr7 | 56209935 | rs2430222 | | C/T |
| chr7 | 56262284 | 2 | 4 | chr7 | 56262282 | rs11238399 | | C/T |
| chr7 | 57328699 | 6 | 1 | chr7 | 57328696 | rs7792593 | | C/G |
| chr7 | 57402889 | 1 | 4 | chr7 | 57402888 | rs1916774 | | A/G |

| | | | | | | | |
|---|---|---|---|---|---|---|---|
| chr7 | 57477486 | 4 | 3 | chr7 | 57477485 | rs12531446 | A/G |
| chr7 | 62194334 | 4 | 2 | chr7 | 62194333 | rs4302702 | A/G |
| chr7 | 62845858 | 2 | 7 | chr7 | 62845857 | rs2952510 | A/G |
| chr7 | 63784450 | 5 | 3 | chr7 | 63784449 | rs10263594 | A/G |
| chr7 | 63966199 | 3 | 1 | chr7 | 63966198 | rs4718151 | A/G |
| chr7 | 63987222 | 1 | 6 | chr7 | 63987221 | rs4718160 | A/G |
| chr7 | 65073572 | 3 | 1 | chr7 | 65073570 | rs4718296 | A/C |
| chr7 | 66746736 | 2 | 2 | chr7 | 66746734 | rs4718604 | C/T |
| chr7 | 67029853 | 1 | 3 | chr7 | 67029852 | rs17144952 | A/G |
| chr7 | 67112636 | 2 | 2 | chr7 | 67112635 | rs4349877 | A/G |
| chr7 | 67357155 | 7 | 6 | chr7 | 67357154 | rs3108033 | A/G |
| chr7 | 68301722 | 3 | 2 | chr7 | 68301720 | rs62460350 | C/T |
| chr7 | 70016932 | 4 | 1 | chr7 | 70016931 | rs4719020 | A/G |
| chr7 | 70152893 | 2 | 2 | chr7 | 70152891 | rs7783701 | C/T |
| chr7 | 70184436 | 8 | 2 | chr7 | 70184435 | rs1464856 | C/G |
| chr7 | 70656756 | 4 | 1 | chr7 | 70656754 | rs10242825 | A/C |
| chr7 | 70656756 | 4 | 1 | chr7 | 70656755 | rs7779947 | A/G |
| chr7 | 70686529 | 5 | 1 | chr7 | 70686527 | rs11975246 | C/T |
| chr7 | 70688418 | 2 | 4 | chr7 | 70688417 | rs10807732 | A/G |
| chr7 | 70796278 | 1 | 11 | chr7 | 70796276 | rs503001 | A/G |
| chr7 | 72756134 | 1 | 5 | chr7 | 72756131 | rs4363087 | C/T |
| chr7 | 72903113 | 1 | 3 | chr7 | 72903112 | rs4717111 | A/G |
| chr7 | 72913438 | 4 | 1 | chr7 | 72913436 | rs11770024 | C/T |
| chr7 | 73391187 | 10 | 1 | chr7 | 73391185 | rs539518 | A/G |
| chr7 | 75539664 | 2 | 2 | chr7 | 75539663 | rs1639617 | A/G |
| chr7 | 75971719 | 0 | 0 | chr7 | 75971718 | rs2260306 | C/G |
| chr7 | 75982497 | 9 | 3 | chr7 | 75982495 | rs1799126 | C/T |
| chr7 | 76794711 | 2 | 2 | chr7 | 76794709 | rs10271991 | C/T |
| chr7 | 78066738 | 2 | 3 | chr7 | 78066736 | rs319869 | C/T |
| chr7 | 78087231 | 1 | 7 | chr7 | 78087229 | rs38112 | C/T |
| chr7 | 79039704 | 3 | 1 | chr7 | 79039704 | rs2966584 | A/G |
| chr7 | 80502817 | 1 | 3 | chr7 | 80502815 | rs853077 | A/G |

| | | | | | | | | |
|---|---|---|---|---|---|---|---|---|
| chr7 | 81267526 | 6 | 8 | chr7 | 81267525 | rs6951168 | | G/T |
| chr7 | 81388293 | 3 | 3 | chr7 | 81388291 | rs1229477 | | C/T |
| chr7 | 81845236 | 2 | 4 | chr7 | 81845234 | rs1544464 | | C/T |
| chr7 | 81910893 | 2 | 2 | chr7 | 81910893 | rs6954596 | | A/G |
| chr7 | 83347995 | 3 | 3 | chr7 | 83347994 | rs11509918 | | G/T |
| chr7 | 86106844 | 1 | 8 | chr7 | 86106843 | rs274631 | | A/G |
| chr7 | 86193152 | 1 | 5 | chr7 | 86193150 | rs10952890 | | C/T |
| chr7 | 87928350 | 6 | 3 | chr7 | 87928348 | rs2213990 | | A/G |
| chr7 | 90160819 | 3 | 2 | chr7 | 90160816 | rs6465281 | | C/G |
| chr7 | 93172383 | 3 | 4 | chr7 | 93172381 | rs2078032 | | A/G |
| chr7 | 95708168 | 12 | 2 | chr7 | 95708167 | rs62472363 | | A/G |
| chr7 | 96912893 | 4 | 1 | chr7 | 96912891 | rs722065 | | A/G |
| chr7 | 96963991 | 4 | 1 | chr7 | 96963989 | rs4729364 | | C/T |
| chr7 | 97244201 | 2 | 6 | chr7 | 97244199 | rs6465617 | | C/T |
| chr7 | 97848213 | 4 | 1 | chr7 | 97848212 | rs3779194 | | C/T |
| chr7 | 98271403 | 3 | 1 | chr7 | 98271401 | rs10953275 | | C/T |
| chr7 | 98274542 | 5 | 1 | chr7 | 98274539 | rs6465725 | | C/T |
| chr7 | 99909968 | 2 | 3 | chr7 | 99909966 | rs7809801 | | C/T |
| chr7 | 100248593 | 7 | 2 | chr7 | 100248591 | rs61651680 | | C/T |
| chr7 | 100248593 | 7 | 2 | chr7 | 100248592 | rs314358 | | C/T |
| chr7 | 100398807 | 9 | 4 | chr7 | 100398806 | rs73168314 | | A/G |
| chr7 | 100399619 | 1 | 9 | chr7 | 100399617 | rs73168327 | | C/T |
| chr7 | 100518550 | 2 | 8 | chr7 | 100518548 | rs6948536 | | C/T |
| chr7 | 100604396 | 5 | 4 | chr7 | 100604395 | rs740107 | | C/T |
| chr7 | 100791291 | 2 | 6 | chr7 | 100791289 | rs7776842 | | C/T |
| chr7 | 100975573 | 8 | 5 | chr7 | 100975571 | rs9886175 | | C/T |
| chr7 | 100983687 | 5 | 9 | chr7 | 100983685 | rs13222730 | | C/T |
| chr7 | 101142933 | 5 | 2 | chr7 | 101142932 | rs2690893 | | C/T |
| chr7 | 101625822 | 3 | 1 | chr7 | 101625822 | rs6954742 | | C/G |
| chr7 | 101755951 | 3 | 5 | chr7 | 101755949 | rs2734624 | | C/T |
| chr7 | 101878741 | 7 | 12 | chr7 | 101878739 | rs4481505 | | C/T |
| chr7 | 104203881 | 1 | 4 | chr7 | 104203880 | rs13310494 | | A/G |

| | | | | | | | |
|---|---|---|---|---|---|---|---|
| chr7 | 104311703 | 6 | 1 | chr7 | 104311701 | rs17173 | A/G |
| chr7 | 105308446 | 3 | 2 | chr7 | 105308444 | rs212434 | C/T |
| chr7 | 107873800 | 5 | 1 | chr7 | 107873798 | rs17338462 | C/T |
| chr7 | 108015548 | 1 | 7 | chr7 | 108015547 | rs4730324 | A/G |
| chr7 | 108679664 | 1 | 6 | chr7 | 108679663 | rs766706 | C/T |
| chr7 | 108945008 | 2 | 5 | chr7 | 108945007 | rs6972727 | A/G |
| chr7 | 109034551 | 8 | 4 | chr7 | 109034550 | rs1721951 | A/G |
| chr7 | 109140144 | 1 | 3 | chr7 | 109140142 | rs73206879 | C/T |
| chr7 | 110054258 | 2 | 3 | chr7 | 110054256 | rs12533170 | C/T |
| chr7 | 110162066 | 1 | 6 | chr7 | 110162064 | rs34896727 | C/T |
| chr7 | 111598240 | 2 | 4 | chr7 | 111598239 | rs10487348 | A/G |
| chr7 | 111890291 | 4 | 5 | chr7 | 111890290 | rs2529588 | A/G |
| chr7 | 111916214 | 7 | 3 | chr7 | 111916213 | rs3095031 | A/G |
| chr7 | 112283351 | 2 | 2 | chr7 | 112283350 | rs649658 | A/G |
| chr7 | 113761985 | 2 | 6 | chr7 | 113761982 | rs2694935 | C/T |
| chr7 | 120939715 | 2 | 3 | chr7 | 120939714 | rs1406194 | A/C |
| chr7 | 124591701 | 5 | 1 | chr7 | 124591700 | rs58599819 | C/G |
| chr7 | 127583041 | 2 | 2 | chr7 | 127583039 | rs10447854 | C/T |
| chr7 | 128539160 | 3 | 3 | chr7 | 128539159 | rs4728153 | A/G |
| chr7 | 128554742 | 4 | 10 | chr7 | 128554741 | rs7809687 | G/T |
| chr7 | 128566907 | 3 | 2 | chr7 | 128566904 | rs4728155 | C/T |
| chr7 | 128576824 | 0 | 0 | chr7 | 128576823 | rs10215953 | A/G |
| chr7 | 129141890 | 2 | 5 | chr7 | 129141888 | rs3800599 | A/C |
| chr7 | 130909668 | 1 | 5 | chr7 | 130909668 | rs1643278 | A/G |
| chr7 | 131502051 | 1 | 5 | chr7 | 131502050 | rs4731852 | A/G |
| chr7 | 131867081 | 7 | 5 | chr7 | 131867080 | rs6467439 | G/T |
| chr7 | 132814190 | 3 | 7 | chr7 | 132814188 | rs1424584 | C/T |
| chr7 | 133116969 | 2 | 2 | chr7 | 133116968 | rs1833331 | A/G |
| chr7 | 133676357 | 1 | 6 | chr7 | 133676356 | rs1643053 | A/G |
| chr7 | 133743514 | 2 | 3 | chr7 | 133743513 | rs6975650 | A/G |
| chr7 | 133757639 | 4 | 3 | chr7 | 133757638 | rs706154 | C/T |
| chr7 | 134059477 | 1 | 5 | chr7 | 134059476 | rs10243536 | A/G |

| | | | | | | | |
|---|---|---|---|---|---|---|---|
| chr7 | 134148837 | 3 | 2 | chr7 | 134148836 | rs1563046 | C/T |
| chr7 | 134546924 | 5 | 2 | chr7 | 134546922 | rs292602 | C/T |
| chr7 | 134599145 | 4 | 2 | chr7 | 134599144 | rs292646 | A/G |
| chr7 | 136021352 | 3 | 2 | chr7 | 136021351 | rs10257276 | A/G |
| chr7 | 136146323 | 4 | 4 | chr7 | 136146321 | rs6961476 | C/T |
| chr7 | 136743323 | 4 | 2 | chr7 | 136743320 | rs7800454 | C/T |
| chr7 | 138253064 | 3 | 5 | chr7 | 138253062 | rs2774962 | C/T |
| chr7 | 138257290 | 9 | 5 | chr7 | 138257289 | rs10279875 | A/G |
| chr7 | 138676691 | 2 | 5 | chr7 | 138676691 | rs10265 | G/T |
| chr7 | 140426210 | 6 | 2 | chr7 | 140426208 | rs557962 | C/T |
| chr7 | 140426210 | 6 | 2 | chr7 | 140426209 | rs73738933 | A/G |
| chr7 | 140552744 | 1 | 10 | chr7 | 140552742 | rs6464420 | C/T |
| chr7 | 140564025 | 1 | 4 | chr7 | 140564024 | rs6959814 | G/T |
| chr7 | 141659295 | 1 | 4 | chr7 | 141659294 | rs2008057 | A/G |
| chr7 | 142616127 | 4 | 3 | chr7 | 142616126 | rs562509 | A/G |
| chr7 | 142768187 | 6 | 5 | chr7 | 142768184 | rs6967727 | C/G |
| chr7 | 146023001 | 1 | 5 | chr7 | 146022999 | rs2109280 | A/G |
| chr7 | 146405830 | 1 | 5 | chr7 | 146405828 | rs1548743 | A/C |
| chr7 | 147163920 | 3 | 1 | chr7 | 147163919 | rs12533240 | A/G |
| chr7 | 147944295 | 2 | 2 | chr7 | 147944292 | rs6942631 | C/G |
| chr7 | 148622645 | 2 | 3 | chr7 | 148622644 | rs13236978 | A/G |
| chr7 | 148788931 | 4 | 2 | chr7 | 148788929 | rs2293345 | A/G |
| chr7 | 149196423 | 1 | 14 | chr7 | 149196421 | rs3735334 | C/T |
| chr7 | 149201627 | 10 | 3 | chr7 | 149201625 | rs4401760 | A/C |
| chr7 | 150195009 | 2 | 9 | chr7 | 150195007 | rs10236489 | C/T |
| chr7 | 150299448 | 3 | 2 | chr7 | 150299446 | rs4725984 | C/T |
| chr7 | 150329812 | 11 | 2 | chr7 | 150329811 | rs1800780 | A/G |
| chr7 | 150386105 | 4 | 3 | chr7 | 150386105 | rs2069443 | A/C |
| chr7 | 151040414 | 4 | 1 | chr7 | 151040412 | rs4725424 | C/T |
| chr7 | 151739355 | 2 | 6 | chr7 | 151739353 | rs2982764 | C/G |
| chr7 | 151792909 | 3 | 1 | chr7 | 151792907 | rs10254368 | C/T |
| chr7 | 151943397 | 6 | 2 | chr7 | 151943395 | rs7797722 | C/T |

| | | | | | | | |
|---|---|---|---|---|---|---|---|
| chr7 | 152188237 | 4 | 5 | chr7 | 152188235 | rs11768555 | C/T |
| chr7 | 152547632 | 3 | 2 | chr7 | 152547630 | rs1124152 | C/T |
| chr7 | 152547632 | 3 | 2 | chr7 | 152547631 | rs4266561 | A/G |
| chr7 | 152976007 | 2 | 4 | chr7 | 152976006 | rs58711591 | G/T |
| chr7 | 153250881 | 2 | 2 | chr7 | 153250880 | rs11974352 | A/G |
| chr7 | 153851754 | 2 | 3 | chr7 | 153851754 | rs10264387 | C/G |
| chr7 | 153877262 | 2 | 3 | chr7 | 153877261 | rs28479560 | A/G |
| chr7 | 154042842 | 3 | 2 | chr7 | 154042840 | rs4960608 | C/T |
| chr7 | 154493526 | 3 | 2 | chr7 | 154493523 | rs1800883 | C/G |
| chr7 | 154493526 | 3 | 2 | chr7 | 154493524 | rs55967309 | C/T |
| chr7 | 154522411 | 5 | 5 | chr7 | 154522410 | rs2698492 | A/G |
| chr7 | 154633529 | 5 | 2 | chr7 | 154633528 | rs11765475 | A/G |
| chr7 | 154643215 | 2 | 2 | chr7 | 154643214 | rs10265531 | A/G |
| chr7 | 154713429 | 6 | 4 | chr7 | 154713428 | rs9691746 | A/G |
| chr7 | 154844197 | 0 | 0 | chr7 | 154844197 | rs10233765 | C/T |
| chr7 | 154895951 | 8 | 2 | chr7 | 154895949 | rs13237337 | C/T |
| chr7 | 154910644 | 1 | 11 | chr7 | 154910641 | rs1861970 | C/G |
| chr7 | 154923882 | 1 | 13 | chr7 | 154923881 | rs732399 | A/G |
| chr7 | 154970660 | 3 | 3 | chr7 | 154970659 | rs11973080 | A/G |
| chr7 | 155026593 | 1 | 6 | chr7 | 155026592 | rs4716685 | A/G |
| chr7 | 155295920 | 2 | 5 | chr7 | 155295917 | rs1233561 | A/C |
| chr7 | 155304828 | 7 | 5 | chr7 | 155304827 | rs9654733 | A/G |
| chr7 | 155313049 | 1 | 4 | chr7 | 155313049 | rs12113158 | G/T |
| chr7 | 155316986 | 2 | 4 | chr7 | 155316986 | rs288756 | A/G |
| chr7 | 155349364 | 1 | 3 | chr7 | 155349363 | rs10261156 | A/G |
| chr7 | 155852039 | 2 | 4 | chr7 | 155852038 | rs10266659 | A/G |
| chr7 | 155913186 | 2 | 9 | chr7 | 155913185 | rs1404219 | C/T |
| chr7 | 155964344 | 1 | 7 | chr7 | 155964342 | rs2037623 | C/T |
| chr7 | 155991460 | 3 | 2 | chr7 | 155991458 | rs73506555 | A/C |
| chr7 | 156574276 | 2 | 2 | chr7 | 156574275 | rs10249680 | G/T |
| chr7 | 156675132 | 2 | 2 | chr7 | 156675129 | rs4716693 | C/G |
| chr7 | 156768051 | 3 | 8 | chr7 | 156768049 | rs1182429 | A/G |

| | | | | | | | |
|---|---|---|---|---|---|---|---|
| chr7 | 156778253 | 4 | 2 | chr7 | 156778252 | rs2527872 | C/T |
| chr7 | 156928843 | 0 | 0 | chr7 | 156928841 | rs4716479 | A/C |
| chr7 | 156973018 | 6 | 9 | chr7 | 156973016 | rs221215 | C/T |
| chr7 | 157281988 | 4 | 1 | chr7 | 157281986 | rs4716498 | C/T |
| chr7 | 157387634 | 1 | 3 | chr7 | 157387632 | rs10280879 | C/T |
| chr7 | 157403944 | 7 | 6 | chr7 | 157403942 | rs10263646 | A/C |
| chr7 | 157403944 | 7 | 6 | chr7 | 157403943 | rs35722158 | -/G |
| chr7 | 157430619 | 2 | 5 | chr7 | 157430618 | rs6977123 | A/G |
| chr7 | 157483790 | 11 | 4 | chr7 | 157483789 | rs11984274 | A/G |
| chr7 | 157488311 | 4 | 2 | chr7 | 157488309 | rs10280324 | C/T |
| chr7 | 157489438 | 3 | 6 | chr7 | 157489436 | rs6459827 | C/T |
| chr7 | 157513951 | 12 | 7 | chr7 | 157513950 | rs892732 | C/T |
| chr7 | 157652269 | 4 | 1 | chr7 | 157652266 | rs10236479 | C/T |
| chr7 | 157660700 | 1 | 5 | chr7 | 157660699 | rs6947028 | A/G |
| chr7 | 157672973 | 3 | 6 | chr7 | 157672971 | rs4909127 | C/T |
| chr7 | 157763025 | 4 | 4 | chr7 | 157763025 | rs2335478 | C/T |
| chr7 | 157810008 | 2 | 10 | chr7 | 157810006 | rs10949715 | A/C |
| chr7 | 158108765 | 1 | 4 | chr7 | 158108763 | rs11760681 | C/T |
| chr7 | 158108765 | 1 | 4 | chr7 | 158108763 | rs56428147 | C/T |
| chr7 | 158781419 | 3 | 1 | chr7 | 158781417 | rs3976714 | A/G |
| chr8 | 281630 | 5 | 3 | chr8 | 281628 | rs10104540 | C/T |
| chr8 | 536487 | 3 | 1 | chr8 | 536486 | rs35639704 | A/G |
| chr8 | 607718 | 1 | 3 | chr8 | 607717 | rs896521 | C/T |
| chr8 | 667915 | 4 | 2 | chr8 | 667914 | rs73670602 | A/G |
| chr8 | 1129457 | 5 | 3 | chr8 | 1129456 | rs6558420 | A/G |
| chr8 | 1183260 | 3 | 4 | chr8 | 1183259 | rs4976879 | C/G |
| chr8 | 1262237 | 3 | 2 | chr8 | 1262235 | rs11136382 | C/T |
| chr8 | 1270252 | 5 | 1 | chr8 | 1270249 | rs11778654 | C/T |
| chr8 | 1630694 | 3 | 2 | chr8 | 1630693 | rs7835892 | A/G |
| chr8 | 1736348 | 4 | 1 | chr8 | 1736347 | rs6558541 | A/G |
| chr8 | 1809829 | 1 | 5 | chr8 | 1809829 | rs4875947 | A/C |
| chr8 | 1861126 | 2 | 5 | chr8 | 1861124 | rs55742374 | C/T |

| | | | | | | | |
|---|---|---|---|---|---|---|---|
| chr8 | 1898481 | 1 | 5 | chr8 | 1898478 | rs13268460 | A/C |
| chr8 | 1971137 | 1 | 4 | chr8 | 1971136 | rs6558586 | A/G |
| chr8 | 2082386 | 1 | 9 | chr8 | 2082385 | rs11774909 | A/G |
| chr8 | 2190869 | 1 | 5 | chr8 | 2190867 | rs12155785 | C/T |
| chr8 | 3177303 | 1 | 15 | chr8 | 3177302 | rs2551043 | C/T |
| chr8 | 4016923 | 3 | 3 | chr8 | 4016920 | rs1714757 | G/T |
| chr8 | 5339167 | 2 | 2 | chr8 | 5339166 | rs974997 | A/G |
| chr8 | 6458964 | 3 | 4 | chr8 | 6458962 | rs2980663 | A/G |
| chr8 | 6634555 | 3 | 2 | chr8 | 6634554 | rs13275062 | C/G |
| chr8 | 6680073 | 1 | 4 | chr8 | 6680071 | rs2978897 | C/G |
| chr8 | 6822923 | 2 | 2 | chr8 | 6822923 | rs4840655 | G/T |
| chr8 | 6842040 | 2 | 2 | chr8 | 6842040 | rs4840655 | G/T |
| chr8 | 8890268 | 3 | 2 | chr8 | 8890265 | rs6987569 | C/T |
| chr8 | 8906609 | 3 | 3 | chr8 | 8906608 | rs9650616 | G/T |
| chr8 | 9027986 | 3 | 2 | chr8 | 9027986 | rs189798 | C/T |
| chr8 | 9288832 | 6 | 1 | chr8 | 9288830 | rs10106249 | C/G |
| chr8 | 9434224 | 1 | 11 | chr8 | 9434223 | rs7825581 | A/G |
| chr8 | 9777700 | 19 | 2 | chr8 | 9777698 | rs10094513 | C/T |
| chr8 | 9833600 | 5 | 6 | chr8 | 9833598 | rs615171 | C/T |
| chr8 | 9850728 | 4 | 2 | chr8 | 9850726 | rs671370 | A/G |
| chr8 | 10111071 | 2 | 5 | chr8 | 10111070 | rs12234935 | A/G |
| chr8 | 10181720 | 5 | 1 | chr8 | 10181719 | rs10903322 | A/G |
| chr8 | 10391100 | 8 | 2 | chr8 | 10391097 | rs7357476 | A/C |
| chr8 | 10443570 | 4 | 5 | chr8 | 10443567 | rs7846306 | C/G |
| chr8 | 10497248 | 5 | 1 | chr8 | 10497245 | rs11250047 | C/T |
| chr8 | 10506643 | 2 | 4 | chr8 | 10506642 | rs35602868 | A/G |
| chr8 | 10520149 | 5 | 1 | chr8 | 10520149 | rs4292650 | C/G |
| chr8 | 10524633 | 4 | 2 | chr8 | 10524631 | rs4289770 | C/T |
| chr8 | 10821180 | 5 | 5 | chr8 | 10821178 | rs10086521 | C/T |
| chr8 | 10862153 | 1 | 5 | chr8 | 10862150 | rs9792334 | C/G |
| chr8 | 10862153 | 1 | 5 | chr8 | 10862151 | rs10107105 | A/C |
| chr8 | 11352402 | 0 | 0 | chr8 | 11352400 | rs13253092 | C/T |

| | | | | | | | |
|---|---|---|---|---|---|---|---|
| chr8 | 11413676 | 1 | 4 | chr8 | 11413674 | rs2618451 | A/G |
| chr8 | 11457513 | 1 | 5 | chr8 | 11457512 | rs35401006 | C/G |
| chr8 | 11515765 | 3 | 6 | chr8 | 11515764 | rs1017803 | C/T |
| chr8 | 11523760 | 12 | 4 | chr8 | 11523759 | rs34457264 | A/G |
| chr8 | 11532066 | 4 | 2 | chr8 | 11532065 | rs2256241 | C/T |
| chr8 | 11617363 | 3 | 1 | chr8 | 11617362 | rs12550668 | A/G |
| chr8 | 11658179 | 2 | 5 | chr8 | 11658177 | rs17153782 | C/T |
| chr8 | 11725558 | 1 | 4 | chr8 | 11725556 | rs4841600 | C/T |
| chr8 | 12895576 | 5 | 11 | chr8 | 12895575 | rs11204005 | A/G |
| chr8 | 13034793 | 1 | 3 | chr8 | 13034792 | rs10107321 | A/G |
| chr8 | 14121186 | 1 | 3 | chr8 | 14121185 | rs34207874 | -/C |
| chr8 | 14121186 | 1 | 3 | chr8 | 14121186 | rs4831555 | C/G |
| chr8 | 14721184 | 4 | 2 | chr8 | 14721182 | rs4831651 | C/T |
| chr8 | 15408837 | 3 | 5 | chr8 | 15408835 | rs7838229 | C/T |
| chr8 | 15793101 | 2 | 2 | chr8 | 15793099 | rs2604333 | A/G |
| chr8 | 16041168 | 8 | 1 | chr8 | 16041167 | rs12718375 | A/G |
| chr8 | 17820370 | 1 | 12 | chr8 | 17820369 | rs208751 | A/G |
| chr8 | 17926318 | 7 | 11 | chr8 | 17926316 | rs527173 | A/G |
| chr8 | 18283660 | 4 | 1 | chr8 | 18283658 | rs6995693 | C/T |
| chr8 | 18848992 | 4 | 2 | chr8 | 18848991 | rs6982330 | A/G |
| chr8 | 18978624 | 5 | 7 | chr8 | 18978622 | rs2063079 | C/T |
| chr8 | 18982882 | 3 | 2 | chr8 | 18982880 | rs10101681 | C/T |
| chr8 | 19315313 | 9 | 2 | chr8 | 19315311 | rs12679712 | C/G |
| chr8 | 19445754 | 1 | 5 | chr8 | 19445753 | rs10282948 | G/T |
| chr8 | 20206390 | 5 | 6 | chr8 | 20206388 | rs28695244 | A/C |
| chr8 | 20285470 | 1 | 4 | chr8 | 20285469 | rs10788701 | A/G |
| chr8 | 20531909 | 2 | 2 | chr8 | 20531908 | rs17492771 | A/G |
| chr8 | 21609313 | 1 | 3 | chr8 | 21609313 | rs10088934 | C/G |
| chr8 | 21658844 | 6 | 1 | chr8 | 21658843 | rs6587005 | A/G |
| chr8 | 21784834 | 6 | 2 | chr8 | 21784833 | rs755423 | C/G |
| chr8 | 22318267 | 4 | 2 | chr8 | 22318265 | rs896378 | C/T |
| chr8 | 22886424 | 1 | 3 | chr8 | 22886423 | rs7009934 | A/G |

| | | | | | | | |
|---|---|---|---|---|---|---|---|
| chr8 | 23201793 | 3 | 1 | chr8 | 23201792 | rs13278751 | A/G |
| chr8 | 25246637 | 6 | 1 | chr8 | 25246636 | rs2468895 | A/G |
| chr8 | 25956508 | 3 | 5 | chr8 | 25956506 | rs6557875 | C/T |
| chr8 | 26123050 | 1 | 8 | chr8 | 26123049 | rs7828019 | A/G |
| chr8 | 26951350 | 5 | 1 | chr8 | 26951348 | rs7834392 | C/T |
| chr8 | 27048921 | 4 | 2 | chr8 | 27048920 | rs6557985 | A/G |
| chr8 | 27374461 | 2 | 4 | chr8 | 27374460 | rs2292976 | A/G |
| chr8 | 27616501 | 2 | 3 | chr8 | 27616499 | rs482530 | C/T |
| chr8 | 27616549 | 2 | 3 | chr8 | 27616548 | rs13253543 | A/G |
| chr8 | 27675467 | 2 | 2 | chr8 | 27675466 | rs4236674 | A/G |
| chr8 | 27820547 | 2 | 3 | chr8 | 27820545 | rs2726987 | C/T |
| chr8 | 27948736 | 1 | 4 | chr8 | 27948733 | rs6987753 | C/G |
| chr8 | 28226449 | 5 | 1 | chr8 | 28226448 | rs2614082 | A/G |
| chr8 | 28242394 | 3 | 1 | chr8 | 28242392 | rs2645722 | G/T |
| chr8 | 29130322 | 1 | 3 | chr8 | 29130321 | rs62502853 | C/G |
| chr8 | 29192779 | 3 | 2 | chr8 | 29192778 | rs694464 | A/G |
| chr8 | 29510417 | 4 | 1 | chr8 | 29510415 | rs7829283 | A/C |
| chr8 | 30004409 | 2 | 2 | chr8 | 30004407 | rs13259846 | C/T |
| chr8 | 30606887 | 7 | 2 | chr8 | 30606885 | rs6991816 | C/T |
| chr8 | 31453459 | 4 | 3 | chr8 | 31453457 | rs17658614 | C/T |
| chr8 | 31564950 | 2 | 5 | chr8 | 31564948 | rs763552 | A/G |
| chr8 | 31701039 | 3 | 10 | chr8 | 31701037 | rs4469409 | C/T |
| chr8 | 32045409 | 1 | 3 | chr8 | 32045408 | rs1564126 | G/T |
| chr8 | 32340531 | 1 | 4 | chr8 | 32340529 | rs55986591 | C/G |
| chr8 | 36908749 | 3 | 4 | chr8 | 36908748 | rs7386222 | A/G |
| chr8 | 37611017 | 2 | 2 | chr8 | 37611016 | rs7830397 | G/T |
| chr8 | 39062449 | 2 | 7 | chr8 | 39062447 | rs10958533 | C/T |
| chr8 | 39306414 | 3 | 1 | chr8 | 39306412 | rs7009846 | C/T |
| chr8 | 40342404 | 3 | 1 | chr8 | 40342403 | rs369891 | A/G |
| chr8 | 40355818 | 11 | 4 | chr8 | 40355816 | rs12680256 | C/T |
| chr8 | 40566100 | 4 | 2 | chr8 | 40566098 | rs10504029 | C/T |
| chr8 | 40759489 | 2 | 3 | chr8 | 40759487 | rs10216610 | C/T |

| | | | | | | | |
|---|---|---|---|---|---|---|---|
| chr8 | 40994660 | 2 | 2 | chr8 | 40994659 | rs11990579 | A/G |
| chr8 | 42634957 | 3 | 2 | chr8 | 42634957 | rs1868859 | C/T |
| chr8 | 43543567 | 1 | 9 | chr8 | 43543566 | rs6987552 | A/G |
| chr8 | 47414395 | 6 | 9 | chr8 | 47414394 | rs10435660 | A/G |
| chr8 | 49220524 | 1 | 15 | chr8 | 49220522 | rs13263949 | C/T |
| chr8 | 49222451 | 3 | 3 | chr8 | 49222450 | rs35040443 | A/G |
| chr8 | 50671657 | 1 | 3 | chr8 | 50671655 | rs7017960 | C/T |
| chr8 | 51541879 | 6 | 3 | chr8 | 51541877 | rs1904997 | G/T |
| chr8 | 53542653 | 1 | 9 | chr8 | 53542651 | rs2613273 | A/G |
| chr8 | 54635627 | 4 | 2 | chr8 | 54635626 | rs1600078 | A/G |
| chr8 | 55251063 | 7 | 9 | chr8 | 55251062 | rs413974 | A/G |
| chr8 | 55277307 | 6 | 3 | chr8 | 55277306 | rs311407 | C/T |
| chr8 | 56954714 | 1 | 3 | chr8 | 56954712 | rs1450741 | C/T |
| chr8 | 58823124 | 2 | 2 | chr8 | 58823122 | rs4738619 | C/T |
| chr8 | 59407311 | 5 | 7 | chr8 | 59407309 | rs2925660 | C/T |
| chr8 | 59589434 | 6 | 1 | chr8 | 59589432 | rs2326078 | C/T |
| chr8 | 60004357 | 1 | 10 | chr8 | 60004355 | rs10094258 | C/T |
| chr8 | 61466175 | 2 | 2 | chr8 | 61466175 | rs17814894 | A/G |
| chr8 | 61651379 | 1 | 3 | chr8 | 61651377 | rs2981277 | A/G |
| chr8 | 62011640 | 9 | 2 | chr8 | 62011639 | rs10087102 | A/G |
| chr8 | 64068122 | 1 | 5 | chr8 | 64068120 | rs10957262 | C/T |
| chr8 | 64116051 | 1 | 3 | chr8 | 64116049 | rs12547126 | C/T |
| chr8 | 64373776 | 2 | 3 | chr8 | 64373775 | rs7459882 | A/G |
| chr8 | 64441191 | 4 | 3 | chr8 | 64441190 | rs6472092 | A/G |
| chr8 | 66315057 | 6 | 1 | chr8 | 66315056 | rs4448317 | A/G |
| chr8 | 66620301 | 2 | 4 | chr8 | 66620299 | rs10089790 | C/T |
| chr8 | 68511040 | 3 | 2 | chr8 | 68511040 | rs10957386 | A/G |
| chr8 | 68597481 | 2 | 2 | chr8 | 68597479 | rs7821969 | C/T |
| chr8 | 68800981 | 3 | 2 | chr8 | 68800979 | rs2911954 | C/T |
| chr8 | 68943166 | 4 | 3 | chr8 | 68943165 | rs4737862 | A/G |
| chr8 | 70216771 | 2 | 3 | chr8 | 70216769 | rs2472141 | C/T |
| chr8 | 70535527 | 7 | 19 | chr8 | 70535526 | rs7829823 | A/G |

| | | | | | | | |
|---|---|---|---|---|---|---|---|
| chr8 | 70586728 | 9 | 5 | chr8 | 70586727 | rs7830174 | G/T |
| chr8 | 70786149 | 2 | 6 | chr8 | 70786149 | rs60255097 | C/G |
| chr8 | 71133363 | 1 | 5 | chr8 | 71133362 | rs7016464 | C/G |
| chr8 | 71682578 | 2 | 2 | chr8 | 71682577 | rs268602 | G/T |
| chr8 | 72353784 | 3 | 3 | chr8 | 72353784 | rs13254654 | A/G |
| chr8 | 73470730 | 11 | 1 | chr8 | 73470729 | rs830495 | C/T |
| chr8 | 73797427 | 2 | 5 | chr8 | 73797425 | rs2247329 | A/G |
| chr8 | 74109264 | 4 | 7 | chr8 | 74109263 | rs1905078 | A/C |
| chr8 | 74556130 | 9 | 4 | chr8 | 74556128 | rs6472780 | C/T |
| chr8 | 74561971 | 7 | 7 | chr8 | 74561970 | rs2383915 | A/G |
| chr8 | 74862632 | 7 | 2 | chr8 | 74862630 | rs1564530 | A/G |
| chr8 | 77479165 | 2 | 3 | chr8 | 77479164 | rs3812486 | C/T |
| chr8 | 78938576 | 2 | 3 | chr8 | 78938575 | rs2129465 | C/T |
| chr8 | 79609970 | 12 | 1 | chr8 | 79609968 | rs7837755 | C/T |
| chr8 | 80359806 | 3 | 1 | chr8 | 80359804 | rs1870575 | A/G |
| chr8 | 81421660 | 1 | 3 | chr8 | 81421659 | rs12542061 | -/A/G |
| chr8 | 84092830 | 3 | 1 | chr8 | 84092829 | rs7825303 | A/G |
| chr8 | 84180149 | 2 | 4 | chr8 | 84180147 | rs1449803 | A/G |
| chr8 | 84712810 | 10 | 2 | chr8 | 84712809 | rs1387241 | C/T |
| chr8 | 87360715 | 7 | 1 | chr8 | 87360714 | rs13266677 | A/G |
| chr8 | 88328327 | 6 | 6 | chr8 | 88328326 | rs13264977 | C/G |
| chr8 | 89056685 | 1 | 3 | chr8 | 89056684 | rs34030179 | A/G |
| chr8 | 92120425 | 2 | 2 | chr8 | 92120423 | rs6983337 | C/T |
| chr8 | 92120425 | 2 | 2 | chr8 | 92120423 | rs71510468 | CGG/TGA |
| chr8 | 92120425 | 2 | 2 | chr8 | 92120425 | rs7017271 | A/G |
| chr8 | 92176246 | 2 | 2 | chr8 | 92176245 | rs12542178 | A/G |
| chr8 | 93330147 | 9 | 2 | chr8 | 93330145 | rs1444507 | C/G |
| chr8 | 94253316 | 3 | 6 | chr8 | 94253314 | rs278606 | G/T |
| chr8 | 95064921 | 6 | 1 | chr8 | 95064920 | rs4391396 | A/G |
| chr8 | 97095555 | 3 | 2 | chr8 | 97095553 | rs261579 | C/T |
| chr8 | 97448456 | 3 | 3 | chr8 | 97448455 | rs938831 | A/G |
| chr8 | 99012623 | 2 | 2 | chr8 | 99012621 | rs2290470 | C/T |

| | | | | | | | |
|---|---|---|---|---|---|---|---|
| chr8 | 99024477 | 2 | 3 | chr8 | 99024476 | rs12547144 | A/G |
| chr8 | 99484078 | 3 | 7 | chr8 | 99484077 | rs6997978 | G/T |
| chr8 | 101541545 | 6 | 1 | chr8 | 101541545 | rs2022920 | C/G |
| chr8 | 101730779 | 2 | 2 | chr8 | 101730778 | rs7016136 | G/T |
| chr8 | 102807844 | 1 | 3 | chr8 | 102807843 | rs2211919 | C/T |
| chr8 | 102836180 | 3 | 3 | chr8 | 102836179 | rs9693504 | A/G |
| chr8 | 103264809 | 2 | 2 | chr8 | 103264808 | rs1265128 | A/G |
| chr8 | 103872190 | 4 | 4 | chr8 | 103872189 | rs2436871 | C/T |
| chr8 | 104582572 | 1 | 5 | chr8 | 104582570 | rs36056848 | C/T |
| chr8 | 104879269 | 5 | 4 | chr8 | 104879268 | rs3104250 | A/G |
| chr8 | 105769760 | 1 | 4 | chr8 | 105769758 | rs4734805 | C/T |
| chr8 | 107023593 | 3 | 4 | chr8 | 107023591 | rs7818235 | C/T |
| chr8 | 107059451 | 3 | 1 | chr8 | 107059450 | rs4734893 | G/T |
| chr8 | 107493832 | 11 | 4 | chr8 | 107493830 | rs7017001 | C/T |
| chr8 | 112750568 | 5 | 3 | chr8 | 112750566 | rs13259383 | C/T |
| chr8 | 112772268 | 3 | 4 | chr8 | 112772267 | rs13267773 | A/G |
| chr8 | 112874501 | 9 | 1 | chr8 | 112874499 | rs16882595 | C/T |
| chr8 | 114643302 | 11 | 1 | chr8 | 114643301 | rs4242555 | G/T |
| chr8 | 118046783 | 4 | 4 | chr8 | 118046782 | rs13269147 | A/G |
| chr8 | 119479103 | 3 | 1 | chr8 | 119479101 | rs6997433 | C/T |
| chr8 | 119544808 | 6 | 7 | chr8 | 119544807 | rs4075614 | C/T |
| chr8 | 120617858 | 2 | 4 | chr8 | 120617857 | rs11782176 | A/G |
| chr8 | 120776781 | 2 | 2 | chr8 | 120776780 | rs9297602 | A/G |
| chr8 | 120848971 | 4 | 1 | chr8 | 120848971 | rs4871577 | A/G |
| chr8 | 121289002 | 5 | 1 | chr8 | 121289000 | rs957694 | C/T |
| chr8 | 121364222 | 9 | 1 | chr8 | 121364220 | rs7817682 | C/T |
| chr8 | 122348024 | 7 | 1 | chr8 | 122348023 | rs12541012 | G/T |
| chr8 | 123642796 | 5 | 13 | chr8 | 123642793 | rs9656809 | C/G |
| chr8 | 124013581 | 8 | 1 | chr8 | 124013580 | rs7001245 | A/G |
| chr8 | 124241449 | 2 | 2 | chr8 | 124241447 | rs1670188 | A/G |
| chr8 | 124537652 | 1 | 3 | chr8 | 124537651 | rs10216826 | A/G |
| chr8 | 126350246 | 3 | 1 | chr8 | 126350245 | rs6995986 | A/G |

| | | | | | | | |
|---|---|---|---|---|---|---|---|
| chr8 | 126758872 | 6 | 1 | chr8 | 126758871 | rs34583020 | A/G |
| chr8 | 128404429 | 3 | 4 | chr8 | 128404427 | rs437980 | G/T |
| chr8 | 128418909 | 2 | 4 | chr8 | 128418907 | rs73705800 | C/T |
| chr8 | 128418909 | 2 | 4 | chr8 | 128418908 | rs13275275 | A/G |
| chr8 | 128498844 | 12 | 3 | chr8 | 128498841 | rs6470510 | C/T |
| chr8 | 128642480 | 8 | 2 | chr8 | 128642479 | rs12543106 | A/G |
| chr8 | 129112550 | 1 | 6 | chr8 | 129112549 | rs2720687 | C/T |
| chr8 | 129222415 | 1 | 4 | chr8 | 129222413 | rs2608038 | A/G |
| chr8 | 130385235 | 8 | 4 | chr8 | 130385233 | rs6470720 | C/T |
| chr8 | 132584575 | 5 | 1 | chr8 | 132584573 | rs17619279 | C/T |
| chr8 | 132971386 | 3 | 1 | chr8 | 132971384 | rs10956618 | C/T |
| chr8 | 134119860 | 4 | 2 | chr8 | 134119859 | rs3739266 | C/T |
| chr8 | 134355376 | 2 | 14 | chr8 | 134355374 | rs2977505 | C/T |
| chr8 | 134456538 | 3 | 1 | chr8 | 134456536 | rs6980814 | A/C |
| chr8 | 134498672 | 16 | 2 | chr8 | 134498670 | rs2736868 | C/T |
| chr8 | 134620815 | 3 | 1 | chr8 | 134620812 | rs62520289 | C/G |
| chr8 | 134746072 | 1 | 4 | chr8 | 134746070 | rs2922500 | C/T |
| chr8 | 135387646 | 2 | 4 | chr8 | 135387644 | rs884515 | C/T |
| chr8 | 135393358 | 8 | 2 | chr8 | 135393357 | rs4243512 | A/G |
| chr8 | 135745614 | 1 | 3 | chr8 | 135745613 | rs7843350 | A/G |
| chr8 | 136108805 | 1 | 3 | chr8 | 136108804 | rs6578070 | A/G |
| chr8 | 136125230 | 4 | 4 | chr8 | 136125229 | rs1480810 | G/T |
| chr8 | 136247132 | 3 | 3 | chr8 | 136247130 | rs10101981 | A/C |
| chr8 | 137198264 | 2 | 5 | chr8 | 137198262 | rs11166633 | C/T |
| chr8 | 137310803 | 2 | 3 | chr8 | 137310802 | rs12679776 | A/G |
| chr8 | 137727659 | 1 | 3 | chr8 | 137727657 | rs305358 | C/T |
| chr8 | 138842368 | 3 | 1 | chr8 | 138842367 | rs4517159 | A/G |
| chr8 | 139231335 | 2 | 2 | chr8 | 139231334 | rs16908376 | A/G |
| chr8 | 139426621 | 2 | 6 | chr8 | 139426620 | rs11166814 | A/G |
| chr8 | 139716444 | 4 | 1 | chr8 | 139716443 | rs10091563 | A/G |
| chr8 | 139894575 | 4 | 1 | chr8 | 139894574 | rs6577951 | A/G |
| chr8 | 140758657 | 4 | 11 | chr8 | 140758655 | rs7825701 | C/T |

| | | | | | | | |
|---|---|---|---|---|---|---|---|
| chr8 | 140871927 | 5 | 1 | chr8 | 140871925 | rs4736095 | C/T |
| chr8 | 140874320 | 9 | 5 | chr8 | 140874318 | rs13260415 | A/C |
| chr8 | 140956823 | 1 | 4 | chr8 | 140956821 | rs1870810 | C/T |
| chr8 | 141343611 | 5 | 8 | chr8 | 141343610 | rs4074946 | A/G |
| chr8 | 141365567 | 0 | 0 | chr8 | 141365566 | rs12542930 | A/G |
| chr8 | 141455494 | 7 | 3 | chr8 | 141455492 | rs7834518 | C/T |
| chr8 | 141476025 | 2 | 5 | chr8 | 141476024 | rs4577934 | A/G |
| chr8 | 142168159 | 3 | 4 | chr8 | 142168158 | rs6990155 | A/G |
| chr8 | 142175301 | 2 | 3 | chr8 | 142175299 | rs11167022 | A/C |
| chr8 | 142359927 | 6 | 1 | chr8 | 142359925 | rs1019961 | C/T |
| chr8 | 142394644 | 3 | 6 | chr8 | 142394642 | rs2369616 | C/T |
| chr8 | 142405712 | 7 | 1 | chr8 | 142405710 | rs2060356 | C/T |
| chr8 | 142640225 | 4 | 3 | chr8 | 142640223 | rs13267815 | C/T |
| chr8 | 142831658 | 3 | 1 | chr8 | 142831656 | rs11775168 | C/T |
| chr8 | 142876022 | 4 | 2 | chr8 | 142876020 | rs58874513 | C/T |
| chr8 | 142880128 | 2 | 4 | chr8 | 142880126 | rs6583642 | C/T |
| chr8 | 143069976 | 2 | 6 | chr8 | 143069974 | rs7004581 | A/C |
| chr8 | 143109202 | 3 | 2 | chr8 | 143109200 | rs2029620 | A/G |
| chr8 | 143155845 | 3 | 1 | chr8 | 143155844 | rs4917235 | G/T |
| chr8 | 143192316 | 1 | 10 | chr8 | 143192314 | rs1109507 | C/T |
| chr8 | 143215110 | 0 | 0 | chr8 | 143215109 | rs35064710 | -/T |
| chr8 | 143215110 | 0 | 0 | chr8 | 143215110 | rs67859635 | -/T |
| chr8 | 143244462 | 2 | 5 | chr8 | 143244461 | rs7462473 | A/G |
| chr8 | 143250850 | 2 | 6 | chr8 | 143250849 | rs57070783 | A/G |
| chr8 | 143252456 | 12 | 6 | chr8 | 143252455 | rs73375424 | A/G |
| chr8 | 143261517 | 2 | 5 | chr8 | 143261515 | rs7821413 | C/T |
| chr8 | 143261517 | 2 | 5 | chr8 | 143261518 | rs35518249 | -/G |
| chr8 | 143371401 | 5 | 4 | chr8 | 143371400 | rs4976994 | A/G |
| chr8 | 143402026 | 3 | 2 | chr8 | 143402024 | rs34132475 | C/T |
| chr8 | 143420556 | 2 | 8 | chr8 | 143420555 | rs7388259 | A/G |
| chr8 | 143437945 | 5 | 2 | chr8 | 143437944 | rs7842501 | A/G |
| chr8 | 143522824 | 6 | 3 | chr8 | 143522823 | rs10092920 | A/G |

| | | | | | | | |
|---|---|---|---|---|---|---|---|
| chr8 | 143693043 | 4 | 3 | chr8 | 143693042 | rs28427138 | A/G |
| chr8 | 143703786 | 2 | 6 | chr8 | 143703785 | rs28682551 | A/G |
| chr8 | 144356085 | 3 | 4 | chr8 | 144356083 | rs4075205 | A/G |
| chr8 | 144358120 | 0 | 0 | chr8 | 144358119 | rs62523430 | A/G |
| chr8 | 144358234 | 0 | 0 | chr8 | 144358233 | rs71518762 | A/G |
| chr8 | 144358348 | 0 | 0 | chr8 | 144358347 | rs71518763 | A/G |
| chr8 | 144358633 | 0 | 0 | chr8 | 144358632 | rs71518771 | G/T |
| chr8 | 144447543 | 2 | 11 | chr8 | 144447541 | rs11136282 | C/T |
| chr8 | 144517660 | 6 | 6 | chr8 | 144517659 | rs2467938 | A/G |
| chr8 | 144547573 | 4 | 1 | chr8 | 144547572 | rs10113635 | A/G |
| chr8 | 144549873 | 10 | 2 | chr8 | 144549872 | rs7813939 | A/G |
| chr8 | 144581611 | 1 | 3 | chr8 | 144581609 | rs1466622 | A/G |
| chr8 | 144589491 | 6 | 2 | chr8 | 144589490 | rs4874122 | A/G |
| chr8 | 144645054 | 2 | 3 | chr8 | 144645052 | rs388357 | G/T |
| chr8 | 144712897 | 3 | 3 | chr8 | 144712895 | rs4874151 | A/C |
| chr8 | 144721694 | 3 | 2 | chr8 | 144721692 | rs10101426 | C/T |
| chr8 | 144727488 | 8 | 1 | chr8 | 144727486 | rs4873805 | C/T |
| chr8 | 144800986 | 2 | 4 | chr8 | 144800985 | rs7827272 | A/G |
| chr8 | 144893333 | 4 | 2 | chr8 | 144893332 | rs7816354 | A/G |
| chr8 | 144966003 | 4 | 4 | chr8 | 144966002 | rs4875059 | G/T |
| chr8 | 145190636 | 2 | 3 | chr8 | 145190635 | rs7017102 | A/G |
| chr8 | 145538246 | 3 | 4 | chr8 | 145538245 | rs13254769 | A/G |
| chr8 | 145611219 | 1 | 8 | chr8 | 145611218 | rs2977838 | C/T |
| chr8 | 145995249 | 5 | 7 | chr8 | 145995249 | rs2955229 | A/C |
| chr8 | 146037223 | 1 | 3 | chr8 | 146037221 | rs2242650 | C/T |
| chr9 | 372372 | 1 | 5 | chr9 | 372371 | rs913702 | C/T |
| chr9 | 494297 | 3 | 2 | chr9 | 494297 | rs12344733 | C/G |
| chr9 | 507421 | 4 | 6 | chr9 | 507419 | rs7862091 | C/G |
| chr9 | 976085 | 1 | 7 | chr9 | 976084 | rs279880 | A/G |
| chr9 | 1032497 | 3 | 6 | chr9 | 1032496 | rs10116849 | A/G |
| chr9 | 1444419 | 2 | 3 | chr9 | 1444418 | rs10810152 | A/G |
| chr9 | 1629577 | 2 | 5 | chr9 | 1629576 | rs771888 | A/G |

| | | | | | | | |
|---|---|---|---|---|---|---|---|
| chr9 | 2215432 | 2 | 3 | chr9 | 2215429 | rs7873444 | C/G |
| chr9 | 2600308 | 2 | 3 | chr9 | 2600306 | rs1003047 | G/T |
| chr9 | 3254849 | 3 | 1 | chr9 | 3254847 | rs605946 | C/T |
| chr9 | 3697976 | 4 | 1 | chr9 | 3697975 | rs7858920 | A/G |
| chr9 | 3971735 | 6 | 1 | chr9 | 3971733 | rs641255 | A/G |
| chr9 | 4131357 | 5 | 1 | chr9 | 4131355 | rs67648867 | C/G |
| chr9 | 4319830 | 8 | 6 | chr9 | 4319828 | rs4336659 | C/T |
| chr9 | 4743234 | 3 | 1 | chr9 | 4743232 | rs296866 | C/T |
| chr9 | 4829512 | 3 | 1 | chr9 | 4829510 | rs6476915 | C/T |
| chr9 | 4954774 | 3 | 9 | chr9 | 4954773 | rs10974892 | A/G |
| chr9 | 5252349 | 1 | 5 | chr9 | 5252348 | rs12351715 | A/G |
| chr9 | 5833416 | 2 | 3 | chr9 | 5833415 | rs4532647 | G/T |
| chr9 | 6786168 | 2 | 3 | chr9 | 6786166 | rs7851954 | C/T |
| chr9 | 7049737 | 7 | 3 | chr9 | 7049735 | rs2381544 | C/T |
| chr9 | 8053984 | 4 | 6 | chr9 | 8053983 | rs1322467 | A/G |
| chr9 | 8065139 | 2 | 2 | chr9 | 8065138 | rs16927027 | A/G |
| chr9 | 8225563 | 1 | 4 | chr9 | 8225562 | rs6477283 | A/G |
| chr9 | 10344206 | 6 | 2 | chr9 | 10344204 | rs10121269 | C/T |
| chr9 | 10980842 | 4 | 5 | chr9 | 10980840 | rs1330259 | C/T |
| chr9 | 11001800 | 6 | 2 | chr9 | 11001799 | rs10756158 | A/G |
| chr9 | 11036998 | 1 | 5 | chr9 | 11036997 | rs4741092 | A/G |
| chr9 | 13754435 | 3 | 6 | chr9 | 13754433 | rs17805200 | C/T |
| chr9 | 14292735 | 3 | 1 | chr9 | 14292732 | rs36033212 | C/T |
| chr9 | 14292735 | 3 | 1 | chr9 | 14292734 | rs10810129 | C/G |
| chr9 | 14292735 | 3 | 1 | chr9 | 14292736 | rs34989278 | -/G |
| chr9 | 14372210 | 3 | 1 | chr9 | 14372208 | rs7036029 | C/T |
| chr9 | 15087020 | 3 | 6 | chr9 | 15087019 | rs7019934 | A/G |
| chr9 | 16648364 | 3 | 3 | chr9 | 16648363 | rs10756775 | A/G |
| chr9 | 18352775 | 3 | 15 | chr9 | 18352773 | rs7028195 | C/T |
| chr9 | 18948107 | 2 | 8 | chr9 | 18948106 | rs6475276 | A/G |
| chr9 | 20619337 | 1 | 4 | chr9 | 20619335 | rs2780838 | C/T |
| chr9 | 20712099 | 2 | 2 | chr9 | 20712097 | rs7027768 | C/T |

| | | | | | | | |
|---|---|---|---|---|---|---|---|
| chr9 | 21818111 | 1 | 5 | chr9 | 21818109 | rs3900787 | A/C |
| chr9 | 21913279 | 5 | 3 | chr9 | 21913278 | rs10811638 | A/G |
| chr9 | 21944953 | 2 | 3 | chr9 | 21944952 | rs10757261 | A/G |
| chr9 | 21958199 | 2 | 11 | chr9 | 21958198 | rs11515 | C/G |
| chr9 | 23727248 | 2 | 2 | chr9 | 23727247 | rs10811964 | A/G |
| chr9 | 24199720 | 3 | 2 | chr9 | 24199719 | rs17195843 | G/T |
| chr9 | 24285926 | 1 | 4 | chr9 | 24285925 | rs17197726 | G/T |
| chr9 | 25417466 | 4 | 17 | chr9 | 25417465 | rs1576035 | C/G |
| chr9 | 26763232 | 4 | 1 | chr9 | 26763230 | rs2383713 | C/T |
| chr9 | 27262582 | 1 | 3 | chr9 | 27262581 | rs1885378 | C/T |
| chr9 | 27564016 | 5 | 1 | chr9 | 27564014 | rs1373537 | C/T |
| chr9 | 27882290 | 2 | 4 | chr9 | 27882288 | rs7034687 | C/T |
| chr9 | 28965453 | 2 | 2 | chr9 | 28965451 | rs992962 | C/T |
| chr9 | 29021759 | 6 | 3 | chr9 | 29021759 | rs7026652 | A/G |
| chr9 | 29620811 | 1 | 3 | chr9 | 29620810 | rs7044847 | A/G |
| chr9 | 30789995 | 2 | 4 | chr9 | 30789994 | rs7031762 | C/G |
| chr9 | 31109647 | 1 | 4 | chr9 | 31109646 | rs10813469 | A/G |
| chr9 | 31812096 | 2 | 2 | chr9 | 31812094 | rs1980906 | A/C |
| chr9 | 34799931 | 2 | 3 | chr9 | 34799929 | rs928146 | C/G |
| chr9 | 36316287 | 7 | 2 | chr9 | 36316286 | rs7036467 | A/G |
| chr9 | 36706003 | 2 | 2 | chr9 | 36706002 | rs4880004 | A/G |
| chr9 | 36784531 | 5 | 1 | chr9 | 36784529 | rs2768621 | A/G |
| chr9 | 36918289 | 1 | 7 | chr9 | 36918287 | rs4880038 | C/T |
| chr9 | 36930902 | 8 | 2 | chr9 | 36930899 | rs7043783 | A/C |
| chr9 | 36946571 | 3 | 1 | chr9 | 36946570 | rs7028325 | A/G |
| chr9 | 36964991 | 5 | 1 | chr9 | 36964990 | rs10814495 | A/G |
| chr9 | 36993075 | 2 | 2 | chr9 | 36993074 | rs7852624 | A/G |
| chr9 | 37419661 | 2 | 3 | chr9 | 37419660 | rs309459 | A/G |
| chr9 | 37451223 | 3 | 1 | chr9 | 37451221 | rs2768656 | A/G |
| chr9 | 66195006 | 4 | 6 | chr9 | 66195004 | rs73447814 | C/T |
| chr9 | 66198180 | 3 | 7 | chr9 | 66198179 | rs2321644 | C/T |
| chr9 | 66578390 | 3 | 9 | chr9 | 66578389 | rs7046389 | A/C |

| | | | | | | | |
|---|---|---|---|---|---|---|---|
| chr9 | 67889619 | 4 | 2 | chr9 | 67889618 | rs4071885 | C/G |
| chr9 | 67903855 | 16 | 12 | chr9 | 67903852 | rs4928850 | A/G |
| chr9 | 67914215 | 1 | 3 | chr9 | 67914213 | rs67740384 | -/C |
| chr9 | 67921616 | 44 | 10 | chr9 | 67921614 | rs1872909 | C/T |
| chr9 | 67921616 | 44 | 10 | chr9 | 67921614 | rs62543328 | C/T |
| chr9 | 67921616 | 44 | 10 | chr9 | 67921615 | rs34419651 | A/G |
| chr9 | 70346383 | 1 | 11 | chr9 | 70346382 | rs7865055 | A/G |
| chr9 | 70620641 | 2 | 3 | chr9 | 70620640 | rs10115536 | A/G |
| chr9 | 70840572 | 2 | 5 | chr9 | 70840571 | rs2481598 | A/G |
| chr9 | 71149598 | 4 | 4 | chr9 | 71149597 | rs7021089 | G/T |
| chr9 | 71195419 | 4 | 3 | chr9 | 71195418 | rs11138412 | A/G |
| chr9 | 71928159 | 3 | 7 | chr9 | 71928158 | rs10511980 | A/G |
| chr9 | 73494326 | 3 | 1 | chr9 | 73494325 | rs4745114 | A/G |
| chr9 | 73583663 | 11 | 3 | chr9 | 73583662 | rs4745123 | A/G |
| chr9 | 73965457 | 2 | 3 | chr9 | 73965455 | rs10735614 | C/T |
| chr9 | 75046855 | 4 | 3 | chr9 | 75046853 | rs4400456 | C/T |
| chr9 | 75178220 | 2 | 3 | chr9 | 75178219 | rs7042358 | A/G |
| chr9 | 77014712 | 2 | 5 | chr9 | 77014710 | rs7018494 | C/T |
| chr9 | 77083747 | 3 | 1 | chr9 | 77083746 | rs72734988 | A/G |
| chr9 | 78656430 | 7 | 1 | chr9 | 78656429 | rs11145104 | A/G |
| chr9 | 79248856 | 1 | 4 | chr9 | 79248854 | rs7019154 | C/T |
| chr9 | 79665440 | 1 | 4 | chr9 | 79665437 | rs10781466 | C/G |
| chr9 | 79993293 | 2 | 2 | chr9 | 79993291 | rs62568309 | C/T |
| chr9 | 79993293 | 2 | 2 | chr9 | 79993292 | rs73653040 | A/G |
| chr9 | 80102217 | 9 | 2 | chr9 | 80102216 | rs10746571 | C/G |
| chr9 | 80778155 | 4 | 1 | chr9 | 80778153 | rs11137981 | C/T |
| chr9 | 81354825 | 4 | 2 | chr9 | 81354824 | rs13291796 | A/G |
| chr9 | 81377570 | 6 | 3 | chr9 | 81377569 | rs1934610 | C/T |
| chr9 | 82979214 | 4 | 1 | chr9 | 82979212 | rs2809841 | G/T |
| chr9 | 84707325 | 4 | 1 | chr9 | 84707324 | rs10117523 | A/G |
| chr9 | 85097336 | 4 | 3 | chr9 | 85097335 | rs10867977 | A/G |
| chr9 | 85342978 | 5 | 1 | chr9 | 85342978 | rs4529536 | G/T |

| | | | | | | | |
|---|---|---|---|---|---|---|---|
| chr9 | 86383285 | 2 | 2 | chr9 | 86383283 | rs2889960 | A/G |
| chr9 | 87129235 | 1 | 4 | chr9 | 87129233 | rs10780709 | C/T |
| chr9 | 88111535 | 1 | 3 | chr9 | 88111534 | rs2031264 | C/G |
| chr9 | 88316606 | 5 | 3 | chr9 | 88316605 | rs7029017 | A/G |
| chr9 | 88862868 | 3 | 1 | chr9 | 88862866 | rs4878040 | C/T |
| chr9 | 89081749 | 3 | 5 | chr9 | 89081748 | rs10868547 | A/G |
| chr9 | 89186386 | 1 | 3 | chr9 | 89186385 | rs1958938 | G/T |
| chr9 | 89629194 | 1 | 3 | chr9 | 89629193 | rs7039795 | A/G |
| chr9 | 90054553 | 0 | 0 | chr9 | 90054552 | rs10124913 | A/G |
| chr9 | 90772109 | 2 | 7 | chr9 | 90772107 | rs7025161 | C/T |
| chr9 | 91173984 | 1 | 10 | chr9 | 91173983 | rs11507706 | A/G |
| chr9 | 91459730 | 1 | 7 | chr9 | 91459728 | rs1467721 | A/G |
| chr9 | 91657833 | 3 | 4 | chr9 | 91657832 | rs9777444 | A/G |
| chr9 | 92373971 | 2 | 7 | chr9 | 92373970 | rs944552 | A/G |
| chr9 | 92565835 | 3 | 4 | chr9 | 92565834 | rs1173027 | C/T |
| chr9 | 92565850 | 3 | 5 | chr9 | 92565848 | rs4744003 | C/T |
| chr9 | 92565867 | 3 | 4 | chr9 | 92565866 | rs1172994 | C/T |
| chr9 | 92565882 | 3 | 5 | chr9 | 92565880 | rs4744004 | C/T |
| chr9 | 92565899 | 3 | 4 | chr9 | 92565898 | rs891165 | C/T |
| chr9 | 92565914 | 3 | 5 | chr9 | 92565912 | rs4744005 | C/T |
| chr9 | 92565931 | 3 | 4 | chr9 | 92565930 | rs1172992 | C/T |
| chr9 | 92565946 | 3 | 5 | chr9 | 92565944 | rs4744006 | C/T |
| chr9 | 92565963 | 3 | 4 | chr9 | 92565962 | rs1173024 | C/T |
| chr9 | 92565978 | 3 | 5 | chr9 | 92565976 | rs4744007 | C/T |
| chr9 | 92565995 | 3 | 4 | chr9 | 92565994 | rs4002855 | A/G |
| chr9 | 92566010 | 3 | 5 | chr9 | 92566008 | rs4744008 | C/T |
| chr9 | 92566010 | 3 | 5 | chr9 | 92566008 | rs59727661 | -/CGGTC |
| chr9 | 92566010 | 3 | 5 | chr9 | 92566011 | rs57028349 | -/ATGAAAGACCAGATGTCTTTCATCCGG |
| chr9 | 92566027 | 3 | 5 | chr9 | 92566026 | rs1148851 | C/T |
| chr9 | 93382928 | 2 | 3 | chr9 | 93382927 | rs9409431 | A/G |
| chr9 | 93542660 | 1 | 3 | chr9 | 93542658 | rs4604505 | C/G |
| chr9 | 95095766 | 1 | 6 | chr9 | 95095764 | rs12683662 | C/T |

| | | | | | | | |
|---|---|---|---|---|---|---|---|
| chr9 | 95392887 | 3 | 6 | chr9 | 95392886 | rs2150751 | A/G |
| chr9 | 95741484 | 2 | 2 | chr9 | 95741483 | rs10992997 | A/G |
| chr9 | 96815801 | 4 | 19 | chr9 | 96815799 | rs6479589 | C/T |
| chr9 | 98841696 | 5 | 2 | chr9 | 98841695 | rs7035822 | A/G |
| chr9 | 99894585 | 1 | 3 | chr9 | 99894585 | rs10818475 | C/G |
| chr9 | 100115911 | 2 | 3 | chr9 | 100115908 | rs12345664 | A/C |
| chr9 | 100320554 | 4 | 3 | chr9 | 100320551 | rs2779571 | C/T |
| chr9 | 100373274 | 19 | 1 | chr9 | 100373273 | rs928425 | A/G |
| chr9 | 100374377 | 2 | 2 | chr9 | 100374376 | rs1167769 | A/G |
| chr9 | 100440044 | 2 | 2 | chr9 | 100440042 | rs4743242 | C/T |
| chr9 | 100460547 | 3 | 1 | chr9 | 100460545 | rs1571929 | A/G |
| chr9 | 100649121 | 2 | 2 | chr9 | 100649119 | rs10739719 | C/T |
| chr9 | 102616210 | 11 | 9 | chr9 | 102616208 | rs10819844 | A/C |
| chr9 | 102618702 | 2 | 3 | chr9 | 102618700 | rs1538413 | C/T |
| chr9 | 102717485 | 5 | 5 | chr9 | 102717484 | rs10739811 | A/G |
| chr9 | 103151460 | 4 | 1 | chr9 | 103151458 | rs7026000 | C/T |
| chr9 | 104163054 | 4 | 3 | chr9 | 104163053 | rs4743542 | A/G |
| chr9 | 106785574 | 2 | 5 | chr9 | 106785573 | rs2028093 | A/G |
| chr9 | 109103019 | 1 | 4 | chr9 | 109103017 | rs11573662 | C/T |
| chr9 | 109206990 | 1 | 5 | chr9 | 109206988 | rs9299131 | C/T |
| chr9 | 110317628 | 5 | 4 | chr9 | 110317626 | rs7033555 | C/T |
| chr9 | 110326639 | 1 | 8 | chr9 | 110326637 | rs11794355 | A/C |
| chr9 | 110907112 | 1 | 3 | chr9 | 110907110 | rs12683988 | C/T |
| chr9 | 110981679 | 4 | 4 | chr9 | 110981677 | rs908859 | A/G |
| chr9 | 111619034 | 5 | 1 | chr9 | 111619033 | rs10816884 | A/G |
| chr9 | 111872148 | 6 | 2 | chr9 | 111872146 | rs491533 | C/T |
| chr9 | 112144167 | 4 | 2 | chr9 | 112144166 | rs62569742 | A/G |
| chr9 | 112632393 | 2 | 3 | chr9 | 112632391 | rs2767005 | C/T |
| chr9 | 113049149 | 5 | 1 | chr9 | 113049147 | rs7029557 | C/T |
| chr9 | 113918612 | 2 | 2 | chr9 | 113918611 | rs4978474 | A/G |
| chr9 | 114183334 | 2 | 5 | chr9 | 114183333 | rs10759556 | A/G |
| chr9 | 114347785 | 5 | 1 | chr9 | 114347783 | rs6477950 | C/T |

| | | | | | | | |
|---|---|---|---|---|---|---|---|
| chr9 | 114568577 | 3 | 3 | chr9 | 114568575 | rs2796028 | A/G |
| chr9 | 114709702 | 2 | 3 | chr9 | 114709700 | rs912662 | G/T |
| chr9 | 115571140 | 5 | 1 | chr9 | 115571138 | rs6478034 | C/T |
| chr9 | 115890897 | 2 | 6 | chr9 | 115890896 | rs2093785 | A/G |
| chr9 | 116109045 | 2 | 3 | chr9 | 116109043 | rs1249761 | A/G |
| chr9 | 116269221 | 2 | 2 | chr9 | 116269220 | rs1535971 | G/T |
| chr9 | 116285781 | 3 | 1 | chr9 | 116285780 | rs7865843 | A/G |
| chr9 | 116299360 | 3 | 2 | chr9 | 116299358 | rs10817628 | A/C |
| chr9 | 116437590 | 3 | 1 | chr9 | 116437588 | rs7855505 | C/T |
| chr9 | 116802406 | 0 | 0 | chr9 | 116802405 | rs35583826 | -/G |
| chr9 | 116802406 | 0 | 0 | chr9 | 116802406 | rs5900111 | -/G |
| chr9 | 117303698 | 10 | 1 | chr9 | 117303696 | rs10739447 | A/C |
| chr9 | 118089383 | 3 | 1 | chr9 | 118089381 | rs7875237 | C/T |
| chr9 | 118169647 | 3 | 3 | chr9 | 118169645 | rs57480127 | C/T |
| chr9 | 118169647 | 3 | 3 | chr9 | 118169646 | rs7020762 | A/G |
| chr9 | 118697529 | 2 | 2 | chr9 | 118697528 | rs3903957 | A/G |
| chr9 | 118822203 | 1 | 9 | chr9 | 118822201 | rs1334091 | C/T |
| chr9 | 119108341 | 3 | 1 | chr9 | 119108340 | rs7022764 | A/G |
| chr9 | 119575166 | 3 | 2 | chr9 | 119575165 | rs7357627 | A/G |
| chr9 | 119891401 | 2 | 2 | chr9 | 119891399 | rs2809380 | C/T |
| chr9 | 121339968 | 5 | 1 | chr9 | 121339966 | rs1331608 | C/T |
| chr9 | 121850698 | 2 | 3 | chr9 | 121850696 | rs4836798 | C/T |
| chr9 | 121994465 | 4 | 6 | chr9 | 121994464 | rs4837730 | A/G |
| chr9 | 122045330 | 5 | 1 | chr9 | 122045328 | rs4997435 | A/C |
| chr9 | 123374373 | 4 | 2 | chr9 | 123374372 | rs645650 | C/T |
| chr9 | 123559035 | 3 | 1 | chr9 | 123559034 | rs55835505 | A/G |
| chr9 | 123617720 | 8 | 5 | chr9 | 123617717 | rs6478528 | C/G |
| chr9 | 124173301 | 2 | 2 | chr9 | 124173299 | rs1236913 | C/T |
| chr9 | 125204384 | 2 | 2 | chr9 | 125204382 | rs10739631 | C/T |
| chr9 | 125831826 | 2 | 3 | chr9 | 125831825 | rs10760309 | A/G |
| chr9 | 126009216 | 2 | 5 | chr9 | 126009214 | rs7032624 | C/T |
| chr9 | 126044861 | 1 | 6 | chr9 | 126044861 | rs10986285 | A/G |

| | | | | | | | |
|---|---|---|---|---|---|---|---|
| chr9 | 126055573 | 4 | 4 | chr9 | 126055572 | rs10760343 | A/G |
| chr9 | 126107184 | 3 | 3 | chr9 | 126107183 | rs10739647 | A/G |
| chr9 | 126141448 | 4 | 1 | chr9 | 126141446 | rs942277 | C/T |
| chr9 | 126273028 | 4 | 5 | chr9 | 126273027 | rs1886124 | A/G |
| chr9 | 126598192 | 10 | 2 | chr9 | 126598190 | rs4836993 | C/G |
| chr9 | 126623206 | 2 | 13 | chr9 | 126623205 | rs10818990 | A/G |
| chr9 | 126655816 | 9 | 3 | chr9 | 126655815 | rs3814131 | C/T |
| chr9 | 126948127 | 1 | 3 | chr9 | 126948126 | rs2113352 | G/T |
| chr9 | 127349443 | 13 | 1 | chr9 | 127349441 | rs527969 | C/T |
| chr9 | 127474123 | 17 | 2 | chr9 | 127474122 | rs479595 | A/G |
| chr9 | 127794056 | 0 | 0 | chr9 | 127794054 | rs2149990 | A/G |
| chr9 | 127882539 | 5 | 2 | chr9 | 127882538 | rs4838332 | A/G |
| chr9 | 128216551 | 7 | 2 | chr9 | 128216550 | rs10760432 | C/G |
| chr9 | 128232499 | 1 | 12 | chr9 | 128232497 | rs12351417 | C/T |
| chr9 | 128309831 | 3 | 11 | chr9 | 128309829 | rs929561 | A/G |
| chr9 | 128417848 | 8 | 1 | chr9 | 128417846 | rs945686 | C/G |
| chr9 | 128435386 | 2 | 3 | chr9 | 128435384 | rs12378303 | C/T |
| chr9 | 129162838 | 3 | 1 | chr9 | 129162837 | rs10987612 | A/G |
| chr9 | 129572257 | 1 | 3 | chr9 | 129572256 | rs869386 | A/G |
| chr9 | 129708778 | 5 | 1 | chr9 | 129708777 | rs10819317 | A/G |
| chr9 | 129939052 | 5 | 4 | chr9 | 129939051 | rs73615701 | A/G |
| chr9 | 129954255 | 1 | 4 | chr9 | 129954254 | rs2232629 | A/G |
| chr9 | 129956590 | 5 | 1 | chr9 | 129956589 | rs4443758 | A/G |
| chr9 | 130694990 | 4 | 7 | chr9 | 130694988 | rs4837329 | C/T |
| chr9 | 131663327 | 3 | 13 | chr9 | 131663325 | rs10760639 | C/T |
| chr9 | 131670688 | 0 | 0 | chr9 | 131670686 | rs7033280 | C/T |
| chr9 | 131697778 | 6 | 1 | chr9 | 131697777 | rs2047836 | A/G |
| chr9 | 131930938 | 5 | 1 | chr9 | 131930935 | rs7853219 | C/T |
| chr9 | 132002461 | 4 | 7 | chr9 | 132002461 | rs62585969 | C/G |
| chr9 | 132086433 | 0 | 0 | chr9 | 132086432 | rs7043286 | A/G |
| chr9 | 132349304 | 1 | 4 | chr9 | 132349302 | rs529569 | A/G |
| chr9 | 132366622 | 4 | 2 | chr9 | 132366620 | rs688430 | A/G |

| | | | | | | | |
|---|---|---|---|---|---|---|---|
| chr9 | 132382862 | 9 | 2 | chr9 | 132382862 | rs610599 | C/T |
| chr9 | 132388869 | 2 | 6 | chr9 | 132388868 | rs11243628 | A/G |
| chr9 | 132778272 | 2 | 3 | chr9 | 132778270 | rs10113902 | C/T |
| chr9 | 132778416 | 3 | 4 | chr9 | 132778415 | rs10125388 | A/G |
| chr9 | 132884608 | 3 | 1 | chr9 | 132884607 | rs7868734 | C/G |
| chr9 | 132985103 | 1 | 15 | chr9 | 132985102 | rs353515 | C/T |
| chr9 | 133441015 | 1 | 4 | chr9 | 133441013 | rs943852 | A/G |
| chr9 | 133601448 | 1 | 4 | chr9 | 133601447 | rs928013 | A/G |
| chr9 | 133620916 | 1 | 5 | chr9 | 133620915 | rs3012771 | A/G |
| chr9 | 133656033 | 2 | 3 | chr9 | 133656031 | rs2987361 | C/T |
| chr9 | 134326661 | 3 | 1 | chr9 | 134326659 | rs558731 | C/T |
| chr9 | 134591176 | 3 | 3 | chr9 | 134591174 | rs396819 | A/G |
| chr9 | 134652956 | 3 | 12 | chr9 | 134652954 | rs215157 | G/T |
| chr9 | 134653987 | 4 | 11 | chr9 | 134653986 | rs2772008 | G/T |
| chr9 | 134712299 | 0 | 0 | chr9 | 134712298 | rs2809257 | A/G |
| chr9 | 134992056 | 2 | 2 | chr9 | 134992054 | rs576303 | C/T |
| chr9 | 135402831 | 1 | 7 | chr9 | 135402831 | rs10121435 | A/G |
| chr9 | 135403773 | 4 | 6 | chr9 | 135403772 | rs9776782 | A/G |
| chr9 | 135453678 | 2 | 4 | chr9 | 135453677 | rs12380304 | A/G |
| chr9 | 135563648 | 1 | 6 | chr9 | 135563647 | rs2427987 | C/T |
| chr9 | 135631426 | 6 | 3 | chr9 | 135631425 | rs2510255 | A/G |
| chr9 | 135633442 | 3 | 3 | chr9 | 135633440 | rs2510259 | C/T |
| chr9 | 135743958 | 2 | 7 | chr9 | 135743956 | rs2486353 | C/T |
| chr9 | 135771249 | 8 | 7 | chr9 | 135771248 | rs2789833 | C/G |
| chr9 | 135775694 | 4 | 3 | chr9 | 135775692 | rs2810508 | C/T |
| chr9 | 135801568 | 5 | 2 | chr9 | 135801567 | rs2519112 | A/G |
| chr9 | 135814825 | 1 | 6 | chr9 | 135814823 | rs2789862 | C/T |
| chr9 | 135916918 | 4 | 1 | chr9 | 135916917 | rs2506713 | A/G |
| chr9 | 135919957 | 1 | 5 | chr9 | 135919954 | rs11795079 | C/T |
| chr9 | 136303811 | 1 | 7 | chr9 | 136303809 | rs10156443 | C/T |
| chr9 | 136303971 | 4 | 2 | chr9 | 136303969 | rs1930789 | C/T |
| chr9 | 136519220 | 2 | 3 | chr9 | 136519218 | rs7860818 | C/T |

| | | | | | | | |
|---|---|---|---|---|---|---|---|
| chr9 | 136694502 | 2 | 6 | chr9 | 136694500 | rs10046876 | C/G |
| chr9 | 136730648 | 4 | 9 | chr9 | 136730647 | rs4401948 | C/G |
| chr9 | 136817860 | 1 | 3 | chr9 | 136817858 | rs3124934 | C/T |
| chr9 | 136858018 | 10 | 1 | chr9 | 136858016 | rs4841937 | C/T |
| chr9 | 136992823 | 3 | 6 | chr9 | 136992821 | rs4842200 | C/T |
| chr9 | 137153838 | 2 | 6 | chr9 | 137153837 | rs7039212 | A/G |
| chr9 | 137297206 | 1 | 4 | chr9 | 137297203 | rs2382861 | C/G |
| chr9 | 137361624 | 2 | 3 | chr9 | 137361622 | rs11103821 | C/T |
| chr9 | 137374640 | 0 | 0 | chr9 | 137374638 | rs4240714 | C/T |
| chr9 | 137488212 | 2 | 3 | chr9 | 137488211 | rs10858112 | G/T |
| chr9 | 137518823 | 6 | 5 | chr9 | 137518822 | rs1975518 | G/T |
| chr9 | 137636889 | 5 | 4 | chr9 | 137636887 | rs1572049 | A/G |
| chr9 | 137705865 | 2 | 2 | chr9 | 137705863 | rs560742 | C/T |
| chr9 | 137773624 | 1 | 8 | chr9 | 137773622 | rs10858161 | C/T |
| chr9 | 137779290 | 3 | 1 | chr9 | 137779289 | rs11103155 | A/G |
| chr9 | 137956484 | 2 | 3 | chr9 | 137956482 | rs4842045 | C/T |
| chr9 | 138018550 | 4 | 7 | chr9 | 138018547 | rs2152175 | C/T |
| chr9 | 138024775 | 2 | 2 | chr9 | 138024774 | rs4273927 | A/G |
| chr9 | 138090438 | 7 | 1 | chr9 | 138090437 | rs11103293 | A/G |
| chr9 | 138090835 | 3 | 1 | chr9 | 138090833 | rs11103294 | C/T |
| chr9 | 138140233 | 1 | 4 | chr9 | 138140232 | rs3811128 | C/G |
| chr9 | 138164053 | 0 | 0 | chr9 | 138164052 | rs7021573 | A/G |
| chr9 | 138175430 | 3 | 1 | chr9 | 138175429 | rs11103350 | G/T |
| chr9 | 138192102 | 0 | 0 | chr9 | 138192100 | rs7044581 | A/C |
| chr9 | 138202396 | 1 | 6 | chr9 | 138202395 | rs10858235 | A/G |
| chr9 | 138448373 | 1 | 4 | chr9 | 138448371 | rs10870199 | C/T |
| chr9 | 138507439 | 1 | 3 | chr9 | 138507438 | rs7856092 | A/G |
| chr9 | 138522485 | 4 | 1 | chr9 | 138522483 | rs3124597 | C/T |
| chr9 | 138530245 | 3 | 3 | chr9 | 138530244 | rs3125006 | A/G |
| chr9 | 138569384 | 2 | 7 | chr9 | 138569382 | rs1571987 | A/G |
| chr9 | 138569846 | 2 | 6 | chr9 | 138569845 | rs7854561 | A/G |
| chr9 | 138569973 | 5 | 2 | chr9 | 138569972 | rs6560634 | A/G |

| | | | | | | | |
|---|---|---|---|---|---|---|---|
| chr9 | 138692497 | 4 | 5 | chr9 | 138692495 | rs4880063 | C/T |
| chr9 | 138742687 | 1 | 3 | chr9 | 138742686 | rs9411222 | A/G |
| chr9 | 138841335 | 2 | 4 | chr9 | 138841333 | rs2811751 | A/G |
| chr9 | 139093058 | 2 | 3 | chr9 | 139093056 | rs7873997 | C/T |
| chr9 | 139589940 | 5 | 1 | chr9 | 139589939 | rs863701 | A/G |
| chr9 | 139695463 | 4 | 4 | chr9 | 139695461 | rs2480110 | C/T |
| chr9 | 139731258 | 3 | 5 | chr9 | 139731256 | rs3812497 | C/T |
| chr9 | 139787048 | 4 | 3 | chr9 | 139787047 | rs4247463 | A/G |
| chr9 | 140131592 | 3 | 2 | chr9 | 140131591 | rs2606358 | A/G |
| chr9 | 140158098 | 1 | 5 | chr9 | 140158097 | rs28521831 | A/G |
| chr10 | 504034 | 4 | 3 | chr10 | 504031 | rs2066314 | C/G |
| chr10 | 674670 | 2 | 2 | chr10 | 674669 | rs816575 | A/G |
| chr10 | 912976 | 4 | 1 | chr10 | 912974 | rs947404 | A/C |
| chr10 | 1006748 | 13 | 2 | chr10 | 1006747 | rs947402 | C/T |
| chr10 | 1415169 | 3 | 1 | chr10 | 1415167 | rs2805510 | C/T |
| chr10 | 1445545 | 2 | 3 | chr10 | 1445542 | rs2255595 | C/T |
| chr10 | 1501151 | 11 | 2 | chr10 | 1501149 | rs4880509 | C/G |
| chr10 | 1567563 | 1 | 3 | chr10 | 1567562 | rs2676732 | C/T |
| chr10 | 1583891 | 4 | 4 | chr10 | 1583890 | rs4880869 | A/G |
| chr10 | 1584335 | 4 | 1 | chr10 | 1584333 | rs7070465 | C/T |
| chr10 | 1585302 | 5 | 1 | chr10 | 1585301 | rs4620649 | A/G |
| chr10 | 1655031 | 4 | 4 | chr10 | 1655028 | rs10794773 | C/T |
| chr10 | 1677798 | 1 | 3 | chr10 | 1677798 | rs2173524 | G/T |
| chr10 | 1718841 | 3 | 8 | chr10 | 1718839 | rs4880911 | C/T |
| chr10 | 2558498 | 3 | 2 | chr10 | 2558496 | rs55699572 | C/T |
| chr10 | 2725176 | 4 | 1 | chr10 | 2725175 | rs2050344 | A/G |
| chr10 | 2832783 | 6 | 1 | chr10 | 2832781 | rs1999647 | C/T |
| chr10 | 3090322 | 2 | 2 | chr10 | 3090321 | rs10736965 | A/G |
| chr10 | 3260103 | 1 | 7 | chr10 | 3260101 | rs2477764 | A/G |
| chr10 | 3376999 | 2 | 2 | chr10 | 3376997 | rs10736969 | C/T |
| chr10 | 3939993 | 3 | 11 | chr10 | 3939992 | rs4457648 | A/G |
| chr10 | 3959813 | 3 | 1 | chr10 | 3959811 | rs4504967 | C/T |

| | | | | | | | |
|---|---|---|---|---|---|---|---|
| chr10 | 3994343 | 4 | 4 | chr10 | 3994342 | rs2025680 | C/T |
| chr10 | 4241995 | 3 | 2 | chr10 | 4241993 | rs7909661 | A/C |
| chr10 | 4342318 | 1 | 9 | chr10 | 4342317 | rs4579834 | A/G |
| chr10 | 4488602 | 5 | 4 | chr10 | 4488601 | rs1751298 | A/G |
| chr10 | 4878140 | 1 | 4 | chr10 | 4878139 | rs2397986 | C/T |
| chr10 | 5596650 | 2 | 10 | chr10 | 5596648 | rs4880734 | C/T |
| chr10 | 5736495 | 9 | 1 | chr10 | 5736493 | rs3750639 | A/G |
| chr10 | 5955632 | 3 | 1 | chr10 | 5955630 | rs626040 | C/T |
| chr10 | 6070755 | 1 | 3 | chr10 | 6070754 | rs10795726 | A/G |
| chr10 | 6298058 | 0 | 0 | chr10 | 6298057 | rs2148294 | A/G |
| chr10 | 6304117 | 6 | 10 | chr10 | 6304115 | rs2025720 | C/T |
| chr10 | 6326611 | 2 | 3 | chr10 | 6326611 | rs4750166 | A/G |
| chr10 | 7189861 | 1 | 3 | chr10 | 7189859 | rs2462698 | C/T |
| chr10 | 7546484 | 4 | 1 | chr10 | 7546482 | rs4748937 | C/T |
| chr10 | 7546484 | 4 | 1 | chr10 | 7546482 | rs35231143 | CG/TA |
| chr10 | 7546484 | 4 | 1 | chr10 | 7546483 | rs4748938 | A/G |
| chr10 | 7623618 | 5 | 7 | chr10 | 7623616 | rs4749002 | C/T |
| chr10 | 8489461 | 6 | 1 | chr10 | 8489460 | rs2646414 | A/G |
| chr10 | 8595535 | 4 | 3 | chr10 | 8595534 | rs7100251 | C/G |
| chr10 | 10615843 | 3 | 2 | chr10 | 10615841 | rs1324319 | A/G |
| chr10 | 11368721 | 3 | 4 | chr10 | 11368720 | rs10905926 | A/G |
| chr10 | 11786915 | 1 | 10 | chr10 | 11786914 | rs11257267 | A/G |
| chr10 | 11801817 | 3 | 5 | chr10 | 11801815 | rs6602517 | C/T |
| chr10 | 11820303 | 2 | 5 | chr10 | 11820302 | rs10795882 | A/G |
| chr10 | 11869715 | 1 | 5 | chr10 | 11869712 | rs7084173 | C/T |
| chr10 | 11933859 | 3 | 7 | chr10 | 11933857 | rs7079574 | C/T |
| chr10 | 11960709 | 1 | 3 | chr10 | 11960708 | rs7095018 | A/G |
| chr10 | 11984182 | 1 | 6 | chr10 | 11984181 | rs11257407 | A/G |
| chr10 | 12240412 | 2 | 4 | chr10 | 12240411 | rs4589194 | A/G |
| chr10 | 12284521 | 3 | 4 | chr10 | 12284519 | rs4264073 | C/T |
| chr10 | 12461297 | 7 | 2 | chr10 | 12461296 | rs4747979 | A/G |
| chr10 | 12815730 | 7 | 10 | chr10 | 12815728 | rs4747998 | A/C |

| | | | | | | | |
|---|---|---|---|---|---|---|---|
| chr10 | 12848284 | 2 | 2 | chr10 | 12848282 | rs2493777 | A/G |
| chr10 | 13381844 | 5 | 6 | chr10 | 13381841 | rs511959 | C/G |
| chr10 | 13418075 | 3 | 1 | chr10 | 13418073 | rs7894075 | A/C |
| chr10 | 13551993 | 7 | 1 | chr10 | 13551991 | rs4748035 | C/T |
| chr10 | 13857402 | 2 | 5 | chr10 | 13857401 | rs10737081 | A/G |
| chr10 | 13893858 | 3 | 1 | chr10 | 13893857 | rs7088455 | A/G |
| chr10 | 17201247 | 3 | 2 | chr10 | 17201245 | rs7898283 | C/T |
| chr10 | 18611417 | 1 | 5 | chr10 | 18611416 | rs11013317 | A/G |
| chr10 | 19800515 | 1 | 8 | chr10 | 19800514 | rs10764029 | A/G |
| chr10 | 20553568 | 3 | 1 | chr10 | 20553567 | rs10764215 | A/G |
| chr10 | 20626750 | 4 | 6 | chr10 | 20626748 | rs10740971 | C/T |
| chr10 | 24892407 | 5 | 6 | chr10 | 24892405 | rs10764479 | C/T |
| chr10 | 26249341 | 1 | 3 | chr10 | 26249340 | rs1934448 | C/T |
| chr10 | 26677264 | 1 | 4 | chr10 | 26677263 | rs3006845 | A/G |
| chr10 | 27176179 | 1 | 7 | chr10 | 27176178 | rs11015338 | A/G |
| chr10 | 27828898 | 10 | 1 | chr10 | 27828897 | rs2477340 | A/G |
| chr10 | 27991029 | 3 | 1 | chr10 | 27991027 | rs2637282 | A/G |
| chr10 | 28493232 | 2 | 2 | chr10 | 28493230 | rs1781834 | C/T |
| chr10 | 28657425 | 1 | 7 | chr10 | 28657424 | rs1148185 | C/T |
| chr10 | 28660150 | 3 | 2 | chr10 | 28660148 | rs1249269 | A/G |
| chr10 | 29265293 | 0 | 0 | chr10 | 29265291 | rs6481552 | C/T |
| chr10 | 29404712 | 1 | 3 | chr10 | 29404710 | rs4749388 | C/T |
| chr10 | 29586479 | 3 | 2 | chr10 | 29586478 | rs1860403 | C/T |
| chr10 | 29845602 | 2 | 4 | chr10 | 29845600 | rs3780844 | A/G |
| chr10 | 30250488 | 1 | 3 | chr10 | 30250486 | rs2096159 | A/G |
| chr10 | 31184228 | 1 | 5 | chr10 | 31184226 | rs923902 | A/G |
| chr10 | 32386714 | 1 | 4 | chr10 | 32386713 | rs12415335 | A/G |
| chr10 | 32754345 | 2 | 3 | chr10 | 32754343 | rs2505389 | C/T |
| chr10 | 34202089 | 5 | 1 | chr10 | 34202088 | rs2800812 | A/G |
| chr10 | 35855907 | 1 | 4 | chr10 | 35855906 | rs2474742 | C/G |
| chr10 | 36493915 | 1 | 8 | chr10 | 36493914 | rs315647 | C/T |
| chr10 | 41714496 | 5 | 2 | chr10 | 41714494 | rs12049687 | C/T |

| | | | | | | | |
|---|---|---|---|---|---|---|---|
| chr10 | 42894599 | 3 | 1 | chr10 | 42894597 | rs2435365 | A/G |
| chr10 | 42917095 | 2 | 2 | chr10 | 42917093 | rs2472738 | C/T |
| chr10 | 43078462 | 2 | 3 | chr10 | 43078460 | rs869184 | A/C/G/T |
| chr10 | 43166936 | 5 | 1 | chr10 | 43166934 | rs2460561 | A/G |
| chr10 | 43236925 | 11 | 2 | chr10 | 43236923 | rs2066043 | A/G |
| chr10 | 43569244 | 10 | 7 | chr10 | 43569242 | rs11238689 | A/C |
| chr10 | 43824366 | 1 | 10 | chr10 | 43824364 | rs4948595 | C/T |
| chr10 | 44080964 | 1 | 4 | chr10 | 44080963 | rs620828 | C/T |
| chr10 | 44173117 | 3 | 5 | chr10 | 44173115 | rs1144474 | C/T |
| chr10 | 44190247 | 0 | 0 | chr10 | 44190245 | rs197452 | C/T |
| chr10 | 44696301 | 2 | 2 | chr10 | 44696299 | rs11239271 | C/T |
| chr10 | 44791429 | 0 | 0 | chr10 | 44791427 | rs914701 | C/T |
| chr10 | 44851923 | 3 | 1 | chr10 | 44851921 | rs12782937 | C/T |
| chr10 | 46403937 | 11 | 3 | chr10 | 46403936 | rs1414563 | A/C |
| chr10 | 46412067 | 13 | 1 | chr10 | 46412066 | rs2105088 | C/T |
| chr10 | 46413038 | 13 | 5 | chr10 | 46413036 | rs9732469 | C/T |
| chr10 | 46420869 | 6 | 4 | chr10 | 46420868 | rs3127686 | A/G |
| chr10 | 46463855 | 2 | 5 | chr10 | 46463852 | rs9330498 | C/G |
| chr10 | 46531537 | 1 | 11 | chr10 | 46531535 | rs4375381 | A/C |
| chr10 | 46542419 | 1 | 4 | chr10 | 46542417 | rs28368414 | C/T |
| chr10 | 46542419 | 1 | 4 | chr10 | 46542418 | rs6599600 | A/G |
| chr10 | 48070399 | 2 | 2 | chr10 | 48070397 | rs35644500 | C/T |
| chr10 | 48114343 | 3 | 2 | chr10 | 48114341 | rs11204238 | A/C |
| chr10 | 49335141 | 1 | 3 | chr10 | 49335138 | rs10857573 | C/G |
| chr10 | 49335141 | 1 | 3 | chr10 | 49335138 | rs71185252 | CC/GA |
| chr10 | 49335141 | 1 | 3 | chr10 | 49335139 | rs10776602 | A/C |
| chr10 | 49337802 | 5 | 6 | chr10 | 49337800 | rs3853761 | C/T |
| chr10 | 49370436 | 5 | 2 | chr10 | 49370434 | rs4838606 | C/T |
| chr10 | 50430440 | 2 | 3 | chr10 | 50430439 | rs7902661 | A/G |
| chr10 | 50590562 | 7 | 2 | chr10 | 50590561 | rs1916564 | C/T |
| chr10 | 52906029 | 3 | 10 | chr10 | 52906028 | rs10997716 | A/G |
| chr10 | 53304364 | 2 | 4 | chr10 | 53304362 | rs2339888 | A/G |

| | | | | | | | |
|---|---|---|---|---|---|---|---|
| chr10 | 55490709 | 3 | 3 | chr10 | 55490707 | rs4418714 | C/T |
| chr10 | 55818964 | 4 | 1 | chr10 | 55818963 | rs10825317 | A/G |
| chr10 | 56024390 | 2 | 2 | chr10 | 56024388 | rs1937407 | A/G |
| chr10 | 56429366 | 1 | 3 | chr10 | 56429364 | rs4317894 | C/T |
| chr10 | 56812969 | 9 | 4 | chr10 | 56812968 | rs1900489 | C/T |
| chr10 | 57058069 | 1 | 3 | chr10 | 57058068 | rs2463947 | A/G |
| chr10 | 57272765 | 2 | 2 | chr10 | 57272763 | rs11005066 | C/T |
| chr10 | 58122227 | 5 | 1 | chr10 | 58122226 | rs12775593 | A/G |
| chr10 | 59152866 | 4 | 1 | chr10 | 59152864 | rs10740711 | C/T |
| chr10 | 59152866 | 4 | 1 | chr10 | 59152865 | rs10733974 | A/CA/G/TG |
| chr10 | 59152866 | 4 | 1 | chr10 | 59152865 | rs63359261 | A/G |
| chr10 | 59512613 | 1 | 3 | chr10 | 59512612 | rs1626909 | A/G |
| chr10 | 59518867 | 6 | 1 | chr10 | 59518865 | rs1759343 | C/T |
| chr10 | 59518867 | 6 | 1 | chr10 | 59518867 | rs1623687 | A/G |
| chr10 | 60730835 | 7 | 1 | chr10 | 60730833 | rs284598 | C/T |
| chr10 | 62133245 | 1 | 3 | chr10 | 62133242 | rs7895268 | C/T |
| chr10 | 62133245 | 1 | 3 | chr10 | 62133243 | rs7909972 | C/T |
| chr10 | 62567741 | 1 | 3 | chr10 | 62567740 | rs7099382 | A/G |
| chr10 | 63975395 | 2 | 21 | chr10 | 63975394 | rs10822021 | A/G |
| chr10 | 64031706 | 3 | 1 | chr10 | 64031705 | rs10822031 | A/G |
| chr10 | 64057941 | 3 | 1 | chr10 | 64057939 | rs2893906 | A/G |
| chr10 | 64340299 | 3 | 2 | chr10 | 64340298 | rs7915640 | A/G |
| chr10 | 64474032 | 7 | 3 | chr10 | 64474031 | rs59153379 | A/G |
| chr10 | 67675047 | 3 | 1 | chr10 | 67675046 | rs10822776 | A/G |
| chr10 | 68619584 | 3 | 2 | chr10 | 68619583 | rs7073762 | A/G |
| chr10 | 69666461 | 2 | 3 | chr10 | 69666460 | rs4745950 | A/G |
| chr10 | 69666630 | 11 | 5 | chr10 | 69666629 | rs4745951 | A/G |
| chr10 | 70420999 | 2 | 16 | chr10 | 70420998 | rs2255850 | A/G |
| chr10 | 70726334 | 2 | 2 | chr10 | 70726332 | rs7914285 | C/T |
| chr10 | 70726334 | 2 | 2 | chr10 | 70726333 | rs12263326 | A/G |
| chr10 | 71718900 | 4 | 2 | chr10 | 71718899 | rs12780221 | A/G |
| chr10 | 71733935 | 4 | 2 | chr10 | 71733933 | rs10762362 | C/T |

| | | | | | | | |
|---|---|---|---|---|---|---|---|
| chr10 | 72127256 | 3 | 2 | chr10 | 72127255 | rs11599210 | A/G |
| chr10 | 72135147 | 3 | 2 | chr10 | 72135145 | rs11596392 | C/G |
| chr10 | 72231199 | 2 | 3 | chr10 | 72231199 | rs2461884 | C/G |
| chr10 | 72341116 | 6 | 10 | chr10 | 72341114 | rs827341 | C/T |
| chr10 | 72347029 | 2 | 3 | chr10 | 72347029 | rs4747111 | C/G |
| chr10 | 72459033 | 13 | 1 | chr10 | 72459033 | rs7094779 | C/G |
| chr10 | 72463859 | 1 | 5 | chr10 | 72463857 | rs12261506 | C/T |
| chr10 | 72465883 | 3 | 5 | chr10 | 72465882 | rs1891159 | A/G |
| chr10 | 72714960 | 11 | 2 | chr10 | 72714959 | rs6480503 | A/G |
| chr10 | 72717599 | 0 | 0 | chr10 | 72717598 | rs7074280 | A/G |
| chr10 | 72750194 | 3 | 2 | chr10 | 72750192 | rs7090611 | C/T |
| chr10 | 73139448 | 3 | 2 | chr10 | 73139447 | rs7072160 | A/G |
| chr10 | 73197682 | 3 | 2 | chr10 | 73197681 | rs3747865 | C/T |
| chr10 | 73265744 | 1 | 4 | chr10 | 73265743 | rs56041351 | A/G |
| chr10 | 73493110 | 2 | 2 | chr10 | 73493107 | rs1530801 | A/G |
| chr10 | 73738158 | 3 | 2 | chr10 | 73738156 | rs4275534 | C/T |
| chr10 | 73764672 | 6 | 2 | chr10 | 73764670 | rs1879471 | C/T |
| chr10 | 75581348 | 5 | 1 | chr10 | 75581347 | rs10824083 | C/G |
| chr10 | 77240067 | 13 | 1 | chr10 | 77240065 | rs9299529 | C/T |
| chr10 | 77506509 | 2 | 3 | chr10 | 77506507 | rs7087016 | C/T |
| chr10 | 77748166 | 4 | 16 | chr10 | 77748164 | rs967948 | C/T |
| chr10 | 77894410 | 3 | 1 | chr10 | 77894409 | rs10740458 | A/G |
| chr10 | 78337197 | 1 | 10 | chr10 | 78337196 | rs7069982 | A/G |
| chr10 | 78595726 | 1 | 6 | chr10 | 78595724 | rs7900755 | C/T |
| chr10 | 79116908 | 2 | 3 | chr10 | 79116906 | rs11002247 | C/T |
| chr10 | 79316910 | 3 | 1 | chr10 | 79316909 | rs2801826 | A/G |
| chr10 | 79790200 | 2 | 3 | chr10 | 79790199 | rs2559536 | G/T |
| chr10 | 80085592 | 3 | 2 | chr10 | 80085591 | rs1655624 | A/G |
| chr10 | 80297589 | 6 | 1 | chr10 | 80297587 | rs2993816 | A/G |
| chr10 | 80364195 | 1 | 6 | chr10 | 80364194 | rs6480911 | A/G |
| chr10 | 82031731 | 3 | 1 | chr10 | 82031730 | rs756208 | A/G |
| chr10 | 82194241 | 2 | 3 | chr10 | 82194238 | rs55823231 | C/G |

| | | | | | | | |
|---|---|---|---|---|---|---|---|
| chr10 | 82785474 | 2 | 2 | chr10 | 82785473 | rs7068114 | A/G |
| chr10 | 82908523 | 1 | 3 | chr10 | 82908521 | rs7095884 | C/T |
| chr10 | 83430917 | 2 | 2 | chr10 | 83430917 | rs857919 | A/C |
| chr10 | 84854400 | 1 | 6 | chr10 | 84854399 | rs10885758 | A/G |
| chr10 | 87151753 | 2 | 2 | chr10 | 87151752 | rs4991677 | A/G |
| chr10 | 88081830 | 2 | 2 | chr10 | 88081829 | rs7905184 | C/G |
| chr10 | 88286236 | 4 | 9 | chr10 | 88286235 | rs2803583 | A/G |
| chr10 | 88405592 | 3 | 2 | chr10 | 88405590 | rs2675704 | A/C |
| chr10 | 88425106 | 8 | 4 | chr10 | 88425105 | rs12268509 | A/G |
| chr10 | 90580726 | 2 | 5 | chr10 | 90580725 | rs10736363 | A/G |
| chr10 | 92239739 | 5 | 1 | chr10 | 92239738 | rs55662597 | A/G |
| chr10 | 93730410 | 4 | 3 | chr10 | 93730408 | rs2792022 | C/T |
| chr10 | 94819456 | 1 | 4 | chr10 | 94819455 | rs4244352 | A/G |
| chr10 | 97311171 | 6 | 2 | chr10 | 97311171 | rs1078641 | A/G |
| chr10 | 98838925 | 4 | 2 | chr10 | 98838924 | rs2805602 | A/G |
| chr10 | 98960901 | 2 | 4 | chr10 | 98960899 | rs1253426 | G/T |
| chr10 | 99105673 | 1 | 3 | chr10 | 99105672 | rs10882904 | A/G |
| chr10 | 99319426 | 3 | 2 | chr10 | 99319424 | rs7919450 | C/T |
| chr10 | 99531094 | 4 | 11 | chr10 | 99531093 | rs7072751 | A/G |
| chr10 | 99674358 | 3 | 1 | chr10 | 99674355 | rs10883020 | C/T |
| chr10 | 100101698 | 1 | 3 | chr10 | 100101696 | rs4917822 | A/C |
| chr10 | 100101698 | 1 | 3 | chr10 | 100101697 | rs56757394 | A/G |
| chr10 | 100267172 | 3 | 2 | chr10 | 100267170 | rs2169462 | A/G |
| chr10 | 100947920 | 3 | 1 | chr10 | 100947919 | rs4456193 | A/G |
| chr10 | 101333423 | 2 | 7 | chr10 | 101333420 | rs10736145 | C/T |
| chr10 | 101354515 | 6 | 1 | chr10 | 101354513 | rs7075305 | C/T |
| chr10 | 102489259 | 1 | 3 | chr10 | 102489257 | rs4244340 | C/G |
| chr10 | 102529418 | 2 | 3 | chr10 | 102529418 | rs7901210 | C/G |
| chr10 | 102877153 | 3 | 5 | chr10 | 102877152 | rs2788730 | C/T |
| chr10 | 104186457 | 4 | 5 | chr10 | 104186454 | rs2224374 | C/T |
| chr10 | 104367753 | 1 | 5 | chr10 | 104367751 | rs7091457 | C/T |
| chr10 | 104850385 | 1 | 5 | chr10 | 104850384 | rs2863727 | G/T |

| | | | | | | | |
|---|---|---|---|---|---|---|---|
| chr10 | 105544324 | 5 | 3 | chr10 | 105544323 | rs6584570 | C/G |
| chr10 | 105665937 | 1 | 6 | chr10 | 105665935 | rs9419958 | C/T |
| chr10 | 107517091 | 6 | 14 | chr10 | 107517090 | rs4421687 | A/G |
| chr10 | 107885950 | 7 | 4 | chr10 | 107885948 | rs12782910 | A/C |
| chr10 | 108794830 | 6 | 2 | chr10 | 108794829 | rs10509826 | A/G |
| chr10 | 109222383 | 1 | 5 | chr10 | 109222381 | rs11193499 | A/C |
| chr10 | 111495188 | 2 | 3 | chr10 | 111495187 | rs10884906 | A/G |
| chr10 | 111625203 | 3 | 1 | chr10 | 111625201 | rs3818285 | C/T |
| chr10 | 112183464 | 2 | 4 | chr10 | 112183462 | rs10884968 | C/G |
| chr10 | 112425299 | 6 | 8 | chr10 | 112425297 | rs2039876 | C/G |
| chr10 | 112688572 | 3 | 1 | chr10 | 112688571 | rs11195382 | A/G |
| chr10 | 113255753 | 2 | 6 | chr10 | 113255751 | rs1914106 | A/G |
| chr10 | 114399005 | 9 | 3 | chr10 | 114399004 | rs2057577 | A/G |
| chr10 | 114888084 | 5 | 1 | chr10 | 114888082 | rs3814573 | C/T |
| chr10 | 114890489 | 2 | 3 | chr10 | 114890488 | rs7912600 | A/G |
| chr10 | 115287557 | 1 | 6 | chr10 | 115287556 | rs4918837 | A/G |
| chr10 | 115732467 | 1 | 6 | chr10 | 115732466 | rs180909 | A/G |
| chr10 | 117568439 | 5 | 3 | chr10 | 117568438 | rs2960665 | G/T |
| chr10 | 117776746 | 3 | 1 | chr10 | 117776744 | rs59195060 | C/T |
| chr10 | 117776746 | 3 | 1 | chr10 | 117776745 | rs180620 | C/T |
| chr10 | 117779524 | 2 | 14 | chr10 | 117779522 | rs180612 | A/C |
| chr10 | 118480758 | 6 | 5 | chr10 | 118480757 | rs1004155 | C/T |
| chr10 | 118535255 | 2 | 3 | chr10 | 118535254 | rs3095119 | C/T |
| chr10 | 119013180 | 2 | 2 | chr10 | 119013179 | rs2803813 | C/T |
| chr10 | 119326010 | 8 | 1 | chr10 | 119326009 | rs728558 | C/T |
| chr10 | 119421593 | 3 | 1 | chr10 | 119421592 | rs6585448 | A/G |
| chr10 | 120319160 | 3 | 3 | chr10 | 120319159 | rs1711884 | C/T |
| chr10 | 120817070 | 4 | 1 | chr10 | 120817068 | rs10749284 | C/T |
| chr10 | 120832460 | 2 | 3 | chr10 | 120832458 | rs7083985 | C/T |
| chr10 | 121178326 | 1 | 11 | chr10 | 121178324 | rs933049 | A/G |
| chr10 | 121723984 | 3 | 5 | chr10 | 121723982 | rs10788008 | C/T |
| chr10 | 121872459 | 2 | 2 | chr10 | 121872457 | rs10736282 | C/T |

| | | | | | | | | |
|---|---|---|---|---|---|---|---|---|
| chr10 | 122393696 | 1 | 3 | chr10 | 122393695 | rs12781455 | | A/G |
| chr10 | 122399802 | 2 | 5 | chr10 | 122399801 | rs7904483 | | A/G |
| chr10 | 123162229 | 2 | 2 | chr10 | 123162227 | rs11199951 | | C/T |
| chr10 | 123275425 | 7 | 5 | chr10 | 123275424 | rs2912768 | | A/G |
| chr10 | 123330424 | 0 | 0 | chr10 | 123330422 | rs35054928 | | -/C |
| chr10 | 123393934 | 2 | 4 | chr10 | 123393933 | rs7894302 | | A/G |
| chr10 | 123463785 | 3 | 1 | chr10 | 123463784 | rs2935699 | | C/T |
| chr10 | 123471190 | 1 | 3 | chr10 | 123471189 | rs2935695 | | C/T |
| chr10 | 123762999 | 3 | 2 | chr10 | 123762997 | rs3750841 | | C/T |
| chr10 | 123835313 | 3 | 7 | chr10 | 123835311 | rs7073433 | | C/T |
| chr10 | 124060143 | 3 | 1 | chr10 | 124060142 | rs10788277 | | A/G |
| chr10 | 124169087 | 4 | 1 | chr10 | 124169085 | rs41307060 | | C/T |
| chr10 | 124234960 | 4 | 1 | chr10 | 124234958 | rs2268344 | | C/T |
| chr10 | 124311793 | 5 | 3 | chr10 | 124311791 | rs2981750 | | C/T |
| chr10 | 124823810 | 4 | 1 | chr10 | 124823808 | rs7087887 | | A/C |
| chr10 | 124887511 | 6 | 2 | chr10 | 124887508 | rs10794597 | | C/T |
| chr10 | 125322823 | 4 | 2 | chr10 | 125322820 | rs1710960 | | A/G |
| chr10 | 125322823 | 4 | 2 | chr10 | 125322821 | rs7072179 | | A/C |
| chr10 | 125364545 | 5 | 2 | chr10 | 125364542 | rs2185134 | | C/T |
| chr10 | 125419019 | 1 | 3 | chr10 | 125419018 | rs1914000 | | C/T |
| chr10 | 125588315 | 6 | 1 | chr10 | 125588314 | rs7895642 | | A/G |
| chr10 | 125755964 | 2 | 7 | chr10 | 125755962 | rs4590806 | | A/G |
| chr10 | 125962999 | 8 | 5 | chr10 | 125962998 | rs7900964 | | A/G |
| chr10 | 125969721 | 1 | 3 | chr10 | 125969720 | rs7908113 | | A/G |
| chr10 | 126222937 | 2 | 3 | chr10 | 126222936 | rs7093795 | | A/G |
| chr10 | 126231962 | 4 | 3 | chr10 | 126231961 | rs2085174 | | C/T |
| chr10 | 126284506 | 1 | 10 | chr10 | 126284504 | rs10901784 | | C/T |
| chr10 | 126323690 | 1 | 4 | chr10 | 126323688 | rs7100830 | | C/T |
| chr10 | 126813705 | 6 | 2 | chr10 | 126813703 | rs1693624 | | C/T |
| chr10 | 127468054 | 1 | 3 | chr10 | 127468053 | rs35711389 | | C/G |
| chr10 | 127564966 | 7 | 22 | chr10 | 127564964 | rs7477787 | | C/T |
| chr10 | 127572716 | 10 | 33 | chr10 | 127572714 | rs3863578 | | A/G |

| | | | | | | | |
|---|---|---|---|---|---|---|---|
| chr10 | 127572716 | 10 | 33 | chr10 | 127572715 | rs4989984 | C/G |
| chr10 | 127574050 | 5 | 5 | chr10 | 127574048 | rs5010795 | C/T |
| chr10 | 127574078 | 1 | 40 | chr10 | 127574076 | rs5010792 | C/T |
| chr10 | 127574078 | 1 | 40 | chr10 | 127574078 | rs66743445 | -/G |
| chr10 | 127631265 | 2 | 4 | chr10 | 127631264 | rs10794041 | G/T |
| chr10 | 128170102 | 3 | 1 | chr10 | 128170100 | rs4497314 | C/G |
| chr10 | 128466059 | 1 | 4 | chr10 | 128466058 | rs11599718 | A/G |
| chr10 | 128525193 | 2 | 5 | chr10 | 128525190 | rs7913621 | C/T |
| chr10 | 128583658 | 3 | 11 | chr10 | 128583657 | rs12781269 | C/G |
| chr10 | 128819367 | 3 | 6 | chr10 | 128819365 | rs2251922 | A/C |
| chr10 | 128830296 | 7 | 1 | chr10 | 128830295 | rs2483853 | C/T |
| chr10 | 128831967 | 4 | 5 | chr10 | 128831965 | rs951078 | A/G |
| chr10 | 128884377 | 2 | 2 | chr10 | 128884377 | rs2489380 | C/G |
| chr10 | 129146705 | 7 | 1 | chr10 | 129146704 | rs7087658 | A/G |
| chr10 | 129162899 | 1 | 7 | chr10 | 129162897 | rs11018133 | C/T |
| chr10 | 129256664 | 3 | 2 | chr10 | 129256663 | rs1547323 | A/G |
| chr10 | 129380294 | 3 | 2 | chr10 | 129380292 | rs10765151 | C/T |
| chr10 | 129515441 | 2 | 5 | chr10 | 129515439 | rs487253 | A/G |
| chr10 | 129740536 | 5 | 5 | chr10 | 129740535 | rs10829320 | A/G |
| chr10 | 129749056 | 3 | 1 | chr10 | 129749054 | rs11016046 | C/T |
| chr10 | 129766641 | 4 | 6 | chr10 | 129766639 | rs7080285 | C/T |
| chr10 | 130267686 | 1 | 3 | chr10 | 130267684 | rs61863636 | C/G |
| chr10 | 130379643 | 4 | 1 | chr10 | 130379641 | rs7916502 | C/T |
| chr10 | 130502430 | 2 | 4 | chr10 | 130502427 | rs12252560 | C/T |
| chr10 | 130527553 | 3 | 1 | chr10 | 130527551 | rs35481173 | C/T |
| chr10 | 130825576 | 14 | 4 | chr10 | 130825575 | rs4750741 | A/G |
| chr10 | 130913099 | 3 | 3 | chr10 | 130913097 | rs543469 | A/G |
| chr10 | 130962239 | 1 | 3 | chr10 | 130962238 | rs2542626 | C/T |
| chr10 | 131010214 | 2 | 3 | chr10 | 131010211 | rs3095757 | G/T |
| chr10 | 131113442 | 0 | 0 | chr10 | 131113441 | rs10764879 | A/G |
| chr10 | 131166411 | 6 | 2 | chr10 | 131166410 | rs1711661 | C/T |
| chr10 | 131339477 | 1 | 5 | chr10 | 131339476 | rs2026976 | C/G |

| | | | | | | | |
|---|---|---|---|---|---|---|---|
| chr10 | 131392302 | 2 | 3 | chr10 | 131392301 | rs1978756 | A/G |
| chr10 | 131880835 | 5 | 4 | chr10 | 131880833 | rs10829706 | C/T |
| chr10 | 131905278 | 0 | 0 | chr10 | 131905276 | rs9943466 | C/T |
| chr10 | 131950554 | 3 | 4 | chr10 | 131950552 | rs7898442 | C/T |
| chr10 | 131950554 | 3 | 4 | chr10 | 131950553 | rs6482783 | A/G |
| chr10 | 131961763 | 1 | 5 | chr10 | 131961761 | rs318929 | C/T |
| chr10 | 132432178 | 1 | 7 | chr10 | 132432177 | rs7905791 | G/T |
| chr10 | 132494819 | 3 | 1 | chr10 | 132494817 | rs7896546 | C/T |
| chr10 | 132509390 | 1 | 4 | chr10 | 132509389 | rs10829807 | A/G |
| chr10 | 132813950 | 6 | 1 | chr10 | 132813949 | rs10829908 | A/G |
| chr10 | 132843122 | 1 | 4 | chr10 | 132843121 | rs10829933 | A/G |
| chr10 | 132916184 | 9 | 4 | chr10 | 132916183 | rs61861222 | G/T |
| chr10 | 133526246 | 1 | 4 | chr10 | 133526244 | rs10747291 | A/C |
| chr10 | 133796327 | 2 | 4 | chr10 | 133796326 | rs2818392 | C/T |
| chr10 | 134068286 | 7 | 4 | chr10 | 134068285 | rs10747057 | C/G |
| chr10 | 134119303 | 2 | 4 | chr10 | 134119302 | rs11146379 | A/G |
| chr10 | 134166612 | 1 | 9 | chr10 | 134166611 | rs10870315 | A/G |
| chr10 | 134185147 | 3 | 3 | chr10 | 134185146 | rs10747068 | A/G |
| chr10 | 134399682 | 8 | 1 | chr10 | 134399681 | rs12259699 | A/G |
| chr10 | 134469429 | 4 | 2 | chr10 | 134469429 | rs2804020 | G/T |
| chr10 | 134534231 | 1 | 4 | chr10 | 134534229 | rs7100926 | C/T |
| chr10 | 134574572 | 2 | 7 | chr10 | 134574571 | rs13376869 | A/G |
| chr10 | 134651069 | 2 | 2 | chr10 | 134651067 | rs10870219 | C/T |
| chr10 | 134673806 | 1 | 8 | chr10 | 134673803 | rs10870230 | C/G |
| chr10 | 134752968 | 4 | 6 | chr10 | 134752968 | rs2998113 | C/T |
| chr10 | 134821593 | 4 | 4 | chr10 | 134821591 | rs4326710 | C/T |
| chr10 | 134862153 | 5 | 5 | chr10 | 134862151 | rs2998139 | A/G |
| chr10 | 134864419 | 4 | 1 | chr10 | 134864418 | rs2998135 | C/T |
| chr10 | 134873443 | 5 | 1 | chr10 | 134873440 | rs10857686 | C/G |
| chr10 | 134873591 | 4 | 7 | chr10 | 134873589 | rs9419027 | C/T |
| chr10 | 135014559 | 8 | 3 | chr10 | 135014558 | rs28392834 | A/G |
| chr10 | 135052585 | 3 | 2 | chr10 | 135052583 | rs10776679 | C/T |

| | | | | | | | |
|---|---|---|---|---|---|---|---|
| chr10 | 135117729 | 19 | 1 | chr10 | 135117727 | rs2253529 | A/G |
| chr10 | 135319833 | 1 | 3 | chr10 | 135319832 | rs3020476 | A/G |
| chr10 | 135350313 | 4 | 2 | chr10 | 135350311 | rs71227432 | A/G |
| chr10 | 135350313 | 4 | 2 | chr10 | 135350312 | rs71209551 | G/T |
| chr10 | 135350313 | 4 | 2 | chr10 | 135350312 | rs35619444 | -/G |
| chr10 | 135350313 | 4 | 2 | chr10 | 135350313 | rs35336654 | C/G |
| chr11 | 179644 | 5 | 6 | chr11 | 179641 | rs2948211 | C/T |
| chr11 | 179644 | 5 | 6 | chr11 | 179641 | rs61878361 | C/T |
| chr11 | 234552 | 2 | 4 | chr11 | 234551 | rs2272566 | A/G |
| chr11 | 247309 | 1 | 3 | chr11 | 247307 | rs939924 | C/T |
| chr11 | 281694 | 3 | 6 | chr11 | 281693 | rs11246055 | A/G |
| chr11 | 361515 | 3 | 1 | chr11 | 361513 | rs72847390 | C/T |
| chr11 | 383212 | 2 | 2 | chr11 | 383211 | rs10902155 | A/G |
| chr11 | 396474 | 1 | 3 | chr11 | 396472 | rs3087588 | A/C |
| chr11 | 426462 | 2 | 3 | chr11 | 426460 | rs11246159 | C/T |
| chr11 | 428662 | 3 | 3 | chr11 | 428661 | rs7481614 | C/T |
| chr11 | 826024 | 0 | 0 | chr11 | 826022 | rs28475448 | C/T |
| chr11 | 845116 | 5 | 6 | chr11 | 845115 | rs28666948 | A/G |
| chr11 | 845935 | 1 | 4 | chr11 | 845934 | rs28457608 | A/G |
| chr11 | 857283 | 2 | 2 | chr11 | 857281 | rs28699999 | C/T |
| chr11 | 875686 | 3 | 3 | chr11 | 875684 | rs7938542 | A/G |
| chr11 | 890928 | 2 | 2 | chr11 | 890928 | rs10794339 | C/G |
| chr11 | 961167 | 3 | 5 | chr11 | 961165 | rs7103585 | A/G |
| chr11 | 983745 | 2 | 2 | chr11 | 983744 | rs7104956 | C/G |
| chr11 | 1071277 | 12 | 3 | chr11 | 1071276 | rs41352846 | A/G |
| chr11 | 1114522 | 4 | 4 | chr11 | 1114519 | rs55722966 | C/T |
| chr11 | 1114522 | 4 | 4 | chr11 | 1114521 | rs6597964 | C/T |
| chr11 | 1136677 | 1 | 3 | chr11 | 1136677 | rs28624253 | A/G |
| chr11 | 1170036 | 1 | 10 | chr11 | 1170036 | rs61867534 | G/T |
| chr11 | 1174704 | 0 | 0 | chr11 | 1174703 | rs28503875 | A/G |
| chr11 | 1175727 | 4 | 7 | chr11 | 1175726 | rs35915689 | C/G |
| chr11 | 1175727 | 4 | 7 | chr11 | 1175727 | rs34474233 | A/G |

| | | | | | | | |
|---|---|---|---|---|---|---|---|
| chr11 | 1176373 | 12 | 2 | chr11 | 1176372 | rs56142040 | G/T |
| chr11 | 1181655 | 1 | 3 | chr11 | 1181653 | rs28483241 | C/T |
| chr11 | 1198425 | 0 | 0 | chr11 | 1198423 | rs2735726 | C/T |
| chr11 | 1399132 | 6 | 2 | chr11 | 1399129 | rs11026429 | A/C |
| chr11 | 1399132 | 6 | 2 | chr11 | 1399130 | rs10766917 | C/T |
| chr11 | 1441643 | 2 | 2 | chr11 | 1441642 | rs1554857 | C/T |
| chr11 | 1460204 | 3 | 1 | chr11 | 1460202 | rs2878234 | A/G |
| chr11 | 1465008 | 2 | 2 | chr11 | 1465007 | rs2334556 | C/T |
| chr11 | 1506262 | 12 | 1 | chr11 | 1506260 | rs6578484 | C/T |
| chr11 | 1532812 | 3 | 6 | chr11 | 1532810 | rs6578504 | C/T |
| chr11 | 1594121 | 1 | 8 | chr11 | 1594119 | rs10837692 | C/T |
| chr11 | 1619346 | 2 | 9 | chr11 | 1619345 | rs11037751 | A/G |
| chr11 | 1630882 | 2 | 2 | chr11 | 1630881 | rs28688280 | A/G |
| chr11 | 1731243 | 2 | 3 | chr11 | 1731241 | rs12214 | A/G |
| chr11 | 1821140 | 1 | 4 | chr11 | 1821139 | rs628988 | C/T |
| chr11 | 1821169 | 2 | 4 | chr11 | 1821167 | rs504297 | C/G |
| chr11 | 2017315 | 5 | 1 | chr11 | 2017314 | rs7129946 | A/G |
| chr11 | 2094715 | 2 | 2 | chr11 | 2094714 | rs6578985 | A/G |
| chr11 | 2106202 | 4 | 4 | chr11 | 2106200 | rs7107701 | C/T |
| chr11 | 2126485 | 5 | 2 | chr11 | 2126483 | rs3741204 | A/G |
| chr11 | 2143974 | 3 | 4 | chr11 | 2143973 | rs4072823 | G/T |
| chr11 | 2164819 | 1 | 5 | chr11 | 2164816 | rs61871299 | A/C |
| chr11 | 2164819 | 1 | 5 | chr11 | 2164816 | rs71472145 | AA/CC |
| chr11 | 2164819 | 1 | 5 | chr11 | 2164817 | rs61871300 | A/C |
| chr11 | 2173622 | 2 | 4 | chr11 | 2173621 | rs10770163 | A/G |
| chr11 | 2180021 | 2 | 3 | chr11 | 2180019 | rs7480469 | C/T |
| chr11 | 2190373 | 3 | 1 | chr11 | 2190371 | rs12363029 | C/T |
| chr11 | 2190373 | 3 | 1 | chr11 | 2190372 | rs10840603 | A/G |
| chr11 | 2508956 | 3 | 2 | chr11 | 2508954 | rs2741963 | C/T |
| chr11 | 2692759 | 2 | 3 | chr11 | 2692758 | rs231847 | C/T |
| chr11 | 2780410 | 1 | 5 | chr11 | 2780409 | rs163169 | C/T |
| chr11 | 2783064 | 2 | 3 | chr11 | 2783062 | rs163164 | C/T |

| | | | | | | | |
|---|---|---|---|---|---|---|---|
| chr11 | 2831739 | 7 | 2 | chr11 | 2831738 | rs7108037 | A/G |
| chr11 | 2876762 | 5 | 2 | chr11 | 2876761 | rs449318 | A/G |
| chr11 | 2890675 | 2 | 4 | chr11 | 2890674 | rs408531 | A/G |
| chr11 | 3054433 | 2 | 4 | chr11 | 3054432 | rs4758467 | A/G |
| chr11 | 3099607 | 3 | 1 | chr11 | 3099605 | rs6578322 | C/T |
| chr11 | 3132145 | 3 | 2 | chr11 | 3132143 | rs4536189 | C/T |
| chr11 | 3173344 | 4 | 1 | chr11 | 3173342 | rs4373897 | C/T |
| chr11 | 3176138 | 7 | 7 | chr11 | 3176137 | rs3898775 | A/G |
| chr11 | 3176183 | 3 | 1 | chr11 | 3176181 | rs1318727 | C/T |
| chr11 | 3333911 | 4 | 3 | chr11 | 3333910 | rs7395099 | A/G |
| chr11 | 3489694 | 2 | 3 | chr11 | 3489692 | rs4980410 | C/T |
| chr11 | 3615493 | 2 | 6 | chr11 | 3615492 | rs6578406 | A/G |
| chr11 | 5162871 | 5 | 4 | chr11 | 5162869 | rs7934373 | C/T |
| chr11 | 5398706 | 4 | 3 | chr11 | 5398704 | rs2647575 | G/T |
| chr11 | 5489977 | 2 | 2 | chr11 | 5489977 | rs317780 | G/T |
| chr11 | 6297282 | 4 | 5 | chr11 | 6297281 | rs1051992 | C/T |
| chr11 | 6822335 | 4 | 2 | chr11 | 6822333 | rs2595437 | C/T |
| chr11 | 7629683 | 1 | 6 | chr11 | 7629681 | rs11041499 | C/T |
| chr11 | 8321332 | 3 | 6 | chr11 | 8321330 | rs11041888 | C/T |
| chr11 | 9766656 | 5 | 1 | chr11 | 9766654 | rs360130 | C/T |
| chr11 | 10366317 | 3 | 10 | chr11 | 10366315 | rs4243913 | -/A/C |
| chr11 | 10479422 | 7 | 4 | chr11 | 10479421 | rs4910147 | A/G |
| chr11 | 10623851 | 1 | 3 | chr11 | 10623850 | rs6484437 | A/G |
| chr11 | 10625805 | 4 | 7 | chr11 | 10625803 | rs7940646 | C/T |
| chr11 | 11030803 | 1 | 10 | chr11 | 11030801 | rs1586383 | A/G |
| chr11 | 11280484 | 1 | 3 | chr11 | 11280482 | rs4300388 | C/T |
| chr11 | 11375405 | 3 | 4 | chr11 | 11375404 | rs1487119 | A/G |
| chr11 | 11771103 | 1 | 4 | chr11 | 11771101 | rs1455250 | A/C |
| chr11 | 12218858 | 2 | 4 | chr11 | 12218856 | rs2706630 | A/G |
| chr11 | 12773064 | 3 | 2 | chr11 | 12773063 | rs3927025 | G/T |
| chr11 | 12910081 | 1 | 3 | chr11 | 12910080 | rs4757066 | A/G |
| chr11 | 14212184 | 2 | 2 | chr11 | 14212183 | rs11023156 | A/G |

| | | | | | | | |
|---|---|---|---|---|---|---|---|
| chr11 | 14218951 | 4 | 2 | chr11 | 14218950 | rs7116756 | A/G |
| chr11 | 14769559 | 4 | 3 | chr11 | 14769558 | rs2060794 | C/G |
| chr11 | 15379707 | 1 | 6 | chr11 | 15379705 | rs11023563 | C/T |
| chr11 | 15474182 | 7 | 1 | chr11 | 15474180 | rs7935111 | C/T |
| chr11 | 15527791 | 3 | 3 | chr11 | 15527790 | rs10766267 | A/G |
| chr11 | 15544525 | 1 | 3 | chr11 | 15544523 | rs7928688 | C/T |
| chr11 | 16918165 | 6 | 2 | chr11 | 16918163 | rs2353367 | C/T |
| chr11 | 17584191 | 1 | 3 | chr11 | 17584188 | rs7934079 | C/G |
| chr11 | 18661439 | 8 | 5 | chr11 | 18661437 | rs1446658 | C/T |
| chr11 | 19018500 | 4 | 3 | chr11 | 19018499 | rs56959156 | A/G |
| chr11 | 19203227 | 3 | 2 | chr11 | 19203226 | rs793275 | A/C |
| chr11 | 19295752 | 5 | 3 | chr11 | 19295751 | rs4757788 | A/G |
| chr11 | 19609909 | 3 | 5 | chr11 | 19609907 | rs1872804 | C/T |
| chr11 | 19966021 | 8 | 4 | chr11 | 19966020 | rs17614100 | A/G |
| chr11 | 20058084 | 4 | 1 | chr11 | 20058083 | rs4237748 | A/G |
| chr11 | 20090750 | 6 | 1 | chr11 | 20090748 | rs7127722 | C/T |
| chr11 | 22369610 | 5 | 1 | chr11 | 22369608 | rs2593649 | C/T |
| chr11 | 24756634 | 1 | 3 | chr11 | 24756633 | rs1486702 | C/T |
| chr11 | 28672059 | 12 | 2 | chr11 | 28672057 | rs4923560 | C/T |
| chr11 | 30809726 | 2 | 3 | chr11 | 30809722 | rs71481499 | AGG/GGC |
| chr11 | 30809726 | 2 | 3 | chr11 | 30809724 | rs4922561 | C/T |
| chr11 | 31406217 | 2 | 3 | chr11 | 31406216 | rs1223075 | A/G |
| chr11 | 31955549 | 2 | 3 | chr11 | 31955547 | rs2473645 | A/G |
| chr11 | 32024752 | 2 | 2 | chr11 | 32024751 | rs2658577 | A/G |
| chr11 | 32913196 | 4 | 9 | chr11 | 32913195 | rs7481878 | A/G |
| chr11 | 33165195 | 1 | 6 | chr11 | 33165194 | rs34143165 | A/G |
| chr11 | 33348231 | 1 | 5 | chr11 | 33348229 | rs10734418 | C/T |
| chr11 | 33511261 | 3 | 1 | chr11 | 33511260 | rs2615930 | C/T |
| chr11 | 34187093 | 4 | 1 | chr11 | 34187092 | rs289985 | A/G |
| chr11 | 34293915 | 3 | 6 | chr11 | 34293914 | rs4756132 | A/G |
| chr11 | 34461235 | 3 | 1 | chr11 | 34461234 | rs3781708 | A/G |
| chr11 | 35797736 | 8 | 2 | chr11 | 35797735 | rs17735796 | A/G |

| | | | | | | | | |
|---|---|---|---|---|---|---|---|---|
| chr11 | 36092646 | 2 | 9 | chr11 | 36092644 | rs4756282 | C/T |
| chr11 | 36098815 | 3 | 3 | chr11 | 36098814 | rs10742362 | A/G |
| chr11 | 36174460 | 1 | 13 | chr11 | 36174459 | rs2204508 | C/G |
| chr11 | 36443645 | 3 | 1 | chr11 | 36443644 | rs331434 | A/G |
| chr11 | 36678852 | 5 | 2 | chr11 | 36678851 | rs332431 | A/C |
| chr11 | 36838861 | 4 | 4 | chr11 | 36838860 | rs10734445 | A/G |
| chr11 | 36909168 | 2 | 4 | chr11 | 36909167 | rs10836640 | A/G |
| chr11 | 38802422 | 2 | 3 | chr11 | 38802421 | rs1913048 | C/T |
| chr11 | 40058318 | 19 | 3 | chr11 | 40058316 | rs11035677 | C/T |
| chr11 | 40576071 | 5 | 1 | chr11 | 40576070 | rs10837440 | A/G |
| chr11 | 41373507 | 3 | 2 | chr11 | 41373505 | rs1857932 | C/T |
| chr11 | 41929127 | 3 | 1 | chr11 | 41929125 | rs2713820 | A/G |
| chr11 | 42076918 | 18 | 2 | chr11 | 42076917 | rs2200725 | C/T |
| chr11 | 42346519 | 2 | 2 | chr11 | 42346518 | rs11036860 | A/G |
| chr11 | 43494608 | 3 | 5 | chr11 | 43494606 | rs10838115 | C/T |
| chr11 | 43732309 | 16 | 1 | chr11 | 43732309 | rs4643069 | C/T |
| chr11 | 43772176 | 1 | 3 | chr11 | 43772175 | rs10838170 | A/G |
| chr11 | 43959394 | 2 | 2 | chr11 | 43959392 | rs756954 | C/T |
| chr11 | 43960273 | 4 | 6 | chr11 | 43960271 | rs7125768 | C/T |
| chr11 | 44242434 | 3 | 13 | chr11 | 44242432 | rs4755796 | C/T |
| chr11 | 44344652 | 2 | 4 | chr11 | 44344651 | rs7928154 | A/G |
| chr11 | 44354609 | 6 | 1 | chr11 | 44354606 | rs922017 | A/G |
| chr11 | 44476134 | 2 | 3 | chr11 | 44476133 | rs17611951 | A/G |
| chr11 | 44690081 | 5 | 3 | chr11 | 44690080 | rs7120723 | A/G |
| chr11 | 45278454 | 3 | 3 | chr11 | 45278452 | rs7130748 | C/T |
| chr11 | 45356858 | 11 | 2 | chr11 | 45356858 | rs717653 | G/T |
| chr11 | 45497309 | 7 | 2 | chr11 | 45497307 | rs923374 | C/T |
| chr11 | 46108067 | 7 | 6 | chr11 | 46108065 | rs10769190 | C/T |
| chr11 | 47327534 | 2 | 4 | chr11 | 47327532 | rs2071305 | G/T |
| chr11 | 47847648 | 3 | 3 | chr11 | 47847646 | rs7945302 | C/G |
| chr11 | 48156474 | 3 | 10 | chr11 | 48156472 | rs2047815 | A/G |
| chr11 | 49974462 | 1 | 3 | chr11 | 49974460 | rs11493756 | C/T |

| | | | | | | | |
|---|---|---|---|---|---|---|---|
| chr11 | 50174763 | 4 | 4 | chr11 | 50174760 | rs4881692 | A/G |
| chr11 | 50409524 | 5 | 4 | chr11 | 50409522 | rs4963111 | C/G |
| chr11 | 55382007 | 5 | 1 | chr11 | 55382005 | rs1848558 | C/T |
| chr11 | 56626879 | 2 | 2 | chr11 | 56626877 | rs518808 | A/G |
| chr11 | 56626879 | 2 | 2 | chr11 | 56626878 | rs650364 | A/CA/G/TG |
| chr11 | 57861631 | 6 | 2 | chr11 | 57861629 | rs10896754 | C/T |
| chr11 | 58085528 | 4 | 6 | chr11 | 58085527 | rs2515348 | A/G |
| chr11 | 58150498 | 6 | 2 | chr11 | 58150497 | rs663721 | A/G |
| chr11 | 58193225 | 2 | 2 | chr11 | 58193223 | rs4427587 | C/T |
| chr11 | 59717721 | 2 | 4 | chr11 | 59717719 | rs684977 | C/G |
| chr11 | 59872135 | 4 | 2 | chr11 | 59872134 | rs6591572 | A/G |
| chr11 | 60322891 | 4 | 3 | chr11 | 60322889 | rs550602 | A/G |
| chr11 | 60348853 | 6 | 4 | chr11 | 60348851 | rs595018 | A/G |
| chr11 | 60535562 | 6 | 7 | chr11 | 60535561 | rs3016163 | C/T |
| chr11 | 60633787 | 1 | 4 | chr11 | 60633786 | rs572350 | C/T |
| chr11 | 61559216 | 2 | 3 | chr11 | 61559214 | rs4963283 | A/C/T |
| chr11 | 61636721 | 3 | 4 | chr11 | 61636719 | rs11230908 | A/C |
| chr11 | 62207740 | 5 | 3 | chr11 | 62207738 | rs2958240 | C/G |
| chr11 | 62320587 | 1 | 11 | chr11 | 62320584 | rs4693 | A/G |
| chr11 | 62942026 | 1 | 10 | chr11 | 62942024 | rs4963416 | C/T |
| chr11 | 63436873 | 2 | 2 | chr11 | 63436872 | rs320110 | A/G |
| chr11 | 63677265 | 3 | 7 | chr11 | 63677264 | rs2325785 | A/G |
| chr11 | 63779035 | 3 | 7 | chr11 | 63779034 | rs632439 | C/T |
| chr11 | 63868505 | 3 | 3 | chr11 | 63868503 | rs685870 | C/T |
| chr11 | 64025956 | 6 | 3 | chr11 | 64025954 | rs4244816 | C/T |
| chr11 | 64122520 | 2 | 13 | chr11 | 64122518 | rs1529909 | -/A/G |
| chr11 | 64161540 | 2 | 2 | chr11 | 64161538 | rs2849041 | A/G |
| chr11 | 64206875 | 5 | 1 | chr11 | 64206873 | rs1152630 | C/T |
| chr11 | 64361847 | 3 | 3 | chr11 | 64361845 | rs58790228 | C/T |
| chr11 | 64795969 | 5 | 5 | chr11 | 64795968 | rs677740 | A/G |
| chr11 | 65306184 | 5 | 3 | chr11 | 65306181 | rs12801731 | C/T |
| chr11 | 65388457 | 0 | 0 | chr11 | 65388456 | rs630755 | C/T |

| | | | | | | | |
|---|---|---|---|---|---|---|---|
| chr11 | 65406350 | 2 | 8 | chr11 | 65406349 | rs604630 | A/G |
| chr11 | 65570946 | 3 | 2 | chr11 | 65570944 | rs9667922 | C/T |
| chr11 | 65788027 | 11 | 4 | chr11 | 65788025 | rs525698 | C/T |
| chr11 | 66242720 | 4 | 3 | chr11 | 66242718 | rs551708 | C/T |
| chr11 | 67156812 | 5 | 1 | chr11 | 67156810 | rs10896189 | C/T |
| chr11 | 67194772 | 6 | 3 | chr11 | 67194770 | rs4313593 | C/T |
| chr11 | 67864403 | 4 | 5 | chr11 | 67864402 | rs678765 | A/G |
| chr11 | 68573402 | 2 | 14 | chr11 | 68573400 | rs4453241 | C/T |
| chr11 | 68624413 | 2 | 2 | chr11 | 68624410 | rs72930631 | C/T |
| chr11 | 68637994 | 1 | 3 | chr11 | 68637992 | rs3019778 | C/T |
| chr11 | 68638398 | 0 | 0 | chr11 | 68638396 | rs3019780 | C/T |
| chr11 | 68679408 | 4 | 4 | chr11 | 68679406 | rs2924524 | A/G |
| chr11 | 68703433 | 5 | 2 | chr11 | 68703433 | rs12417953 | A/G |
| chr11 | 68879570 | 1 | 4 | chr11 | 68879569 | rs12280075 | G/T |
| chr11 | 68906172 | 5 | 2 | chr11 | 68906172 | rs4980737 | A/G |
| chr11 | 69098036 | 2 | 7 | chr11 | 69098033 | rs602124 | C/G |
| chr11 | 69298488 | 13 | 5 | chr11 | 69298488 | rs9666457 | A/G |
| chr11 | 69336943 | 3 | 4 | chr11 | 69336942 | rs10908229 | A/G |
| chr11 | 69507366 | 3 | 1 | chr11 | 69507364 | rs72931768 | C/G |
| chr11 | 69527847 | 5 | 2 | chr11 | 69527846 | rs10899055 | A/G |
| chr11 | 69641401 | 1 | 4 | chr11 | 69641398 | rs2509145 | A/G |
| chr11 | 70074734 | 2 | 2 | chr11 | 70074733 | rs11236606 | A/G |
| chr11 | 70127863 | 5 | 1 | chr11 | 70127862 | rs7938947 | G/T |
| chr11 | 70350047 | 0 | 0 | chr11 | 70350046 | rs3019835 | C/T |
| chr11 | 70357743 | 1 | 3 | chr11 | 70357742 | rs7932689 | A/G |
| chr11 | 70363825 | 4 | 1 | chr11 | 70363824 | rs2921341 | A/G |
| chr11 | 70518446 | 3 | 4 | chr11 | 70518444 | rs11232163 | C/T |
| chr11 | 70541362 | 4 | 1 | chr11 | 70541361 | rs11232297 | A/G |
| chr11 | 70671901 | 1 | 8 | chr11 | 70671900 | rs11820718 | A/G |
| chr11 | 70850824 | 1 | 3 | chr11 | 70850822 | rs1792319 | C/G |
| chr11 | 71385138 | 2 | 2 | chr11 | 71385136 | rs3829215 | A/G |
| chr11 | 71950236 | 1 | 3 | chr11 | 71950234 | rs1110011 | C/T |

| | | | | | | | |
|---|---|---|---|---|---|---|---|
| chr11 | 71963805 | 2 | 2 | chr11 | 71963803 | rs402479 | C/T |
| chr11 | 72073183 | 7 | 2 | chr11 | 72073182 | rs1872126 | C/T |
| chr11 | 72080442 | 11 | 1 | chr11 | 72080441 | rs7944113 | A/G |
| chr11 | 72139893 | 7 | 3 | chr11 | 72139892 | rs1785213 | C/T |
| chr11 | 72215790 | 1 | 26 | chr11 | 72215788 | rs3862793 | C/T |
| chr11 | 73036312 | 3 | 2 | chr11 | 73036310 | rs667350 | C/G |
| chr11 | 73951606 | 2 | 5 | chr11 | 73951605 | rs7941941 | A/G |
| chr11 | 75108598 | 3 | 2 | chr11 | 75108597 | rs650861 | A/G |
| chr11 | 75594983 | 3 | 2 | chr11 | 75594981 | rs1879633 | C/T |
| chr11 | 76036410 | 2 | 5 | chr11 | 76036408 | rs11602550 | C/T |
| chr11 | 76047696 | 2 | 4 | chr11 | 76047694 | rs1320646 | A/G |
| chr11 | 76104752 | 10 | 7 | chr11 | 76104750 | rs7949035 | C/T |
| chr11 | 76124012 | 11 | 1 | chr11 | 76124011 | rs7951607 | A/G |
| chr11 | 76476653 | 3 | 2 | chr11 | 76476651 | rs7121748 | C/T |
| chr11 | 78256831 | 2 | 3 | chr11 | 78256829 | rs2725808 | C/T |
| chr11 | 79187898 | 4 | 2 | chr11 | 79187897 | rs12805269 | A/G |
| chr11 | 79518550 | 1 | 7 | chr11 | 79518548 | rs4281499 | C/T |
| chr11 | 79574768 | 12 | 1 | chr11 | 79574768 | rs1009015 | C/T |
| chr11 | 79882613 | 1 | 7 | chr11 | 79882612 | rs4430541 | A/G |
| chr11 | 82998970 | 1 | 3 | chr11 | 82998969 | rs497917 | A/G |
| chr11 | 83018964 | 1 | 4 | chr11 | 83018963 | rs2060147 | A/G |
| chr11 | 83231620 | 1 | 3 | chr11 | 83231619 | rs10898157 | A/G |
| chr11 | 84243833 | 3 | 1 | chr11 | 84243831 | rs10501574 | C/T |
| chr11 | 84798047 | 11 | 6 | chr11 | 84798046 | rs286040 | A/G |
| chr11 | 85199477 | 3 | 11 | chr11 | 85199476 | rs2044391 | C/G |
| chr11 | 85217265 | 5 | 1 | chr11 | 85217264 | rs11234429 | A/G |
| chr11 | 86132395 | 6 | 1 | chr11 | 86132393 | rs2433438 | C/T |
| chr11 | 88079083 | 1 | 9 | chr11 | 88079082 | rs6483387 | A/G |
| chr11 | 88994235 | 7 | 2 | chr11 | 88994233 | rs4145712 | A/C |
| chr11 | 89597721 | 3 | 2 | chr11 | 89597720 | rs72958754 | G/T |
| chr11 | 91586255 | 5 | 1 | chr11 | 91586253 | rs1379802 | A/G |
| chr11 | 93383913 | 8 | 7 | chr11 | 93383911 | rs2020351 | A/G |

| | | | | | | | |
|---|---|---|---|---|---|---|---|
| chr11 | 93655567 | 2 | 8 | chr11 | 93655565 | rs525569 | A/C |
| chr11 | 94398707 | 2 | 3 | chr11 | 94398706 | rs10752685 | A/G |
| chr11 | 94398707 | 2 | 3 | chr11 | 94398706 | rs28412010 | A/G |
| chr11 | 94422571 | 2 | 3 | chr11 | 94422570 | rs28412010 | A/G |
| chr11 | 94620971 | 1 | 7 | chr11 | 94620970 | rs9888201 | A/G |
| chr11 | 95468082 | 1 | 4 | chr11 | 95468081 | rs10732450 | A/G |
| chr11 | 96749988 | 3 | 1 | chr11 | 96749986 | rs1365445 | A/G |
| chr11 | 96749988 | 3 | 1 | chr11 | 96749987 | rs12802951 | A/G |
| chr11 | 97076170 | 1 | 3 | chr11 | 97076169 | rs11212342 | A/G |
| chr11 | 97116671 | 2 | 7 | chr11 | 97116670 | rs1849142 | C/T |
| chr11 | 97759926 | 3 | 2 | chr11 | 97759925 | rs12290455 | A/G |
| chr11 | 98256790 | 1 | 10 | chr11 | 98256789 | rs1601688 | A/G |
| chr11 | 98605310 | 4 | 1 | chr11 | 98605308 | rs1461682 | G/T |
| chr11 | 99937655 | 2 | 4 | chr11 | 99937654 | rs7947335 | C/G |
| chr11 | 101193488 | 2 | 2 | chr11 | 101193487 | rs10459003 | C/G |
| chr11 | 101928869 | 3 | 3 | chr11 | 101928866 | rs10895313 | A/C |
| chr11 | 102629539 | 2 | 4 | chr11 | 102629537 | rs663457 | C/T |
| chr11 | 102629539 | 2 | 4 | chr11 | 102629538 | rs72971585 | A/G |
| chr11 | 104120606 | 10 | 3 | chr11 | 104120604 | rs10750713 | C/G |
| chr11 | 105345574 | 4 | 2 | chr11 | 105345573 | rs2155338 | A/G |
| chr11 | 105504791 | 2 | 3 | chr11 | 105504789 | rs1940770 | G/T |
| chr11 | 106554606 | 6 | 4 | chr11 | 106554604 | rs7949427 | C/T |
| chr11 | 106554606 | 6 | 4 | chr11 | 106554604 | rs71471737 | ACA/CCG |
| chr11 | 106554606 | 6 | 4 | chr11 | 106554606 | rs7949428 | G/T |
| chr11 | 108775645 | 3 | 6 | chr11 | 108775644 | rs12276077 | A/G |
| chr11 | 109327685 | 2 | 4 | chr11 | 109327682 | rs6650176 | C/T |
| chr11 | 110514584 | 1 | 3 | chr11 | 110514583 | rs4936336 | A/G |
| chr11 | 110988516 | 3 | 3 | chr11 | 110988515 | rs1784785 | A/G |
| chr11 | 111044536 | 5 | 2 | chr11 | 111044536 | rs4630327 | A/G |
| chr11 | 111141837 | 1 | 5 | chr11 | 111141836 | rs643506 | A/C |
| chr11 | 111640971 | 4 | 7 | chr11 | 111640969 | rs2564883 | A/G |
| chr11 | 111737051 | 8 | 4 | chr11 | 111737049 | rs1525415 | A/G |

| | | | | | | | |
|---|---|---|---|---|---|---|---|
| chr11 | 111737051 | 8 | 4 | chr11 | 111737049 | rs61598243 | -/T |
| chr11 | 111737051 | 8 | 4 | chr11 | 111737050 | rs59103466 | C/G |
| chr11 | 111751207 | 9 | 3 | chr11 | 111751206 | rs7102382 | C/G |
| chr11 | 111965946 | 4 | 5 | chr11 | 111965945 | rs12797014 | A/G |
| chr11 | 111992242 | 5 | 2 | chr11 | 111992240 | rs10466528 | C/T |
| chr11 | 112127980 | 3 | 2 | chr11 | 112127979 | rs7102916 | A/G |
| chr11 | 112192033 | 4 | 7 | chr11 | 112192031 | rs608535 | C/T |
| chr11 | 112937170 | 4 | 1 | chr11 | 112937169 | rs4936277 | A/G |
| chr11 | 113144443 | 1 | 4 | chr11 | 113144441 | rs2459970 | C/T |
| chr11 | 113437669 | 1 | 3 | chr11 | 113437669 | rs2509220 | A/G |
| chr11 | 113455362 | 2 | 3 | chr11 | 113455360 | rs1784691 | C/T |
| chr11 | 114567760 | 3 | 1 | chr11 | 114567759 | rs4245160 | A/G |
| chr11 | 114583703 | 3 | 1 | chr11 | 114583701 | rs3802858 | A/G |
| chr11 | 114992951 | 0 | 0 | chr11 | 114992949 | rs2846895 | A/G |
| chr11 | 115041421 | 1 | 3 | chr11 | 115041419 | rs1784998 | A/G |
| chr11 | 116698464 | 1 | 6 | chr11 | 116698463 | rs61903732 | A/G |
| chr11 | 116797186 | 2 | 2 | chr11 | 116797184 | rs1902627 | C/T |
| chr11 | 116874015 | 1 | 10 | chr11 | 116874014 | rs524968 | A/G |
| chr11 | 116886461 | 3 | 9 | chr11 | 116886460 | rs523825 | C/T |
| chr11 | 116949254 | 3 | 16 | chr11 | 116949253 | rs746910 | C/T |
| chr11 | 117001511 | 5 | 5 | chr11 | 117001510 | rs10892145 | A/G |
| chr11 | 119185528 | 10 | 3 | chr11 | 119185527 | rs6589778 | A/G |
| chr11 | 119189355 | 1 | 3 | chr11 | 119189354 | rs895647 | A/C |
| chr11 | 119355935 | 6 | 1 | chr11 | 119355934 | rs615539 | C/T |
| chr11 | 119439268 | 3 | 1 | chr11 | 119439267 | rs671334 | C/T |
| chr11 | 119444365 | 2 | 6 | chr11 | 119444365 | rs597253 | C/T |
| chr11 | 119582468 | 1 | 6 | chr11 | 119582468 | rs489454 | A/G |
| chr11 | 119891052 | 1 | 3 | chr11 | 119891050 | rs2243746 | C/T |
| chr11 | 120208127 | 3 | 2 | chr11 | 120208125 | rs12283648 | A/C |
| chr11 | 120487317 | 7 | 1 | chr11 | 120487314 | rs606916 | C/G |
| chr11 | 120622036 | 2 | 9 | chr11 | 120622033 | rs7115463 | C/T |
| chr11 | 122354183 | 2 | 2 | chr11 | 122354183 | rs1381246 | C/G |

| | | | | | | | |
|---|---|---|---|---|---|---|---|
| chr11 | 122592675 | 2 | 2 | chr11 | 122592675 | rs1790229 | G/T |
| chr11 | 122999063 | 1 | 5 | chr11 | 122999062 | rs1148116 | C/T |
| chr11 | 123218222 | 2 | 6 | chr11 | 123218220 | rs4936853 | C/T |
| chr11 | 123304643 | 1 | 4 | chr11 | 123304641 | rs72547747 | C/G |
| chr11 | 123304643 | 1 | 4 | chr11 | 123304642 | rs10750251 | C/G |
| chr11 | 124267979 | 5 | 2 | chr11 | 124267977 | rs61677926 | A/G |
| chr11 | 124267979 | 5 | 2 | chr11 | 124267978 | rs11219831 | A/G |
| chr11 | 124664438 | 1 | 3 | chr11 | 124664436 | rs12279920 | C/T |
| chr11 | 124664438 | 1 | 3 | chr11 | 124664437 | rs1863445 | A/G |
| chr11 | 124705457 | 5 | 2 | chr11 | 124705456 | rs4935918 | A/G |
| chr11 | 125208840 | 1 | 3 | chr11 | 125208838 | rs12224816 | C/T |
| chr11 | 125321079 | 1 | 5 | chr11 | 125321078 | rs636750 | C/T |
| chr11 | 125323909 | 4 | 1 | chr11 | 125323908 | rs634053 | C/T |
| chr11 | 125350668 | 2 | 7 | chr11 | 125350668 | rs676172 | A/G |
| chr11 | 125586614 | 2 | 2 | chr11 | 125586612 | rs2282580 | A/G |
| chr11 | 125591979 | 1 | 4 | chr11 | 125591977 | rs633607 | C/T |
| chr11 | 125791926 | 2 | 2 | chr11 | 125791923 | rs7925454 | A/C |
| chr11 | 126171494 | 3 | 1 | chr11 | 126171493 | rs4935993 | C/G |
| chr11 | 126476131 | 1 | 7 | chr11 | 126476130 | rs6590243 | A/G |
| chr11 | 127441678 | 3 | 5 | chr11 | 127441676 | rs1425871 | A/G |
| chr11 | 128134362 | 5 | 1 | chr11 | 128134361 | rs55859508 | G/T |
| chr11 | 128190565 | 3 | 2 | chr11 | 128190563 | rs549941 | A/C |
| chr11 | 128539035 | 1 | 3 | chr11 | 128539035 | rs506772 | C/T |
| chr11 | 128933298 | 3 | 2 | chr11 | 128933296 | rs7117782 | C/T |
| chr11 | 128933298 | 3 | 2 | chr11 | 128933297 | rs3019821 | C/T |
| chr11 | 129017036 | 1 | 3 | chr11 | 129017035 | rs1493272 | A/G |
| chr11 | 129077941 | 5 | 2 | chr11 | 129077940 | rs58506785 | A/G |
| chr11 | 129481546 | 5 | 1 | chr11 | 129481546 | rs34160650 | G/T |
| chr11 | 129821155 | 3 | 2 | chr11 | 129821153 | rs2220171 | A/C |
| chr11 | 130255853 | 1 | 6 | chr11 | 130255851 | rs2298566 | A/C |
| chr11 | 130272293 | 1 | 3 | chr11 | 130272291 | rs3794138 | A/G |
| chr11 | 130958223 | 3 | 7 | chr11 | 130958222 | rs12787028 | A/G |

| | | | | | | | |
|---|---|---|---|---|---|---|---|
| chr11 | 130959824 | 6 | 10 | chr11 | 130959823 | rs2458763 | A/C |
| chr11 | 131161032 | 5 | 2 | chr11 | 131161031 | rs4937654 | G/T |
| chr11 | 131291688 | 6 | 5 | chr11 | 131291685 | rs564406 | C/G |
| chr11 | 131536775 | 1 | 11 | chr11 | 131536774 | rs3936011 | C/T |
| chr11 | 131682844 | 7 | 4 | chr11 | 131682842 | rs2305271 | G/T |
| chr11 | 133207165 | 3 | 2 | chr11 | 133207165 | rs2097101 | C/T |
| chr11 | 133668987 | 1 | 4 | chr11 | 133668986 | rs512731 | A/G |
| chr11 | 133750495 | 1 | 3 | chr11 | 133750493 | rs9783402 | C/T |
| chr11 | 133847993 | 1 | 9 | chr11 | 133847990 | rs11223853 | C/T |
| chr11 | 133847993 | 1 | 9 | chr11 | 133847991 | rs4128665 | C/T |
| chr11 | 133875502 | 7 | 2 | chr11 | 133875500 | rs7950968 | C/T |
| chr11 | 133875502 | 7 | 2 | chr11 | 133875501 | rs11223876 | A/G |
| chr11 | 133889599 | 2 | 2 | chr11 | 133889596 | rs10750572 | C/G |
| chr12 | 147806 | 9 | 5 | chr12 | 147804 | rs4980860 | C/T |
| chr12 | 175516 | 4 | 1 | chr12 | 175514 | rs533572 | C/T |
| chr12 | 216212 | 2 | 7 | chr12 | 216210 | rs7960324 | C/T |
| chr12 | 219138 | 1 | 4 | chr12 | 219136 | rs10774020 | C/T |
| chr12 | 343226 | 11 | 2 | chr12 | 343225 | rs7298995 | A/G |
| chr12 | 537204 | 4 | 1 | chr12 | 537203 | rs7300933 | A/G |
| chr12 | 621985 | 14 | 3 | chr12 | 621983 | rs4980956 | A/C |
| chr12 | 1510703 | 1 | 3 | chr12 | 1510702 | rs929318 | C/G |
| chr12 | 1619096 | 2 | 13 | chr12 | 1619094 | rs2270036 | C/T |
| chr12 | 1825925 | 0 | 0 | chr12 | 1825924 | rs7137553 | G/T |
| chr12 | 1870539 | 3 | 1 | chr12 | 1870538 | rs2470393 | A/C |
| chr12 | 1896420 | 5 | 7 | chr12 | 1896418 | rs11062021 | C/T |
| chr12 | 2158698 | 4 | 3 | chr12 | 2158696 | rs10848626 | C/T |
| chr12 | 2170268 | 2 | 3 | chr12 | 2170268 | rs2238047 | A/G |
| chr12 | 2403766 | 5 | 1 | chr12 | 2403764 | rs994899 | C/G |
| chr12 | 2754569 | 3 | 1 | chr12 | 2754568 | rs4075718 | C/T |
| chr12 | 2825897 | 6 | 3 | chr12 | 2825896 | rs7139245 | A/G |
| chr12 | 2953970 | 2 | 5 | chr12 | 2953969 | rs4759436 | C/T |
| chr12 | 2957265 | 8 | 3 | chr12 | 2957263 | rs7975912 | C/T |

| | | | | | | | |
|---|---|---|---|---|---|---|---|
| chr12 | 2978997 | 9 | 1 | chr12 | 2978996 | rs4759435 | C/T |
| chr12 | 3157522 | 2 | 5 | chr12 | 3157520 | rs887362 | A/G |
| chr12 | 4003734 | 1 | 7 | chr12 | 4003732 | rs2532542 | C/T |
| chr12 | 4003734 | 1 | 7 | chr12 | 4003733 | rs74058918 | A/G |
| chr12 | 4011138 | 5 | 6 | chr12 | 4011136 | rs2540119 | C/T |
| chr12 | 5009755 | 5 | 1 | chr12 | 5009754 | rs9919707 | A/G |
| chr12 | 5925011 | 1 | 5 | chr12 | 5925008 | rs6489681 | C/T |
| chr12 | 5950796 | 6 | 8 | chr12 | 5950794 | rs10849365 | C/T |
| chr12 | 6749464 | 7 | 2 | chr12 | 6749462 | rs4764622 | A/G |
| chr12 | 6749540 | 6 | 10 | chr12 | 6749539 | rs4764621 | C/T |
| chr12 | 6931727 | 2 | 8 | chr12 | 6931726 | rs4963514 | C/T |
| chr12 | 8961846 | 3 | 4 | chr12 | 8961844 | rs4883199 | C/T |
| chr12 | 9366477 | 2 | 3 | chr12 | 9366476 | rs10843418 | A/G |
| chr12 | 9493631 | 5 | 4 | chr12 | 9493629 | rs34364780 | A/G |
| chr12 | 9493631 | 5 | 4 | chr12 | 9493631 | rs60859240 | A/G |
| chr12 | 9501299 | 3 | 2 | chr12 | 9501297 | rs11613863 | C/T |
| chr12 | 9501299 | 3 | 2 | chr12 | 9501297 | rs41360147 | C/T |
| chr12 | 10890976 | 1 | 4 | chr12 | 10890974 | rs1047699 | C/T |
| chr12 | 11091002 | 10 | 4 | chr12 | 11091000 | rs2597984 | C/T |
| chr12 | 11703868 | 10 | 2 | chr12 | 11703866 | rs759484 | A/G |
| chr12 | 11907172 | 4 | 3 | chr12 | 11907171 | rs10845414 | A/G |
| chr12 | 11938544 | 4 | 4 | chr12 | 11938543 | rs2156932 | A/G |
| chr12 | 12237160 | 1 | 3 | chr12 | 12237159 | rs11054724 | A/G |
| chr12 | 12508955 | 6 | 2 | chr12 | 12508954 | rs7303432 | A/G |
| chr12 | 12516856 | 6 | 2 | chr12 | 12516854 | rs2111271 | C/G |
| chr12 | 12703519 | 1 | 5 | chr12 | 12703518 | rs7309021 | A/G |
| chr12 | 13605364 | 2 | 3 | chr12 | 13605362 | rs7307302 | C/T |
| chr12 | 13677119 | 4 | 1 | chr12 | 13677118 | rs11055556 | A/G |
| chr12 | 14684381 | 1 | 6 | chr12 | 14684380 | rs2111178 | A/G |
| chr12 | 15095039 | 1 | 4 | chr12 | 15095037 | rs11056307 | C/T |
| chr12 | 15668754 | 2 | 5 | chr12 | 15668752 | rs4623959 | C/T |
| chr12 | 18597537 | 7 | 1 | chr12 | 18597535 | rs10505826 | C/T |

| | | | | | | | |
|---|---|---|---|---|---|---|---|
| chr12 | 19512987 | 6 | 2 | chr12 | 19512986 | rs7968825 | A/G |
| chr12 | 19618309 | 1 | 5 | chr12 | 19618307 | rs11044698 | C/G |
| chr12 | 19705474 | 2 | 8 | chr12 | 19705473 | rs6486998 | A/G |
| chr12 | 19877064 | 6 | 1 | chr12 | 19877062 | rs2694991 | A/G |
| chr12 | 20695428 | 7 | 3 | chr12 | 20695426 | rs6487125 | C/T |
| chr12 | 20797647 | 3 | 1 | chr12 | 20797645 | rs10841613 | A/C |
| chr12 | 22179302 | 5 | 1 | chr12 | 22179300 | rs10770893 | C/T |
| chr12 | 22822131 | 2 | 3 | chr12 | 22822129 | rs260887 | C/T |
| chr12 | 25006141 | 7 | 5 | chr12 | 25006140 | rs4350438 | A/G |
| chr12 | 25259106 | 4 | 1 | chr12 | 25259104 | rs4963859 | A/C |
| chr12 | 25360677 | 13 | 1 | chr12 | 25360676 | rs3885679 | C/T |
| chr12 | 25430584 | 2 | 2 | chr12 | 25430582 | rs7301334 | C/G |
| chr12 | 25551846 | 3 | 1 | chr12 | 25551845 | rs10743565 | A/G |
| chr12 | 25574763 | 1 | 8 | chr12 | 25574762 | rs279014 | A/G |
| chr12 | 26020242 | 1 | 6 | chr12 | 26020241 | rs56169892 | A/G |
| chr12 | 26404368 | 3 | 7 | chr12 | 26404366 | rs10842720 | C/T |
| chr12 | 27026056 | 3 | 2 | chr12 | 27026055 | rs17408108 | A/G |
| chr12 | 27206559 | 2 | 2 | chr12 | 27206557 | rs841623 | A/G |
| chr12 | 27206559 | 2 | 2 | chr12 | 27206558 | rs12322350 | G/T |
| chr12 | 27612851 | 2 | 4 | chr12 | 27612850 | rs10771342 | A/G |
| chr12 | 28897260 | 2 | 4 | chr12 | 28897258 | rs4931109 | C/T |
| chr12 | 28899164 | 5 | 3 | chr12 | 28899161 | rs7978472 | C/T |
| chr12 | 28914564 | 1 | 3 | chr12 | 28914563 | rs11049889 | A/G |
| chr12 | 30139762 | 2 | 2 | chr12 | 30139760 | rs11050652 | C/T |
| chr12 | 31108869 | 2 | 3 | chr12 | 31108868 | rs28884601 | A/G |
| chr12 | 31108869 | 2 | 3 | chr12 | 31108868 | rs41360147 | C/T |
| chr12 | 31265528 | 1 | 7 | chr12 | 31265527 | rs11051335 | A/G |
| chr12 | 32150854 | 1 | 15 | chr12 | 32150853 | rs1151017 | A/G |
| chr12 | 32371323 | 7 | 9 | chr12 | 32371322 | rs261889 | C/T |
| chr12 | 32999810 | 4 | 5 | chr12 | 32999808 | rs7133084 | C/T |
| chr12 | 34237985 | 2 | 3 | chr12 | 34237984 | rs73098223 | G/T |
| chr12 | 34255480 | 2 | 2 | chr12 | 34255479 | rs10743843 | G/T |

| | | | | | | | |
|---|---|---|---|---|---|---|---|
| chr12 | 36736697 | 13 | 1 | chr12 | 36736694 | rs7308707 | C/G |
| chr12 | 36834234 | 5 | 3 | chr12 | 36834232 | rs12425056 | C/T |
| chr12 | 38635770 | 1 | 7 | chr12 | 38635769 | rs1390952 | A/G |
| chr12 | 39309019 | 1 | 4 | chr12 | 39309017 | rs7310024 | A/C |
| chr12 | 39586558 | 1 | 3 | chr12 | 39586556 | rs17621741 | A/C |
| chr12 | 40043612 | 1 | 6 | chr12 | 40043611 | rs6582331 | A/G |
| chr12 | 40369657 | 11 | 1 | chr12 | 40369656 | rs7969921 | A/G |
| chr12 | 41331280 | 4 | 1 | chr12 | 41331279 | rs10880337 | A/G |
| chr12 | 44865857 | 5 | 4 | chr12 | 44865856 | rs11183385 | A/G |
| chr12 | 44900587 | 3 | 2 | chr12 | 44900586 | rs58776177 | A/G |
| chr12 | 44947654 | 3 | 2 | chr12 | 44947653 | rs1060735 | A/G |
| chr12 | 45339532 | 4 | 4 | chr12 | 45339531 | rs235618 | A/G |
| chr12 | 45482772 | 4 | 3 | chr12 | 45482771 | rs2465608 | C/T |
| chr12 | 46415946 | 3 | 3 | chr12 | 46415945 | rs11168214 | A/G |
| chr12 | 46462104 | 3 | 9 | chr12 | 46462103 | rs4760654 | A/G |
| chr12 | 46463340 | 3 | 5 | chr12 | 46463339 | rs2408874 | A/G |
| chr12 | 46464780 | 2 | 9 | chr12 | 46464779 | rs7972177 | A/G |
| chr12 | 46787678 | 2 | 2 | chr12 | 46787677 | rs10492080 | C/T |
| chr12 | 47629207 | 1 | 10 | chr12 | 47629205 | rs7963018 | C/T |
| chr12 | 48547093 | 2 | 2 | chr12 | 48547091 | rs1044677 | A/G |
| chr12 | 48625555 | 4 | 2 | chr12 | 48625554 | rs427758 | C/G |
| chr12 | 49673136 | 10 | 1 | chr12 | 49673133 | rs372144 | A/G |
| chr12 | 49673136 | 10 | 1 | chr12 | 49673134 | rs224570 | A/G |
| chr12 | 50068338 | 4 | 1 | chr12 | 50068336 | rs12316391 | C/G |
| chr12 | 50293270 | 4 | 2 | chr12 | 50293269 | rs1905248 | A/G |
| chr12 | 51031355 | 6 | 3 | chr12 | 51031353 | rs7953123 | C/T |
| chr12 | 51250046 | 3 | 3 | chr12 | 51250045 | rs73107558 | A/G |
| chr12 | 51251715 | 5 | 1 | chr12 | 51251714 | rs61927455 | A/G |
| chr12 | 51448474 | 4 | 3 | chr12 | 51448472 | rs2280481 | G/T |
| chr12 | 51502451 | 6 | 3 | chr12 | 51502450 | rs4415849 | A/G |
| chr12 | 52440441 | 5 | 3 | chr12 | 52440440 | rs11170678 | G/T |
| chr12 | 53553795 | 5 | 1 | chr12 | 53553794 | rs2370973 | C/T |

| | | | | | | | |
|---|---|---|---|---|---|---|---|
| chr12 | 54072157 | 1 | 9 | chr12 | 54072156 | rs7956183 | A/G |
| chr12 | 55892653 | 2 | 15 | chr12 | 55892652 | rs7304504 | A/G |
| chr12 | 55907477 | 0 | 0 | chr12 | 55907475 | rs6581130 | A/C |
| chr12 | 57483490 | 3 | 6 | chr12 | 57483489 | rs1148531 | A/G |
| chr12 | 59095877 | 2 | 2 | chr12 | 59095875 | rs1857729 | A/G |
| chr12 | 60068125 | 3 | 1 | chr12 | 60068124 | rs1516013 | C/T |
| chr12 | 60822902 | 4 | 1 | chr12 | 60822900 | rs1245446 | C/T |
| chr12 | 60897291 | 3 | 1 | chr12 | 60897290 | rs7314968 | A/G |
| chr12 | 61411399 | 4 | 2 | chr12 | 61411397 | rs12315418 | C/T |
| chr12 | 61736949 | 3 | 4 | chr12 | 61736948 | rs3913039 | C/T |
| chr12 | 61951321 | 2 | 2 | chr12 | 61951320 | rs35761509 | A/G |
| chr12 | 63263118 | 3 | 3 | chr12 | 63263116 | rs867400 | A/G |
| chr12 | 63598835 | 3 | 7 | chr12 | 63598834 | rs10878216 | A/G |
| chr12 | 63619574 | 3 | 2 | chr12 | 63619573 | rs462059 | C/T |
| chr12 | 63624725 | 2 | 4 | chr12 | 63624723 | rs424351 | A/G |
| chr12 | 64312413 | 6 | 2 | chr12 | 64312413 | rs7305981 | A/G |
| chr12 | 64400897 | 5 | 8 | chr12 | 64400896 | rs11175885 | A/G |
| chr12 | 65416902 | 5 | 2 | chr12 | 65416900 | rs10784579 | C/T |
| chr12 | 65748070 | 8 | 3 | chr12 | 65748068 | rs1526835 | C/T |
| chr12 | 65801361 | 5 | 1 | chr12 | 65801360 | rs1526839 | A/G |
| chr12 | 68616354 | 1 | 11 | chr12 | 68616353 | rs813147 | A/G |
| chr12 | 70638811 | 5 | 1 | chr12 | 70638809 | rs1386494 | A/G |
| chr12 | 72063152 | 2 | 4 | chr12 | 72063150 | rs7295470 | C/T |
| chr12 | 73756289 | 2 | 5 | chr12 | 73756288 | rs12371801 | A/G |
| chr12 | 77720704 | 2 | 3 | chr12 | 77720702 | rs12315768 | C/T |
| chr12 | 77864658 | 3 | 7 | chr12 | 77864656 | rs1913626 | C/T |
| chr12 | 78029268 | 3 | 1 | chr12 | 78029266 | rs11112829 | C/T |
| chr12 | 78640538 | 5 | 1 | chr12 | 78640537 | rs10778687 | A/G |
| chr12 | 78853374 | 7 | 6 | chr12 | 78853374 | rs3762111 | A/C |
| chr12 | 79907635 | 2 | 4 | chr12 | 79907633 | rs7961694 | C/T |
| chr12 | 79956653 | 5 | 7 | chr12 | 79956652 | rs10778781 | A/G |
| chr12 | 79968740 | 2 | 2 | chr12 | 79968739 | rs10862226 | A/G |

| | | | | | | | |
|---|---|---|---|---|---|---|---|
| chr12 | 81252855 | 4 | 6 | chr12 | 81252853 | rs2401037 | A/G |
| chr12 | 81268318 | 1 | 6 | chr12 | 81268317 | rs4495950 | A/G |
| chr12 | 82247712 | 2 | 2 | chr12 | 82247710 | rs73149470 | C/T |
| chr12 | 82426714 | 4 | 2 | chr12 | 82426713 | rs10862684 | G/T |
| chr12 | 84526494 | 3 | 1 | chr12 | 84526492 | rs11116923 | C/T |
| chr12 | 86281455 | 4 | 5 | chr12 | 86281454 | rs4269999 | A/G |
| chr12 | 88150438 | 5 | 4 | chr12 | 88150435 | rs73208333 | C/T |
| chr12 | 88417843 | 6 | 2 | chr12 | 88417841 | rs55824511 | C/T |
| chr12 | 88417843 | 6 | 2 | chr12 | 88417842 | rs10858884 | A/G |
| chr12 | 89909866 | 2 | 3 | chr12 | 89909864 | rs1920763 | A/G |
| chr12 | 91579461 | 6 | 3 | chr12 | 91579459 | rs7294830 | C/T |
| chr12 | 92385385 | 3 | 9 | chr12 | 92385383 | rs2290879 | C/T |
| chr12 | 92398427 | 0 | 0 | chr12 | 92398425 | rs10777518 | C/T |
| chr12 | 93059190 | 1 | 3 | chr12 | 93059189 | rs11614826 | C/G |
| chr12 | 93776005 | 6 | 2 | chr12 | 93776003 | rs10777639 | C/T |
| chr12 | 94626768 | 2 | 3 | chr12 | 94626766 | rs2468357 | A/G |
| chr12 | 94626768 | 2 | 3 | chr12 | 94626767 | rs6538662 | A/G |
| chr12 | 94779836 | 3 | 2 | chr12 | 94779834 | rs12820313 | C/T |
| chr12 | 96790283 | 4 | 2 | chr12 | 96790281 | rs1450066 | G/T |
| chr12 | 97435287 | 3 | 3 | chr12 | 97435286 | rs11109509 | A/G |
| chr12 | 98142724 | 3 | 1 | chr12 | 98142722 | rs2638572 | A/C |
| chr12 | 99238634 | 3 | 2 | chr12 | 99238633 | rs10860579 | A/G |
| chr12 | 99727771 | 3 | 2 | chr12 | 99727770 | rs10860636 | A/G |
| chr12 | 101769932 | 9 | 1 | chr12 | 101769931 | rs1722383 | C/T |
| chr12 | 103512500 | 1 | 5 | chr12 | 103512499 | rs10861247 | A/G |
| chr12 | 104885373 | 9 | 1 | chr12 | 104885371 | rs35819527 | C/T |
| chr12 | 106599990 | 1 | 3 | chr12 | 106599988 | rs11113437 | C/T |
| chr12 | 106715175 | 10 | 1 | chr12 | 106715173 | rs7307409 | C/T |
| chr12 | 107091514 | 3 | 4 | chr12 | 107091513 | rs4964645 | A/G |
| chr12 | 107236656 | 2 | 6 | chr12 | 107236655 | rs1399820 | A/G |
| chr12 | 107245625 | 11 | 10 | chr12 | 107245623 | rs10219723 | C/T |
| chr12 | 107282306 | 7 | 1 | chr12 | 107282304 | rs803556 | A/G |

| | | | | | | | |
|---|---|---|---|---|---|---|---|
| chr12 | 107762088 | 3 | 3 | chr12 | 107762087 | rs11114068 | C/G |
| chr12 | 108119460 | 4 | 1 | chr12 | 108119458 | rs2268392 | A/G |
| chr12 | 108604257 | 1 | 5 | chr12 | 108604256 | rs73202432 | A/G |
| chr12 | 109459274 | 15 | 3 | chr12 | 109459272 | rs10849900 | C/T |
| chr12 | 109894920 | 1 | 3 | chr12 | 109894919 | rs991817 | C/T |
| chr12 | 111821546 | 1 | 4 | chr12 | 111821545 | rs10492028 | A/G |
| chr12 | 112474593 | 3 | 2 | chr12 | 112474592 | rs11066600 | A/G |
| chr12 | 112847222 | 3 | 1 | chr12 | 112847220 | rs16943403 | C/T |
| chr12 | 112874071 | 1 | 4 | chr12 | 112874070 | rs60377154 | A/G |
| chr12 | 113291267 | 6 | 4 | chr12 | 113291266 | rs12314739 | A/G |
| chr12 | 113291267 | 6 | 4 | chr12 | 113291267 | rs1895583 | C/T |
| chr12 | 113603911 | 2 | 2 | chr12 | 113603909 | rs541524 | C/T |
| chr12 | 113825834 | 7 | 1 | chr12 | 113825832 | rs2384546 | C/G |
| chr12 | 114555641 | 4 | 2 | chr12 | 114555640 | rs11613384 | A/G |
| chr12 | 115369322 | 9 | 7 | chr12 | 115369320 | rs58787390 | C/T |
| chr12 | 115371337 | 16 | 4 | chr12 | 115371335 | rs56037837 | C/T |
| chr12 | 115430691 | 7 | 1 | chr12 | 115430689 | rs1241231 | C/G |
| chr12 | 115548610 | 19 | 2 | chr12 | 115548608 | rs7971781 | C/T |
| chr12 | 115637646 | 5 | 3 | chr12 | 115637645 | rs339460 | A/G |
| chr12 | 116375331 | 1 | 5 | chr12 | 116375329 | rs816284 | A/G |
| chr12 | 116569814 | 5 | 4 | chr12 | 116569812 | rs4767593 | C/T |
| chr12 | 116612402 | 2 | 2 | chr12 | 116612401 | rs7976208 | A/G |
| chr12 | 116630613 | 4 | 5 | chr12 | 116630612 | rs2393253 | A/G |
| chr12 | 117134744 | 4 | 9 | chr12 | 117134743 | rs353888 | C/T |
| chr12 | 117509010 | 5 | 1 | chr12 | 117509008 | rs2682759 | A/G |
| chr12 | 117939865 | 3 | 1 | chr12 | 117939863 | rs4767758 | C/G |
| chr12 | 117958469 | 5 | 5 | chr12 | 117958469 | rs1525971 | C/G |
| chr12 | 118305726 | 3 | 2 | chr12 | 118305726 | rs7297109 | C/G |
| chr12 | 118439956 | 5 | 1 | chr12 | 118439955 | rs1727428 | A/G |
| chr12 | 119239841 | 7 | 3 | chr12 | 119239841 | rs2522133 | A/G |
| chr12 | 119269028 | 1 | 12 | chr12 | 119269026 | rs1179433 | C/T |
| chr12 | 120011779 | 1 | 3 | chr12 | 120011778 | rs1180016 | A/G |

| | | | | | | | |
|---|---|---|---|---|---|---|---|
| chr12 | 121414051 | 6 | 4 | chr12 | 121414048 | rs2341439 | C/G |
| chr12 | 121685426 | 4 | 1 | chr12 | 121685424 | rs7397990 | C/T |
| chr12 | 121911314 | 3 | 2 | chr12 | 121911312 | rs7298176 | C/T |
| chr12 | 121915364 | 7 | 3 | chr12 | 121915362 | rs2061098 | A/G |
| chr12 | 123168456 | 0 | 0 | chr12 | 123168455 | rs446580 | C/T |
| chr12 | 123347645 | 5 | 6 | chr12 | 123347644 | rs10773079 | G/T |
| chr12 | 123391400 | 10 | 2 | chr12 | 123391399 | rs1702334 | C/T |
| chr12 | 123398559 | 2 | 4 | chr12 | 123398558 | rs1244063 | C/T |
| chr12 | 123401781 | 3 | 5 | chr12 | 123401779 | rs61934025 | A/C |
| chr12 | 123541227 | 2 | 2 | chr12 | 123541226 | rs12580511 | A/G |
| chr12 | 123550892 | 1 | 3 | chr12 | 123550891 | rs1242995 | C/T |
| chr12 | 123601366 | 4 | 4 | chr12 | 123601365 | rs701022 | C/T |
| chr12 | 123750413 | 3 | 3 | chr12 | 123750412 | rs838938 | C/T |
| chr12 | 123923856 | 5 | 5 | chr12 | 123923854 | rs10846763 | C/G |
| chr12 | 123955487 | 1 | 11 | chr12 | 123955484 | rs7301263 | A/C |
| chr12 | 123976515 | 5 | 1 | chr12 | 123976514 | rs7297676 | G/T |
| chr12 | 124156371 | 3 | 1 | chr12 | 124156369 | rs7970937 | C/T |
| chr12 | 124486597 | 1 | 5 | chr12 | 124486595 | rs7310173 | C/T |
| chr12 | 125602504 | 3 | 1 | chr12 | 125602502 | rs4997747 | C/T |
| chr12 | 125612469 | 4 | 1 | chr12 | 125612467 | rs10773298 | C/T |
| chr12 | 125812774 | 4 | 4 | chr12 | 125812772 | rs1194045 | C/T |
| chr12 | 126949772 | 3 | 2 | chr12 | 126949770 | rs10847474 | C/T |
| chr12 | 127169718 | 2 | 2 | chr12 | 127169717 | rs11613419 | A/G |
| chr12 | 127237063 | 10 | 1 | chr12 | 127237062 | rs67068200 | A/G |
| chr12 | 127362541 | 11 | 3 | chr12 | 127362539 | rs4882733 | C/G |
| chr12 | 127476203 | 1 | 4 | chr12 | 127476202 | rs10744376 | A/G |
| chr12 | 127574695 | 2 | 9 | chr12 | 127574694 | rs4627148 | A/G |
| chr12 | 127622510 | 1 | 4 | chr12 | 127622508 | rs4882787 | C/T |
| chr12 | 127853942 | 2 | 4 | chr12 | 127853940 | rs10773577 | C/T |
| chr12 | 128016786 | 1 | 4 | chr12 | 128016784 | rs602163 | C/T |
| chr12 | 128235045 | 5 | 1 | chr12 | 128235043 | rs57549565 | C/T |
| chr12 | 128235170 | 2 | 4 | chr12 | 128235168 | rs7294821 | C/T |

| | | | | | | | |
|---|---|---|---|---|---|---|---|
| chr12 | 128717448 | 4 | 4 | chr12 | 128717446 | rs628439 | C/G |
| chr12 | 128795934 | 1 | 5 | chr12 | 128795932 | rs11060528 | C/T |
| chr12 | 129511715 | 1 | 10 | chr12 | 129511713 | rs4759688 | C/T |
| chr12 | 129567422 | 3 | 2 | chr12 | 129567420 | rs4759709 | C/T |
| chr12 | 129643877 | 6 | 2 | chr12 | 129643876 | rs7301578 | C/G |
| chr12 | 129668932 | 1 | 7 | chr12 | 129668930 | rs34984549 | C/T |
| chr12 | 129753596 | 3 | 2 | chr12 | 129753595 | rs2695882 | C/T |
| chr12 | 129757812 | 8 | 5 | chr12 | 129757810 | rs2178076 | C/T |
| chr12 | 129765393 | 6 | 3 | chr12 | 129765392 | rs1918562 | A/G |
| chr12 | 129782527 | 1 | 5 | chr12 | 129782526 | rs1195576 | C/T |
| chr12 | 129784067 | 1 | 5 | chr12 | 129784065 | rs1195572 | A/G |
| chr12 | 130198243 | 1 | 12 | chr12 | 130198241 | rs10734987 | C/T |
| chr12 | 130402234 | 3 | 2 | chr12 | 130402233 | rs10848359 | A/G |
| chr12 | 130438307 | 3 | 1 | chr12 | 130438306 | rs7953324 | C/G |
| chr12 | 130484882 | 2 | 3 | chr12 | 130484881 | rs73153786 | A/G |
| chr12 | 130616044 | 0 | 0 | chr12 | 130616042 | rs6598149 | C/T |
| chr12 | 130896554 | 0 | 0 | chr12 | 130896554 | rs4964931 | G/T |
| chr12 | 130975204 | 8 | 1 | chr12 | 130975204 | rs10751707 | A/G |
| chr12 | 131192622 | 2 | 4 | chr12 | 131192620 | rs1133690 | A/G |
| chr12 | 131260266 | 2 | 3 | chr12 | 131260264 | rs10751710 | C/G |
| chr12 | 131322012 | 0 | 0 | chr12 | 131322011 | rs28972385 | A/G |
| chr12 | 131389747 | 2 | 5 | chr12 | 131389744 | rs34708049 | C/T |
| chr12 | 131389747 | 2 | 5 | chr12 | 131389744 | rs58703305 | -/TC |
| chr12 | 131407885 | 3 | 5 | chr12 | 131407883 | rs61945704 | C/T |
| chr12 | 131480975 | 0 | 0 | chr12 | 131480974 | rs35420162 | A/G |
| chr12 | 131494838 | 6 | 6 | chr12 | 131494836 | rs28753682 | A/C |
| chr12 | 131501658 | 18 | 5 | chr12 | 131501655 | rs28668197 | C/T |
| chr12 | 131520741 | 3 | 5 | chr12 | 131520740 | rs1574251 | C/T |
| chr12 | 131569972 | 2 | 4 | chr12 | 131569971 | rs10781666 | A/G |
| chr12 | 131573841 | 0 | 0 | chr12 | 131573840 | rs11610045 | A/G |
| chr12 | 132202403 | 2 | 5 | chr12 | 132202401 | rs12422899 | C/T |
| chr13 | 18630722 | 0 | 0 | chr13 | 18630720 | rs34590375 | C/T |

| | | | | | | | |
|---|---|---|---|---|---|---|---|
| chr13 | 18816645 | 2 | 3 | chr13 | 18816645 | rs7986947 | A/G |
| chr13 | 18816645 | 2 | 3 | chr13 | 18816645 | rs71421776 | A/G |
| chr13 | 18931956 | 2 | 3 | chr13 | 18931955 | rs2497244 | A/G |
| chr13 | 18931956 | 2 | 3 | chr13 | 18931955 | rs72621787 | A/G |
| chr13 | 19336884 | 1 | 5 | chr13 | 19336882 | rs711831 | C/G |
| chr13 | 19550255 | 2 | 5 | chr13 | 19550253 | rs259780 | C/T |
| chr13 | 19590896 | 1 | 3 | chr13 | 19590894 | rs1009054 | C/T |
| chr13 | 19788333 | 2 | 3 | chr13 | 19788332 | rs1832141 | C/T |
| chr13 | 19888323 | 4 | 1 | chr13 | 19888322 | rs4769108 | A/G |
| chr13 | 19908626 | 2 | 4 | chr13 | 19908624 | rs4770030 | C/G |
| chr13 | 21168472 | 4 | 5 | chr13 | 21168471 | rs7337457 | A/G |
| chr13 | 21551053 | 9 | 2 | chr13 | 21551052 | rs3945860 | A/G |
| chr13 | 21592562 | 2 | 2 | chr13 | 21592561 | rs4769197 | G/T |
| chr13 | 21718494 | 5 | 2 | chr13 | 21718492 | rs951903 | A/C |
| chr13 | 21718494 | 5 | 2 | chr13 | 21718494 | rs951905 | A/G |
| chr13 | 22310523 | 7 | 3 | chr13 | 22310523 | rs7333531 | A/G |
| chr13 | 22371263 | 1 | 5 | chr13 | 22371261 | rs9510412 | A/C |
| chr13 | 22580836 | 4 | 1 | chr13 | 22580835 | rs1336846 | G/T |
| chr13 | 22655670 | 1 | 4 | chr13 | 22655668 | rs598656 | C/T |
| chr13 | 22806034 | 1 | 5 | chr13 | 22806033 | rs2737700 | C/T |
| chr13 | 23365343 | 5 | 3 | chr13 | 23365341 | rs7331047 | C/T |
| chr13 | 23751645 | 2 | 2 | chr13 | 23751644 | rs4769339 | C/G |
| chr13 | 24014371 | 3 | 2 | chr13 | 24014368 | rs61946881 | C/T |
| chr13 | 24014371 | 3 | 2 | chr13 | 24014368 | rs71421776 | A/G |
| chr13 | 24667846 | 13 | 1 | chr13 | 24667845 | rs10747197 | A/G |
| chr13 | 25837906 | 11 | 1 | chr13 | 25837905 | rs7336578 | G/T |
| chr13 | 26243887 | 2 | 2 | chr13 | 26243886 | rs9507787 | -/A/G |
| chr13 | 26410509 | 8 | 2 | chr13 | 26410508 | rs1410431 | A/C |
| chr13 | 26773302 | 3 | 3 | chr13 | 26773300 | rs9319356 | C/T |
| chr13 | 26955292 | 3 | 5 | chr13 | 26955291 | rs1535641 | A/G |
| chr13 | 27198202 | 3 | 2 | chr13 | 27198200 | rs4769565 | C/T |
| chr13 | 27324836 | 1 | 6 | chr13 | 27324834 | rs2892224 | C/T |

| | | | | | | | |
|---|---|---|---|---|---|---|---|
| chr13 | 27324836 | 1 | 6 | chr13 | 27324834 | rs67665786 | CCG/GCA |
| chr13 | 27324836 | 1 | 6 | chr13 | 27324836 | rs2892225 | C/G |
| chr13 | 27358658 | 27 | 1 | chr13 | 27358657 | rs2892227 | A/G |
| chr13 | 27485135 | 4 | 7 | chr13 | 27485134 | rs9507977 | A/G |
| chr13 | 28268358 | 3 | 4 | chr13 | 28268355 | rs9508153 | C/G |
| chr13 | 28397756 | 2 | 3 | chr13 | 28397755 | rs327119 | C/T |
| chr13 | 28426155 | 1 | 6 | chr13 | 28426153 | rs6490337 | C/T |
| chr13 | 28426155 | 1 | 6 | chr13 | 28426154 | rs647767 | A/G |
| chr13 | 28426155 | 1 | 6 | chr13 | 28426154 | rs66996220 | CA/TG |
| chr13 | 28463869 | 4 | 1 | chr13 | 28463867 | rs8002557 | C/T |
| chr13 | 29071267 | 2 | 4 | chr13 | 29071266 | rs529696 | C/T |
| chr13 | 29566539 | 14 | 3 | chr13 | 29566537 | rs658198 | C/T |
| chr13 | 29630041 | 3 | 1 | chr13 | 29630040 | rs12860032 | A/G |
| chr13 | 29828188 | 1 | 4 | chr13 | 29828187 | rs2149853 | A/G |
| chr13 | 30295180 | 7 | 1 | chr13 | 30295179 | rs9741673 | A/G |
| chr13 | 30302026 | 1 | 6 | chr13 | 30302025 | rs4769893 | A/G |
| chr13 | 30378371 | 1 | 5 | chr13 | 30378370 | rs11617899 | A/G |
| chr13 | 30480469 | 5 | 3 | chr13 | 30480467 | rs4941562 | C/T |
| chr13 | 32378144 | 4 | 2 | chr13 | 32378143 | rs481686 | A/G |
| chr13 | 33215422 | 3 | 6 | chr13 | 33215420 | rs7333219 | C/T |
| chr13 | 35128486 | 2 | 2 | chr13 | 35128484 | rs9544915 | C/T |
| chr13 | 35593347 | 2 | 2 | chr13 | 35593346 | rs9575331 | A/G |
| chr13 | 36088213 | 3 | 3 | chr13 | 36088211 | rs618055 | C/T |
| chr13 | 36749814 | 3 | 5 | chr13 | 36749812 | rs1324047 | A/G |
| chr13 | 38018051 | 6 | 8 | chr13 | 38018050 | rs2323885 | A/G |
| chr13 | 38677916 | 1 | 9 | chr13 | 38677914 | rs7335737 | C/T |
| chr13 | 38685407 | 1 | 8 | chr13 | 38685405 | rs7324440 | C/T |
| chr13 | 39781324 | 9 | 13 | chr13 | 39781323 | rs4943765 | A/G |
| chr13 | 40784568 | 6 | 2 | chr13 | 40784567 | rs9525471 | A/G |
| chr13 | 40860865 | 1 | 6 | chr13 | 40860864 | rs4281609 | A/G |
| chr13 | 40942508 | 2 | 9 | chr13 | 40942507 | rs396963 | A/G |
| chr13 | 41823629 | 0 | 0 | chr13 | 41823627 | rs238258 | A/G |

| | | | | | | | | |
|---|---|---|---|---|---|---|---|---|
| chr13 | 41968206 | 2 | 3 | chr13 | 41968205 | rs35860234 | G/T |
| chr13 | 42362132 | 1 | 4 | chr13 | 42362130 | rs9533291 | C/T |
| chr13 | 42362132 | 1 | 4 | chr13 | 42362131 | rs9533292 | A/G |
| chr13 | 42781849 | 3 | 1 | chr13 | 42781847 | rs9316012 | C/T |
| chr13 | 43103683 | 4 | 1 | chr13 | 43103682 | rs9562501 | A/G |
| chr13 | 43915345 | 1 | 3 | chr13 | 43915343 | rs2325130 | C/T |
| chr13 | 44812958 | 1 | 3 | chr13 | 44812956 | rs2234216 | A/G |
| chr13 | 45262515 | 4 | 6 | chr13 | 45262514 | rs1536183 | A/G |
| chr13 | 45387308 | 2 | 2 | chr13 | 45387305 | rs2573278 | A/G |
| chr13 | 45837900 | 4 | 6 | chr13 | 45837898 | rs2478043 | A/G |
| chr13 | 46705628 | 2 | 2 | chr13 | 46705626 | rs7985427 | C/T |
| chr13 | 47056090 | 1 | 4 | chr13 | 47056088 | rs9534693 | C/T |
| chr13 | 47970064 | 3 | 3 | chr13 | 47970063 | rs9535035 | A/G |
| chr13 | 48398192 | 3 | 3 | chr13 | 48398191 | rs2596246 | C/T |
| chr13 | 48986358 | 2 | 2 | chr13 | 48986357 | rs7988060 | A/G |
| chr13 | 49021624 | 6 | 1 | chr13 | 49021622 | rs3751384 | C/G |
| chr13 | 49112314 | 1 | 3 | chr13 | 49112314 | rs61959911 | G/T |
| chr13 | 50503961 | 3 | 4 | chr13 | 50503959 | rs7994604 | C/T |
| chr13 | 50814168 | 11 | 2 | chr13 | 50814167 | rs9535647 | A/G |
| chr13 | 51584242 | 2 | 3 | chr13 | 51584240 | rs9535866 | C/T |
| chr13 | 51920519 | 4 | 2 | chr13 | 51920518 | rs4286007 | A/G |
| chr13 | 54398762 | 4 | 10 | chr13 | 54398760 | rs12585613 | C/T |
| chr13 | 54729944 | 3 | 1 | chr13 | 54729942 | rs9563305 | C/T |
| chr13 | 54861931 | 3 | 1 | chr13 | 54861930 | rs3105043 | C/T |
| chr13 | 55050280 | 3 | 5 | chr13 | 55050279 | rs34530982 | A/G |
| chr13 | 56614014 | 2 | 14 | chr13 | 56614013 | rs1217726 | A/G |
| chr13 | 56615371 | 3 | 2 | chr13 | 56615370 | rs9597513 | A/G |
| chr13 | 56620584 | 2 | 14 | chr13 | 56620583 | rs1217726 | A/G |
| chr13 | 56621941 | 3 | 2 | chr13 | 56621940 | rs9597513 | A/G |
| chr13 | 56627157 | 2 | 14 | chr13 | 56627156 | rs1217726 | A/G |
| chr13 | 56628514 | 3 | 2 | chr13 | 56628513 | rs9597513 | A/G |
| chr13 | 56633728 | 2 | 14 | chr13 | 56633727 | rs1217726 | A/G |

| | | | | | | | |
|---|---|---|---|---|---|---|---|
| chr13 | 56635085 | 3 | 2 | chr13 | 56635084 | rs9597513 | A/G |
| chr13 | 56640293 | 2 | 14 | chr13 | 56640292 | rs1217726 | A/G |
| chr13 | 56641650 | 3 | 2 | chr13 | 56641649 | rs9597513 | A/G |
| chr13 | 56757902 | 11 | 5 | chr13 | 56757901 | rs9527635 | C/G |
| chr13 | 58662982 | 1 | 5 | chr13 | 58662980 | rs7329781 | C/T |
| chr13 | 59200340 | 3 | 1 | chr13 | 59200338 | rs342587 | A/G |
| chr13 | 60152720 | 3 | 2 | chr13 | 60152718 | rs9538868 | C/T |
| chr13 | 60197398 | 1 | 4 | chr13 | 60197397 | rs1360192 | C/T |
| chr13 | 62546483 | 1 | 5 | chr13 | 62546482 | rs73503643 | A/G |
| chr13 | 63655986 | 4 | 3 | chr13 | 63655985 | rs9317395 | A/G |
| chr13 | 64166129 | 4 | 3 | chr13 | 64166127 | rs9317435 | A/C |
| chr13 | 67920757 | 3 | 5 | chr13 | 67920757 | rs73198046 | G/T |
| chr13 | 68651338 | 10 | 3 | chr13 | 68651336 | rs9541808 | C/T |
| chr13 | 68863566 | 1 | 4 | chr13 | 68863565 | rs2782481 | A/G |
| chr13 | 69758307 | 4 | 3 | chr13 | 69758305 | rs7982253 | C/T |
| chr13 | 71462446 | 2 | 2 | chr13 | 71462444 | rs2706440 | A/G |
| chr13 | 72376181 | 5 | 1 | chr13 | 72376178 | rs7984920 | A/C |
| chr13 | 72425952 | 2 | 3 | chr13 | 72425951 | rs9530120 | A/G |
| chr13 | 72983005 | 4 | 7 | chr13 | 72983003 | rs1415409 | A/G |
| chr13 | 73878962 | 2 | 2 | chr13 | 73878960 | rs9543623 | C/T |
| chr13 | 73878962 | 2 | 2 | chr13 | 73878960 | rs35760968 | -/C |
| chr13 | 73937508 | 10 | 1 | chr13 | 73937507 | rs9318278 | A/G |
| chr13 | 74871754 | 6 | 1 | chr13 | 74871752 | rs4334162 | C/T |
| chr13 | 76513310 | 3 | 2 | chr13 | 76513308 | rs9565315 | C/T |
| chr13 | 77619297 | 2 | 3 | chr13 | 77619295 | rs2209375 | A/G |
| chr13 | 80044542 | 1 | 3 | chr13 | 80044540 | rs6563178 | C/T |
| chr13 | 80948843 | 3 | 2 | chr13 | 80948841 | rs1984109 | C/T |
| chr13 | 80948843 | 3 | 2 | chr13 | 80948842 | rs9601616 | A/G |
| chr13 | 82594757 | 1 | 9 | chr13 | 82594756 | rs265697 | A/G |
| chr13 | 84081381 | 6 | 3 | chr13 | 84081379 | rs9565948 | C/T |
| chr13 | 85149794 | 2 | 3 | chr13 | 85149792 | rs61970114 | A/C |
| chr13 | 85714506 | 1 | 13 | chr13 | 85714505 | rs7399444 | A/G |

| | | | | | | | |
|---|---|---|---|---|---|---|---|
| chr13 | 86035563 | 2 | 2 | chr13 | 86035562 | rs7996543 | A/G |
| chr13 | 86208285 | 2 | 3 | chr13 | 86208284 | rs9556075 | A/G |
| chr13 | 87517764 | 1 | 5 | chr13 | 87517761 | rs11319869 | -/C |
| chr13 | 87517764 | 1 | 5 | chr13 | 87517763 | rs1559906 | C/T |
| chr13 | 87781690 | 3 | 2 | chr13 | 87781688 | rs186833 | A/G |
| chr13 | 87785684 | 3 | 1 | chr13 | 87785683 | rs1335487 | C/T |
| chr13 | 91176559 | 2 | 5 | chr13 | 91176557 | rs16952625 | A/C |
| chr13 | 92653226 | 6 | 3 | chr13 | 92653225 | rs433203 | A/G |
| chr13 | 94056946 | 1 | 4 | chr13 | 94056944 | rs6492721 | C/T |
| chr13 | 94203250 | 5 | 2 | chr13 | 94203248 | rs9524606 | C/T |
| chr13 | 94220947 | 3 | 1 | chr13 | 94220946 | rs9524609 | A/G |
| chr13 | 94364512 | 7 | 1 | chr13 | 94364511 | rs1952338 | A/G |
| chr13 | 94530362 | 4 | 1 | chr13 | 94530362 | rs1729752 | C/G |
| chr13 | 94643664 | 1 | 4 | chr13 | 94643662 | rs1751015 | C/T |
| chr13 | 94678484 | 2 | 3 | chr13 | 94678483 | rs4773856 | A/G |
| chr13 | 94776000 | 1 | 4 | chr13 | 94775998 | rs7982036 | C/T |
| chr13 | 95068502 | 2 | 2 | chr13 | 95068500 | rs7992753 | A/C |
| chr13 | 95366127 | 3 | 1 | chr13 | 95366126 | rs9561981 | A/G |
| chr13 | 95646818 | 1 | 5 | chr13 | 95646817 | rs7987437 | A/G |
| chr13 | 97547828 | 5 | 9 | chr13 | 97547826 | rs602626 | C/T |
| chr13 | 97547828 | 5 | 9 | chr13 | 97547827 | rs9513350 | A/G |
| chr13 | 97627178 | 4 | 1 | chr13 | 97627176 | rs628778 | A/G |
| chr13 | 98195915 | 3 | 8 | chr13 | 98195911 | rs72545518 | C/T |
| chr13 | 98195915 | 3 | 8 | chr13 | 98195912 | rs4772132 | C/T |
| chr13 | 98586218 | 5 | 1 | chr13 | 98586217 | rs2390193 | G/T |
| chr13 | 98836234 | 6 | 1 | chr13 | 98836233 | rs1058083 | A/G |
| chr13 | 98934035 | 2 | 2 | chr13 | 98934033 | rs9517745 | C/T |
| chr13 | 99549906 | 5 | 6 | chr13 | 99549905 | rs7991210 | A/G |
| chr13 | 99772100 | 1 | 3 | chr13 | 99772098 | rs2390383 | A/C |
| chr13 | 100746191 | 1 | 4 | chr13 | 100746189 | rs565574 | C/T |
| chr13 | 101243545 | 3 | 1 | chr13 | 101243543 | rs10851137 | C/T |
| chr13 | 101243545 | 3 | 1 | chr13 | 101243543 | rs9518549 | C/T |

| | | | | | | | |
|---|---|---|---|---|---|---|---|
| chr13 | 101742068 | 8 | 1 | chr13 | 101742066 | rs2210898 | C/T |
| chr13 | 102502105 | 12 | 1 | chr13 | 102502104 | rs183965 | A/G |
| chr13 | 102706612 | 9 | 1 | chr13 | 102706610 | rs1529281 | A/G |
| chr13 | 103093036 | 2 | 3 | chr13 | 103093034 | rs7323477 | C/T |
| chr13 | 103160336 | 6 | 1 | chr13 | 103160334 | rs9519141 | C/T |
| chr13 | 104075538 | 1 | 5 | chr13 | 104075537 | rs4534697 | A/G |
| chr13 | 104202917 | 2 | 8 | chr13 | 104202915 | rs323432 | A/G |
| chr13 | 104286375 | 4 | 1 | chr13 | 104286375 | rs2031635 | A/G |
| chr13 | 104304572 | 2 | 3 | chr13 | 104304572 | rs8001367 | A/G |
| chr13 | 104391426 | 1 | 5 | chr13 | 104391424 | rs9300978 | C/T |
| chr13 | 104513928 | 1 | 3 | chr13 | 104513926 | rs9514350 | C/T |
| chr13 | 104984773 | 3 | 2 | chr13 | 104984771 | rs7331815 | C/T |
| chr13 | 105448745 | 1 | 4 | chr13 | 105448744 | rs4483718 | G/T |
| chr13 | 105583267 | 3 | 3 | chr13 | 105583265 | rs1372791 | C/T |
| chr13 | 105848918 | 3 | 6 | chr13 | 105848916 | rs9520053 | C/T |
| chr13 | 105909891 | 8 | 2 | chr13 | 105909889 | rs9520076 | C/T |
| chr13 | 108557080 | 6 | 2 | chr13 | 108557078 | rs157009 | C/T |
| chr13 | 109049613 | 4 | 2 | chr13 | 109049613 | rs9521437 | A/G |
| chr13 | 109292778 | 1 | 4 | chr13 | 109292777 | rs336241 | C/T |
| chr13 | 109456086 | 4 | 4 | chr13 | 109456084 | rs2225778 | A/G |
| chr13 | 109574734 | 2 | 2 | chr13 | 109574733 | rs9515150 | A/G |
| chr13 | 109706778 | 1 | 3 | chr13 | 109706777 | rs562992 | A/C |
| chr13 | 109761064 | 4 | 2 | chr13 | 109761062 | rs4773146 | C/T |
| chr13 | 109761090 | 5 | 3 | chr13 | 109761087 | rs4773147 | C/T |
| chr13 | 109797869 | 3 | 3 | chr13 | 109797867 | rs4771668 | C/T |
| chr13 | 109797869 | 3 | 3 | chr13 | 109797868 | rs4771669 | A/G |
| chr13 | 109870467 | 1 | 3 | chr13 | 109870466 | rs1927348 | C/T |
| chr13 | 109901550 | 7 | 5 | chr13 | 109901548 | rs10851244 | C/T |
| chr13 | 109953775 | 15 | 3 | chr13 | 109953773 | rs438758 | A/G |
| chr13 | 109971981 | 1 | 5 | chr13 | 109971980 | rs4773204 | A/G |
| chr13 | 109975673 | 1 | 3 | chr13 | 109975672 | rs2479429 | A/G |
| chr13 | 110482164 | 5 | 1 | chr13 | 110482162 | rs7321094 | C/T |

| | | | | | | | |
|---|---|---|---|---|---|---|---|
| chr13 | 110779401 | 4 | 1 | chr13 | 110779399 | rs2479956 | C/T |
| chr13 | 110796676 | 5 | 2 | chr13 | 110796675 | rs486322 | C/T |
| chr13 | 110821314 | 4 | 2 | chr13 | 110821312 | rs2770246 | A/G |
| chr13 | 110840118 | 1 | 3 | chr13 | 110840117 | rs1164146 | C/G |
| chr13 | 110849990 | 4 | 2 | chr13 | 110849988 | rs1164135 | C/T |
| chr13 | 110855888 | 3 | 7 | chr13 | 110855887 | rs12585085 | A/G |
| chr13 | 110855888 | 3 | 7 | chr13 | 110855888 | rs56343836 | A/G |
| chr13 | 110877636 | 5 | 1 | chr13 | 110877633 | rs1163847 | C/T |
| chr13 | 110886022 | 3 | 7 | chr13 | 110886020 | rs8000060 | C/T |
| chr13 | 110934707 | 1 | 3 | chr13 | 110934705 | rs1183183 | A/C |
| chr13 | 110936931 | 2 | 7 | chr13 | 110936929 | rs1151451 | C/T |
| chr13 | 111060074 | 1 | 3 | chr13 | 111060073 | rs9515459 | A/G |
| chr13 | 111095193 | 3 | 3 | chr13 | 111095192 | rs4773416 | A/G |
| chr13 | 111109205 | 1 | 4 | chr13 | 111109203 | rs4773420 | C/T |
| chr13 | 111579067 | 1 | 7 | chr13 | 111579066 | rs9549439 | A/G |
| chr13 | 111601539 | 3 | 4 | chr13 | 111601538 | rs4553492 | A/G |
| chr13 | 111610773 | 2 | 12 | chr13 | 111610772 | rs4907707 | A/G |
| chr13 | 111827123 | 3 | 5 | chr13 | 111827122 | rs9549850 | A/G |
| chr13 | 112145685 | 4 | 3 | chr13 | 112145684 | rs2149216 | C/T |
| chr13 | 112342972 | 6 | 4 | chr13 | 112342971 | rs9550180 | A/G |
| chr13 | 112419311 | 3 | 7 | chr13 | 112419309 | rs282570 | A/G |
| chr13 | 112515619 | 4 | 2 | chr13 | 112515616 | rs9324330 | C/T |
| chr13 | 112571992 | 2 | 8 | chr13 | 112571990 | rs4907552 | C/T |
| chr13 | 112605722 | 1 | 15 | chr13 | 112605721 | rs1890203 | A/G |
| chr13 | 112608381 | 4 | 4 | chr13 | 112608379 | rs1320526 | C/T |
| chr13 | 112613429 | 1 | 5 | chr13 | 112613428 | rs9603802 | C/G |
| chr13 | 112703637 | 4 | 1 | chr13 | 112703636 | rs3742239 | C/T |
| chr13 | 112729077 | 2 | 2 | chr13 | 112729076 | rs2993308 | A/G |
| chr13 | 112752333 | 8 | 1 | chr13 | 112752332 | rs4578540 | A/G |
| chr13 | 112804541 | 3 | 1 | chr13 | 112804540 | rs555212 | A/G |
| chr13 | 113051918 | 6 | 5 | chr13 | 113051917 | rs9577516 | A/G |
| chr13 | 113106886 | 1 | 3 | chr13 | 113106884 | rs7334623 | C/T |

| | | | | | | | |
|---|---|---|---|---|---|---|---|
| chr13 | 113359882 | 1 | 5 | chr13 | 113359880 | rs9670412 | C/T |
| chr13 | 113555136 | 11 | 1 | chr13 | 113555134 | rs56865280 | C/T |
| chr13 | 113591348 | 2 | 3 | chr13 | 113591347 | rs7999037 | A/G |
| chr13 | 113834642 | 0 | 0 | chr13 | 113834640 | rs9525353 | C/G |
| chr13 | 113834642 | 0 | 0 | chr13 | 113834641 | rs9525354 | C/G |
| chr13 | 113834642 | 0 | 0 | chr13 | 113834641 | rs68158955 | CG/GC |
| chr13 | 113891470 | 1 | 4 | chr13 | 113891469 | rs9314892 | A/G |
| chr13 | 113948458 | 3 | 2 | chr13 | 113948456 | rs445307 | A/G |
| chr14 | 18863939 | 1 | 8 | chr14 | 18863938 | rs4456412 | C/T |
| chr14 | 19973190 | 4 | 7 | chr14 | 19973188 | rs1713408 | A/G |
| chr14 | 19978582 | 6 | 5 | chr14 | 19978580 | rs938889 | A/G |
| chr14 | 20191634 | 10 | 1 | chr14 | 20191632 | rs17277550 | C/T |
| chr14 | 20299673 | 4 | 2 | chr14 | 20299671 | rs35783402 | A/C |
| chr14 | 20569724 | 1 | 3 | chr14 | 20569723 | rs1243461 | A/G |
| chr14 | 21203837 | 1 | 6 | chr14 | 21203836 | rs970382 | A/G |
| chr14 | 21429113 | 12 | 1 | chr14 | 21429111 | rs2280758 | C/T |
| chr14 | 21710679 | 4 | 5 | chr14 | 21710677 | rs12881037 | C/T |
| chr14 | 21919804 | 10 | 3 | chr14 | 21919802 | rs2204983 | C/T |
| chr14 | 21919804 | 10 | 3 | chr14 | 21919803 | rs4982620 | A/G |
| chr14 | 21926266 | 1 | 9 | chr14 | 21926265 | rs3811249 | C/T |
| chr14 | 21930286 | 6 | 4 | chr14 | 21930285 | rs7148380 | A/G |
| chr14 | 21943150 | 1 | 5 | chr14 | 21943148 | rs10149541 | C/T |
| chr14 | 22387665 | 1 | 5 | chr14 | 22387664 | rs17882077 | A/G |
| chr14 | 23236605 | 1 | 4 | chr14 | 23236604 | rs60719220 | A/G |
| chr14 | 24020907 | 1 | 4 | chr14 | 24020906 | rs911375 | A/C |
| chr14 | 24252537 | 5 | 1 | chr14 | 24252536 | rs762084 | A/G |
| chr14 | 25559140 | 6 | 1 | chr14 | 25559138 | rs72663191 | C/T |
| chr14 | 25598185 | 2 | 4 | chr14 | 25598184 | rs61986124 | C/G |
| chr14 | 26713573 | 4 | 1 | chr14 | 26713571 | rs1245400 | A/G |
| chr14 | 27106044 | 6 | 2 | chr14 | 27106043 | rs1954677 | C/T |
| chr14 | 27429497 | 1 | 5 | chr14 | 27429495 | rs6575914 | C/T |
| chr14 | 27536869 | 3 | 1 | chr14 | 27536867 | rs2251721 | C/T |

| | | | | | | | |
|---|---|---|---|---|---|---|---|
| chr14 | 29032504 | 1 | 4 | chr14 | 29032502 | rs1191569 | A/G |
| chr14 | 30306294 | 6 | 2 | chr14 | 30306292 | rs179531 | A/G |
| chr14 | 31300293 | 2 | 4 | chr14 | 31300290 | rs7493577 | C/G |
| chr14 | 31488986 | 2 | 3 | chr14 | 31488985 | rs1278941 | A/G |
| chr14 | 31574185 | 1 | 3 | chr14 | 31574184 | rs6571491 | G/T |
| chr14 | 31950022 | 6 | 10 | chr14 | 31950021 | rs35534415 | A/G |
| chr14 | 32667333 | 3 | 1 | chr14 | 32667331 | rs10139424 | C/T |
| chr14 | 36354713 | 2 | 2 | chr14 | 36354712 | rs1408968 | C/G |
| chr14 | 36673432 | 6 | 2 | chr14 | 36673431 | rs6571788 | A/G |
| chr14 | 40691934 | 1 | 10 | chr14 | 40691933 | rs9989148 | A/G |
| chr14 | 41144274 | 1 | 4 | chr14 | 41144273 | rs1950834 | A/G |
| chr14 | 46027673 | 2 | 5 | chr14 | 46027671 | rs1389607 | A/G |
| chr14 | 46126737 | 1 | 3 | chr14 | 46126736 | rs11845763 | A/G |
| chr14 | 47602929 | 4 | 5 | chr14 | 47602927 | rs698330 | C/T |
| chr14 | 47904917 | 2 | 2 | chr14 | 47904915 | rs1683210 | C/T |
| chr14 | 49027083 | 3 | 1 | chr14 | 49027081 | rs12323543 | C/T |
| chr14 | 49135109 | 2 | 3 | chr14 | 49135108 | rs2281837 | C/G |
| chr14 | 49333701 | 1 | 3 | chr14 | 49333700 | rs56053777 | G/T |
| chr14 | 49404910 | 0 | 0 | chr14 | 49404910 | rs11850738 | C/G |
| chr14 | 49551677 | 1 | 8 | chr14 | 49551675 | rs12588061 | C/T |
| chr14 | 49588772 | 3 | 1 | chr14 | 49588771 | rs8021027 | A/G |
| chr14 | 50068724 | 1 | 3 | chr14 | 50068722 | rs35245386 | A/C |
| chr14 | 50367706 | 8 | 2 | chr14 | 50367704 | rs9323195 | A/C |
| chr14 | 50565772 | 1 | 4 | chr14 | 50565770 | rs1556926 | A/G |
| chr14 | 50652949 | 1 | 4 | chr14 | 50652947 | rs2999375 | A/G |
| chr14 | 50988283 | 8 | 3 | chr14 | 50988282 | rs2356916 | A/G |
| chr14 | 51381133 | 4 | 1 | chr14 | 51381132 | rs2748137 | A/G |
| chr14 | 51645263 | 4 | 1 | chr14 | 51645261 | rs10151177 | C/T |
| chr14 | 51654107 | 0 | 0 | chr14 | 51654106 | rs4901200 | A/G |
| chr14 | 52693645 | 1 | 4 | chr14 | 52693643 | rs972171 | A/G |
| chr14 | 52866358 | 5 | 8 | chr14 | 52866357 | rs1255335 | C/T |
| chr14 | 53539814 | 2 | 2 | chr14 | 53539813 | rs12888986 | A/G |

| | | | | | | | |
|---|---|---|---|---|---|---|---|
| chr14 | 54909554 | 3 | 2 | chr14 | 54909553 | rs7150763 | A/G |
| chr14 | 55052017 | 0 | 0 | chr14 | 55052016 | rs2147118 | A/G |
| chr14 | 56255159 | 3 | 4 | chr14 | 56255158 | rs198258 | A/G |
| chr14 | 56406642 | 3 | 1 | chr14 | 56406640 | rs1604890 | A/G |
| chr14 | 57002211 | 5 | 15 | chr14 | 57002209 | rs1152536 | C/T |
| chr14 | 57823655 | 1 | 3 | chr14 | 57823653 | rs10137327 | C/T |
| chr14 | 57854535 | 2 | 3 | chr14 | 57854533 | rs6573199 | C/T |
| chr14 | 58368812 | 2 | 3 | chr14 | 58368811 | rs409518 | C/T |
| chr14 | 58515988 | 3 | 1 | chr14 | 58515987 | rs7148715 | A/G |
| chr14 | 58680213 | 3 | 1 | chr14 | 58680212 | rs10498489 | G/T |
| chr14 | 59113175 | 3 | 1 | chr14 | 59113174 | rs4898996 | A/G |
| chr14 | 60174491 | 2 | 3 | chr14 | 60174490 | rs8014133 | A/C/G |
| chr14 | 61029693 | 1 | 4 | chr14 | 61029691 | rs4444237 | C/T |
| chr14 | 61794590 | 8 | 1 | chr14 | 61794589 | rs12588013 | G/T |
| chr14 | 62249347 | 3 | 2 | chr14 | 62249345 | rs2171877 | C/T |
| chr14 | 62850477 | 4 | 1 | chr14 | 62850475 | rs728526 | C/T |
| chr14 | 64047945 | 1 | 3 | chr14 | 64047944 | rs74056445 | A/G |
| chr14 | 64312033 | 3 | 3 | chr14 | 64312031 | rs2012515 | C/G |
| chr14 | 64745706 | 6 | 4 | chr14 | 64745705 | rs6573593 | A/G |
| chr14 | 65408258 | 2 | 2 | chr14 | 65408256 | rs7153259 | A/C |
| chr14 | 65540118 | 4 | 18 | chr14 | 65540117 | rs1147440 | A/G |
| chr14 | 65585913 | 4 | 1 | chr14 | 65585912 | rs57036551 | A/G |
| chr14 | 67820209 | 3 | 2 | chr14 | 67820208 | rs2331666 | A/G |
| chr14 | 68140517 | 1 | 8 | chr14 | 68140516 | rs2236185 | C/T |
| chr14 | 68357237 | 1 | 9 | chr14 | 68357235 | rs194742 | C/T |
| chr14 | 68475207 | 6 | 1 | chr14 | 68475205 | rs1009145 | A/G |
| chr14 | 68747069 | 1 | 5 | chr14 | 68747067 | rs4902701 | C/G |
| chr14 | 69912508 | 3 | 6 | chr14 | 69912507 | rs2275297 | G/T |
| chr14 | 70198217 | 2 | 2 | chr14 | 70198214 | rs1205239 | C/G |
| chr14 | 70717326 | 3 | 1 | chr14 | 70717324 | rs56027927 | C/T |
| chr14 | 70717326 | 3 | 1 | chr14 | 70717325 | rs7144329 | A/G |
| chr14 | 71878074 | 1 | 3 | chr14 | 71878073 | rs8022859 | A/G |

| | | | | | | | |
|---|---|---|---|---|---|---|---|
| chr14 | 72025138 | 3 | 3 | chr14 | 72025136 | rs12588021 | C/T |
| chr14 | 72975700 | 3 | 2 | chr14 | 72975699 | rs4903111 | A/G |
| chr14 | 73388538 | 3 | 1 | chr14 | 73388538 | rs8020523 | C/G |
| chr14 | 73823032 | 10 | 1 | chr14 | 73823030 | rs929449 | A/G |
| chr14 | 74088911 | 3 | 1 | chr14 | 74088909 | rs11622992 | C/T |
| chr14 | 74514302 | 2 | 3 | chr14 | 74514300 | rs175004 | C/T |
| chr14 | 74521572 | 4 | 7 | chr14 | 74521570 | rs175010 | A/G |
| chr14 | 74931957 | 3 | 2 | chr14 | 74931955 | rs8011493 | C/T |
| chr14 | 75577587 | 4 | 2 | chr14 | 75577586 | rs721979 | C/T |
| chr14 | 75827931 | 1 | 6 | chr14 | 75827930 | rs1107598 | C/T |
| chr14 | 75844666 | 6 | 2 | chr14 | 75844664 | rs12887790 | C/T |
| chr14 | 75858502 | 2 | 5 | chr14 | 75858501 | rs8012684 | A/G |
| chr14 | 75860516 | 3 | 1 | chr14 | 75860514 | rs72729693 | C/T |
| chr14 | 76083376 | 5 | 3 | chr14 | 76083374 | rs1621109 | C/T |
| chr14 | 76418944 | 3 | 1 | chr14 | 76418942 | rs8007899 | C/T |
| chr14 | 76494825 | 4 | 3 | chr14 | 76494822 | rs759594 | C/G |
| chr14 | 76700039 | 1 | 5 | chr14 | 76700038 | rs158104 | G/T |
| chr14 | 76880467 | 3 | 1 | chr14 | 76880465 | rs11159255 | C/G |
| chr14 | 77405860 | 2 | 4 | chr14 | 77405859 | rs4903662 | A/G |
| chr14 | 77440208 | 2 | 6 | chr14 | 77440207 | rs12590199 | A/G |
| chr14 | 77676577 | 1 | 4 | chr14 | 77676576 | rs888412 | A/G |
| chr14 | 78047934 | 2 | 2 | chr14 | 78047933 | rs6574451 | A/G |
| chr14 | 79383064 | 1 | 3 | chr14 | 79383062 | rs2049826 | A/C |
| chr14 | 80954610 | 6 | 3 | chr14 | 80954609 | rs8004137 | A/G |
| chr14 | 83522212 | 2 | 3 | chr14 | 83522211 | rs1756294 | C/T |
| chr14 | 83783148 | 2 | 2 | chr14 | 83783146 | rs55934425 | C/T |
| chr14 | 83941418 | 7 | 4 | chr14 | 83941417 | rs4365225 | A/G |
| chr14 | 83969176 | 1 | 3 | chr14 | 83969174 | rs61985646 | C/T |
| chr14 | 84380082 | 1 | 4 | chr14 | 84380081 | rs12147229 | A/G |
| chr14 | 84779193 | 1 | 3 | chr14 | 84779191 | rs71418985 | C/T |
| chr14 | 85605269 | 8 | 1 | chr14 | 85605267 | rs7150201 | A/C |
| chr14 | 85796097 | 4 | 1 | chr14 | 85796094 | rs2135102 | C/T |

| | | | | | | | |
|---|---|---|---|---|---|---|---|
| chr14 | 85804940 | 2 | 2 | chr14 | 85804938 | rs1542941 | A/G |
| chr14 | 85900220 | 1 | 3 | chr14 | 85900218 | rs11847558 | C/T |
| chr14 | 86626160 | 4 | 2 | chr14 | 86626158 | rs4541001 | A/C |
| chr14 | 86626160 | 4 | 2 | chr14 | 86626160 | rs35032724 | A/G |
| chr14 | 87645895 | 7 | 2 | chr14 | 87645894 | rs4301957 | G/T |
| chr14 | 88443063 | 1 | 6 | chr14 | 88443061 | rs4904471 | C/T |
| chr14 | 88467934 | 3 | 3 | chr14 | 88467933 | rs743232 | A/G |
| chr14 | 89890273 | 3 | 8 | chr14 | 89890271 | rs12883844 | C/T |
| chr14 | 90218631 | 7 | 1 | chr14 | 90218629 | rs12147413 | A/C |
| chr14 | 90953022 | 2 | 3 | chr14 | 90953019 | rs1285844 | A/C |
| chr14 | 90960431 | 3 | 2 | chr14 | 90960429 | rs11160006 | C/T |
| chr14 | 91232295 | 9 | 1 | chr14 | 91232293 | rs61988226 | C/T |
| chr14 | 91743464 | 6 | 7 | chr14 | 91743462 | rs1241719 | C/T |
| chr14 | 91749701 | 2 | 2 | chr14 | 91749701 | rs1241738 | A/G |
| chr14 | 91799899 | 10 | 2 | chr14 | 91799898 | rs4904856 | A/G |
| chr14 | 91989195 | 0 | 0 | chr14 | 91989195 | rs7149167 | A/G |
| chr14 | 91989195 | 0 | 0 | chr14 | 91989195 | rs71412341 | GGC/TGT |
| chr14 | 92052870 | 1 | 4 | chr14 | 92052869 | rs7154465 | G/T |
| chr14 | 92185765 | 2 | 2 | chr14 | 92185763 | rs942056 | A/G |
| chr14 | 92185765 | 2 | 2 | chr14 | 92185765 | rs61992599 | A/G |
| chr14 | 93370310 | 3 | 1 | chr14 | 93370308 | rs4900197 | C/T |
| chr14 | 93441883 | 1 | 5 | chr14 | 93441882 | rs3748333 | C/T |
| chr14 | 93457738 | 1 | 3 | chr14 | 93457737 | rs11627710 | A/G |
| chr14 | 93464079 | 1 | 3 | chr14 | 93464078 | rs28515866 | A/G |
| chr14 | 93613413 | 1 | 3 | chr14 | 93613411 | rs11626017 | C/T |
| chr14 | 93676324 | 2 | 2 | chr14 | 93676322 | rs8015878 | C/T |
| chr14 | 94550677 | 1 | 5 | chr14 | 94550676 | rs7147709 | A/G |
| chr14 | 94615341 | 2 | 2 | chr14 | 94615339 | rs1187642 | C/T |
| chr14 | 94919583 | 10 | 3 | chr14 | 94919581 | rs1187727 | A/G |
| chr14 | 94922019 | 1 | 3 | chr14 | 94922017 | rs10140398 | C/T |
| chr14 | 95105665 | 1 | 3 | chr14 | 95105663 | rs1570265 | C/T |
| chr14 | 95142357 | 9 | 6 | chr14 | 95142356 | rs7149067 | A/G |

| | | | | | | | | |
|---|---|---|---|---|---|---|---|---|
| chr14 | 95147448 | 1 | 4 | chr14 | 95147446 | rs11624701 | | C/T |
| chr14 | 95216210 | 2 | 8 | chr14 | 95216208 | rs1743513 | | C/G |
| chr14 | 95626875 | 3 | 6 | chr14 | 95626873 | rs10148419 | | C/T |
| chr14 | 96174903 | 2 | 2 | chr14 | 96174902 | rs234569 | | C/T |
| chr14 | 96220556 | 2 | 4 | chr14 | 96220554 | rs17244398 | | C/T |
| chr14 | 96468072 | 3 | 2 | chr14 | 96468070 | rs856224 | | C/T |
| chr14 | 96472582 | 13 | 3 | chr14 | 96472581 | rs746127 | | A/G |
| chr14 | 96592007 | 6 | 1 | chr14 | 96592006 | rs1683147 | | A/G |
| chr14 | 96739967 | 2 | 2 | chr14 | 96739965 | rs4146513 | | A/G |
| chr14 | 96761695 | 1 | 5 | chr14 | 96761694 | rs10150389 | | A/G |
| chr14 | 97336302 | 3 | 1 | chr14 | 97336300 | rs10129347 | | A/C |
| chr14 | 97701037 | 4 | 3 | chr14 | 97701036 | rs8007838 | | A/G |
| chr14 | 97707232 | 6 | 1 | chr14 | 97707231 | rs7160630 | | A/G |
| chr14 | 98123717 | 3 | 2 | chr14 | 98123716 | rs4905720 | | A/G |
| chr14 | 98663552 | 1 | 6 | chr14 | 98663551 | rs1257516 | | A/G |
| chr14 | 99004672 | 1 | 3 | chr14 | 99004671 | rs8021023 | | A/G |
| chr14 | 99065863 | 4 | 9 | chr14 | 99065861 | rs6575729 | | C/T |
| chr14 | 99170440 | 1 | 3 | chr14 | 99170439 | rs941549 | | C/G |
| chr14 | 99679641 | 1 | 3 | chr14 | 99679640 | rs11621814 | | A/G |
| chr14 | 99834141 | 10 | 7 | chr14 | 99834139 | rs6575768 | | C/T |
| chr14 | 100068154 | 5 | 3 | chr14 | 100068153 | rs8008247 | | A/G |
| chr14 | 100647838 | 1 | 6 | chr14 | 100647837 | rs4906046 | | A/G |
| chr14 | 100661699 | 3 | 4 | chr14 | 100661698 | rs11628167 | | A/G |
| chr14 | 100772134 | 4 | 1 | chr14 | 100772133 | rs8006867 | | A/G |
| chr14 | 100794572 | 1 | 3 | chr14 | 100794571 | rs2210801 | | C/T |
| chr14 | 100875509 | 4 | 2 | chr14 | 100875507 | rs10141103 | | C/T |
| chr14 | 101469047 | 1 | 3 | chr14 | 101469046 | rs3993391 | | A/G |
| chr14 | 101981660 | 2 | 6 | chr14 | 101981659 | rs1190552 | | A/G |
| chr14 | 102631415 | 4 | 1 | chr14 | 102631413 | rs11621573 | | C/T |
| chr14 | 102646168 | 1 | 5 | chr14 | 102646167 | rs729184 | | C/T |
| chr14 | 102838034 | 19 | 1 | chr14 | 102838032 | rs4906311 | | C/T |
| chr14 | 103062813 | 16 | 8 | chr14 | 103062812 | rs2765040 | | C/T |

| | | | | | | | |
|---|---|---|---|---|---|---|---|
| chr14 | 103430730 | 3 | 1 | chr14 | 103430729 | rs10144862 | G/T |
| chr14 | 103464589 | 2 | 7 | chr14 | 103464587 | rs9324065 | C/T |
| chr14 | 103677821 | 5 | 3 | chr14 | 103677820 | rs2011069 | C/T |
| chr14 | 103689648 | 2 | 4 | chr14 | 103689646 | rs12892623 | C/T |
| chr14 | 103695512 | 0 | 0 | chr14 | 103695511 | rs2153557 | C/G |
| chr14 | 103708194 | 1 | 4 | chr14 | 103708193 | rs947001 | C/T |
| chr14 | 103729284 | 6 | 9 | chr14 | 103729282 | rs2487308 | A/G |
| chr14 | 103748113 | 1 | 3 | chr14 | 103748111 | rs10134193 | C/T |
| chr14 | 103799452 | 4 | 2 | chr14 | 103799451 | rs8021989 | A/G |
| chr14 | 104136497 | 3 | 7 | chr14 | 104136496 | rs8017525 | A/G |
| chr14 | 104163349 | 0 | 0 | chr14 | 104163347 | rs11627935 | C/T |
| chr14 | 104251753 | 3 | 1 | chr14 | 104251750 | rs1128840 | A/C/G |
| chr14 | 104430350 | 0 | 0 | chr14 | 104430349 | rs3742938 | A/G |
| chr14 | 104430350 | 0 | 0 | chr14 | 104430350 | rs3742939 | C/G |
| chr14 | 104544743 | 2 | 6 | chr14 | 104544740 | rs7141972 | C/G |
| chr14 | 104579621 | 4 | 4 | chr14 | 104579621 | rs4633639 | A/G |
| chr14 | 104580287 | 2 | 2 | chr14 | 104580286 | rs12436313 | A/G |
| chr14 | 104603048 | 3 | 2 | chr14 | 104603046 | rs3809469 | A/G |
| chr14 | 104633810 | 3 | 6 | chr14 | 104633809 | rs12885324 | A/G |
| chr14 | 104645889 | 4 | 1 | chr14 | 104645887 | rs9324081 | C/T |
| chr14 | 104679448 | 3 | 5 | chr14 | 104679447 | rs760269 | C/T |
| chr14 | 104719947 | 10 | 3 | chr14 | 104719945 | rs11160838 | C/T |
| chr14 | 104758817 | 8 | 4 | chr14 | 104758815 | rs17620990 | C/T |
| chr14 | 104963231 | 0 | 0 | chr14 | 104963230 | rs4983616 | C/G |
| chr14 | 104990471 | 0 | 0 | chr14 | 104990469 | rs6576104 | C/T |
| chr14 | 105243542 | 4 | 1 | chr14 | 105243540 | rs71423284 | C/T |
| chr14 | 105415697 | 2 | 8 | chr14 | 105415696 | rs2753518 | A/G |
| chr14 | 105428021 | 1 | 10 | chr14 | 105428018 | rs2753529 | C/G |
| chr14 | 105998074 | 1 | 5 | chr14 | 105998073 | rs10150684 | A/G |
| chr14 | 106073188 | 10 | 1 | chr14 | 106073186 | rs11160987 | C/T |
| chr14 | 106087129 | 2 | 3 | chr14 | 106087128 | rs17112924 | A/G |
| chr15 | 18873428 | 6 | 4 | chr15 | 18873428 | rs1972036 | A/C |

| | | | | | | | |
|---|---|---|---|---|---|---|---|
| chr15 | 20509545 | 3 | 2 | chr15 | 20509544 | rs4134803 | A/G |
| chr15 | 20597847 | 2 | 2 | chr15 | 20597844 | rs12902722 | C/T |
| chr15 | 23182234 | 1 | 3 | chr15 | 23182233 | rs4488416 | A/G |
| chr15 | 23563637 | 4 | 1 | chr15 | 23563636 | rs1444623 | A/G |
| chr15 | 23594335 | 4 | 5 | chr15 | 23594333 | rs2873451 | A/G |
| chr15 | 23661566 | 7 | 14 | chr15 | 23661564 | rs1444621 | C/T |
| chr15 | 23661566 | 7 | 14 | chr15 | 23661565 | rs2076747 | C/T |
| chr15 | 23706254 | 3 | 1 | chr15 | 23706254 | rs12914791 | C/G |
| chr15 | 24894466 | 1 | 5 | chr15 | 24894465 | rs17562513 | A/G |
| chr15 | 25375486 | 3 | 2 | chr15 | 25375484 | rs4432228 | C/T |
| chr15 | 25473492 | 2 | 2 | chr15 | 25473490 | rs28399194 | A/C |
| chr15 | 25725654 | 0 | 0 | chr15 | 25725653 | rs12916836 | A/G |
| chr15 | 27320089 | 8 | 1 | chr15 | 27320088 | rs28688063 | A/G |
| chr15 | 27440582 | 1 | 6 | chr15 | 27440580 | rs1520941 | C/T |
| chr15 | 29102862 | 3 | 2 | chr15 | 29102861 | rs2911855 | C/T |
| chr15 | 29378250 | 1 | 3 | chr15 | 29378250 | rs4238554 | C/G |
| chr15 | 29405082 | 5 | 4 | chr15 | 29405079 | rs6493604 | A/C |
| chr15 | 30004980 | 3 | 1 | chr15 | 30004978 | rs953326 | C/T |
| chr15 | 30171497 | 3 | 1 | chr15 | 30171495 | rs2133966 | A/G |
| chr15 | 32514252 | 7 | 2 | chr15 | 32514250 | rs1874377 | A/G |
| chr15 | 32603809 | 2 | 2 | chr15 | 32603806 | rs16973574 | C/T |
| chr15 | 32661379 | 1 | 4 | chr15 | 32661378 | rs36155480 | C/T |
| chr15 | 34026847 | 2 | 3 | chr15 | 34026845 | rs1510377 | C/T |
| chr15 | 34871174 | 3 | 1 | chr15 | 34871173 | rs12899256 | A/G |
| chr15 | 35348561 | 7 | 5 | chr15 | 35348559 | rs2222299 | A/G |
| chr15 | 36423202 | 1 | 6 | chr15 | 36423201 | rs3942501 | A/G |
| chr15 | 37073830 | 8 | 3 | chr15 | 37073829 | rs1368806 | A/C |
| chr15 | 37433118 | 9 | 13 | chr15 | 37433117 | rs8030000 | A/G |
| chr15 | 37779164 | 3 | 1 | chr15 | 37779163 | rs2631696 | A/G |
| chr15 | 38350994 | 1 | 3 | chr15 | 38350992 | rs6492940 | C/T |
| chr15 | 38891155 | 4 | 2 | chr15 | 38891153 | rs2412567 | C/T |
| chr15 | 38936455 | 2 | 4 | chr15 | 38936452 | rs690458 | C/G |

| | | | | | | | |
|---|---|---|---|---|---|---|---|
| chr15 | 39934005 | 3 | 5 | chr15 | 39934003 | rs890506 | A/G |
| chr15 | 40804717 | 2 | 4 | chr15 | 40804717 | rs16957091 | G/T |
| chr15 | 41472366 | 1 | 3 | chr15 | 41472365 | rs35158584 | G/T |
| chr15 | 41543502 | 3 | 1 | chr15 | 41543501 | rs694725 | A/G |
| chr15 | 42815643 | 1 | 6 | chr15 | 42815642 | rs935889 | G/T |
| chr15 | 43304586 | 2 | 4 | chr15 | 43304584 | rs2668751 | C/T |
| chr15 | 44406512 | 1 | 4 | chr15 | 44406511 | rs7171202 | A/G |
| chr15 | 44429400 | 2 | 9 | chr15 | 44429398 | rs17574125 | C/T |
| chr15 | 45042736 | 3 | 1 | chr15 | 45042734 | rs1512210 | A/G |
| chr15 | 45125650 | 1 | 6 | chr15 | 45125648 | rs7165757 | C/T |
| chr15 | 45362346 | 5 | 15 | chr15 | 45362345 | rs2045158 | C/T |
| chr15 | 45430545 | 3 | 1 | chr15 | 45430544 | rs531595 | A/G |
| chr15 | 45661671 | 3 | 2 | chr15 | 45661670 | rs2413878 | A/G |
| chr15 | 46108374 | 3 | 1 | chr15 | 46108372 | rs1377686 | C/T |
| chr15 | 46743560 | 6 | 1 | chr15 | 46743558 | rs8038489 | C/T |
| chr15 | 47065618 | 3 | 10 | chr15 | 47065617 | rs4775791 | A/G |
| chr15 | 47145327 | 3 | 2 | chr15 | 47145324 | rs17393601 | A/C |
| chr15 | 49063420 | 3 | 3 | chr15 | 49063419 | rs2306335 | A/G |
| chr15 | 50228605 | 2 | 2 | chr15 | 50228602 | rs3794541 | C/T |
| chr15 | 50389042 | 1 | 4 | chr15 | 50389041 | rs2414136 | A/G |
| chr15 | 50870907 | 2 | 10 | chr15 | 50870905 | rs2440316 | A/G |
| chr15 | 51049196 | 3 | 4 | chr15 | 51049195 | rs13313444 | A/G |
| chr15 | 51294235 | 5 | 1 | chr15 | 51294233 | rs2661576 | C/T |
| chr15 | 52317289 | 5 | 2 | chr15 | 52317288 | rs7183952 | A/G |
| chr15 | 52455189 | 2 | 3 | chr15 | 52455188 | rs56090546 | G/T |
| chr15 | 52455324 | 3 | 1 | chr15 | 52455323 | rs56310458 | A/G |
| chr15 | 54851335 | 1 | 5 | chr15 | 54851334 | rs7167131 | C/G |
| chr15 | 55277582 | 4 | 1 | chr15 | 55277581 | rs2703595 | A/G |
| chr15 | 55595200 | 2 | 3 | chr15 | 55595199 | rs16977560 | G/T |
| chr15 | 56046890 | 5 | 5 | chr15 | 56046888 | rs2899609 | C/T |
| chr15 | 56077032 | 3 | 1 | chr15 | 56077030 | rs1441829 | A/G |
| chr15 | 56313174 | 4 | 2 | chr15 | 56313172 | rs1663252 | C/T |

| | | | | | | | |
|---|---|---|---|---|---|---|---|
| chr15 | 56474662 | 4 | 2 | chr15 | 56474661 | rs17240832 | A/G |
| chr15 | 56553439 | 5 | 6 | chr15 | 56553438 | rs1869136 | A/G |
| chr15 | 57303765 | 3 | 4 | chr15 | 57303764 | rs3794491 | C/T |
| chr15 | 57509480 | 1 | 7 | chr15 | 57509478 | rs59326013 | C/T |
| chr15 | 57737064 | 1 | 4 | chr15 | 57737061 | rs7163436 | A/C |
| chr15 | 57906822 | 3 | 1 | chr15 | 57906821 | rs7181522 | A/G |
| chr15 | 58292211 | 8 | 3 | chr15 | 58292210 | rs1680198 | C/T |
| chr15 | 58311239 | 1 | 3 | chr15 | 58311237 | rs7166014 | C/T |
| chr15 | 58919944 | 4 | 1 | chr15 | 58919943 | rs1224251 | A/G |
| chr15 | 59065515 | 5 | 4 | chr15 | 59065513 | rs12101478 | C/T |
| chr15 | 59296753 | 10 | 2 | chr15 | 59296751 | rs7175393 | C/T |
| chr15 | 59296861 | 11 | 4 | chr15 | 59296859 | rs17303572 | C/T |
| chr15 | 59658129 | 15 | 2 | chr15 | 59658127 | rs7497302 | C/T |
| chr15 | 60503934 | 4 | 1 | chr15 | 60503932 | rs12911081 | C/T |
| chr15 | 60538325 | 3 | 11 | chr15 | 60538324 | rs410732 | A/C |
| chr15 | 60671934 | 3 | 3 | chr15 | 60671933 | rs6494325 | A/G |
| chr15 | 61077332 | 2 | 5 | chr15 | 61077331 | rs11071708 | A/G |
| chr15 | 61943518 | 3 | 3 | chr15 | 61943517 | rs8028348 | A/G |
| chr15 | 62823032 | 2 | 2 | chr15 | 62823031 | rs3935716 | A/G |
| chr15 | 63463671 | 6 | 1 | chr15 | 63463669 | rs2292933 | C/T |
| chr15 | 63463671 | 6 | 1 | chr15 | 63463670 | rs61747070 | C/T |
| chr15 | 63609875 | 4 | 1 | chr15 | 63609875 | rs422390 | C/G |
| chr15 | 64058879 | 5 | 3 | chr15 | 64058877 | rs407596 | A/G |
| chr15 | 64139019 | 1 | 4 | chr15 | 64139018 | rs6494545 | A/G |
| chr15 | 64702650 | 7 | 4 | chr15 | 64702649 | rs2469098 | C/T |
| chr15 | 65001669 | 2 | 3 | chr15 | 65001669 | rs904365 | C/G |
| chr15 | 65344728 | 3 | 3 | chr15 | 65344726 | rs8032868 | C/T |
| chr15 | 66396888 | 3 | 1 | chr15 | 66396886 | rs1516871 | A/G |
| chr15 | 66411451 | 5 | 2 | chr15 | 66411449 | rs7168069 | A/C |
| chr15 | 66451906 | 4 | 9 | chr15 | 66451905 | rs12443350 | A/G |
| chr15 | 66880997 | 4 | 2 | chr15 | 66880996 | rs6494774 | A/G |
| chr15 | 66893158 | 3 | 5 | chr15 | 66893157 | rs11635552 | A/G |

| | | | | | | | |
|---|---|---|---|---|---|---|---|
| chr15 | 67228531 | 2 | 5 | chr15 | 67228530 | rs11634860 | A/G |
| chr15 | 67581206 | 3 | 4 | chr15 | 67581204 | rs8028075 | C/T |
| chr15 | 67674508 | 3 | 2 | chr15 | 67674507 | rs8036001 | A/G |
| chr15 | 67914660 | 2 | 4 | chr15 | 67914659 | rs304987 | A/G |
| chr15 | 68134357 | 8 | 3 | chr15 | 68134356 | rs661936 | C/T |
| chr15 | 68268275 | 1 | 3 | chr15 | 68268273 | rs61031118 | A/C |
| chr15 | 68268275 | 1 | 3 | chr15 | 68268273 | rs71457460 | AA/CG |
| chr15 | 68268275 | 1 | 3 | chr15 | 68268274 | rs61630692 | A/G |
| chr15 | 69199079 | 4 | 2 | chr15 | 69199078 | rs726225 | C/T |
| chr15 | 69679269 | 1 | 4 | chr15 | 69679268 | rs4398066 | A/G |
| chr15 | 69753656 | 2 | 5 | chr15 | 69753655 | rs28664089 | A/G |
| chr15 | 69885726 | 5 | 1 | chr15 | 69885725 | rs4776576 | A/G |
| chr15 | 70132359 | 4 | 3 | chr15 | 70132357 | rs59903101 | C/T |
| chr15 | 70132359 | 4 | 3 | chr15 | 70132358 | rs7172557 | G/T |
| chr15 | 70596726 | 7 | 3 | chr15 | 70596724 | rs12902245 | C/T |
| chr15 | 70667736 | 6 | 3 | chr15 | 70667735 | rs6495011 | A/G |
| chr15 | 70882722 | 6 | 3 | chr15 | 70882720 | rs12438864 | C/T |
| chr15 | 70892785 | 5 | 1 | chr15 | 70892784 | rs58232781 | A/G |
| chr15 | 71210075 | 2 | 2 | chr15 | 71210074 | rs12899246 | A/G |
| chr15 | 71793745 | 2 | 2 | chr15 | 71793743 | rs1044581 | C/T |
| chr15 | 71829551 | 6 | 1 | chr15 | 71829549 | rs751033 | C/T |
| chr15 | 71909278 | 4 | 2 | chr15 | 71909276 | rs4886421 | C/T |
| chr15 | 71945772 | 1 | 4 | chr15 | 71945771 | rs11629922 | A/G |
| chr15 | 72012721 | 2 | 5 | chr15 | 72012719 | rs8027093 | C/T |
| chr15 | 72270469 | 5 | 6 | chr15 | 72270467 | rs351228 | A/G |
| chr15 | 72394310 | 9 | 1 | chr15 | 72394309 | rs874712 | C/T |
| chr15 | 72404701 | 1 | 6 | chr15 | 72404701 | rs1484216 | A/G |
| chr15 | 72502259 | 3 | 1 | chr15 | 72502257 | rs11852686 | C/T |
| chr15 | 72868799 | 2 | 3 | chr15 | 72868797 | rs3784790 | C/G |
| chr15 | 72901376 | 11 | 2 | chr15 | 72901374 | rs11636952 | C/T |
| chr15 | 73194351 | 3 | 2 | chr15 | 73194350 | rs4334268 | A/G |
| chr15 | 73286673 | 2 | 2 | chr15 | 73286671 | rs11072531 | C/T |

| | | | | | | | |
|---|---|---|---|---|---|---|---|
| chr15 | 73658681 | 4 | 1 | chr15 | 73658680 | rs62027209 | A/G |
| chr15 | 73739013 | 6 | 1 | chr15 | 73739013 | rs4243036 | A/G |
| chr15 | 74539335 | 2 | 4 | chr15 | 74539334 | rs508756 | C/T |
| chr15 | 75090795 | 1 | 3 | chr15 | 75090793 | rs8030698 | C/T |
| chr15 | 75652062 | 4 | 9 | chr15 | 75652060 | rs2682922 | G/T |
| chr15 | 75821663 | 3 | 2 | chr15 | 75821661 | rs3935684 | C/T |
| chr15 | 75867115 | 3 | 2 | chr15 | 75867113 | rs7170191 | C/T |
| chr15 | 75884269 | 5 | 3 | chr15 | 75884267 | rs72730742 | A/C |
| chr15 | 75995778 | 3 | 1 | chr15 | 75995777 | rs11072700 | A/G |
| chr15 | 76117780 | 8 | 3 | chr15 | 76117779 | rs1837912 | A/G |
| chr15 | 76220291 | 2 | 5 | chr15 | 76220289 | rs8026337 | C/T |
| chr15 | 76354625 | 2 | 2 | chr15 | 76354624 | rs967819 | G/T |
| chr15 | 76420649 | 3 | 1 | chr15 | 76420649 | rs2139440 | A/C |
| chr15 | 76831385 | 4 | 3 | chr15 | 76831384 | rs11632672 | A/G |
| chr15 | 76840069 | 3 | 1 | chr15 | 76840068 | rs2002854 | A/G |
| chr15 | 76910393 | 2 | 2 | chr15 | 76910392 | rs7165042 | C/G |
| chr15 | 77166395 | 3 | 1 | chr15 | 77166394 | rs6495369 | A/G |
| chr15 | 77498310 | 2 | 2 | chr15 | 77498309 | rs769766 | C/T |
| chr15 | 77574890 | 3 | 1 | chr15 | 77574889 | rs4778700 | A/G |
| chr15 | 77608339 | 2 | 3 | chr15 | 77608338 | rs11858937 | A/G |
| chr15 | 77997906 | 3 | 4 | chr15 | 77997905 | rs4778734 | A/G |
| chr15 | 79841607 | 2 | 3 | chr15 | 79841606 | rs12912239 | A/G |
| chr15 | 79993244 | 1 | 3 | chr15 | 79993242 | rs6495631 | C/T |
| chr15 | 81340760 | 8 | 6 | chr15 | 81340759 | rs1256452 | C/T |
| chr15 | 81368697 | 11 | 5 | chr15 | 81368696 | rs750234 | A/G |
| chr15 | 81379899 | 5 | 8 | chr15 | 81379898 | rs12148275 | A/G |
| chr15 | 84050477 | 7 | 1 | chr15 | 84050475 | rs338525 | A/G |
| chr15 | 84323781 | 1 | 4 | chr15 | 84323779 | rs6496293 | A/C |
| chr15 | 86454625 | 4 | 1 | chr15 | 86454624 | rs3825883 | C/T |
| chr15 | 88569964 | 5 | 2 | chr15 | 88569962 | rs908044 | G/T |
| chr15 | 89166174 | 2 | 2 | chr15 | 89166172 | rs372088 | A/G |
| chr15 | 89741045 | 1 | 4 | chr15 | 89741044 | rs4356456 | A/G |

| | | | | | | | |
|---|---|---|---|---|---|---|---|
| chr15 | 90056896 | 3 | 6 | chr15 | 90056894 | rs4486862 | C/T |
| chr15 | 90210101 | 4 | 2 | chr15 | 90210100 | rs7166146 | A/G |
| chr15 | 90368255 | 2 | 17 | chr15 | 90368253 | rs28700927 | C/T |
| chr15 | 90440443 | 1 | 4 | chr15 | 90440441 | rs207973 | G/T |
| chr15 | 90676395 | 2 | 3 | chr15 | 90676394 | rs936928 | C/T |
| chr15 | 90787920 | 1 | 4 | chr15 | 90787919 | rs3784732 | C/T |
| chr15 | 90996948 | 9 | 1 | chr15 | 90996946 | rs2438193 | C/T |
| chr15 | 91248635 | 5 | 1 | chr15 | 91248634 | rs1439619 | A/C |
| chr15 | 91763921 | 2 | 5 | chr15 | 91763920 | rs1532093 | A/G |
| chr15 | 91799100 | 2 | 3 | chr15 | 91799099 | rs988108 | C/G |
| chr15 | 92285902 | 4 | 1 | chr15 | 92285900 | rs7403002 | C/T |
| chr15 | 92356647 | 4 | 1 | chr15 | 92356646 | rs7173640 | A/G |
| chr15 | 93933123 | 2 | 2 | chr15 | 93933122 | rs4575483 | A/G |
| chr15 | 95731533 | 1 | 3 | chr15 | 95731531 | rs11636002 | C/T |
| chr15 | 96508052 | 1 | 5 | chr15 | 96508050 | rs922582 | C/G |
| chr15 | 96568740 | 2 | 13 | chr15 | 96568738 | rs2620862 | C/T |
| chr15 | 96924745 | 2 | 2 | chr15 | 96924744 | rs11639026 | A/G |
| chr15 | 97421273 | 1 | 3 | chr15 | 97421272 | rs2167737 | C/T |
| chr15 | 98054552 | 6 | 1 | chr15 | 98054552 | rs325388 | C/G |
| chr15 | 98414814 | 5 | 1 | chr15 | 98414813 | rs8038037 | G/T |
| chr15 | 98607401 | 2 | 2 | chr15 | 98607400 | rs4128195 | A/G |
| chr15 | 99214347 | 3 | 2 | chr15 | 99214346 | rs6598394 | A/G |
| chr15 | 99257343 | 1 | 5 | chr15 | 99257341 | rs56063524 | A/C |
| chr15 | 99265280 | 4 | 1 | chr15 | 99265279 | rs4646685 | G/T |
| chr15 | 99275538 | 3 | 2 | chr15 | 99275536 | rs4646693 | C/T |
| chr15 | 99276699 | 1 | 4 | chr15 | 99276699 | rs62019449 | C/G |
| chr15 | 99399370 | 7 | 7 | chr15 | 99399369 | rs6598423 | A/G |
| chr15 | 99471836 | 1 | 4 | chr15 | 99471835 | rs11633319 | C/G |
| chr15 | 99525862 | 9 | 14 | chr15 | 99525862 | rs1982489 | A/G |
| chr15 | 99832314 | 3 | 1 | chr15 | 99832312 | rs6598483 | C/T |
| chr15 | 99832314 | 3 | 1 | chr15 | 99832313 | rs55722037 | A/G |
| chr15 | 99960604 | 4 | 2 | chr15 | 99960602 | rs7175583 | C/T |

| | | | | | | | |
|---|---|---|---|---|---|---|---|
| chr16 | 64473 | 4 | 6 | chr16 | 64471 | rs2562155 | C/T |
| chr16 | 523532 | 1 | 5 | chr16 | 523530 | rs11864059 | C/T |
| chr16 | 533854 | 3 | 6 | chr16 | 533853 | rs7197307 | G/T |
| chr16 | 554821 | 6 | 4 | chr16 | 554821 | rs1010761 | A/G |
| chr16 | 815052 | 3 | 3 | chr16 | 815050 | rs593569 | A/G |
| chr16 | 898507 | 3 | 2 | chr16 | 898506 | rs4984974 | A/G |
| chr16 | 927360 | 4 | 1 | chr16 | 927358 | rs5015439 | C/T |
| chr16 | 927360 | 4 | 1 | chr16 | 927359 | rs5015438 | C/G |
| chr16 | 1148871 | 3 | 2 | chr16 | 1148869 | rs1609697 | A/G |
| chr16 | 1253383 | 6 | 6 | chr16 | 1253382 | rs6600126 | A/G |
| chr16 | 1304769 | 3 | 2 | chr16 | 1304767 | rs2281226 | A/C |
| chr16 | 1394084 | 6 | 3 | chr16 | 1394082 | rs909922 | C/T |
| chr16 | 1394173 | 6 | 1 | chr16 | 1394171 | rs909923 | C/T |
| chr16 | 1454653 | 1 | 4 | chr16 | 1454652 | rs2744996 | A/G |
| chr16 | 1464851 | 2 | 5 | chr16 | 1464850 | rs3751884 | A/G |
| chr16 | 1764076 | 1 | 10 | chr16 | 1764075 | rs1033476 | A/G |
| chr16 | 1777902 | 3 | 2 | chr16 | 1777900 | rs344358 | C/T |
| chr16 | 1778642 | 1 | 5 | chr16 | 1778640 | rs344359 | C/T |
| chr16 | 1779379 | 5 | 5 | chr16 | 1779377 | rs182939 | C/T |
| chr16 | 1784782 | 1 | 5 | chr16 | 1784781 | rs2256923 | C/T |
| chr16 | 1821558 | 2 | 2 | chr16 | 1821556 | rs34392705 | C/T |
| chr16 | 1836025 | 8 | 1 | chr16 | 1836024 | rs62038434 | A/G |
| chr16 | 1888871 | 2 | 6 | chr16 | 1888870 | rs56077812 | G/T |
| chr16 | 2023156 | 5 | 2 | chr16 | 2023155 | rs28373896 | A/G |
| chr16 | 2778002 | 0 | 0 | chr16 | 2778000 | rs12599113 | C/T |
| chr16 | 2872494 | 3 | 3 | chr16 | 2872492 | rs6500687 | C/G |
| chr16 | 2902131 | 3 | 4 | chr16 | 2902129 | rs11647026 | C/G |
| chr16 | 3036079 | 1 | 3 | chr16 | 3036077 | rs3760079 | C/G |
| chr16 | 3071567 | 0 | 0 | chr16 | 3071566 | rs2741927 | G/T |
| chr16 | 3076921 | 6 | 2 | chr16 | 3076919 | rs4786384 | C/T |
| chr16 | 3968061 | 2 | 6 | chr16 | 3968060 | rs2601775 | A/G |
| chr16 | 3968146 | 0 | 0 | chr16 | 3968145 | rs2531989 | C/T |

| | | | | | | | |
|---|---|---|---|---|---|---|---|
| chr16 | 4043873 | 2 | 3 | chr16 | 4043871 | rs2601828 | A/G |
| chr16 | 4075760 | 3 | 4 | chr16 | 4075758 | rs72764609 | C/T |
| chr16 | 4116365 | 2 | 3 | chr16 | 4116364 | rs397666 | A/C |
| chr16 | 4318179 | 6 | 6 | chr16 | 4318178 | rs2906899 | A/G |
| chr16 | 4469614 | 4 | 5 | chr16 | 4469613 | rs6500610 | A/G |
| chr16 | 4539861 | 3 | 1 | chr16 | 4539861 | rs9932498 | A/G |
| chr16 | 4674592 | 8 | 2 | chr16 | 4674590 | rs841226 | C/T |
| chr16 | 4786070 | 3 | 2 | chr16 | 4786068 | rs2075465 | C/T |
| chr16 | 4874564 | 1 | 9 | chr16 | 4874564 | rs1049207 | C/G |
| chr16 | 5018843 | 8 | 1 | chr16 | 5018841 | rs1995278 | A/G |
| chr16 | 5152290 | 2 | 9 | chr16 | 5152289 | rs4786633 | A/G |
| chr16 | 5312482 | 4 | 2 | chr16 | 5312481 | rs2049694 | A/G |
| chr16 | 5480706 | 4 | 1 | chr16 | 5480703 | rs8059274 | C/G |
| chr16 | 5606256 | 2 | 5 | chr16 | 5606254 | rs73527617 | C/T |
| chr16 | 5931704 | 5 | 3 | chr16 | 5931702 | rs11644345 | A/C |
| chr16 | 6472746 | 2 | 2 | chr16 | 6472744 | rs58775805 | C/G |
| chr16 | 6892126 | 3 | 1 | chr16 | 6892124 | rs4786949 | A/C/T |
| chr16 | 6938238 | 6 | 2 | chr16 | 6938237 | rs7200577 | A/G |
| chr16 | 7300134 | 5 | 3 | chr16 | 7300132 | rs4479249 | C/T |
| chr16 | 7578522 | 3 | 1 | chr16 | 7578519 | rs7195207 | A/C |
| chr16 | 7929339 | 5 | 1 | chr16 | 7929338 | rs4074917 | C/T |
| chr16 | 8391797 | 3 | 3 | chr16 | 8391795 | rs34112454 | C/T |
| chr16 | 8632284 | 3 | 6 | chr16 | 8632282 | rs8061405 | A/C |
| chr16 | 8645898 | 6 | 2 | chr16 | 8645898 | rs1641065 | A/G |
| chr16 | 8755256 | 3 | 3 | chr16 | 8755254 | rs1641016 | C/T |
| chr16 | 8887563 | 3 | 1 | chr16 | 8887561 | rs30777 | C/T |
| chr16 | 8887911 | 8 | 10 | chr16 | 8887910 | rs39865 | G/T |
| chr16 | 8945619 | 6 | 1 | chr16 | 8945617 | rs12449090 | C/T |
| chr16 | 9009110 | 1 | 4 | chr16 | 9009109 | rs4985018 | A/G |
| chr16 | 9128948 | 1 | 3 | chr16 | 9128947 | rs17567434 | C/G |
| chr16 | 9744226 | 5 | 1 | chr16 | 9744225 | rs4371155 | A/G |
| chr16 | 10118186 | 2 | 5 | chr16 | 10118184 | rs13337372 | C/T |

| | | | | | | | |
|---|---|---|---|---|---|---|---|
| chr16 | 10191482 | 2 | 2 | chr16 | 10191481 | rs12930589 | A/G |
| chr16 | 10260240 | 4 | 2 | chr16 | 10260239 | rs2162695 | C/T |
| chr16 | 10862957 | 4 | 1 | chr16 | 10862956 | rs7501308 | A/G |
| chr16 | 11155279 | 3 | 1 | chr16 | 11155277 | rs27867 | C/T |
| chr16 | 11155279 | 3 | 1 | chr16 | 11155279 | rs7203629 | A/G |
| chr16 | 11408243 | 2 | 7 | chr16 | 11408241 | rs4781089 | C/T |
| chr16 | 11575956 | 1 | 3 | chr16 | 11575954 | rs12708730 | C/T |
| chr16 | 12259499 | 5 | 1 | chr16 | 12259498 | rs2904423 | A/G |
| chr16 | 12631386 | 3 | 3 | chr16 | 12631385 | rs11075139 | A/G |
| chr16 | 12900351 | 1 | 4 | chr16 | 12900349 | rs2014726 | A/G |
| chr16 | 13019113 | 3 | 1 | chr16 | 13019111 | rs7197910 | C/T |
| chr16 | 13473075 | 5 | 1 | chr16 | 13473074 | rs2078548 | A/G |
| chr16 | 13672564 | 9 | 1 | chr16 | 13672563 | rs7200727 | G/T |
| chr16 | 13778578 | 2 | 4 | chr16 | 13778576 | rs6498470 | C/T |
| chr16 | 14015219 | 3 | 1 | chr16 | 14015217 | rs1651188 | G/T |
| chr16 | 14309839 | 7 | 1 | chr16 | 14309837 | rs30230 | C/T |
| chr16 | 15045949 | 1 | 6 | chr16 | 15045947 | rs3803575 | C/T |
| chr16 | 15787262 | 2 | 2 | chr16 | 15787260 | rs9929522 | C/T |
| chr16 | 15944762 | 2 | 3 | chr16 | 15944761 | rs8050881 | A/G |
| chr16 | 15995067 | 9 | 3 | chr16 | 15995065 | rs246218 | C/T |
| chr16 | 16171339 | 2 | 7 | chr16 | 16171337 | rs7201980 | C/T |
| chr16 | 16188423 | 3 | 1 | chr16 | 16188421 | rs8056103 | C/T |
| chr16 | 16962895 | 3 | 1 | chr16 | 16962893 | rs6498647 | C/G |
| chr16 | 17268025 | 2 | 2 | chr16 | 17268023 | rs12708817 | C/T |
| chr16 | 17339930 | 1 | 3 | chr16 | 17339927 | rs4782002 | A/G |
| chr16 | 17346407 | 4 | 2 | chr16 | 17346406 | rs8050915 | A/G |
| chr16 | 17562789 | 2 | 5 | chr16 | 17562787 | rs62030762 | C/T |
| chr16 | 17989850 | 2 | 2 | chr16 | 17989849 | rs238848 | A/G |
| chr16 | 19002586 | 1 | 4 | chr16 | 19002585 | rs7184530 | A/G |
| chr16 | 19046150 | 5 | 3 | chr16 | 19046149 | rs4782208 | A/G |
| chr16 | 19792991 | 1 | 4 | chr16 | 19792990 | rs6497418 | A/G |
| chr16 | 21108454 | 12 | 2 | chr16 | 21108452 | rs2141997 | C/T |

| | | | | | | | |
|---|---|---|---|---|---|---|---|
| chr16 | 22676322 | 2 | 6 | chr16 | 22676321 | rs1895494 | A/G |
| chr16 | 22856893 | 1 | 7 | chr16 | 22856891 | rs756801 | G/T |
| chr16 | 23716769 | 3 | 1 | chr16 | 23716768 | rs2520026 | A/G |
| chr16 | 23820404 | 8 | 1 | chr16 | 23820403 | rs7193673 | A/G |
| chr16 | 24073632 | 3 | 6 | chr16 | 24073630 | rs432998 | C/T |
| chr16 | 24131774 | 1 | 6 | chr16 | 24131772 | rs198181 | C/T |
| chr16 | 24218004 | 6 | 3 | chr16 | 24218002 | rs2107119 | C/T |
| chr16 | 24919500 | 5 | 1 | chr16 | 24919499 | rs56278661 | G/T |
| chr16 | 24967122 | 3 | 1 | chr16 | 24967120 | rs72782559 | A/C |
| chr16 | 25046612 | 1 | 6 | chr16 | 25046612 | rs1549852 | C/G |
| chr16 | 25668566 | 1 | 6 | chr16 | 25668564 | rs7193807 | C/T |
| chr16 | 25790647 | 2 | 8 | chr16 | 25790645 | rs4628978 | C/T |
| chr16 | 25790647 | 2 | 8 | chr16 | 25790645 | rs71378394 | CG/TA |
| chr16 | 25790647 | 2 | 8 | chr16 | 25790646 | rs4787776 | A/G |
| chr16 | 26193347 | 1 | 5 | chr16 | 26193346 | rs9939804 | A/G |
| chr16 | 26240340 | 1 | 3 | chr16 | 26240340 | rs9923624 | A/G |
| chr16 | 26772221 | 5 | 6 | chr16 | 26772220 | rs11074802 | A/G |
| chr16 | 26879177 | 3 | 6 | chr16 | 26879175 | rs62029786 | A/C |
| chr16 | 26898386 | 1 | 7 | chr16 | 26898384 | rs4787889 | C/T |
| chr16 | 26898386 | 1 | 7 | chr16 | 26898385 | rs74015942 | A/G |
| chr16 | 27122882 | 1 | 4 | chr16 | 27122880 | rs908382 | G/T |
| chr16 | 27149110 | 2 | 2 | chr16 | 27149108 | rs34356479 | C/T |
| chr16 | 27852680 | 2 | 9 | chr16 | 27852679 | rs11861424 | C/G |
| chr16 | 29098581 | 2 | 10 | chr16 | 29098580 | rs153181 | A/G |
| chr16 | 29098829 | 1 | 4 | chr16 | 29098828 | rs12929818 | A/G |
| chr16 | 30423993 | 2 | 2 | chr16 | 30423992 | rs2073917 | C/T |
| chr16 | 31244021 | 2 | 3 | chr16 | 31244019 | rs7206295 | C/T |
| chr16 | 31620902 | 1 | 4 | chr16 | 31620900 | rs1919041 | C/G |
| chr16 | 31632339 | 8 | 2 | chr16 | 31632336 | rs2884713 | A/G |
| chr16 | 31632339 | 8 | 2 | chr16 | 31632337 | rs34798317 | -/A |
| chr16 | 31691169 | 6 | 1 | chr16 | 31691168 | rs2078924 | C/T |
| chr16 | 32208256 | 3 | 7 | chr16 | 32208255 | rs62044450 | A/G |

| | | | | | | | |
|---|---|---|---|---|---|---|---|
| chr16 | 32208256 | 3 | 7 | chr16 | 32208255 | rs67022513 | C/T |
| chr16 | 32208256 | 3 | 7 | chr16 | 32208255 | rs71244337 | A/G |
| chr16 | 33851777 | 2 | 8 | chr16 | 33851775 | rs74015499 | A/C |
| chr16 | 33857766 | 2 | 3 | chr16 | 33857765 | rs2017550 | C/G |
| chr16 | 33858596 | 421 | 2 | chr16 | 33858595 | rs62026787 | A/G |
| chr16 | 33860882 | 20 | 105 | chr16 | 33860880 | rs62026813 | C/G |
| chr16 | 33864465 | 101 | 527 | chr16 | 33864464 | rs62027914 | A/G |
| chr16 | 33865079 | 2 | 174 | chr16 | 33865076 | rs62027925 | C/T |
| chr16 | 33867145 | 402 | 1 | chr16 | 33867145 | rs62028792 | C/G |
| chr16 | 33869129 | 10 | 4 | chr16 | 33869128 | rs72797455 | A/G |
| chr16 | 33873843 | 395 | 2 | chr16 | 33873843 | rs72806958 | A/G |
| chr16 | 34233696 | 2 | 2 | chr16 | 34233695 | rs2623781 | C/T |
| chr16 | 44948253 | 28 | 43 | chr16 | 44948250 | rs9796843 | A/C |
| chr16 | 44948253 | 28 | 43 | chr16 | 44948250 | rs71241109 | G/T |
| chr16 | 44948253 | 28 | 43 | chr16 | 44948251 | rs4609877 | C/T |
| chr16 | 44948253 | 28 | 43 | chr16 | 44948251 | rs55747152 | C/T |
| chr16 | 44948253 | 28 | 43 | chr16 | 44948252 | rs56959057 | A/G |
| chr16 | 44948253 | 28 | 43 | chr16 | 44948253 | rs28826681 | A/G/T |
| chr16 | 44955214 | 28 | 43 | chr16 | 44955211 | rs13334940 | A/C |
| chr16 | 44955214 | 28 | 43 | chr16 | 44955212 | rs4616307 | C/T |
| chr16 | 44955214 | 28 | 43 | chr16 | 44955214 | rs7203295 | A/G |
| chr16 | 44960172 | 3 | 1 | chr16 | 44960169 | rs9796843 | A/C |
| chr16 | 44960172 | 3 | 1 | chr16 | 44960169 | rs56741231 | A/C |
| chr16 | 44960172 | 3 | 1 | chr16 | 44960170 | rs4280431 | A/G |
| chr16 | 44960172 | 3 | 1 | chr16 | 44960170 | rs12598315 | C/T |
| chr16 | 44960172 | 3 | 1 | chr16 | 44960170 | rs55747152 | C/T |
| chr16 | 44960172 | 3 | 1 | chr16 | 44960170 | rs67985318 | A/G |
| chr16 | 44960172 | 3 | 1 | chr16 | 44960172 | rs28771096 | C/G |
| chr16 | 44960172 | 3 | 1 | chr16 | 44960172 | rs28826681 | A/G/T |
| chr16 | 44978412 | 43 | 28 | chr16 | 44978409 | rs12597583 | A/C |
| chr16 | 44978412 | 43 | 28 | chr16 | 44978411 | rs4250386 | A/G |
| chr16 | 44978412 | 43 | 28 | chr16 | 44978411 | rs4280431 | A/G |

| | | | | | | | | |
|---|---|---|---|---|---|---|---|---|
| chr16 | 44978412 | 43 | 28 | chr16 | 44978411 | rs67985318 | | A/G |
| chr16 | 44986439 | 4 | 1 | chr16 | 44986437 | rs9936620 | | C/T |
| chr16 | 44986439 | 4 | 1 | chr16 | 44986438 | rs71240592 | | A/G |
| chr16 | 44986439 | 4 | 1 | chr16 | 44986439 | rs71233821 | | G/T |
| chr16 | 44990467 | 43 | 28 | chr16 | 44990464 | rs28819517 | | A/C/T |
| chr16 | 44990467 | 43 | 28 | chr16 | 44990466 | rs4967409 | | A/G |
| chr16 | 44990467 | 43 | 28 | chr16 | 44990467 | rs28824930 | | G/T |
| chr16 | 45646859 | 7 | 3 | chr16 | 45646857 | rs4625742 | | C/T |
| chr16 | 46757715 | 3 | 1 | chr16 | 46757713 | rs74018300 | | C/T |
| chr16 | 47241421 | 3 | 6 | chr16 | 47241420 | rs4493038 | | A/G |
| chr16 | 48089491 | 4 | 1 | chr16 | 48089489 | rs11076483 | | C/T |
| chr16 | 48121958 | 4 | 1 | chr16 | 48121956 | rs12918268 | | A/C |
| chr16 | 48308060 | 2 | 2 | chr16 | 48308059 | rs12448972 | | A/G |
| chr16 | 48456257 | 3 | 2 | chr16 | 48456255 | rs7196379 | | C/T |
| chr16 | 48627273 | 1 | 3 | chr16 | 48627272 | rs4640178 | | A/G |
| chr16 | 48632348 | 4 | 2 | chr16 | 48632346 | rs7206346 | | C/T |
| chr16 | 48859330 | 1 | 6 | chr16 | 48859329 | rs10852608 | | A/G |
| chr16 | 49016548 | 1 | 3 | chr16 | 49016546 | rs8182109 | | C/T |
| chr16 | 49647308 | 1 | 10 | chr16 | 49647307 | rs7196034 | | A/G |
| chr16 | 49847245 | 1 | 3 | chr16 | 49847244 | rs4564559 | | A/G |
| chr16 | 52767338 | 3 | 3 | chr16 | 52767336 | rs2542671 | | A/G |
| chr16 | 53357545 | 1 | 5 | chr16 | 53357543 | rs4238764 | | C/T |
| chr16 | 53979828 | 3 | 4 | chr16 | 53979826 | rs62027451 | | C/T |
| chr16 | 54364559 | 0 | 0 | chr16 | 54364557 | rs28626221 | | C/T |
| chr16 | 54441120 | 2 | 3 | chr16 | 54441118 | rs2160276 | | A/G |
| chr16 | 54588540 | 3 | 1 | chr16 | 54588538 | rs1510197 | | A/G |
| chr16 | 54822905 | 3 | 2 | chr16 | 54822904 | rs58590076 | | A/G |
| chr16 | 54822905 | 3 | 2 | chr16 | 54822905 | rs929872 | | A/G |
| chr16 | 54930409 | 4 | 3 | chr16 | 54930407 | rs12927669 | | A/C |
| chr16 | 55093011 | 3 | 1 | chr16 | 55093010 | rs9929628 | | A/G |
| chr16 | 55557450 | 2 | 2 | chr16 | 55557449 | rs708273 | | C/T |
| chr16 | 55980021 | 1 | 7 | chr16 | 55980019 | rs11859006 | | C/T |

| | | | | | | | |
|---|---|---|---|---|---|---|---|
| chr16 | 56217992 | 2 | 2 | chr16 | 56217991 | rs935739 | A/G |
| chr16 | 56255070 | 0 | 0 | chr16 | 56255068 | rs10852555 | A/C |
| chr16 | 56367451 | 3 | 7 | chr16 | 56367450 | rs2967163 | C/T |
| chr16 | 56504473 | 3 | 2 | chr16 | 56504473 | rs434961 | -/A/G |
| chr16 | 57577817 | 5 | 3 | chr16 | 57577816 | rs6500002 | A/G |
| chr16 | 58208233 | 3 | 5 | chr16 | 58208231 | rs1500297 | C/T |
| chr16 | 58598347 | 3 | 4 | chr16 | 58598346 | rs2550198 | C/T |
| chr16 | 58801417 | 1 | 4 | chr16 | 58801415 | rs12923165 | C/T |
| chr16 | 60172916 | 3 | 6 | chr16 | 60172915 | rs7196867 | G/T |
| chr16 | 60625840 | 12 | 2 | chr16 | 60625839 | rs12927313 | G/T |
| chr16 | 61236189 | 2 | 2 | chr16 | 61236187 | rs288620 | A/G |
| chr16 | 61678581 | 4 | 1 | chr16 | 61678580 | rs169130 | A/G |
| chr16 | 61786652 | 2 | 2 | chr16 | 61786649 | rs1865827 | C/T |
| chr16 | 62704591 | 5 | 5 | chr16 | 62704589 | rs2098721 | C/T |
| chr16 | 64372806 | 2 | 7 | chr16 | 64372805 | rs56336398 | A/G |
| chr16 | 65026014 | 4 | 2 | chr16 | 65026012 | rs893198 | A/G |
| chr16 | 67106097 | 2 | 2 | chr16 | 67106095 | rs696584 | C/T |
| chr16 | 67120464 | 4 | 1 | chr16 | 67120463 | rs4783562 | A/G |
| chr16 | 67414537 | 3 | 4 | chr16 | 67414536 | rs73562605 | C/G |
| chr16 | 68322967 | 5 | 6 | chr16 | 68322966 | rs2917683 | C/G |
| chr16 | 69283317 | 5 | 14 | chr16 | 69283315 | rs936297 | C/T |
| chr16 | 69576893 | 2 | 5 | chr16 | 69576891 | rs1798491 | C/T |
| chr16 | 69587678 | 5 | 1 | chr16 | 69587676 | rs925515 | C/G |
| chr16 | 69595929 | 2 | 6 | chr16 | 69595927 | rs2040825 | C/T |
| chr16 | 69658332 | 6 | 1 | chr16 | 69658330 | rs6416717 | C/T |
| chr16 | 69680966 | 4 | 2 | chr16 | 69680965 | rs8050954 | A/G |
| chr16 | 69859128 | 3 | 1 | chr16 | 69859127 | rs236014 | C/T |
| chr16 | 69972080 | 8 | 1 | chr16 | 69972079 | rs62055052 | C/G |
| chr16 | 70023589 | 1 | 3 | chr16 | 70023588 | rs4620964 | A/G |
| chr16 | 70693858 | 2 | 2 | chr16 | 70693857 | rs30438 | C/T |
| chr16 | 70863160 | 3 | 1 | chr16 | 70863158 | rs8063885 | C/T |
| chr16 | 71494071 | 2 | 2 | chr16 | 71494070 | rs4788481 | G/T |

| | | | | | | | |
|---|---|---|---|---|---|---|---|
| chr16 | 71619370 | 0 | 0 | chr16 | 71619368 | rs62055065 | C/T |
| chr16 | 72732792 | 6 | 2 | chr16 | 72732790 | rs11150440 | C/T |
| chr16 | 73433448 | 6 | 2 | chr16 | 73433446 | rs9941191 | C/T |
| chr16 | 74943375 | 4 | 2 | chr16 | 74943374 | rs9937260 | A/G |
| chr16 | 75605550 | 4 | 1 | chr16 | 75605549 | rs17768446 | A/G |
| chr16 | 75789095 | 6 | 2 | chr16 | 75789093 | rs4887894 | A/C |
| chr16 | 76061509 | 7 | 1 | chr16 | 76061508 | rs11645817 | A/G |
| chr16 | 76282071 | 1 | 5 | chr16 | 76282069 | rs308944 | A/G |
| chr16 | 76624186 | 2 | 3 | chr16 | 76624184 | rs12448742 | C/T |
| chr16 | 76629801 | 2 | 2 | chr16 | 76629800 | rs62043012 | A/G |
| chr16 | 77442684 | 9 | 4 | chr16 | 77442682 | rs8060856 | A/C |
| chr16 | 77581097 | 2 | 3 | chr16 | 77581096 | rs4362402 | A/G |
| chr16 | 77611009 | 11 | 2 | chr16 | 77611008 | rs7196173 | G/T |
| chr16 | 78130079 | 5 | 1 | chr16 | 78130077 | rs250134 | A/G |
| chr16 | 78506850 | 3 | 2 | chr16 | 78506848 | rs7195655 | C/T |
| chr16 | 78808973 | 3 | 1 | chr16 | 78808971 | rs8060494 | C/T |
| chr16 | 78819382 | 1 | 3 | chr16 | 78819379 | rs6564748 | A/C |
| chr16 | 78819382 | 1 | 3 | chr16 | 78819380 | rs12918598 | C/T |
| chr16 | 79751884 | 6 | 13 | chr16 | 79751882 | rs2317117 | A/G |
| chr16 | 79927099 | 2 | 2 | chr16 | 79927098 | rs16955064 | A/G |
| chr16 | 80083228 | 5 | 1 | chr16 | 80083227 | rs2926004 | A/G |
| chr16 | 80106928 | 3 | 2 | chr16 | 80106927 | rs72827196 | A/G |
| chr16 | 80106928 | 3 | 2 | chr16 | 80106928 | rs72827197 | G/T |
| chr16 | 80231040 | 0 | 0 | chr16 | 80231039 | rs17201380 | G/T |
| chr16 | 80459581 | 1 | 9 | chr16 | 80459579 | rs7196695 | C/T |
| chr16 | 80477030 | 27 | 1 | chr16 | 80477029 | rs4133124 | A/C |
| chr16 | 80514804 | 3 | 1 | chr16 | 80514803 | rs16955980 | G/T |
| chr16 | 81043259 | 1 | 6 | chr16 | 81043257 | rs2317976 | C/T |
| chr16 | 81045765 | 5 | 1 | chr16 | 81045763 | rs2873598 | C/T |
| chr16 | 81088548 | 4 | 3 | chr16 | 81088546 | rs8050741 | C/G |
| chr16 | 81599039 | 5 | 2 | chr16 | 81599037 | rs4783306 | A/C |
| chr16 | 81793908 | 3 | 1 | chr16 | 81793906 | rs9928871 | C/T |

| | | | | | | | |
|---|---|---|---|---|---|---|---|
| chr16 | 81821987 | 8 | 1 | chr16 | 81821985 | rs11640568 | C/T |
| chr16 | 82000301 | 1 | 5 | chr16 | 82000300 | rs889719 | C/T |
| chr16 | 82165099 | 3 | 1 | chr16 | 82165098 | rs10781970 | A/G |
| chr16 | 82321706 | 4 | 3 | chr16 | 82321704 | rs3096277 | C/T |
| chr16 | 82324875 | 1 | 17 | chr16 | 82324874 | rs389592 | A/G |
| chr16 | 82447661 | 3 | 2 | chr16 | 82447660 | rs397943 | C/T |
| chr16 | 82640286 | 3 | 3 | chr16 | 82640284 | rs8057673 | C/T |
| chr16 | 82788872 | 7 | 2 | chr16 | 82788871 | rs4410061 | G/T |
| chr16 | 83062637 | 2 | 8 | chr16 | 83062636 | rs692324 | C/T |
| chr16 | 83100831 | 5 | 1 | chr16 | 83100829 | rs193701 | A/G |
| chr16 | 83119460 | 3 | 1 | chr16 | 83119459 | rs405569 | A/C |
| chr16 | 83194282 | 6 | 2 | chr16 | 83194281 | rs12149601 | A/G |
| chr16 | 83242831 | 5 | 1 | chr16 | 83242828 | rs35742287 | C/T |
| chr16 | 83242831 | 5 | 1 | chr16 | 83242829 | rs34043136 | C/G |
| chr16 | 83403917 | 5 | 1 | chr16 | 83403915 | rs1532302 | C/T |
| chr16 | 83419235 | 3 | 12 | chr16 | 83419234 | rs7204018 | A/G |
| chr16 | 83436965 | 2 | 2 | chr16 | 83436964 | rs12051468 | A/G |
| chr16 | 83497981 | 2 | 3 | chr16 | 83497979 | rs1874008 | C/T |
| chr16 | 83778655 | 2 | 6 | chr16 | 83778652 | rs35543547 | -/C |
| chr16 | 83778655 | 2 | 6 | chr16 | 83778653 | rs11149717 | C/G |
| chr16 | 83778655 | 2 | 6 | chr16 | 83778653 | rs71380497 | CG/GA |
| chr16 | 83778655 | 2 | 6 | chr16 | 83778654 | rs11149718 | -/A/G |
| chr16 | 83849652 | 4 | 1 | chr16 | 83849652 | rs9319462 | C/G |
| chr16 | 83878080 | 1 | 3 | chr16 | 83878077 | rs4783156 | C/T |
| chr16 | 83995707 | 0 | 0 | chr16 | 83995707 | rs71992739 | -/T |
| chr16 | 84002421 | 0 | 0 | chr16 | 84002421 | rs67582190 | -/T |
| chr16 | 84003084 | 0 | 0 | chr16 | 84003084 | rs67265252 | -/T |
| chr16 | 84003216 | 0 | 0 | chr16 | 84003214 | rs71372922 | C/T |
| chr16 | 84003216 | 0 | 0 | chr16 | 84003216 | rs72395748 | -/T |
| chr16 | 84003282 | 0 | 0 | chr16 | 84003282 | rs67290577 | -/T |
| chr16 | 84003348 | 0 | 0 | chr16 | 84003348 | rs66476732 | -/T |
| chr16 | 84003414 | 0 | 0 | chr16 | 84003414 | rs72558616 | -/CT |

| | | | | | | | |
|---|---|---|---|---|---|---|---|
| chr16 | 84003646 | 0 | 0 | chr16 | 84003646 | rs72002217 | -/T |
| chr16 | 84003744 | 0 | 0 | chr16 | 84003743 | rs13338750 | G/T |
| chr16 | 84003744 | 0 | 0 | chr16 | 84003744 | rs67926262 | -/T |
| chr16 | 84043476 | 2 | 2 | chr16 | 84043474 | rs4783208 | C/T |
| chr16 | 84044263 | 8 | 3 | chr16 | 84044262 | rs4783210 | A/G |
| chr16 | 84063426 | 1 | 5 | chr16 | 84063424 | rs11149751 | -/C/T |
| chr16 | 84063426 | 1 | 5 | chr16 | 84063424 | rs71380499 | CT/TG |
| chr16 | 84063426 | 1 | 5 | chr16 | 84063425 | rs4783213 | G/T |
| chr16 | 84079868 | 7 | 14 | chr16 | 84079866 | rs9673791 | C/T |
| chr16 | 84106561 | 1 | 3 | chr16 | 84106560 | rs34335274 | G/T |
| chr16 | 84241838 | 3 | 1 | chr16 | 84241835 | rs59424991 | C/T |
| chr16 | 84525266 | 2 | 2 | chr16 | 84525265 | rs16940044 | A/G |
| chr16 | 84539748 | 2 | 3 | chr16 | 84539746 | rs305087 | C/T |
| chr16 | 84552932 | 1 | 3 | chr16 | 84552930 | rs908980 | A/G |
| chr16 | 84561612 | 1 | 4 | chr16 | 84561610 | rs12932970 | C/T |
| chr16 | 85023073 | 7 | 1 | chr16 | 85023070 | rs4843955 | C/T |
| chr16 | 85709346 | 1 | 14 | chr16 | 85709345 | rs6540008 | A/G |
| chr16 | 85918016 | 3 | 5 | chr16 | 85918015 | rs11642774 | A/G |
| chr16 | 86301994 | 2 | 4 | chr16 | 86301991 | rs3794668 | C/T |
| chr16 | 86436209 | 1 | 5 | chr16 | 86436208 | rs8052746 | A/G |
| chr16 | 86505996 | 3 | 2 | chr16 | 86505995 | rs4843737 | A/G |
| chr16 | 86659710 | 7 | 13 | chr16 | 86659708 | rs9921380 | C/T |
| chr16 | 86666629 | 6 | 2 | chr16 | 86666627 | rs28451517 | A/C |
| chr16 | 86722583 | 5 | 8 | chr16 | 86722583 | rs6540174 | G/T |
| chr16 | 86722583 | 5 | 8 | chr16 | 86722583 | rs71156299 | lengthTooLong |
| chr16 | 86722583 | 5 | 8 | chr16 | 86722583 | rs55864952 | -/GGGCCTGTT |
| chr16 | 86736928 | 2 | 10 | chr16 | 86736926 | rs9925529 | C/T |
| chr16 | 86737234 | 0 | 0 | chr16 | 86737232 | rs8052536 | C/T |
| chr16 | 86890798 | 1 | 3 | chr16 | 86890796 | rs11259972 | C/T |
| chr16 | 86920925 | 2 | 13 | chr16 | 86920923 | rs7500143 | C/T |
| chr16 | 86943605 | 3 | 2 | chr16 | 86943603 | rs28528911 | C/T |
| chr16 | 86997066 | 2 | 11 | chr16 | 86997063 | rs4047492 | A/G |

| | | | | | | | |
|---|---|---|---|---|---|---|---|
| chr16 | 87014081 | 4 | 2 | chr16 | 87014080 | rs4782360 | G/T |
| chr16 | 87034242 | 2 | 5 | chr16 | 87034241 | rs3848234 | A/G |
| chr16 | 87236843 | 4 | 11 | chr16 | 87236843 | rs9925947 | A/G |
| chr16 | 87325427 | 4 | 2 | chr16 | 87325426 | rs55924855 | A/G |
| chr16 | 87330626 | 5 | 4 | chr16 | 87330624 | rs13333358 | C/T |
| chr16 | 87350250 | 5 | 3 | chr16 | 87350247 | rs11076713 | C/T |
| chr16 | 87350250 | 5 | 3 | chr16 | 87350249 | rs11649371 | A/G |
| chr16 | 87372028 | 4 | 10 | chr16 | 87372027 | rs2926770 | A/G |
| chr16 | 87422938 | 1 | 4 | chr16 | 87422938 | rs7203543 | A/G |
| chr16 | 87568034 | 4 | 2 | chr16 | 87568032 | rs11862139 | C/T |
| chr16 | 87695898 | 5 | 3 | chr16 | 87695896 | rs4782460 | A/G |
| chr16 | 88210492 | 4 | 3 | chr16 | 88210490 | rs461857 | C/T |
| chr16 | 88258686 | 5 | 3 | chr16 | 88258685 | rs166297 | A/G |
| chr16 | 88324838 | 2 | 4 | chr16 | 88324837 | rs12709092 | C/G |
| chr16 | 88533102 | 3 | 1 | chr16 | 88533101 | rs8059126 | A/G |
| chr17 | 48264 | 6 | 3 | chr17 | 48263 | rs36164509 | A/G |
| chr17 | 52468 | 5 | 3 | chr17 | 52466 | rs7503116 | C/G |
| chr17 | 110579 | 3 | 4 | chr17 | 110578 | rs7219680 | A/G |
| chr17 | 149215 | 8 | 5 | chr17 | 149213 | rs9747497 | C/T |
| chr17 | 752100 | 2 | 4 | chr17 | 752098 | rs62070203 | C/G |
| chr17 | 801756 | 2 | 5 | chr17 | 801754 | rs3813436 | C/G |
| chr17 | 903520 | 2 | 3 | chr17 | 903519 | rs2440041 | A/G |
| chr17 | 980740 | 4 | 1 | chr17 | 980740 | rs2258815 | C/T |
| chr17 | 1147443 | 1 | 3 | chr17 | 1147442 | rs4790833 | C/T |
| chr17 | 1320269 | 5 | 2 | chr17 | 1320267 | rs9905106 | C/T |
| chr17 | 1842657 | 3 | 3 | chr17 | 1842655 | rs7225156 | C/G |
| chr17 | 2558222 | 5 | 1 | chr17 | 2558220 | rs68129009 | C/G |
| chr17 | 2754177 | 1 | 4 | chr17 | 2754176 | rs7503243 | A/G |
| chr17 | 3360911 | 4 | 3 | chr17 | 3360909 | rs2271158 | C/T |
| chr17 | 3445817 | 0 | 0 | chr17 | 3445816 | rs161384 | G/T |
| chr17 | 3475624 | 2 | 2 | chr17 | 3475622 | rs224513 | C/T |
| chr17 | 3492309 | 2 | 3 | chr17 | 3492307 | rs531939 | C/T |

| | | | | | | | |
|---|---|---|---|---|---|---|---|
| chr17 | 3507502 | 6 | 8 | chr17 | 3507500 | rs161401 | C/T |
| chr17 | 3617632 | 2 | 5 | chr17 | 3617631 | rs6502743 | G/T |
| chr17 | 3726026 | 1 | 4 | chr17 | 3726025 | rs7212114 | A/G |
| chr17 | 3766749 | 2 | 2 | chr17 | 3766748 | rs11659011 | A/G |
| chr17 | 3767081 | 7 | 2 | chr17 | 3767080 | rs11078477 | A/G |
| chr17 | 3767813 | 6 | 6 | chr17 | 3767812 | rs12944845 | A/G |
| chr17 | 3993020 | 3 | 1 | chr17 | 3993018 | rs1454122 | C/T |
| chr17 | 4526886 | 3 | 1 | chr17 | 4526885 | rs6502806 | A/G |
| chr17 | 4572708 | 2 | 4 | chr17 | 4572706 | rs34625233 | C/T |
| chr17 | 4585234 | 8 | 7 | chr17 | 4585232 | rs1876444 | C/T |
| chr17 | 4816291 | 3 | 1 | chr17 | 4816289 | rs238230 | C/T |
| chr17 | 5073041 | 4 | 2 | chr17 | 5073039 | rs2585281 | C/T |
| chr17 | 5684618 | 6 | 3 | chr17 | 5684617 | rs7224133 | A/G |
| chr17 | 5742935 | 3 | 1 | chr17 | 5742933 | rs35190120 | C/T |
| chr17 | 5926063 | 5 | 2 | chr17 | 5926061 | rs4448998 | C/T |
| chr17 | 5944613 | 3 | 1 | chr17 | 5944611 | rs4465648 | C/T |
| chr17 | 5949887 | 1 | 4 | chr17 | 5949886 | rs12185234 | A/G |
| chr17 | 5950031 | 4 | 3 | chr17 | 5950029 | rs12945598 | C/T |
| chr17 | 6013700 | 5 | 13 | chr17 | 6013700 | rs6502928 | A/G |
| chr17 | 6312462 | 3 | 2 | chr17 | 6312461 | rs35021251 | A/G |
| chr17 | 6639232 | 3 | 4 | chr17 | 6639230 | rs4556838 | A/C |
| chr17 | 6840283 | 4 | 2 | chr17 | 6840282 | rs312467 | C/G |
| chr17 | 6841109 | 4 | 1 | chr17 | 6841106 | rs312468 | C/T |
| chr17 | 6856125 | 1 | 3 | chr17 | 6856124 | rs2271316 | C/G |
| chr17 | 7283018 | 1 | 3 | chr17 | 7283017 | rs4151121 | A/G |
| chr17 | 7299587 | 4 | 3 | chr17 | 7299584 | rs2302762 | C/T |
| chr17 | 7486447 | 5 | 6 | chr17 | 7486445 | rs1641537 | A/G |
| chr17 | 7486447 | 5 | 6 | chr17 | 7486446 | rs34146209 | A/G |
| chr17 | 7518841 | 7 | 2 | chr17 | 7518839 | rs1625895 | A/G |
| chr17 | 7872014 | 2 | 6 | chr17 | 7872011 | rs8077945 | C/T |
| chr17 | 7964964 | 8 | 5 | chr17 | 7964961 | rs1442850 | C/T |
| chr17 | 8177109 | 3 | 3 | chr17 | 8177108 | rs9889671 | A/G |

| | | | | | | | |
|---|---|---|---|---|---|---|---|
| chr17 | 8321341 | 4 | 11 | chr17 | 8321340 | rs8070563 | A/G |
| chr17 | 8872225 | 2 | 4 | chr17 | 8872223 | rs6503167 | C/T |
| chr17 | 8929291 | 3 | 4 | chr17 | 8929290 | rs11867706 | A/G |
| chr17 | 8952507 | 4 | 5 | chr17 | 8952505 | rs7222641 | C/T |
| chr17 | 8958315 | 2 | 2 | chr17 | 8958313 | rs34136310 | C/G |
| chr17 | 9070606 | 4 | 5 | chr17 | 9070605 | rs2013732 | G/T |
| chr17 | 10059519 | 3 | 6 | chr17 | 10059517 | rs17688231 | C/T |
| chr17 | 10364566 | 7 | 2 | chr17 | 10364564 | rs9916035 | C/T |
| chr17 | 10661576 | 2 | 12 | chr17 | 10661575 | rs6503328 | A/G |
| chr17 | 10782474 | 3 | 1 | chr17 | 10782473 | rs6503336 | A/G |
| chr17 | 12367939 | 4 | 8 | chr17 | 12367938 | rs237346 | C/T |
| chr17 | 12874974 | 6 | 6 | chr17 | 12874971 | rs12452558 | A/C |
| chr17 | 13204171 | 4 | 1 | chr17 | 13204171 | rs2322876 | A/G |
| chr17 | 13397439 | 2 | 2 | chr17 | 13397438 | rs9797140 | A/G |
| chr17 | 13796482 | 1 | 3 | chr17 | 13796480 | rs4792423 | C/T |
| chr17 | 14091160 | 2 | 2 | chr17 | 14091159 | rs12946381 | A/G |
| chr17 | 14154854 | 4 | 1 | chr17 | 14154851 | rs4792467 | C/G |
| chr17 | 14449517 | 2 | 7 | chr17 | 14449516 | rs2079794 | A/G |
| chr17 | 14879160 | 2 | 5 | chr17 | 14879158 | rs67657020 | C/T |
| chr17 | 14964754 | 3 | 4 | chr17 | 14964752 | rs11654880 | C/T |
| chr17 | 15325723 | 2 | 3 | chr17 | 15325720 | rs9904490 | A/C |
| chr17 | 15334981 | 3 | 1 | chr17 | 15334980 | rs7211556 | A/G |
| chr17 | 15384459 | 1 | 4 | chr17 | 15384458 | rs2302253 | C/T |
| chr17 | 16342407 | 3 | 3 | chr17 | 16342406 | rs673760 | A/G |
| chr17 | 17274335 | 4 | 11 | chr17 | 17274333 | rs7217126 | C/T |
| chr17 | 17404060 | 4 | 1 | chr17 | 17404059 | rs4646369 | C/T |
| chr17 | 17995643 | 3 | 1 | chr17 | 17995641 | rs865923 | C/T |
| chr17 | 18038743 | 9 | 4 | chr17 | 18038742 | rs4925145 | A/G |
| chr17 | 18648318 | 4 | 1 | chr17 | 18648316 | rs2302253 | C/T |
| chr17 | 19332777 | 3 | 1 | chr17 | 19332776 | rs2428581 | C/T |
| chr17 | 21028555 | 2 | 6 | chr17 | 21028554 | rs3744225 | A/C |
| chr17 | 21134349 | 4 | 6 | chr17 | 21134347 | rs66859001 | C/G |

| | | | | | | | |
|---|---|---|---|---|---|---|---|
| chr17 | 21134541 | 1 | 9 | chr17 | 21134540 | rs73984411 | C/G |
| chr17 | 21134606 | 6 | 1 | chr17 | 21134605 | rs73984412 | A/G |
| chr17 | 21135379 | 4 | 1 | chr17 | 21135377 | rs72840057 | C/T |
| chr17 | 21136065 | 1 | 4 | chr17 | 21136064 | rs12602785 | G/T |
| chr17 | 21141390 | 3 | 1 | chr17 | 21141389 | rs5018957 | C/T |
| chr17 | 21154733 | 5 | 2 | chr17 | 21154731 | rs72838565 | C/T |
| chr17 | 21155736 | 1 | 11 | chr17 | 21155734 | rs5006071 | A/G |
| chr17 | 21165966 | 5 | 2 | chr17 | 21165964 | rs3866963 | A/G |
| chr17 | 21167596 | 4 | 4 | chr17 | 21167595 | rs1624721 | A/G |
| chr17 | 21178095 | 3 | 3 | chr17 | 21178094 | rs2099569 | A/G |
| chr17 | 21181396 | 4 | 8 | chr17 | 21181394 | rs514251 | C/T |
| chr17 | 21181396 | 4 | 8 | chr17 | 21181395 | rs72843830 | A/G |
| chr17 | 21182161 | 2 | 15 | chr17 | 21182160 | rs3952259 | C/T |
| chr17 | 21186394 | 9 | 1 | chr17 | 21186392 | rs1657748 | C/T |
| chr17 | 21186394 | 9 | 1 | chr17 | 21186393 | rs13342314 | A/G |
| chr17 | 21247100 | 6 | 5 | chr17 | 21247098 | rs4985787 | C/T |
| chr17 | 21249727 | 3 | 2 | chr17 | 21249724 | rs72846644 | C/G |
| chr17 | 21249727 | 3 | 2 | chr17 | 21249725 | rs72846645 | C/T |
| chr17 | 21250745 | 1 | 3 | chr17 | 21250742 | rs11655467 | A/C |
| chr17 | 21257593 | 8 | 4 | chr17 | 21257592 | rs1657733 | A/G |
| chr17 | 21261123 | 5 | 6 | chr17 | 21261121 | rs67680166 | C/T |
| chr17 | 21271138 | 1 | 4 | chr17 | 21271136 | rs4985872 | C/T |
| chr17 | 21278245 | 11 | 2 | chr17 | 21278245 | rs62051642 | G/T |
| chr17 | 21289245 | 24 | 3 | chr17 | 21289243 | rs73319936 | C/T |
| chr17 | 22353979 | 4 | 2 | chr17 | 22353978 | rs73981721 | C/G |
| chr17 | 22505109 | 1 | 8 | chr17 | 22505107 | rs9901179 | C/T |
| chr17 | 23723249 | 3 | 4 | chr17 | 23723247 | rs7212814 | C/G |
| chr17 | 24321954 | 1 | 3 | chr17 | 24321954 | rs16964692 | A/G |
| chr17 | 24784786 | 1 | 8 | chr17 | 24784785 | rs6505133 | A/G |
| chr17 | 25475992 | 3 | 1 | chr17 | 25475990 | rs9898353 | C/T |
| chr17 | 26827932 | 4 | 9 | chr17 | 26827930 | rs111195 | C/T |
| chr17 | 27210005 | 6 | 2 | chr17 | 27210004 | rs11542477 | A/G |

| | | | | | | | |
|---|---|---|---|---|---|---|---|
| chr17 | 27246116 | 4 | 1 | chr17 | 27246114 | rs3760454 | A/G |
| chr17 | 28930784 | 4 | 1 | chr17 | 28930782 | rs4794960 | C/T |
| chr17 | 29731885 | 4 | 2 | chr17 | 29731884 | rs150524 | A/G |
| chr17 | 30084616 | 5 | 3 | chr17 | 30084614 | rs56054512 | C/T |
| chr17 | 30084616 | 5 | 3 | chr17 | 30084615 | rs4795992 | A/G |
| chr17 | 30425569 | 8 | 1 | chr17 | 30425567 | rs72823667 | C/T |
| chr17 | 30521444 | 1 | 3 | chr17 | 30521443 | rs7213921 | A/G |
| chr17 | 31360623 | 3 | 5 | chr17 | 31360622 | rs1734959 | C/T |
| chr17 | 31606042 | 0 | 0 | chr17 | 31606040 | rs3874976 | A/G |
| chr17 | 33176494 | 8 | 7 | chr17 | 33176493 | rs11651052 | A/G |
| chr17 | 33593373 | 0 | 0 | chr17 | 33593371 | rs4026192 | A/G |
| chr17 | 34477944 | 4 | 8 | chr17 | 34477944 | rs4416046 | A/G |
| chr17 | 35273947 | 2 | 3 | chr17 | 35273944 | rs1453559 | A/G |
| chr17 | 35314966 | 2 | 4 | chr17 | 35314964 | rs4795399 | C/T |
| chr17 | 35426468 | 3 | 4 | chr17 | 35426467 | rs2227338 | A/G |
| chr17 | 36165633 | 2 | 6 | chr17 | 36165631 | rs11078956 | C/T |
| chr17 | 37137122 | 5 | 8 | chr17 | 37137120 | rs7216154 | C/T |
| chr17 | 37141628 | 2 | 3 | chr17 | 37141627 | rs3895104 | C/T |
| chr17 | 37212959 | 4 | 1 | chr17 | 37212958 | rs3809876 | A/G |
| chr17 | 37302999 | 3 | 2 | chr17 | 37302998 | rs4796736 | A/G |
| chr17 | 37427379 | 6 | 4 | chr17 | 37427376 | rs9894001 | A/C |
| chr17 | 37510690 | 2 | 4 | chr17 | 37510688 | rs2074158 | A/G |
| chr17 | 37932686 | 4 | 3 | chr17 | 37932684 | rs629286 | C/T |
| chr17 | 38088978 | 3 | 3 | chr17 | 38088976 | rs3760384 | A/C |
| chr17 | 38155351 | 7 | 1 | chr17 | 38155349 | rs752313 | C/T |
| chr17 | 39420994 | 14 | 2 | chr17 | 39420993 | rs1731885 | C/G |
| chr17 | 39827571 | 1 | 4 | chr17 | 39827570 | rs2037958 | A/C |
| chr17 | 40210775 | 3 | 3 | chr17 | 40210773 | rs2070603 | C/T |
| chr17 | 40524918 | 1 | 3 | chr17 | 40524917 | rs2239919 | A/G |
| chr17 | 41016700 | 14 | 1 | chr17 | 41016699 | rs2950659 | A/G |
| chr17 | 41114832 | 5 | 2 | chr17 | 41114830 | rs17687838 | C/T |
| chr17 | 41183202 | 3 | 3 | chr17 | 41183201 | rs12150451 | A/G |

| | | | | | | | | |
|---|---|---|---|---|---|---|---|---|
| chr17 | 41209292 | 2 | 3 | chr17 | 41209291 | rs55849949 | | A/G |
| chr17 | 41274394 | 3 | 3 | chr17 | 41274392 | rs56971664 | | C/T |
| chr17 | 41299446 | 4 | 1 | chr17 | 41299443 | rs62055469 | | A/C |
| chr17 | 41299446 | 4 | 1 | chr17 | 41299445 | rs62055470 | | A/G |
| chr17 | 41707062 | 4 | 2 | chr17 | 41707061 | rs2668617 | | A/G |
| chr17 | 42327419 | 5 | 1 | chr17 | 42327417 | rs197929 | | C/T |
| chr17 | 42423388 | 2 | 2 | chr17 | 42423386 | rs8068715 | | A/C |
| chr17 | 43414701 | 13 | 4 | chr17 | 43414699 | rs2597176 | | C/T |
| chr17 | 44236748 | 3 | 6 | chr17 | 44236746 | rs489005 | | C/T |
| chr17 | 44353901 | 16 | 3 | chr17 | 44353900 | rs58838744 | | A/G |
| chr17 | 44934113 | 3 | 3 | chr17 | 44934111 | rs627905 | | C/T |
| chr17 | 45323240 | 4 | 3 | chr17 | 45323239 | rs430606 | | C/T |
| chr17 | 45331932 | 5 | 3 | chr17 | 45331931 | rs271671 | | A/G |
| chr17 | 45550912 | 1 | 4 | chr17 | 45550912 | rs739499 | | A/C |
| chr17 | 45587906 | 1 | 3 | chr17 | 45587905 | rs2586463 | | C/T |
| chr17 | 45713418 | 25 | 7 | chr17 | 45713417 | rs2254177 | | C/T |
| chr17 | 45716524 | 2 | 9 | chr17 | 45716522 | rs2586436 | | A/G |
| chr17 | 46350777 | 6 | 2 | chr17 | 46350776 | rs758633 | | A/G |
| chr17 | 49444407 | 5 | 2 | chr17 | 49444405 | rs11079107 | | C/T |
| chr17 | 50978009 | 3 | 1 | chr17 | 50978008 | rs4239197 | | A/G |
| chr17 | 52174432 | 9 | 1 | chr17 | 52174431 | rs8073799 | | A/G |
| chr17 | 52312843 | 1 | 6 | chr17 | 52312842 | rs11868917 | | G/T |
| chr17 | 53234754 | 13 | 1 | chr17 | 53234752 | rs2685521 | | A/G |
| chr17 | 53492421 | 1 | 5 | chr17 | 53492419 | rs2526658 | | C/T |
| chr17 | 53770395 | 4 | 1 | chr17 | 53770394 | rs2632527 | | A/G |
| chr17 | 53784672 | 5 | 1 | chr17 | 53784671 | rs2680703 | | C/T |
| chr17 | 53784672 | 5 | 1 | chr17 | 53784671 | rs66475354 | | -/A |
| chr17 | 56568417 | 2 | 2 | chr17 | 56568415 | rs4419113 | | C/T |
| chr17 | 58120518 | 0 | 0 | chr17 | 58120517 | rs2465428 | | A/C |
| chr17 | 58703349 | 11 | 3 | chr17 | 58703348 | rs2440144 | | C/T |
| chr17 | 58910259 | 1 | 4 | chr17 | 58910258 | rs4296 | | A/G |
| chr17 | 59360229 | 1 | 5 | chr17 | 59360228 | rs1051684 | | C/T |

| | | | | | | | |
|---|---|---|---|---|---|---|---|
| chr17 | 59474465 | 4 | 2 | chr17 | 59474464 | rs196909 | A/G |
| chr17 | 59499404 | 13 | 1 | chr17 | 59499403 | rs196939 | A/G |
| chr17 | 59605731 | 3 | 1 | chr17 | 59605729 | rs903286 | C/T |
| chr17 | 59713821 | 3 | 3 | chr17 | 59713819 | rs4968715 | C/T |
| chr17 | 60482823 | 5 | 1 | chr17 | 60482822 | rs7216195 | C/G |
| chr17 | 60964252 | 1 | 7 | chr17 | 60964250 | rs9915936 | C/T |
| chr17 | 61723869 | 3 | 1 | chr17 | 61723868 | rs11079648 | A/G |
| chr17 | 61945869 | 4 | 3 | chr17 | 61945867 | rs8064886 | C/T |
| chr17 | 61976115 | 3 | 3 | chr17 | 61976114 | rs11658528 | G/T |
| chr17 | 62254074 | 3 | 13 | chr17 | 62254072 | rs4791029 | A/G |
| chr17 | 62365469 | 5 | 2 | chr17 | 62365467 | rs7211879 | C/G |
| chr17 | 63668122 | 5 | 2 | chr17 | 63668120 | rs73338770 | C/T |
| chr17 | 63713071 | 4 | 3 | chr17 | 63713070 | rs12952394 | C/G |
| chr17 | 64943925 | 1 | 9 | chr17 | 64943923 | rs817566 | A/G |
| chr17 | 65803715 | 6 | 2 | chr17 | 65803713 | rs401070 | C/T |
| chr17 | 66190289 | 5 | 1 | chr17 | 66190289 | rs9302921 | A/G |
| chr17 | 66776509 | 1 | 5 | chr17 | 66776507 | rs4500786 | C/T |
| chr17 | 67872323 | 3 | 8 | chr17 | 67872322 | rs11654064 | A/G |
| chr17 | 68284388 | 5 | 1 | chr17 | 68284387 | rs2567531 | C/T |
| chr17 | 68284440 | 2 | 4 | chr17 | 68284437 | rs1077768 | C/G |
| chr17 | 68642557 | 2 | 3 | chr17 | 68642555 | rs2344994 | C/T |
| chr17 | 68745331 | 8 | 2 | chr17 | 68745330 | rs966939 | C/T |
| chr17 | 68828737 | 4 | 2 | chr17 | 68828736 | rs1472455 | A/G |
| chr17 | 68907503 | 3 | 1 | chr17 | 68907501 | rs937447 | A/G |
| chr17 | 68911827 | 1 | 5 | chr17 | 68911825 | rs6501629 | C/T |
| chr17 | 68999734 | 1 | 3 | chr17 | 68999733 | rs1872076 | A/G |
| chr17 | 69059052 | 1 | 11 | chr17 | 69059051 | rs28550663 | A/G |
| chr17 | 69214043 | 4 | 5 | chr17 | 69214042 | rs2661602 | A/G |
| chr17 | 69689800 | 5 | 3 | chr17 | 69689798 | rs12950777 | C/T |
| chr17 | 69827812 | 3 | 1 | chr17 | 69827811 | rs6501707 | A/G |
| chr17 | 70047157 | 1 | 4 | chr17 | 70047155 | rs2655610 | A/G |
| chr17 | 70056915 | 3 | 1 | chr17 | 70056914 | rs783235 | A/G |

| | | | | | | | |
|---|---|---|---|---|---|---|---|
| chr17 | 70301969 | 6 | 7 | chr17 | 70301967 | rs8076317 | C/T |
| chr17 | 70303213 | 2 | 10 | chr17 | 70303212 | rs7221435 | A/G |
| chr17 | 70752154 | 3 | 2 | chr17 | 70752153 | rs2306216 | A/G |
| chr17 | 71071224 | 7 | 1 | chr17 | 71071223 | rs1671027 | C/T |
| chr17 | 71099378 | 3 | 2 | chr17 | 71099378 | rs936056 | C/T |
| chr17 | 71132400 | 0 | 0 | chr17 | 71132398 | rs820187 | G/T |
| chr17 | 71324504 | 1 | 9 | chr17 | 71324503 | rs9908146 | A/G |
| chr17 | 71814182 | 1 | 6 | chr17 | 71814180 | rs73996307 | C/T |
| chr17 | 71872258 | 3 | 2 | chr17 | 71872256 | rs8066048 | C/T |
| chr17 | 72432194 | 2 | 4 | chr17 | 72432193 | rs749868 | A/G |
| chr17 | 72441192 | 6 | 3 | chr17 | 72441190 | rs2166086 | C/T |
| chr17 | 72531885 | 3 | 2 | chr17 | 72531885 | rs9890579 | A/G |
| chr17 | 72605743 | 4 | 1 | chr17 | 72605742 | rs2678771 | C/T |
| chr17 | 72820946 | 2 | 8 | chr17 | 72820945 | rs11654929 | A/G |
| chr17 | 72895993 | 6 | 4 | chr17 | 72895991 | rs94056 | A/G |
| chr17 | 72916829 | 5 | 1 | chr17 | 72916828 | rs7216909 | A/G |
| chr17 | 72924273 | 4 | 5 | chr17 | 72924272 | rs35236538 | -/A/G |
| chr17 | 72924273 | 4 | 5 | chr17 | 72924272 | rs67415822 | A/G |
| chr17 | 72924273 | 4 | 5 | chr17 | 72924272 | rs35892416 | -/A |
| chr17 | 73000839 | 4 | 2 | chr17 | 73000838 | rs167498 | A/G |
| chr17 | 73063504 | 2 | 5 | chr17 | 73063502 | rs2304924 | A/G |
| chr17 | 73263401 | 1 | 3 | chr17 | 73263400 | rs9907607 | A/G |
| chr17 | 73652664 | 0 | 0 | chr17 | 73652663 | rs72896265 | A/G |
| chr17 | 73731802 | 4 | 2 | chr17 | 73731800 | rs1042489 | C/T |
| chr17 | 73869488 | 6 | 4 | chr17 | 73869486 | rs34309315 | C/T |
| chr17 | 74118336 | 7 | 7 | chr17 | 74118335 | rs9915834 | A/G |
| chr17 | 74429726 | 4 | 6 | chr17 | 74429725 | rs7212662 | G/T |
| chr17 | 74489062 | 6 | 1 | chr17 | 74489060 | rs4789850 | A/G |
| chr17 | 74598401 | 3 | 1 | chr17 | 74598400 | rs62063826 | A/G |
| chr17 | 74671060 | 0 | 0 | chr17 | 74671059 | rs11649910 | A/G |
| chr17 | 74673838 | 0 | 0 | chr17 | 74673836 | rs11344345 | -/C |
| chr17 | 74754058 | 4 | 1 | chr17 | 74754056 | rs72846829 | C/T |

| | | | | | | | |
|---|---|---|---|---|---|---|---|
| chr17 | 74754058 | 4 | 1 | chr17 | 74754057 | rs12600558 | A/G |
| chr17 | 74760319 | 2 | 2 | chr17 | 74760317 | rs8066616 | C/T |
| chr17 | 74760319 | 2 | 2 | chr17 | 74760318 | rs8066487 | A/G |
| chr17 | 74760319 | 2 | 2 | chr17 | 74760318 | rs67859028 | CA/TG |
| chr17 | 74763014 | 4 | 5 | chr17 | 74763013 | rs898527 | C/T |
| chr17 | 74764964 | 3 | 1 | chr17 | 74764963 | rs8064357 | A/G |
| chr17 | 74774528 | 3 | 2 | chr17 | 74774526 | rs4789877 | C/T |
| chr17 | 74885905 | 1 | 7 | chr17 | 74885903 | rs907916 | A/G |
| chr17 | 74885905 | 1 | 7 | chr17 | 74885904 | rs751847 | C/T |
| chr17 | 74887247 | 3 | 3 | chr17 | 74887245 | rs4789892 | C/T |
| chr17 | 74937174 | 15 | 1 | chr17 | 74937172 | rs9894959 | C/T |
| chr17 | 75688356 | 2 | 2 | chr17 | 75688354 | rs2304851 | G/T |
| chr17 | 76323012 | 2 | 2 | chr17 | 76323012 | rs9915378 | A/G |
| chr17 | 76358343 | 6 | 6 | chr17 | 76358342 | rs4380096 | A/G |
| chr17 | 76362340 | 2 | 2 | chr17 | 76362338 | rs9900956 | C/T |
| chr17 | 76388118 | 1 | 4 | chr17 | 76388116 | rs7208789 | C/T |
| chr17 | 76424475 | 6 | 7 | chr17 | 76424474 | rs2138117 | C/T |
| chr17 | 76434619 | 5 | 8 | chr17 | 76434617 | rs2589157 | A/G |
| chr17 | 76487241 | 3 | 1 | chr17 | 76487239 | rs4969224 | C/T |
| chr17 | 76499545 | 0 | 0 | chr17 | 76499543 | rs4558471 | G/T |
| chr17 | 76509748 | 1 | 3 | chr17 | 76509747 | rs9901846 | A/G |
| chr17 | 76526950 | 3 | 2 | chr17 | 76526948 | rs7219318 | C/T |
| chr17 | 76531912 | 1 | 8 | chr17 | 76531911 | rs4508478 | C/T |
| chr17 | 76628026 | 2 | 7 | chr17 | 76628024 | rs4969357 | C/T |
| chr17 | 76629696 | 3 | 2 | chr17 | 76629694 | rs7405730 | C/T |
| chr17 | 76680246 | 3 | 7 | chr17 | 76680244 | rs4969384 | C/T |
| chr17 | 76787993 | 4 | 2 | chr17 | 76787992 | rs906176 | C/T |
| chr17 | 76919102 | 2 | 5 | chr17 | 76919101 | rs4969432 | A/G |
| chr17 | 77041076 | 3 | 5 | chr17 | 77041075 | rs4969273 | A/G |
| chr17 | 77071069 | 1 | 16 | chr17 | 77071068 | rs9905985 | A/G |
| chr17 | 77077076 | 2 | 4 | chr17 | 77077075 | rs6565578 | G/T |
| chr17 | 77100435 | 6 | 2 | chr17 | 77100433 | rs9646446 | A/G |

| | | | | | | | |
|---|---|---|---|---|---|---|---|
| chr17 | 77183678 | 0 | 0 | chr17 | 77183678 | rs10608622 | -/GCCG |
| chr17 | 77199647 | 6 | 6 | chr17 | 77199646 | rs6565604 | A/G |
| chr17 | 77556008 | 3 | 2 | chr17 | 77556006 | rs7213474 | C/T |
| chr17 | 77600345 | 1 | 6 | chr17 | 77600343 | rs62078730 | C/T |
| chr17 | 77646756 | 2 | 2 | chr17 | 77646754 | rs62078745 | C/T |
| chr17 | 77656293 | 9 | 5 | chr17 | 77656292 | rs6416857 | A/G |
| chr17 | 77868543 | 3 | 1 | chr17 | 77868541 | rs3176829 | A/G |
| chr17 | 77878366 | 9 | 3 | chr17 | 77878364 | rs73999868 | C/T |
| chr17 | 77936331 | 0 | 0 | chr17 | 77936329 | rs9913934 | C/T |
| chr17 | 77966171 | 5 | 2 | chr17 | 77966169 | rs12451322 | C/G |
| chr17 | 78095652 | 5 | 1 | chr17 | 78095650 | rs72861057 | C/T |
| chr17 | 78144608 | 6 | 3 | chr17 | 78144607 | rs8081532 | G/T |
| chr17 | 78161409 | 10 | 2 | chr17 | 78161408 | rs11653029 | A/G |
| chr17 | 78238888 | 4 | 5 | chr17 | 78238886 | rs2244304 | C/T |
| chr17 | 78278416 | 2 | 6 | chr17 | 78278414 | rs2243446 | A/G |
| chr17 | 78289700 | 4 | 9 | chr17 | 78289698 | rs2253149 | A/C |
| chr17 | 78346076 | 5 | 3 | chr17 | 78346075 | rs11655811 | G/T |
| chr17 | 78442313 | 1 | 4 | chr17 | 78442312 | rs7221839 | A/G |
| chr17 | 78574388 | 0 | 0 | chr17 | 78574388 | rs28580036 | C/G |
| chr18 | 1883 | 3 | 2 | chr18 | 1881 | rs6505962 | C/T |
| chr18 | 98157 | 9 | 5 | chr18 | 98147 | rs71274240 | CTGTTAACCAA/TGATAACCTG |
| chr18 | 98157 | 9 | 5 | chr18 | 98154 | rs35091823 | AG/GC |
| chr18 | 98157 | 9 | 5 | chr18 | 98156 | rs36008153 | A/G |
| chr18 | 98157 | 9 | 5 | chr18 | 98156 | rs66474984 | C/T |
| chr18 | 98224 | 6 | 4 | chr18 | 98223 | rs35463262 | A/G |
| chr18 | 98224 | 6 | 4 | chr18 | 98223 | rs35586560 | -/G |
| chr18 | 98245 | 6 | 2 | chr18 | 98242 | rs34398465 | C/T |
| chr18 | 98245 | 6 | 2 | chr18 | 98242 | rs71247656 | A/G |
| chr18 | 98245 | 6 | 2 | chr18 | 98245 | rs28789826 | G/T |
| chr18 | 98871 | 1 | 5 | chr18 | 98868 | rs34478765 | A/C |
| chr18 | 98871 | 1 | 5 | chr18 | 98870 | rs35208587 | CA/GC |
| chr18 | 98871 | 1 | 5 | chr18 | 98870 | rs35711343 | -/G |

| | | | | | | | |
|---|---|---|---|---|---|---|---|
| chr18 | 100443 | 2 | 4 | chr18 | 100440 | rs35336654 | C/G |
| chr18 | 100443 | 2 | 4 | chr18 | 100441 | rs71209551 | G/T |
| chr18 | 100443 | 2 | 4 | chr18 | 100442 | rs71227432 | A/G |
| chr18 | 100443 | 2 | 4 | chr18 | 100442 | rs35619444 | -/G |
| chr18 | 480093 | 14 | 10 | chr18 | 480091 | rs5004259 | C/T |
| chr18 | 585606 | 1 | 8 | chr18 | 585604 | rs543813 | A/G |
| chr18 | 872387 | 3 | 6 | chr18 | 872385 | rs1940579 | A/C/T |
| chr18 | 1818321 | 2 | 2 | chr18 | 1818320 | rs2580154 | C/T |
| chr18 | 2136395 | 1 | 4 | chr18 | 2136394 | rs685058 | A/G |
| chr18 | 2384863 | 2 | 2 | chr18 | 2384861 | rs7242554 | C/G |
| chr18 | 2481933 | 7 | 1 | chr18 | 2481931 | rs2644192 | C/T |
| chr18 | 2482934 | 3 | 2 | chr18 | 2482933 | rs2682136 | C/T |
| chr18 | 2487385 | 2 | 3 | chr18 | 2487384 | rs770225 | A/G |
| chr18 | 2843819 | 4 | 1 | chr18 | 2843817 | rs11659151 | A/C |
| chr18 | 3660407 | 1 | 3 | chr18 | 3660405 | rs1791390 | A/G |
| chr18 | 3775644 | 7 | 3 | chr18 | 3775642 | rs3985696 | C/T |
| chr18 | 4669299 | 8 | 3 | chr18 | 4669297 | rs7238739 | C/T |
| chr18 | 5022382 | 1 | 3 | chr18 | 5022381 | rs2874481 | A/G |
| chr18 | 5142795 | 2 | 2 | chr18 | 5142794 | rs366411 | C/T |
| chr18 | 6673819 | 2 | 3 | chr18 | 6673817 | rs71360046 | C/T |
| chr18 | 6844267 | 1 | 3 | chr18 | 6844265 | rs2376531 | A/G |
| chr18 | 6927973 | 1 | 4 | chr18 | 6927973 | rs2105657 | C/T |
| chr18 | 6980995 | 6 | 2 | chr18 | 6980994 | rs17440727 | A/G |
| chr18 | 7107624 | 2 | 2 | chr18 | 7107622 | rs334406 | C/G |
| chr18 | 7837573 | 4 | 4 | chr18 | 7837571 | rs1443613 | A/G |
| chr18 | 8324016 | 4 | 2 | chr18 | 8324014 | rs101210 | C/T |
| chr18 | 8791634 | 2 | 13 | chr18 | 8791633 | rs516579 | G/T |
| chr18 | 8793227 | 2 | 2 | chr18 | 8793226 | rs471016 | G/T |
| chr18 | 8934209 | 7 | 7 | chr18 | 8934207 | rs11081443 | C/T |
| chr18 | 8980803 | 1 | 4 | chr18 | 8980802 | rs8090221 | A/G |
| chr18 | 9173741 | 4 | 2 | chr18 | 9173741 | rs62085888 | A/G |
| chr18 | 9395274 | 2 | 2 | chr18 | 9395272 | rs8098412 | C/T |

| | | | | | | | |
|---|---|---|---|---|---|---|---|
| chr18 | 9830751 | 2 | 3 | chr18 | 9830749 | rs661092 | C/T |
| chr18 | 9999696 | 2 | 2 | chr18 | 9999694 | rs8088314 | A/C |
| chr18 | 10292240 | 3 | 4 | chr18 | 10292239 | rs10775414 | A/G |
| chr18 | 10502627 | 2 | 2 | chr18 | 10502625 | rs563331 | A/G |
| chr18 | 10755352 | 2 | 4 | chr18 | 10755351 | rs2178100 | A/G |
| chr18 | 10766893 | 3 | 1 | chr18 | 10766891 | rs2178102 | C/T |
| chr18 | 10999275 | 6 | 3 | chr18 | 10999274 | rs12326591 | G/T |
| chr18 | 11136333 | 1 | 4 | chr18 | 11136331 | rs8096811 | C/T |
| chr18 | 11982726 | 3 | 2 | chr18 | 11982724 | rs589247 | C/T |
| chr18 | 12052985 | 3 | 2 | chr18 | 12052984 | rs11875970 | A/G |
| chr18 | 12078280 | 2 | 3 | chr18 | 12078278 | rs7241974 | C/T |
| chr18 | 12693067 | 2 | 4 | chr18 | 12693066 | rs3809916 | A/G |
| chr18 | 12693067 | 2 | 4 | chr18 | 12693066 | rs11537900 | A/G |
| chr18 | 12886961 | 1 | 5 | chr18 | 12886960 | rs11080610 | A/G |
| chr18 | 12983196 | 2 | 10 | chr18 | 12983194 | rs28619103 | A/C |
| chr18 | 13085610 | 1 | 5 | chr18 | 13085608 | rs474337 | A/G |
| chr18 | 13575288 | 3 | 2 | chr18 | 13575286 | rs1284401 | A/G |
| chr18 | 13972686 | 1 | 4 | chr18 | 13972685 | rs56051246 | A/G |
| chr18 | 14135297 | 2 | 2 | chr18 | 14135295 | rs8093275 | C/T |
| chr18 | 18249752 | 2 | 2 | chr18 | 18249750 | rs9946145 | C/T |
| chr18 | 18639000 | 8 | 1 | chr18 | 18638999 | rs2044671 | A/G |
| chr18 | 18951352 | 1 | 17 | chr18 | 18951351 | rs9304284 | A/G |
| chr18 | 19322116 | 2 | 2 | chr18 | 19322115 | rs11877146 | A/G |
| chr18 | 19425978 | 3 | 2 | chr18 | 19425977 | rs9304355 | A/G |
| chr18 | 19993724 | 3 | 1 | chr18 | 19993722 | rs1049684 | A/G |
| chr18 | 20377741 | 4 | 2 | chr18 | 20377740 | rs949315 | C/T |
| chr18 | 20906815 | 3 | 10 | chr18 | 20906814 | rs9957365 | A/G |
| chr18 | 21434903 | 2 | 5 | chr18 | 21434902 | rs273700 | C/T |
| chr18 | 21653977 | 2 | 2 | chr18 | 21653976 | rs12608438 | A/G |
| chr18 | 21658580 | 8 | 1 | chr18 | 21658578 | rs12608228 | C/T |
| chr18 | 21713213 | 3 | 7 | chr18 | 21713212 | rs12604801 | A/G |
| chr18 | 21781806 | 13 | 2 | chr18 | 21781804 | rs1380191 | A/G |

| | | | | | | | |
|---|---|---|---|---|---|---|---|
| chr18 | 22375085 | 3 | 1 | chr18 | 22375084 | rs4800261 | A/G |
| chr18 | 22746098 | 1 | 4 | chr18 | 22746096 | rs232358 | A/G |
| chr18 | 22831218 | 3 | 1 | chr18 | 22831217 | rs1154210 | C/T |
| chr18 | 23471163 | 8 | 5 | chr18 | 23471162 | rs9952238 | A/G |
| chr18 | 25473492 | 2 | 2 | chr18 | 25473490 | rs8091831 | C/T |
| chr18 | 25950832 | 6 | 2 | chr18 | 25950831 | rs4799783 | A/G |
| chr18 | 26000773 | 5 | 3 | chr18 | 26000771 | rs7229818 | C/T |
| chr18 | 26443638 | 4 | 5 | chr18 | 26443637 | rs28580004 | A/G |
| chr18 | 26883145 | 1 | 6 | chr18 | 26883144 | rs72922480 | A/G |
| chr18 | 28030680 | 4 | 2 | chr18 | 28030679 | rs965776 | C/T |
| chr18 | 28664688 | 4 | 3 | chr18 | 28664686 | rs1941282 | C/T |
| chr18 | 29787092 | 7 | 3 | chr18 | 29787090 | rs9966468 | C/T |
| chr18 | 29836889 | 8 | 1 | chr18 | 29836887 | rs17747955 | C/T |
| chr18 | 31752452 | 2 | 2 | chr18 | 31752450 | rs62102248 | C/T |
| chr18 | 31940599 | 7 | 1 | chr18 | 31940597 | rs9807590 | C/T |
| chr18 | 31959133 | 8 | 6 | chr18 | 31959132 | rs2236718 | A/C |
| chr18 | 32185836 | 2 | 3 | chr18 | 32185834 | rs4799417 | C/T |
| chr18 | 32318712 | 6 | 3 | chr18 | 32318711 | rs6507172 | A/G |
| chr18 | 32474490 | 5 | 1 | chr18 | 32474488 | rs2644261 | C/T |
| chr18 | 33069166 | 2 | 2 | chr18 | 33069164 | rs72898336 | C/T |
| chr18 | 33108127 | 4 | 1 | chr18 | 33108126 | rs1662914 | A/G |
| chr18 | 33153862 | 4 | 2 | chr18 | 33153860 | rs650303 | A/G |
| chr18 | 33300063 | 19 | 1 | chr18 | 33300062 | rs1941947 | A/G |
| chr18 | 33345282 | 1 | 4 | chr18 | 33345281 | rs948539 | A/G |
| chr18 | 33359026 | 3 | 1 | chr18 | 33359025 | rs4799937 | A/G |
| chr18 | 33793991 | 8 | 5 | chr18 | 33793989 | rs12971021 | C/T |
| chr18 | 36333260 | 2 | 4 | chr18 | 36333258 | rs6507354 | A/C |
| chr18 | 36822715 | 1 | 7 | chr18 | 36822713 | rs490355 | C/T |
| chr18 | 36931166 | 1 | 3 | chr18 | 36931164 | rs16974065 | C/T |
| chr18 | 38251580 | 2 | 5 | chr18 | 38251579 | rs417913 | A/G |
| chr18 | 39587130 | 2 | 4 | chr18 | 39587128 | rs7227216 | C/T |
| chr18 | 39587130 | 2 | 4 | chr18 | 39587129 | rs7227082 | A/G |

| | | | | | | | |
|---|---|---|---|---|---|---|---|
| chr18 | 40917406 | 2 | 6 | chr18 | 40917405 | rs4890511 | A/G |
| chr18 | 42584603 | 2 | 3 | chr18 | 42584601 | rs4624284 | C/T |
| chr18 | 43458873 | 4 | 2 | chr18 | 43458872 | rs9955855 | A/G |
| chr18 | 43931299 | 1 | 7 | chr18 | 43931298 | rs4289063 | A/G |
| chr18 | 43969554 | 3 | 4 | chr18 | 43969552 | rs11664767 | C/T |
| chr18 | 44190861 | 2 | 2 | chr18 | 44190859 | rs4471754 | C/G |
| chr18 | 44302501 | 2 | 6 | chr18 | 44302500 | rs1512180 | A/G |
| chr18 | 44412443 | 2 | 3 | chr18 | 44412441 | rs8091766 | C/T |
| chr18 | 44597817 | 8 | 1 | chr18 | 44597815 | rs9951895 | C/T |
| chr18 | 44629244 | 3 | 1 | chr18 | 44629244 | rs4939822 | C/G |
| chr18 | 44796788 | 2 | 7 | chr18 | 44796787 | rs357890 | G/T |
| chr18 | 45281903 | 1 | 6 | chr18 | 45281902 | rs8092007 | A/G |
| chr18 | 45447942 | 4 | 2 | chr18 | 45447940 | rs34770920 | C/G |
| chr18 | 46351920 | 6 | 1 | chr18 | 46351919 | rs1437654 | C/T |
| chr18 | 46976078 | 3 | 1 | chr18 | 46976077 | rs62092913 | A/G |
| chr18 | 47363146 | 5 | 3 | chr18 | 47363144 | rs728684 | C/T |
| chr18 | 47536968 | 2 | 4 | chr18 | 47536966 | rs323117 | A/C |
| chr18 | 47545065 | 2 | 2 | chr18 | 47545063 | rs323090 | A/G |
| chr18 | 47545065 | 2 | 2 | chr18 | 47545063 | rs74178642 | CA/TG |
| chr18 | 49438190 | 1 | 3 | chr18 | 49438190 | rs7228288 | A/G |
| chr18 | 50403687 | 4 | 1 | chr18 | 50403686 | rs4801120 | A/G |
| chr18 | 51329797 | 1 | 6 | chr18 | 51329795 | rs8090106 | C/T |
| chr18 | 51478781 | 3 | 4 | chr18 | 51478780 | rs694450 | A/G |
| chr18 | 51815581 | 3 | 1 | chr18 | 51815580 | rs2646953 | A/G |
| chr18 | 52198607 | 2 | 2 | chr18 | 52198605 | rs216573 | C/T |
| chr18 | 52871376 | 2 | 2 | chr18 | 52871374 | rs9945715 | C/T |
| chr18 | 53284832 | 3 | 2 | chr18 | 53284830 | rs580647 | A/G |
| chr18 | 53446688 | 1 | 4 | chr18 | 53446688 | rs523804 | A/G |
| chr18 | 54300028 | 1 | 3 | chr18 | 54300027 | rs3744867 | C/T |
| chr18 | 54318326 | 1 | 4 | chr18 | 54318325 | rs7242547 | A/G |
| chr18 | 54533637 | 2 | 4 | chr18 | 54533636 | rs7242509 | C/G/T |
| chr18 | 54595802 | 11 | 1 | chr18 | 54595800 | rs4536555 | C/T |

| | | | | | | | |
|---|---|---|---|---|---|---|---|
| chr18 | 55098164 | 1 | 4 | chr18 | 55098162 | rs670632 | A/C |
| chr18 | 55098247 | 5 | 12 | chr18 | 55098245 | rs510497 | C/T |
| chr18 | 55098402 | 6 | 2 | chr18 | 55098400 | rs9951712 | C/T |
| chr18 | 55397248 | 3 | 1 | chr18 | 55397246 | rs2564487 | A/G |
| chr18 | 56902715 | 3 | 1 | chr18 | 56902713 | rs171432 | A/G |
| chr18 | 57963255 | 5 | 2 | chr18 | 57963254 | rs12326173 | A/G |
| chr18 | 59003239 | 1 | 10 | chr18 | 59003237 | rs11661511 | C/T |
| chr18 | 59265055 | 1 | 7 | chr18 | 59265053 | rs12457701 | C/T |
| chr18 | 60343280 | 5 | 3 | chr18 | 60343279 | rs11152439 | A/G |
| chr18 | 61063749 | 2 | 7 | chr18 | 61063747 | rs488089 | A/C |
| chr18 | 62065418 | 3 | 1 | chr18 | 62065417 | rs7230005 | A/G |
| chr18 | 62389553 | 4 | 3 | chr18 | 62389552 | rs1943321 | C/T |
| chr18 | 63189470 | 3 | 1 | chr18 | 63189469 | rs7244066 | A/G |
| chr18 | 63344233 | 1 | 7 | chr18 | 63344232 | rs1351408 | A/G |
| chr18 | 63527582 | 3 | 2 | chr18 | 63527580 | rs2448731 | A/G |
| chr18 | 64321172 | 3 | 1 | chr18 | 64321171 | rs1400401 | C/T |
| chr18 | 65280929 | 1 | 3 | chr18 | 65280928 | rs8089373 | A/G |
| chr18 | 67638279 | 3 | 3 | chr18 | 67638278 | rs939720 | C/T |
| chr18 | 68518321 | 4 | 4 | chr18 | 68518320 | rs4455061 | A/G |
| chr18 | 69331047 | 2 | 4 | chr18 | 69331045 | rs2156546 | C/T |
| chr18 | 69559237 | 7 | 3 | chr18 | 69559235 | rs4559994 | C/T |
| chr18 | 69758990 | 1 | 4 | chr18 | 69758989 | rs9952074 | A/G |
| chr18 | 69761923 | 3 | 1 | chr18 | 69761922 | rs4552099 | A/G |
| chr18 | 69911427 | 1 | 3 | chr18 | 69911425 | rs9946681 | C/T |
| chr18 | 69938192 | 5 | 4 | chr18 | 69938191 | rs62097028 | A/G |
| chr18 | 70281412 | 8 | 2 | chr18 | 70281410 | rs9965729 | C/T |
| chr18 | 70351875 | 3 | 3 | chr18 | 70351874 | rs2081548 | C/T |
| chr18 | 70867844 | 3 | 1 | chr18 | 70867842 | rs11873473 | C/T |
| chr18 | 71027822 | 1 | 3 | chr18 | 71027820 | rs652450 | A/G |
| chr18 | 71227939 | 2 | 2 | chr18 | 71227936 | rs10514168 | A/C |
| chr18 | 71227939 | 2 | 2 | chr18 | 71227938 | rs17195541 | G/T |
| chr18 | 71335990 | 4 | 11 | chr18 | 71335988 | rs7242492 | C/T |

| | | | | | | | |
|---|---|---|---|---|---|---|---|
| chr18 | 71711830 | 1 | 5 | chr18 | 71711828 | rs12960586 | C/T |
| chr18 | 71849182 | 1 | 7 | chr18 | 71849181 | rs1627177 | C/T |
| chr18 | 72051749 | 1 | 4 | chr18 | 72051748 | rs3922825 | A/G |
| chr18 | 72218522 | 2 | 5 | chr18 | 72218520 | rs592921 | C/T |
| chr18 | 72420246 | 12 | 3 | chr18 | 72420246 | rs12959860 | A/G |
| chr18 | 72431400 | 3 | 1 | chr18 | 72431398 | rs12327122 | C/T |
| chr18 | 72500727 | 9 | 14 | chr18 | 72500725 | rs937903 | A/G |
| chr18 | 72634093 | 7 | 9 | chr18 | 72634091 | rs1320033 | A/G |
| chr18 | 72896010 | 1 | 3 | chr18 | 72896010 | rs9944542 | C/G |
| chr18 | 72938673 | 1 | 3 | chr18 | 72938672 | rs9958028 | A/G |
| chr18 | 73213331 | 4 | 1 | chr18 | 73213329 | rs1981395 | A/G |
| chr18 | 73471322 | 16 | 8 | chr18 | 73471320 | rs1472978 | C/T |
| chr18 | 73545469 | 1 | 3 | chr18 | 73545467 | rs1539993 | C/T |
| chr18 | 73555769 | 6 | 17 | chr18 | 73555768 | rs1503037 | C/T |
| chr18 | 73783349 | 3 | 1 | chr18 | 73783347 | rs8089795 | C/T |
| chr18 | 73783349 | 3 | 1 | chr18 | 73783348 | rs9957280 | A/G |
| chr18 | 74794851 | 1 | 5 | chr18 | 74794850 | rs2927251 | A/G |
| chr18 | 74804247 | 2 | 2 | chr18 | 74804246 | rs4799262 | A/G |
| chr18 | 74875373 | 0 | 0 | chr18 | 74875372 | rs36133186 | A/G |
| chr18 | 74876327 | 0 | 0 | chr18 | 74876326 | rs36173184 | A/G |
| chr18 | 74877450 | 0 | 0 | chr18 | 74877449 | rs71364777 | A/G |
| chr18 | 75262659 | 5 | 1 | chr18 | 75262658 | rs643705 | C/G |
| chr18 | 75469655 | 0 | 0 | chr18 | 75469654 | rs634644 | C/T |
| chr18 | 75519219 | 1 | 5 | chr18 | 75519217 | rs2364352 | A/G |
| chr18 | 75555225 | 0 | 0 | chr18 | 75555224 | rs7243340 | A/G |
| chr18 | 75616126 | 2 | 4 | chr18 | 75616125 | rs524643 | C/T |
| chr18 | 75631513 | 4 | 3 | chr18 | 75631512 | rs484601 | C/T |
| chr18 | 75640849 | 2 | 4 | chr18 | 75640848 | rs505932 | C/G |
| chr18 | 75695226 | 6 | 2 | chr18 | 75695225 | rs484191 | A/G |
| chr18 | 75695226 | 6 | 2 | chr18 | 75695226 | rs60865767 | A/G |
| chr18 | 75721899 | 4 | 4 | chr18 | 75721898 | rs28865701 | A/G |
| chr18 | 75761850 | 4 | 2 | chr18 | 75761850 | rs1122941 | A/G |

| | | | | | | | |
|---|---|---|---|---|---|---|---|
| chr18 | 75939253 | 2 | 10 | chr18 | 75939251 | rs1240410 | A/G |
| chr18 | 75970952 | 2 | 3 | chr18 | 75970951 | rs1914968 | A/G |
| chr19 | 292894 | 24 | 4 | chr19 | 292893 | rs8101526 | A/G |
| chr19 | 319812 | 1 | 4 | chr19 | 319811 | rs3795000 | A/G |
| chr19 | 382317 | 0 | 0 | chr19 | 382315 | rs34296370 | C/G |
| chr19 | 382595 | 0 | 0 | chr19 | 382594 | rs12986237 | A/G |
| chr19 | 382827 | 0 | 0 | chr19 | 382826 | rs11671798 | A/G |
| chr19 | 383055 | 0 | 0 | chr19 | 383053 | rs34965458 | C/G |
| chr19 | 383113 | 0 | 0 | chr19 | 383111 | rs12460247 | C/G |
| chr19 | 383113 | 0 | 0 | chr19 | 383112 | rs12462858 | A/G |
| chr19 | 383809 | 0 | 0 | chr19 | 383807 | rs57903730 | C/G |
| chr19 | 383979 | 0 | 0 | chr19 | 383978 | rs61240939 | A/G |
| chr19 | 384957 | 0 | 0 | chr19 | 384955 | rs35273652 | C/G |
| chr19 | 385015 | 0 | 0 | chr19 | 385013 | rs34374147 | C/G |
| chr19 | 449213 | 7 | 5 | chr19 | 449211 | rs12461640 | A/C |
| chr19 | 487682 | 2 | 6 | chr19 | 487680 | rs1108113 | C/T |
| chr19 | 609368 | 23 | 2 | chr19 | 609367 | rs2040739 | -/A/G |
| chr19 | 609368 | 23 | 2 | chr19 | 609369 | rs66602400 | -/G |
| chr19 | 646468 | 5 | 2 | chr19 | 646465 | rs72978241 | C/T |
| chr19 | 647906 | 0 | 0 | chr19 | 647904 | rs12975365 | C/T |
| chr19 | 687447 | 1 | 9 | chr19 | 687446 | rs9304945 | A/G |
| chr19 | 874744 | 0 | 0 | chr19 | 874743 | rs350134 | A/G |
| chr19 | 880753 | 5 | 1 | chr19 | 880752 | rs1799595 | A/G |
| chr19 | 905702 | 2 | 5 | chr19 | 905700 | rs349311 | C/T |
| chr19 | 949799 | 5 | 2 | chr19 | 949797 | rs11669927 | C/T |
| chr19 | 979293 | 6 | 4 | chr19 | 979290 | rs62131169 | C/G |
| chr19 | 991765 | 3 | 4 | chr19 | 991764 | rs4147904 | A/G |
| chr19 | 1060908 | 1 | 3 | chr19 | 1060906 | rs56248610 | C/T |
| chr19 | 1277123 | 6 | 1 | chr19 | 1277121 | rs4807081 | C/T |
| chr19 | 1338300 | 13 | 3 | chr19 | 1338298 | rs502121 | A/G |
| chr19 | 1405043 | 1 | 5 | chr19 | 1405041 | rs791459 | C/T |
| chr19 | 1760837 | 3 | 2 | chr19 | 1760835 | rs8107742 | C/T |

| | | | | | | | | |
|---|---|---|---|---|---|---|---|---|
| chr19 | 1802084 | 5 | 1 | chr19 | 1802083 | rs4807152 | | A/G |
| chr19 | 1869024 | 4 | 3 | chr19 | 1869022 | rs10415845 | | C/T |
| chr19 | 1895798 | 4 | 2 | chr19 | 1895796 | rs12608688 | | C/G |
| chr19 | 1898506 | 2 | 3 | chr19 | 1898505 | rs1000845 | | C/T |
| chr19 | 2256592 | 6 | 5 | chr19 | 2256591 | rs7255529 | | A/G |
| chr19 | 2354563 | 4 | 2 | chr19 | 2354563 | rs7248779 | | G/T |
| chr19 | 2426290 | 2 | 4 | chr19 | 2426288 | rs3783492 | | A/C |
| chr19 | 2762832 | 3 | 1 | chr19 | 2762830 | rs8109055 | | C/T |
| chr19 | 2835517 | 3 | 2 | chr19 | 2835515 | rs2058324 | | C/T |
| chr19 | 2851349 | 5 | 1 | chr19 | 2851347 | rs11084969 | | A/C |
| chr19 | 2887443 | 1 | 7 | chr19 | 2887442 | rs7251272 | | A/G |
| chr19 | 3035945 | 0 | 0 | chr19 | 3035943 | rs11880963 | | C/T |
| chr19 | 3061349 | 2 | 6 | chr19 | 3061348 | rs11085000 | | G/T |
| chr19 | 3071344 | 4 | 1 | chr19 | 3071341 | rs372774 | | C/T |
| chr19 | 3071344 | 4 | 1 | chr19 | 3071342 | rs11670197 | | C/G |
| chr19 | 3316583 | 4 | 3 | chr19 | 3316582 | rs3810420 | | A/G |
| chr19 | 3376400 | 5 | 1 | chr19 | 3376399 | rs6510750 | | A/G |
| chr19 | 3502970 | 2 | 5 | chr19 | 3502968 | rs1715094 | | A/G |
| chr19 | 3595510 | 1 | 7 | chr19 | 3595508 | rs4807495 | | C/T |
| chr19 | 3600929 | 1 | 3 | chr19 | 3600927 | rs12978568 | | C/T |
| chr19 | 3909397 | 5 | 1 | chr19 | 3909396 | rs7255123 | | A/G |
| chr19 | 4007347 | 3 | 3 | chr19 | 4007345 | rs10409301 | | C/T |
| chr19 | 4321005 | 6 | 10 | chr19 | 4321003 | rs459906 | | C/T |
| chr19 | 4459945 | 5 | 8 | chr19 | 4459944 | rs8105775 | | A/G |
| chr19 | 4462956 | 6 | 2 | chr19 | 4462954 | rs7260518 | | C/T |
| chr19 | 4829582 | 7 | 1 | chr19 | 4829579 | rs2656941 | | A/G |
| chr19 | 4932096 | 1 | 14 | chr19 | 4932096 | rs197161 | | C/G |
| chr19 | 4982042 | 0 | 0 | chr19 | 4982040 | rs4807680 | | C/T |
| chr19 | 5166938 | 2 | 3 | chr19 | 5166937 | rs10409567 | | A/G |
| chr19 | 5541927 | 2 | 2 | chr19 | 5541926 | rs4807793 | | A/G |
| chr19 | 5734635 | 2 | 3 | chr19 | 5734633 | rs2446210 | | C/T |
| chr19 | 5765677 | 1 | 8 | chr19 | 5765675 | rs2446203 | | A/G |

| | | | | | | | |
|---|---|---|---|---|---|---|---|
| chr19 | 6365646 | 4 | 2 | chr19 | 6365645 | rs1654586 | C/T |
| chr19 | 6417482 | 4 | 6 | chr19 | 6417481 | rs348362 | A/G |
| chr19 | 7051016 | 1 | 3 | chr19 | 7051015 | rs2914562 | A/C |
| chr19 | 7245359 | 3 | 2 | chr19 | 7245357 | rs2860190 | C/G |
| chr19 | 7540594 | 3 | 1 | chr19 | 7540593 | rs7250311 | G/T |
| chr19 | 7869948 | 2 | 2 | chr19 | 7869947 | rs3745379 | A/G |
| chr19 | 7869948 | 2 | 2 | chr19 | 7869948 | rs2042919 | C/T |
| chr19 | 7882627 | 2 | 2 | chr19 | 7882626 | rs480296 | C/T |
| chr19 | 7948431 | 10 | 1 | chr19 | 7948430 | rs3786619 | A/G |
| chr19 | 7988535 | 1 | 4 | chr19 | 7988535 | rs12980552 | A/G |
| chr19 | 8015011 | 1 | 5 | chr19 | 8015009 | rs34944501 | C/T |
| chr19 | 8311713 | 3 | 4 | chr19 | 8311712 | rs2913963 | A/G |
| chr19 | 8464243 | 7 | 1 | chr19 | 8464241 | rs6422427 | C/T |
| chr19 | 8499723 | 3 | 6 | chr19 | 8499721 | rs2967590 | C/G |
| chr19 | 8837713 | 3 | 1 | chr19 | 8837712 | rs3764552 | C/T |
| chr19 | 8979503 | 3 | 1 | chr19 | 8979502 | rs1469081 | C/T |
| chr19 | 9860828 | 7 | 1 | chr19 | 9860826 | rs35833111 | C/T |
| chr19 | 10529384 | 6 | 1 | chr19 | 10529382 | rs3745256 | A/G |
| chr19 | 10928835 | 2 | 2 | chr19 | 10928834 | rs6511716 | A/G |
| chr19 | 11187126 | 0 | 0 | chr19 | 11187124 | rs2304154 | A/G |
| chr19 | 11241295 | 2 | 5 | chr19 | 11241294 | rs4804159 | A/G |
| chr19 | 12635210 | 1 | 5 | chr19 | 12635207 | rs1054486 | C/G |
| chr19 | 12896637 | 3 | 3 | chr19 | 12896637 | rs8107173 | A/G |
| chr19 | 13124511 | 3 | 8 | chr19 | 13124509 | rs3826913 | G/T |
| chr19 | 13229032 | 1 | 8 | chr19 | 13229030 | rs4926242 | C/T |
| chr19 | 13474661 | 2 | 2 | chr19 | 13474660 | rs2900966 | A/G |
| chr19 | 14245737 | 3 | 3 | chr19 | 14245735 | rs498284 | A/G |
| chr19 | 14507835 | 4 | 8 | chr19 | 14507834 | rs73002839 | A/G |
| chr19 | 14517648 | 2 | 4 | chr19 | 14517646 | rs8110587 | C/T |
| chr19 | 14729006 | 2 | 2 | chr19 | 14729004 | rs12327671 | C/T |
| chr19 | 14929734 | 2 | 2 | chr19 | 14929732 | rs1106738 | C/T |
| chr19 | 15692278 | 2 | 2 | chr19 | 15692277 | rs10854150 | C/G |

| | | | | | | | |
|---|---|---|---|---|---|---|---|
| chr19 | 15755515 | 2 | 3 | chr19 | 15755513 | rs4808377 | A/C |
| chr19 | 15893697 | 1 | 3 | chr19 | 15893696 | rs11086013 | G/T |
| chr19 | 16181075 | 2 | 4 | chr19 | 16181074 | rs2290802 | C/G |
| chr19 | 16297263 | 3 | 1 | chr19 | 16297261 | rs3745318 | C/T |
| chr19 | 16472979 | 6 | 4 | chr19 | 16472977 | rs728116 | C/T |
| chr19 | 16916405 | 3 | 3 | chr19 | 16916403 | rs10403452 | C/T |
| chr19 | 17072569 | 1 | 9 | chr19 | 17072567 | rs10423719 | C/T |
| chr19 | 17178252 | 12 | 1 | chr19 | 17178251 | rs2279002 | C/T |
| chr19 | 17494933 | 1 | 5 | chr19 | 17494932 | rs2375592 | A/G |
| chr19 | 17598435 | 4 | 4 | chr19 | 17598434 | rs12610816 | A/G |
| chr19 | 17725353 | 4 | 3 | chr19 | 17725353 | rs4808684 | A/G |
| chr19 | 17956770 | 2 | 3 | chr19 | 17956768 | rs2278992 | C/T |
| chr19 | 18041413 | 4 | 3 | chr19 | 18041412 | rs401502 | C/G |
| chr19 | 18096425 | 1 | 3 | chr19 | 18096423 | rs123203 | C/T |
| chr19 | 18219783 | 3 | 2 | chr19 | 18219782 | rs4808122 | A/G |
| chr19 | 18706786 | 3 | 3 | chr19 | 18706784 | rs57962499 | C/T |
| chr19 | 18747432 | 4 | 3 | chr19 | 18747430 | rs12609934 | C/T |
| chr19 | 18754811 | 5 | 2 | chr19 | 18754810 | rs28494505 | A/G |
| chr19 | 18912900 | 1 | 3 | chr19 | 18912897 | rs11540740 | A/G |
| chr19 | 19095426 | 1 | 4 | chr19 | 19095425 | rs10421019 | A/G |
| chr19 | 19358668 | 2 | 4 | chr19 | 19358668 | rs1858999 | C/G |
| chr19 | 19748579 | 3 | 3 | chr19 | 19748577 | rs248945 | G/T |
| chr19 | 19928752 | 2 | 3 | chr19 | 19928751 | rs7245577 | G/T |
| chr19 | 20750959 | 2 | 4 | chr19 | 20750958 | rs8106412 | G/T |
| chr19 | 22589474 | 3 | 2 | chr19 | 22589473 | rs62121329 | A/G |
| chr19 | 23337090 | 5 | 11 | chr19 | 23337089 | rs1044095 | C/T |
| chr19 | 24023001 | 2 | 4 | chr19 | 24022999 | rs73520396 | C/T |
| chr19 | 32432061 | 2 | 8 | chr19 | 32432058 | rs62133478 | C/T |
| chr19 | 32844995 | 4 | 3 | chr19 | 32844993 | rs73014677 | A/C |
| chr19 | 32860364 | 2 | 2 | chr19 | 32860362 | rs758764 | C/T |
| chr19 | 33060136 | 3 | 1 | chr19 | 33060133 | rs4804967 | C/T |
| chr19 | 33060136 | 3 | 1 | chr19 | 33060136 | rs4804968 | A/G |

| | | | | | | | |
|---|---|---|---|---|---|---|---|
| chr19 | 33073602 | 0 | 0 | chr19 | 33073601 | rs12971524 | A/G |
| chr19 | 33557210 | 11 | 2 | chr19 | 33557208 | rs8113667 | C/T |
| chr19 | 33574805 | 2 | 2 | chr19 | 33574804 | rs10853718 | C/G |
| chr19 | 33998524 | 7 | 2 | chr19 | 33998523 | rs3940362 | A/G |
| chr19 | 34001435 | 5 | 3 | chr19 | 34001433 | rs1473188 | A/G |
| chr19 | 34071727 | 0 | 0 | chr19 | 34071726 | rs6509105 | A/G |
| chr19 | 34907181 | 6 | 5 | chr19 | 34907181 | rs2866312 | A/G |
| chr19 | 35032252 | 3 | 1 | chr19 | 35032251 | rs929814 | A/G |
| chr19 | 35305536 | 1 | 5 | chr19 | 35305534 | rs335771 | C/T |
| chr19 | 35412029 | 3 | 2 | chr19 | 35412027 | rs73019701 | A/C |
| chr19 | 35802942 | 9 | 6 | chr19 | 35802940 | rs2217660 | A/G |
| chr19 | 36045453 | 10 | 1 | chr19 | 36045452 | rs4804943 | A/G |
| chr19 | 36521745 | 2 | 3 | chr19 | 36521742 | rs924150 | A/C |
| chr19 | 36565623 | 2 | 9 | chr19 | 36565622 | rs748888 | C/T |
| chr19 | 37141121 | 14 | 6 | chr19 | 37141119 | rs55634041 | C/T |
| chr19 | 37182777 | 1 | 5 | chr19 | 37182775 | rs1073177 | C/T |
| chr19 | 37325385 | 5 | 4 | chr19 | 37325384 | rs995483 | A/G |
| chr19 | 38424804 | 2 | 5 | chr19 | 38424803 | rs889098 | C/T |
| chr19 | 38766646 | 5 | 3 | chr19 | 38766645 | rs8103090 | G/T |
| chr19 | 38940732 | 3 | 3 | chr19 | 38940730 | rs35846322 | -/C |
| chr19 | 38940732 | 3 | 3 | chr19 | 38940731 | rs461921 | A/G |
| chr19 | 38979882 | 1 | 4 | chr19 | 38979880 | rs7259858 | C/G |
| chr19 | 39002640 | 4 | 7 | chr19 | 39002639 | rs29939 | A/G |
| chr19 | 39607292 | 2 | 10 | chr19 | 39607290 | rs10853936 | C/T |
| chr19 | 39998397 | 1 | 3 | chr19 | 39998395 | rs929773 | C/T |
| chr19 | 40302800 | 10 | 9 | chr19 | 40302799 | rs10414555 | G/T |
| chr19 | 40303753 | 1 | 6 | chr19 | 40303752 | rs1688015 | A/G |
| chr19 | 40576864 | 3 | 1 | chr19 | 40576862 | rs12980912 | C/T |
| chr19 | 40922607 | 3 | 2 | chr19 | 40922606 | rs34562867 | C/T |
| chr19 | 40968607 | 1 | 4 | chr19 | 40968606 | rs120960 | A/G |
| chr19 | 42489102 | 2 | 4 | chr19 | 42489100 | rs10418740 | A/C |
| chr19 | 43246203 | 5 | 1 | chr19 | 43246203 | rs1626441 | C/G |

| | | | | | | | |
|---|---|---|---|---|---|---|---|
| chr19 | 43844749 | 6 | 2 | chr19 | 43844747 | rs12984247 | C/T |
| chr19 | 44185321 | 3 | 7 | chr19 | 44185320 | rs646580 | A/G |
| chr19 | 44310455 | 3 | 1 | chr19 | 44310452 | rs477489 | C/G |
| chr19 | 44310455 | 3 | 1 | chr19 | 44310454 | rs9967595 | G/T |
| chr19 | 44318205 | 2 | 6 | chr19 | 44318204 | rs569287 | A/G |
| chr19 | 44365874 | 2 | 5 | chr19 | 44365873 | rs17641276 | A/G |
| chr19 | 44715147 | 4 | 1 | chr19 | 44715147 | rs1123301 | A/G |
| chr19 | 45393111 | 3 | 2 | chr19 | 45393109 | rs73042673 | A/C |
| chr19 | 45478364 | 3 | 3 | chr19 | 45478362 | rs8102171 | A/C |
| chr19 | 45517964 | 4 | 4 | chr19 | 45517963 | rs890773 | C/T |
| chr19 | 45592706 | 0 | 0 | chr19 | 45592704 | rs268674 | A/G |
| chr19 | 45612936 | 5 | 1 | chr19 | 45612935 | rs268664 | C/T |
| chr19 | 45623706 | 5 | 3 | chr19 | 45623704 | rs268683 | C/T |
| chr19 | 45880931 | 1 | 3 | chr19 | 45880930 | rs2561542 | C/T |
| chr19 | 46239930 | 2 | 6 | chr19 | 46239929 | rs11672352 | A/G |
| chr19 | 46359844 | 5 | 3 | chr19 | 46359842 | rs1645685 | C/T |
| chr19 | 46891222 | 9 | 2 | chr19 | 46891220 | rs4803501 | C/T |
| chr19 | 48695549 | 7 | 1 | chr19 | 48695547 | rs2682551 | C/T |
| chr19 | 48875540 | 3 | 3 | chr19 | 48875537 | rs10403299 | C/G |
| chr19 | 48875540 | 3 | 3 | chr19 | 48875537 | rs34552101 | CC/GT |
| chr19 | 48994905 | 1 | 4 | chr19 | 48994905 | rs11547806 | C/G |
| chr19 | 49716421 | 2 | 4 | chr19 | 49716420 | rs12982449 | C/G |
| chr19 | 50180274 | 2 | 2 | chr19 | 50180273 | rs204472 | A/G |
| chr19 | 50286010 | 4 | 9 | chr19 | 50286009 | rs3178166 | A/G |
| chr19 | 50724682 | 5 | 6 | chr19 | 50724680 | rs12971437 | C/G |
| chr19 | 51048847 | 2 | 4 | chr19 | 51048846 | rs11665742 | A/G |
| chr19 | 51107182 | 3 | 4 | chr19 | 51107182 | rs9967621 | G/T |
| chr19 | 51176663 | 3 | 8 | chr19 | 51176661 | rs8104599 | A/C |
| chr19 | 51211866 | 9 | 4 | chr19 | 51211865 | rs2287306 | A/G |
| chr19 | 51361004 | 6 | 1 | chr19 | 51361002 | rs10420052 | C/T |
| chr19 | 51546818 | 5 | 4 | chr19 | 51546817 | rs759302 | A/G |
| chr19 | 51632876 | 2 | 5 | chr19 | 51632874 | rs35477266 | C/G |

| | | | | | | | |
|---|---|---|---|---|---|---|---|
| chr19 | 51642887 | 2 | 5 | chr19 | 51642886 | rs2311216 | A/G |
| chr19 | 51800024 | 2 | 5 | chr19 | 51800022 | rs56765950 | C/T |
| chr19 | 51806013 | 11 | 3 | chr19 | 51806012 | rs10413370 | A/G |
| chr19 | 52055231 | 3 | 3 | chr19 | 52055230 | rs311358 | A/G |
| chr19 | 52519842 | 0 | 0 | chr19 | 52519841 | rs4802340 | A/G |
| chr19 | 52568841 | 0 | 0 | chr19 | 52568840 | rs2547380 | C/T |
| chr19 | 52959391 | 4 | 4 | chr19 | 52959389 | rs7249470 | C/T |
| chr19 | 53161592 | 3 | 4 | chr19 | 53161590 | rs10418675 | C/T |
| chr19 | 53388749 | 3 | 4 | chr19 | 53388746 | rs7359970 | C/G |
| chr19 | 53491528 | 5 | 1 | chr19 | 53491527 | rs2292112 | A/G |
| chr19 | 53909333 | 4 | 3 | chr19 | 53909331 | rs281387 | C/T |
| chr19 | 54117585 | 6 | 6 | chr19 | 54117583 | rs1532704 | A/G |
| chr19 | 54119966 | 8 | 9 | chr19 | 54119965 | rs4802524 | A/G |
| chr19 | 54523060 | 1 | 3 | chr19 | 54523057 | rs181344 | C/T |
| chr19 | 54788233 | 3 | 9 | chr19 | 54788232 | rs10404887 | C/G |
| chr19 | 54973350 | 1 | 14 | chr19 | 54973348 | rs1273644 | C/T |
| chr19 | 55075402 | 4 | 2 | chr19 | 55075402 | rs8109661 | A/G |
| chr19 | 55419497 | 3 | 1 | chr19 | 55419496 | rs4538112 | A/G |
| chr19 | 55429458 | 3 | 2 | chr19 | 55429457 | rs2892340 | C/T |
| chr19 | 55573139 | 4 | 2 | chr19 | 55573137 | rs28514894 | C/T |
| chr19 | 55712715 | 3 | 1 | chr19 | 55712714 | rs61751956 | A/G |
| chr19 | 56029045 | 2 | 3 | chr19 | 56029043 | rs2659055 | A/G |
| chr19 | 56052580 | 3 | 1 | chr19 | 56052579 | rs266877 | A/G |
| chr19 | 56117427 | 6 | 2 | chr19 | 56117425 | rs7255201 | C/G |
| chr19 | 56351526 | 2 | 2 | chr19 | 56351525 | rs273665 | A/G |
| chr19 | 56489069 | 0 | 0 | chr19 | 56489068 | rs10420757 | C/G |
| chr19 | 56624370 | 2 | 4 | chr19 | 56624369 | rs2411318 | C/G |
| chr19 | 56852119 | 7 | 3 | chr19 | 56852116 | rs7260152 | C/T |
| chr19 | 57366690 | 4 | 2 | chr19 | 57366688 | rs10420136 | C/G/T |
| chr19 | 58279780 | 1 | 4 | chr19 | 58279780 | rs2560953 | C/G |
| chr19 | 58736554 | 3 | 1 | chr19 | 58736552 | rs60999701 | C/T |
| chr19 | 59216648 | 5 | 1 | chr19 | 59216647 | rs2431227 | A/G |

| | | | | | | | |
|---|---|---|---|---|---|---|---|
| chr19 | 59381136 | 7 | 1 | chr19 | 59381134 | rs254281 | C/G |
| chr19 | 59400242 | 5 | 2 | chr19 | 59400241 | rs3852889 | C/T |
| chr19 | 60230884 | 1 | 4 | chr19 | 60230883 | rs892090 | G/T |
| chr19 | 60260048 | 3 | 1 | chr19 | 60260047 | rs73615874 | A/G |
| chr19 | 60270250 | 2 | 4 | chr19 | 60270248 | rs1654465 | C/T |
| chr19 | 60564343 | 2 | 2 | chr19 | 60564342 | rs11084397 | C/G |
| chr19 | 60740210 | 5 | 1 | chr19 | 60740208 | rs12979821 | C/T |
| chr19 | 60782306 | 5 | 3 | chr19 | 60782304 | rs12974617 | A/C |
| chr19 | 61142345 | 4 | 1 | chr19 | 61142343 | rs9636113 | C/T |
| chr19 | 61191157 | 1 | 4 | chr19 | 61191156 | rs306455 | C/T |
| chr19 | 61326830 | 5 | 3 | chr19 | 61326830 | rs583330 | C/T |
| chr19 | 61662562 | 1 | 4 | chr19 | 61662561 | rs4801321 | A/G |
| chr19 | 62078358 | 3 | 1 | chr19 | 62078357 | rs10420571 | A/G |
| chr19 | 62983040 | 3 | 2 | chr19 | 62983039 | rs2158013 | A/G |
| chr19 | 63172292 | 3 | 3 | chr19 | 63172290 | rs257674 | C/T |
| chr19 | 63260771 | 13 | 4 | chr19 | 63260769 | rs11667482 | C/T |
| chr19 | 63407231 | 4 | 2 | chr19 | 63407229 | rs12982863 | C/G |
| chr20 | 491287 | 2 | 4 | chr20 | 491285 | rs204691 | C/T |
| chr20 | 553937 | 1 | 5 | chr20 | 553936 | rs4813760 | A/G |
| chr20 | 577214 | 3 | 3 | chr20 | 577212 | rs3746799 | C/T |
| chr20 | 613378 | 2 | 2 | chr20 | 613376 | rs6107763 | C/T |
| chr20 | 613378 | 2 | 2 | chr20 | 613377 | rs4813791 | A/G |
| chr20 | 1914812 | 3 | 4 | chr20 | 1914810 | rs1418037 | C/T |
| chr20 | 3001498 | 12 | 3 | chr20 | 3001495 | rs2740209 | C/G |
| chr20 | 3138038 | 8 | 1 | chr20 | 3138038 | rs45620433 | C/G |
| chr20 | 3148826 | 1 | 3 | chr20 | 3148824 | rs6107257 | C/T |
| chr20 | 3155749 | 1 | 7 | chr20 | 3155748 | rs3810559 | G/T |
| chr20 | 3608790 | 2 | 7 | chr20 | 3608788 | rs603112 | A/G |
| chr20 | 4172659 | 3 | 1 | chr20 | 4172657 | rs2875939 | A/C |
| chr20 | 4263533 | 3 | 2 | chr20 | 4263531 | rs4815689 | C/T |
| chr20 | 4716137 | 3 | 5 | chr20 | 4716135 | rs55900827 | C/T |
| chr20 | 4799901 | 3 | 1 | chr20 | 4799900 | rs3787456 | A/G |

| | | | | | | | |
|---|---|---|---|---|---|---|---|
| chr20 | 5251316 | 3 | 3 | chr20 | 5251314 | rs7264622 | A/C |
| chr20 | 6721674 | 3 | 1 | chr20 | 6721672 | rs235703 | A/G |
| chr20 | 7224552 | 1 | 12 | chr20 | 7224550 | rs62195419 | C/T |
| chr20 | 8060498 | 2 | 4 | chr20 | 8060496 | rs6086348 | C/T |
| chr20 | 9566761 | 4 | 1 | chr20 | 9566760 | rs6516479 | A/G |
| chr20 | 10117642 | 2 | 2 | chr20 | 10117642 | rs2327262 | C/G |
| chr20 | 10125651 | 9 | 1 | chr20 | 10125650 | rs6133832 | A/G |
| chr20 | 10146956 | 2 | 2 | chr20 | 10146953 | rs6039769 | A/C |
| chr20 | 10585058 | 2 | 3 | chr20 | 10585055 | rs6077865 | A/C |
| chr20 | 10585058 | 2 | 3 | chr20 | 10585056 | rs10485741 | C/T |
| chr20 | 10595951 | 7 | 8 | chr20 | 10595950 | rs3790163 | A/G |
| chr20 | 11106077 | 4 | 2 | chr20 | 11106077 | rs2064525 | C/T |
| chr20 | 11652692 | 3 | 1 | chr20 | 11652690 | rs6078300 | C/T |
| chr20 | 12317521 | 1 | 8 | chr20 | 12317520 | rs6041303 | A/G |
| chr20 | 13206148 | 1 | 4 | chr20 | 13206147 | rs11698516 | A/G |
| chr20 | 14533402 | 3 | 1 | chr20 | 14533401 | rs11087098 | A/G |
| chr20 | 14763876 | 4 | 1 | chr20 | 14763874 | rs407619 | A/G |
| chr20 | 15582854 | 9 | 2 | chr20 | 15582853 | rs462849 | G/T |
| chr20 | 16157524 | 1 | 5 | chr20 | 16157522 | rs62198067 | A/C |
| chr20 | 16482461 | 2 | 2 | chr20 | 16482460 | rs6080300 | A/G |
| chr20 | 17097677 | 3 | 1 | chr20 | 17097675 | rs852094 | C/T |
| chr20 | 17427764 | 2 | 5 | chr20 | 17427762 | rs2235585 | C/T |
| chr20 | 17547215 | 4 | 1 | chr20 | 17547213 | rs2328181 | C/T |
| chr20 | 17576584 | 3 | 6 | chr20 | 17576584 | rs6080760 | A/G |
| chr20 | 18369988 | 1 | 7 | chr20 | 18369987 | rs6045400 | A/G |
| chr20 | 19267845 | 9 | 3 | chr20 | 19267845 | rs4814838 | A/G |
| chr20 | 19686582 | 3 | 4 | chr20 | 19686580 | rs708993 | A/C |
| chr20 | 20259339 | 4 | 2 | chr20 | 20259338 | rs6075645 | A/G |
| chr20 | 20774745 | 5 | 1 | chr20 | 20774743 | rs4813397 | C/T |
| chr20 | 20965279 | 8 | 3 | chr20 | 20965278 | rs4599194 | A/G |
| chr20 | 21051987 | 4 | 1 | chr20 | 21051986 | rs1007226 | A/G |
| chr20 | 21877058 | 8 | 1 | chr20 | 21877056 | rs2424410 | A/C |

| | | | | | | | |
|---|---|---|---|---|---|---|---|
| chr20 | 21915950 | 2 | 4 | chr20 | 21915949 | rs201546 | A/G |
| chr20 | 22141951 | 4 | 1 | chr20 | 22141950 | rs804608 | A/G |
| chr20 | 22270362 | 4 | 1 | chr20 | 22270362 | rs115282 | C/G |
| chr20 | 22432076 | 1 | 12 | chr20 | 22432075 | rs6048135 | A/G |
| chr20 | 22961557 | 1 | 4 | chr20 | 22961556 | rs191987 | A/G |
| chr20 | 23036596 | 2 | 3 | chr20 | 23036594 | rs6137830 | C/T |
| chr20 | 23365705 | 3 | 11 | chr20 | 23365704 | rs2424555 | A/G |
| chr20 | 23404588 | 5 | 1 | chr20 | 23404588 | rs1028522 | A/C |
| chr20 | 23841680 | 3 | 1 | chr20 | 23841679 | rs2995110 | C/T |
| chr20 | 24143469 | 4 | 2 | chr20 | 24143468 | rs492884 | C/T |
| chr20 | 24660083 | 1 | 3 | chr20 | 24660081 | rs4815302 | C/G |
| chr20 | 24699287 | 4 | 3 | chr20 | 24699286 | rs6138372 | G/T |
| chr20 | 24947795 | 1 | 3 | chr20 | 24947794 | rs3746330 | A/G |
| chr20 | 25134502 | 4 | 2 | chr20 | 25134501 | rs3787075 | C/G |
| chr20 | 25142768 | 1 | 5 | chr20 | 25142767 | rs6083780 | A/G |
| chr20 | 25207910 | 2 | 6 | chr20 | 25207909 | rs2261698 | A/G |
| chr20 | 25298222 | 3 | 1 | chr20 | 25298222 | rs4815411 | A/G |
| chr20 | 25713694 | 4 | 2 | chr20 | 25713692 | rs62212611 | C/T |
| chr20 | 25780184 | 1 | 4 | chr20 | 25780183 | rs6050929 | A/G |
| chr20 | 25999484 | 2 | 4 | chr20 | 25999483 | rs62212611 | C/T |
| chr20 | 28223899 | 1 | 3 | chr20 | 28223896 | rs73616359 | C/G |
| chr20 | 28243658 | 10 | 20 | chr20 | 28243656 | rs34419651 | A/G |
| chr20 | 28243658 | 10 | 20 | chr20 | 28243656 | rs60541032 | C/T |
| chr20 | 28243658 | 10 | 20 | chr20 | 28243657 | rs1872909 | C/T |
| chr20 | 28243658 | 10 | 20 | chr20 | 28243657 | rs61115086 | A/G |
| chr20 | 28248711 | 5 | 3 | chr20 | 28248710 | rs62196380 | A/G |
| chr20 | 28248711 | 5 | 3 | chr20 | 28248710 | rs68075917 | CAGTTAGGAACC/TGGTTAGGAACT |
| chr20 | 29548077 | 1 | 3 | chr20 | 29548075 | rs6119442 | C/T |
| chr20 | 29658559 | 3 | 2 | chr20 | 29658559 | rs6058197 | A/G |
| chr20 | 32382880 | 10 | 2 | chr20 | 32382879 | rs1890002 | A/G |
| chr20 | 32430976 | 1 | 6 | chr20 | 32430974 | rs6141465 | C/T |
| chr20 | 32631010 | 2 | 4 | chr20 | 32631009 | rs6059928 | A/G |

| | | | | | | | |
|---|---|---|---|---|---|---|---|
| chr20 | 34979792 | 3 | 3 | chr20 | 34979790 | rs1935919 | A/G |
| chr20 | 35319955 | 5 | 3 | chr20 | 35319953 | rs6017655 | C/T |
| chr20 | 35458643 | 1 | 5 | chr20 | 35458643 | rs6018273 | A/G |
| chr20 | 35501287 | 0 | 0 | chr20 | 35501286 | rs6012200 | A/G |
| chr20 | 35556972 | 3 | 14 | chr20 | 35556970 | rs2425342 | C/T |
| chr20 | 35684349 | 6 | 1 | chr20 | 35684348 | rs4441534 | A/G |
| chr20 | 35960063 | 2 | 12 | chr20 | 35960062 | rs4809891 | A/G |
| chr20 | 36026505 | 1 | 3 | chr20 | 36026504 | rs6126657 | A/G |
| chr20 | 36228378 | 4 | 5 | chr20 | 36228376 | rs7272112 | C/T |
| chr20 | 36381716 | 2 | 4 | chr20 | 36381715 | rs6024842 | A/G |
| chr20 | 37524662 | 4 | 8 | chr20 | 37524660 | rs1883517 | C/T |
| chr20 | 37725830 | 6 | 1 | chr20 | 37725828 | rs6028593 | C/T |
| chr20 | 38959464 | 2 | 4 | chr20 | 38959463 | rs2425452 | A/G |
| chr20 | 39856714 | 2 | 2 | chr20 | 39856712 | rs2208524 | C/T |
| chr20 | 40314585 | 14 | 1 | chr20 | 40314583 | rs6072670 | C/T |
| chr20 | 40472556 | 3 | 1 | chr20 | 40472555 | rs35324452 | A/G |
| chr20 | 40502875 | 6 | 2 | chr20 | 40502873 | rs2179218 | C/T |
| chr20 | 41862555 | 15 | 9 | chr20 | 41862554 | rs244064 | A/G |
| chr20 | 41928752 | 1 | 4 | chr20 | 41928751 | rs6031184 | A/G |
| chr20 | 41986656 | 2 | 8 | chr20 | 41986654 | rs11086906 | C/T |
| chr20 | 41999991 | 1 | 5 | chr20 | 41999990 | rs6093898 | A/G |
| chr20 | 42244657 | 4 | 1 | chr20 | 42244655 | rs6130559 | C/G |
| chr20 | 42709534 | 2 | 11 | chr20 | 42709533 | rs2007515 | A/C |
| chr20 | 42745812 | 4 | 7 | chr20 | 42745810 | rs912914 | C/T |
| chr20 | 42791313 | 1 | 5 | chr20 | 42791312 | rs11698203 | A/G |
| chr20 | 43380183 | 2 | 2 | chr20 | 43380181 | rs736389 | A/G |
| chr20 | 43395411 | 4 | 10 | chr20 | 43395410 | rs6073714 | G/T |
| chr20 | 43508245 | 4 | 1 | chr20 | 43508244 | rs6104159 | A/G |
| chr20 | 44181354 | 3 | 3 | chr20 | 44181353 | rs4810485 | G/T |
| chr20 | 44782125 | 3 | 3 | chr20 | 44782123 | rs2143044 | C/T |
| chr20 | 44822959 | 3 | 1 | chr20 | 44822957 | rs4810553 | C/T |
| chr20 | 44828235 | 1 | 6 | chr20 | 44828233 | rs6018034 | C/T |

| | | | | | | | |
|---|---|---|---|---|---|---|---|
| chr20 | 45128958 | 2 | 6 | chr20 | 45128956 | rs1206811 | C/T |
| chr20 | 46538679 | 2 | 7 | chr20 | 46538677 | rs6012443 | C/T |
| chr20 | 46680136 | 4 | 1 | chr20 | 46680134 | rs6090884 | A/C |
| chr20 | 46852371 | 5 | 3 | chr20 | 46852369 | rs13039688 | C/G |
| chr20 | 47267044 | 5 | 3 | chr20 | 47267042 | rs4810935 | C/T |
| chr20 | 47461464 | 6 | 2 | chr20 | 47461462 | rs6125648 | C/T |
| chr20 | 47645713 | 5 | 5 | chr20 | 47645712 | rs566718 | C/T |
| chr20 | 47766391 | 1 | 3 | chr20 | 47766390 | rs6091031 | A/G |
| chr20 | 47840535 | 3 | 2 | chr20 | 47840533 | rs618019 | C/T |
| chr20 | 47866466 | 2 | 2 | chr20 | 47866465 | rs17785895 | A/G |
| chr20 | 48080187 | 1 | 3 | chr20 | 48080184 | rs6063463 | C/T |
| chr20 | 48226768 | 10 | 2 | chr20 | 48226768 | rs2869961 | A/G |
| chr20 | 48360379 | 2 | 2 | chr20 | 48360377 | rs13037502 | C/T |
| chr20 | 48506040 | 0 | 0 | chr20 | 48506038 | rs6095957 | C/T |
| chr20 | 48560145 | 4 | 1 | chr20 | 48560144 | rs6020570 | A/G |
| chr20 | 48560145 | 4 | 1 | chr20 | 48560144 | rs71214735 | AA/GG |
| chr20 | 48560145 | 4 | 1 | chr20 | 48560145 | rs4140567 | C/T |
| chr20 | 48647563 | 3 | 1 | chr20 | 48647562 | rs6020624 | A/G |
| chr20 | 48701335 | 4 | 2 | chr20 | 48701333 | rs6126061 | C/T |
| chr20 | 48883336 | 2 | 3 | chr20 | 48883335 | rs6020784 | A/G |
| chr20 | 49456828 | 6 | 4 | chr20 | 49456826 | rs12624853 | C/T |
| chr20 | 49477370 | 3 | 11 | chr20 | 49477369 | rs6126227 | A/G |
| chr20 | 49539334 | 12 | 4 | chr20 | 49539333 | rs6067797 | A/G |
| chr20 | 49721332 | 3 | 4 | chr20 | 49721331 | rs2255358 | C/T |
| chr20 | 50610775 | 2 | 2 | chr20 | 50610774 | rs2179885 | A/G |
| chr20 | 51088334 | 3 | 3 | chr20 | 51088332 | rs6022241 | C/T |
| chr20 | 51371914 | 3 | 1 | chr20 | 51371912 | rs200646 | A/G |
| chr20 | 51393395 | 2 | 2 | chr20 | 51393392 | rs200622 | C/T |
| chr20 | 51855424 | 1 | 5 | chr20 | 51855422 | rs6097587 | C/T |
| chr20 | 52037954 | 5 | 3 | chr20 | 52037953 | rs119069 | C/T |
| chr20 | 52234273 | 3 | 1 | chr20 | 52234272 | rs6023005 | A/G |
| chr20 | 52690285 | 2 | 4 | chr20 | 52690284 | rs1569982 | A/G |

| | | | | | | | |
|---|---|---|---|---|---|---|---|
| chr20 | 53117650 | 2 | 2 | chr20 | 53117649 | rs158321 | C/T |
| chr20 | 53211514 | 3 | 1 | chr20 | 53211512 | rs13039015 | C/T |
| chr20 | 53882028 | 1 | 7 | chr20 | 53882027 | rs2295460 | C/T |
| chr20 | 53923414 | 3 | 1 | chr20 | 53923412 | rs456138 | C/T |
| chr20 | 54283385 | 4 | 2 | chr20 | 54283383 | rs57771019 | C/T |
| chr20 | 54517639 | 3 | 6 | chr20 | 54517638 | rs6024919 | A/G |
| chr20 | 54636391 | 3 | 2 | chr20 | 54636390 | rs3827102 | G/T |
| chr20 | 54749545 | 3 | 1 | chr20 | 54749544 | rs6025118 | A/G |
| chr20 | 54952684 | 2 | 4 | chr20 | 54952682 | rs1296595 | C/T |
| chr20 | 54957040 | 1 | 3 | chr20 | 54957039 | rs1276412 | A/G |
| chr20 | 55091051 | 1 | 4 | chr20 | 55091050 | rs753341 | A/G |
| chr20 | 55114976 | 2 | 4 | chr20 | 55114974 | rs4811807 | C/T |
| chr20 | 55451430 | 1 | 3 | chr20 | 55451429 | rs58879921 | A/G |
| chr20 | 55552538 | 3 | 1 | chr20 | 55552537 | rs728064 | C/G |
| chr20 | 55622395 | 3 | 1 | chr20 | 55622394 | rs2865395 | A/G |
| chr20 | 55692981 | 2 | 3 | chr20 | 55692979 | rs157100 | C/T |
| chr20 | 56184045 | 1 | 5 | chr20 | 56184044 | rs6015217 | A/G |
| chr20 | 56226952 | 5 | 5 | chr20 | 56226951 | rs583987 | A/G |
| chr20 | 56661129 | 1 | 5 | chr20 | 56661128 | rs218471 | C/G |
| chr20 | 57759827 | 2 | 7 | chr20 | 57759826 | rs864184 | C/T |
| chr20 | 57783707 | 1 | 3 | chr20 | 57783706 | rs2153170 | A/G |
| chr20 | 58704953 | 7 | 1 | chr20 | 58704951 | rs1201902 | C/T |
| chr20 | 58876270 | 2 | 4 | chr20 | 58876269 | rs34513068 | A/G |
| chr20 | 58957902 | 3 | 3 | chr20 | 58957900 | rs237658 | C/T |
| chr20 | 58977940 | 1 | 4 | chr20 | 58977939 | rs6027938 | A/G |
| chr20 | 59027556 | 3 | 1 | chr20 | 59027555 | rs4277583 | C/G |
| chr20 | 59326236 | 3 | 9 | chr20 | 59326234 | rs2427076 | C/T |
| chr20 | 59590517 | 2 | 2 | chr20 | 59590515 | rs2249795 | C/T |
| chr20 | 59607193 | 1 | 11 | chr20 | 59607191 | rs6142662 | C/T |
| chr20 | 59607193 | 1 | 11 | chr20 | 59607192 | rs2253091 | A/G |
| chr20 | 59715639 | 4 | 1 | chr20 | 59715638 | rs944254 | C/T |
| chr20 | 59740788 | 1 | 3 | chr20 | 59740787 | rs2386945 | C/T |

| | | | | | | | |
|---|---|---|---|---|---|---|---|
| chr20 | 59886438 | 13 | 1 | chr20 | 59886437 | rs6061376 | A/G |
| chr20 | 59904618 | 1 | 3 | chr20 | 59904617 | rs1776248 | C/T |
| chr20 | 59915090 | 11 | 3 | chr20 | 59915089 | rs6061380 | A/G |
| chr20 | 60013570 | 3 | 3 | chr20 | 60013568 | rs4925331 | C/T |
| chr20 | 60024480 | 2 | 7 | chr20 | 60024478 | rs6089608 | C/T |
| chr20 | 60068634 | 4 | 1 | chr20 | 60068632 | rs731657 | C/T |
| chr20 | 60111086 | 3 | 4 | chr20 | 60111085 | rs4244604 | A/G |
| chr20 | 60125287 | 4 | 4 | chr20 | 60125286 | rs6061435 | A/G |
| chr20 | 60214390 | 3 | 1 | chr20 | 60214388 | rs7347529 | C/T |
| chr20 | 60316669 | 3 | 1 | chr20 | 60316667 | rs2236523 | G/T |
| chr20 | 60402853 | 1 | 3 | chr20 | 60402851 | rs2064830 | A/G |
| chr20 | 60402999 | 2 | 2 | chr20 | 60402997 | rs2064829 | A/G |
| chr20 | 60431477 | 4 | 1 | chr20 | 60431475 | rs4925233 | C/T |
| chr20 | 60591790 | 2 | 2 | chr20 | 60591788 | rs28513686 | C/T |
| chr20 | 60769181 | 1 | 3 | chr20 | 60769179 | rs2275326 | C/T |
| chr20 | 60903587 | 2 | 2 | chr20 | 60903585 | rs2294983 | C/T |
| chr20 | 60913992 | 4 | 5 | chr20 | 60913991 | rs2075776 | A/G |
| chr20 | 60918205 | 3 | 4 | chr20 | 60918203 | rs6122316 | C/T |
| chr20 | 60939017 | 3 | 3 | chr20 | 60939015 | rs2294995 | C/T |
| chr20 | 61047721 | 7 | 11 | chr20 | 61047719 | rs2427452 | C/T |
| chr20 | 61061345 | 6 | 2 | chr20 | 61061344 | rs2427461 | A/G |
| chr20 | 61083990 | 1 | 5 | chr20 | 61083988 | rs8184853 | C/G |
| chr20 | 61189377 | 5 | 8 | chr20 | 61189375 | rs6011605 | C/T |
| chr20 | 61191546 | 3 | 7 | chr20 | 61191544 | rs910930 | C/G |
| chr20 | 61202984 | 8 | 1 | chr20 | 61202982 | rs2427496 | C/T |
| chr20 | 61340910 | 3 | 7 | chr20 | 61340909 | rs1075557 | C/T |
| chr20 | 61343878 | 5 | 5 | chr20 | 61343876 | rs6122107 | C/T |
| chr20 | 61421935 | 7 | 1 | chr20 | 61421933 | rs6089881 | C/G |
| chr20 | 61449544 | 0 | 0 | chr20 | 61449543 | rs3787137 | A/G |
| chr20 | 61470114 | 1 | 5 | chr20 | 61470112 | rs4809295 | C/T |
| chr20 | 61520488 | 4 | 3 | chr20 | 61520487 | rs6011822 | A/G |
| chr20 | 61543499 | 0 | 0 | chr20 | 61543498 | rs35932225 | A/G |

| | | | | | | | |
|---|---|---|---|---|---|---|---|
| chr20 | 61716985 | 2 | 2 | chr20 | 61716984 | rs6011928 | A/G |
| chr20 | 61722073 | 3 | 4 | chr20 | 61722072 | rs914563 | C/T |
| chr20 | 61746290 | 7 | 2 | chr20 | 61746287 | rs6010986 | C/T |
| chr20 | 61746290 | 7 | 2 | chr20 | 61746288 | rs73135821 | C/T |
| chr20 | 61771656 | 0 | 0 | chr20 | 61771654 | rs13040839 | C/T |
| chr20 | 61797643 | 4 | 1 | chr20 | 61797642 | rs1056990 | A/G |
| chr20 | 61846672 | 5 | 2 | chr20 | 61846670 | rs2427537 | C/T |
| chr20 | 61983440 | 2 | 5 | chr20 | 61983439 | rs6122205 | A/G |
| chr20 | 62267777 | 2 | 2 | chr20 | 62267776 | rs436670 | A/G |
| chr21 | 10079622 | 4 | 1 | chr21 | 10079621 | rs2838185 | G/T |
| chr21 | 10120791 | 21 | 5 | chr21 | 10120790 | rs71240868 | C/T |
| chr21 | 10131837 | 3 | 4 | chr21 | 10131835 | rs2986802 | G/T |
| chr21 | 10131837 | 3 | 4 | chr21 | 10131835 | rs56077761 | A/C |
| chr21 | 10131837 | 3 | 4 | chr21 | 10131836 | rs2986801 | C/G |
| chr21 | 10151226 | 28 | 2 | chr21 | 10151224 | rs2341121 | C/T |
| chr21 | 10151226 | 28 | 2 | chr21 | 10151225 | rs600561 | A/G |
| chr21 | 10152556 | 3 | 5 | chr21 | 10152556 | rs62219826 | G/T |
| chr21 | 10154857 | 2 | 7 | chr21 | 10154855 | rs62219841 | C/T |
| chr21 | 10154857 | 2 | 7 | chr21 | 10154855 | rs73333380 | C/T |
| chr21 | 15363751 | 4 | 1 | chr21 | 15363749 | rs2823034 | A/C |
| chr21 | 15948563 | 2 | 2 | chr21 | 15948561 | rs9305681 | C/T |
| chr21 | 18889286 | 1 | 6 | chr21 | 18889285 | rs2824933 | A/G |
| chr21 | 19216240 | 4 | 6 | chr21 | 19216238 | rs408162 | A/C |
| chr21 | 19324133 | 1 | 4 | chr21 | 19324131 | rs2205527 | C/T |
| chr21 | 19549991 | 2 | 2 | chr21 | 19549989 | rs10222047 | A/C |
| chr21 | 19848208 | 2 | 3 | chr21 | 19848206 | rs2825637 | C/T |
| chr21 | 20035249 | 2 | 2 | chr21 | 20035247 | rs1811934 | A/C |
| chr21 | 21071264 | 1 | 16 | chr21 | 21071262 | rs12626347 | C/T |
| chr21 | 21071264 | 1 | 16 | chr21 | 21071263 | rs59172121 | C/G |
| chr21 | 21628523 | 2 | 3 | chr21 | 21628521 | rs2826803 | C/T |
| chr21 | 22051716 | 1 | 4 | chr21 | 22051714 | rs12627591 | C/T |
| chr21 | 23636313 | 1 | 4 | chr21 | 23636311 | rs2828079 | A/C |

| | | | | | | | |
|---|---|---|---|---|---|---|---|
| chr21 | 24321829 | 1 | 3 | chr21 | 24321829 | rs2828713 | A/G |
| chr21 | 25312547 | 6 | 2 | chr21 | 25312546 | rs2829515 | A/G |
| chr21 | 27127299 | 3 | 14 | chr21 | 27127299 | rs229038 | C/G |
| chr21 | 27546638 | 1 | 3 | chr21 | 27546636 | rs2078436 | A/G |
| chr21 | 29676340 | 2 | 3 | chr21 | 29676338 | rs2832306 | C/T |
| chr21 | 30728246 | 8 | 4 | chr21 | 30728246 | rs2226549 | A/G |
| chr21 | 31744281 | 4 | 1 | chr21 | 31744280 | rs9984485 | G/T |
| chr21 | 32170157 | 3 | 3 | chr21 | 32170156 | rs55889745 | A/G |
| chr21 | 32366683 | 1 | 3 | chr21 | 32366681 | rs915533 | A/G |
| chr21 | 32651401 | 4 | 1 | chr21 | 32651400 | rs2833786 | A/G |
| chr21 | 32777471 | 1 | 3 | chr21 | 32777471 | rs2833848 | A/G |
| chr21 | 33138383 | 9 | 3 | chr21 | 33138381 | rs9808696 | C/T |
| chr21 | 33533416 | 2 | 3 | chr21 | 33533414 | rs12482193 | C/T |
| chr21 | 34330377 | 3 | 2 | chr21 | 34330375 | rs4817613 | C/T |
| chr21 | 34638007 | 2 | 2 | chr21 | 34638005 | rs915564 | C/T |
| chr21 | 34863447 | 5 | 1 | chr21 | 34863446 | rs7278421 | A/G |
| chr21 | 36373660 | 2 | 4 | chr21 | 36373659 | rs73210610 | C/G |
| chr21 | 36530896 | 1 | 5 | chr21 | 36530894 | rs4817787 | C/T |
| chr21 | 36539500 | 6 | 2 | chr21 | 36539499 | rs4817788 | G/T |
| chr21 | 36746542 | 1 | 4 | chr21 | 36746540 | rs73204244 | A/C |
| chr21 | 36886996 | 7 | 4 | chr21 | 36886994 | rs9978422 | C/T |
| chr21 | 36932063 | 1 | 3 | chr21 | 36932061 | rs449414 | A/C |
| chr21 | 37078352 | 3 | 1 | chr21 | 37078352 | rs7280602 | C/G |
| chr21 | 37740237 | 5 | 7 | chr21 | 37740236 | rs2835749 | A/G |
| chr21 | 38008836 | 8 | 8 | chr21 | 38008834 | rs2070995 | A/G |
| chr21 | 38050587 | 3 | 3 | chr21 | 38050585 | rs857992 | C/T |
| chr21 | 38097338 | 3 | 1 | chr21 | 38097336 | rs2835970 | C/T |
| chr21 | 38154889 | 3 | 1 | chr21 | 38154887 | rs4817899 | C/T |
| chr21 | 38598360 | 4 | 4 | chr21 | 38598359 | rs6517461 | A/G |
| chr21 | 39081260 | 0 | 0 | chr21 | 39081259 | rs1309152 | A/G |
| chr21 | 39116929 | 2 | 3 | chr21 | 39116928 | rs711 | A/G |
| chr21 | 39608198 | 3 | 2 | chr21 | 39608196 | rs4624474 | C/T |

| | | | | | | | |
|---|---|---|---|---|---|---|---|
| chr21 | 40082112 | 4 | 3 | chr21 | 40082111 | rs456818 | A/G |
| chr21 | 40352258 | 1 | 6 | chr21 | 40352257 | rs9305690 | A/G |
| chr21 | 40521017 | 1 | 4 | chr21 | 40521015 | rs370432 | C/T |
| chr21 | 40683549 | 4 | 1 | chr21 | 40683547 | rs2837606 | C/T |
| chr21 | 41358203 | 5 | 6 | chr21 | 41358201 | rs9305718 | C/T |
| chr21 | 41360450 | 7 | 3 | chr21 | 41360449 | rs914171 | A/G |
| chr21 | 41385635 | 15 | 5 | chr21 | 41385633 | rs2837928 | C/T |
| chr21 | 41690841 | 3 | 2 | chr21 | 41690841 | rs395834 | A/C |
| chr21 | 41696411 | 3 | 1 | chr21 | 41696410 | rs466092 | A/G |
| chr21 | 41755098 | 2 | 2 | chr21 | 41755096 | rs460904 | C/T |
| chr21 | 41914218 | 1 | 4 | chr21 | 41914217 | rs9984044 | A/G |
| chr21 | 42062915 | 2 | 2 | chr21 | 42062914 | rs10887971 | A/G |
| chr21 | 42185821 | 3 | 4 | chr21 | 42185819 | rs8133175 | C/T |
| chr21 | 42226718 | 3 | 3 | chr21 | 42226716 | rs7281894 | C/T |
| chr21 | 42370018 | 5 | 1 | chr21 | 42370016 | rs428778 | A/G |
| chr21 | 42370018 | 5 | 1 | chr21 | 42370016 | rs70940578 | CA/TG |
| chr21 | 42370018 | 5 | 1 | chr21 | 42370017 | rs469618 | A/G |
| chr21 | 42403499 | 2 | 2 | chr21 | 42403498 | rs220123 | C/T |
| chr21 | 42578795 | 3 | 2 | chr21 | 42578793 | rs9653812 | A/C |
| chr21 | 42599687 | 4 | 3 | chr21 | 42599686 | rs225435 | A/G |
| chr21 | 42604068 | 6 | 4 | chr21 | 42604067 | rs225438 | A/G |
| chr21 | 42874135 | 1 | 3 | chr21 | 42874132 | rs454849 | C/T |
| chr21 | 43060406 | 3 | 2 | chr21 | 43060405 | rs9325640 | A/G |
| chr21 | 43064573 | 3 | 2 | chr21 | 43064570 | rs13048220 | A/C |
| chr21 | 43268800 | 8 | 9 | chr21 | 43268798 | rs1672115 | C/G |
| chr21 | 43349828 | 3 | 3 | chr21 | 43349827 | rs234701 | A/G |
| chr21 | 43365913 | 4 | 2 | chr21 | 43365911 | rs2605308 | A/C |
| chr21 | 43535510 | 2 | 4 | chr21 | 43535509 | rs9981412 | A/G |
| chr21 | 43582563 | 4 | 2 | chr21 | 43582561 | rs594170 | A/G |
| chr21 | 44387935 | 4 | 4 | chr21 | 44387933 | rs8126564 | C/T |
| chr21 | 44441101 | 4 | 1 | chr21 | 44441099 | rs2070551 | C/T |
| chr21 | 44462932 | 2 | 3 | chr21 | 44462931 | rs2838530 | A/G |

| | | | | | | | |
|---|---|---|---|---|---|---|---|
| chr21 | 44499973 | 1 | 4 | chr21 | 44499971 | rs968447 | C/T |
| chr21 | 44525272 | 1 | 9 | chr21 | 44525270 | rs60503656 | C/T |
| chr21 | 44525272 | 1 | 9 | chr21 | 44525271 | rs2776376 | A/G |
| chr21 | 44571342 | 1 | 3 | chr21 | 44571340 | rs2073436 | C/G |
| chr21 | 44594694 | 1 | 6 | chr21 | 44594691 | rs11910460 | C/T |
| chr21 | 44642049 | 2 | 4 | chr21 | 44642047 | rs1785436 | C/T |
| chr21 | 44944767 | 8 | 3 | chr21 | 44944765 | rs11088959 | C/G |
| chr21 | 45062578 | 1 | 5 | chr21 | 45062578 | rs663223 | G/T |
| chr21 | 45127763 | 3 | 1 | chr21 | 45127762 | rs7280520 | A/G |
| chr21 | 45240446 | 3 | 2 | chr21 | 45240444 | rs4819007 | C/T |
| chr21 | 45241250 | 6 | 1 | chr21 | 45241248 | rs4819009 | C/T |
| chr21 | 45318208 | 2 | 4 | chr21 | 45318207 | rs8132098 | A/G |
| chr21 | 45377427 | 3 | 1 | chr21 | 45377425 | rs372173 | C/T |
| chr21 | 45551838 | 2 | 24 | chr21 | 45551836 | rs8129440 | A/C |
| chr21 | 45588889 | 2 | 4 | chr21 | 45588887 | rs2838881 | C/T |
| chr21 | 45627309 | 7 | 1 | chr21 | 45627308 | rs4819082 | A/G |
| chr21 | 45652545 | 2 | 6 | chr21 | 45652544 | rs4819098 | A/G |
| chr21 | 45775984 | 3 | 3 | chr21 | 45775983 | rs12659 | C/T |
| chr21 | 45800665 | 3 | 1 | chr21 | 45800663 | rs1055343 | C/T |
| chr21 | 45806764 | 4 | 1 | chr21 | 45806762 | rs944422 | C/T |
| chr21 | 45856565 | 3 | 1 | chr21 | 45856563 | rs11701131 | C/T |
| chr21 | 45993203 | 2 | 12 | chr21 | 45993202 | rs8129304 | G/T |
| chr21 | 46156609 | 6 | 2 | chr21 | 46156608 | rs7410105 | A/G |
| chr21 | 46187761 | 5 | 5 | chr21 | 46187759 | rs408750 | C/T |
| chr21 | 46187761 | 5 | 5 | chr21 | 46187760 | rs35564189 | A/G |
| chr21 | 46227583 | 5 | 5 | chr21 | 46227582 | rs9974290 | A/G |
| chr21 | 46228825 | 3 | 4 | chr21 | 46228824 | rs3746993 | A/G |
| chr21 | 46269613 | 4 | 3 | chr21 | 46269611 | rs2839084 | C/T |
| chr21 | 46310024 | 3 | 3 | chr21 | 46310023 | rs56284934 | A/G |
| chr21 | 46363962 | 5 | 5 | chr21 | 46363960 | rs9980483 | C/T |
| chr21 | 46373939 | 2 | 3 | chr21 | 46373937 | rs7717 | C/G |
| chr21 | 46546167 | 5 | 7 | chr21 | 46546166 | rs3747007 | A/G |

| | | | | | | | | |
|---|---|---|---|---|---|---|---|---|
| chr21 | 46549131 | 5 | 1 | chr21 | 46549129 | rs4818835 | | C/T |
| chr21 | 46912273 | 3 | 5 | chr21 | 46912273 | rs8131044 | | G/T |
| chr22 | 15558213 | 3 | 3 | chr22 | 15558212 | rs2845371 | | A/G |
| chr22 | 15605320 | 2 | 4 | chr22 | 15605318 | rs987283 | | A/C |
| chr22 | 15917917 | 2 | 2 | chr22 | 15917916 | rs2845406 | | C/T |
| chr22 | 16636991 | 6 | 3 | chr22 | 16636990 | rs403934 | | G/T |
| chr22 | 16676485 | 1 | 3 | chr22 | 16676483 | rs1550664 | | C/T |
| chr22 | 16681694 | 4 | 1 | chr22 | 16681692 | rs8135914 | | C/T |
| chr22 | 16689412 | 11 | 8 | chr22 | 16689410 | rs5992864 | | C/T |
| chr22 | 16884528 | 1 | 3 | chr22 | 16884526 | rs397152 | | C/T |
| chr22 | 16886774 | 4 | 5 | chr22 | 16886772 | rs9604822 | | C/T |
| chr22 | 17158847 | 3 | 2 | chr22 | 17158846 | rs645912 | | G/T |
| chr22 | 17292678 | 3 | 2 | chr22 | 17292676 | rs11913840 | | C/T |
| chr22 | 17292678 | 3 | 2 | chr22 | 17292677 | rs4819756 | | A/G |
| chr22 | 17364848 | 5 | 5 | chr22 | 17364847 | rs1210641 | | A/G |
| chr22 | 17642853 | 3 | 1 | chr22 | 17642851 | rs362148 | | C/T |
| chr22 | 17670968 | 4 | 2 | chr22 | 17670968 | rs713885 | | A/C |
| chr22 | 18209803 | 2 | 3 | chr22 | 18209802 | rs5748453 | | A/G |
| chr22 | 18326898 | 2 | 4 | chr22 | 18326896 | rs56104268 | | A/C |
| chr22 | 18352825 | 1 | 3 | chr22 | 18352823 | rs917478 | | C/T |
| chr22 | 18391838 | 7 | 2 | chr22 | 18391836 | rs5746852 | | C/T |
| chr22 | 18532911 | 2 | 3 | chr22 | 18532910 | rs72617229 | | C/T |
| chr22 | 18551448 | 10 | 4 | chr22 | 18551448 | rs659918 | | C/G |
| chr22 | 18563346 | 1 | 3 | chr22 | 18563344 | rs637599 | | C/T |
| chr22 | 18743588 | 0 | 0 | chr22 | 18743586 | rs71232379 | | A/C |
| chr22 | 18855021 | 0 | 0 | chr22 | 18855020 | rs2542873 | | C/T |
| chr22 | 18855021 | 0 | 0 | chr22 | 18855020 | rs12484829 | | A/G |
| chr22 | 18855021 | 0 | 0 | chr22 | 18855020 | rs71317880 | | C/T |
| chr22 | 19440241 | 4 | 5 | chr22 | 19440239 | rs738060 | | C/T |
| chr22 | 19652585 | 3 | 1 | chr22 | 19652584 | rs2011016 | | A/G |
| chr22 | 20054424 | 0 | 0 | chr22 | 20054422 | rs2542873 | | C/T |
| chr22 | 20054424 | 0 | 0 | chr22 | 20054422 | rs71317880 | | C/T |

| | | | | | | | |
|---|---|---|---|---|---|---|---|
| chr22 | 20380769 | 2 | 5 | chr22 | 20380768 | rs710173 | A/G |
| chr22 | 20628274 | 5 | 15 | chr22 | 20628272 | rs2329885 | C/T |
| chr22 | 20724145 | 3 | 1 | chr22 | 20724143 | rs365783 | C/T |
| chr22 | 20880450 | 5 | 1 | chr22 | 20880449 | rs2073447 | C/G |
| chr22 | 20880760 | 6 | 6 | chr22 | 20880759 | rs5757323 | A/G |
| chr22 | 21378146 | 2 | 4 | chr22 | 21378144 | rs4820528 | C/T |
| chr22 | 21484887 | 1 | 3 | chr22 | 21484885 | rs7289658 | C/T |
| chr22 | 21554939 | 5 | 2 | chr22 | 21554938 | rs415919 | A/G |
| chr22 | 21841472 | 1 | 3 | chr22 | 21841471 | rs73152614 | A/G |
| chr22 | 22052181 | 3 | 2 | chr22 | 22052179 | rs1812290 | C/T |
| chr22 | 22052181 | 3 | 2 | chr22 | 22052179 | rs72617229 | C/T |
| chr22 | 22095954 | 3 | 2 | chr22 | 22095952 | rs6003705 | A/C |
| chr22 | 22125191 | 2 | 4 | chr22 | 22125189 | rs3827374 | C/T |
| chr22 | 22438288 | 3 | 1 | chr22 | 22438287 | rs140182 | C/G |
| chr22 | 22494509 | 4 | 1 | chr22 | 22494508 | rs2267037 | A/G |
| chr22 | 22522081 | 1 | 5 | chr22 | 22522080 | rs738802 | A/G |
| chr22 | 22570018 | 2 | 2 | chr22 | 22570016 | rs5760091 | C/T |
| chr22 | 23146354 | 2 | 2 | chr22 | 23146352 | rs738815 | C/T |
| chr22 | 24084600 | 4 | 4 | chr22 | 24084599 | rs133195 | A/G |
| chr22 | 24100474 | 1 | 4 | chr22 | 24100472 | rs133215 | C/T |
| chr22 | 24503662 | 4 | 6 | chr22 | 24503660 | rs5761170 | C/T |
| chr22 | 24511768 | 1 | 4 | chr22 | 24511766 | rs3859870 | C/T |
| chr22 | 25080231 | 5 | 8 | chr22 | 25080229 | rs655581 | C/T |
| chr22 | 25122113 | 4 | 4 | chr22 | 25122112 | rs5752318 | A/G |
| chr22 | 25423508 | 3 | 1 | chr22 | 25423507 | rs971280 | A/G |
| chr22 | 25534755 | 2 | 5 | chr22 | 25534753 | rs4822803 | C/T |
| chr22 | 25751632 | 4 | 3 | chr22 | 25751631 | rs4418 | A/G |
| chr22 | 25847802 | 1 | 4 | chr22 | 25847799 | rs134940 | C/T |
| chr22 | 25992176 | 0 | 0 | chr22 | 25992175 | rs525783 | C/T |
| chr22 | 26547320 | 2 | 3 | chr22 | 26547318 | rs16985575 | C/T |
| chr22 | 27796827 | 1 | 6 | chr22 | 27796824 | rs134590 | C/T |
| chr22 | 27946265 | 3 | 4 | chr22 | 27946264 | rs132384 | A/G |

| | | | | | | | |
|---|---|---|---|---|---|---|---|
| chr22 | 28064695 | 1 | 5 | chr22 | 28064695 | rs174766 | G/T |
| chr22 | 28418443 | 3 | 1 | chr22 | 28418441 | rs1009148 | C/T |
| chr22 | 28997278 | 1 | 5 | chr22 | 28997276 | rs9608859 | C/T |
| chr22 | 29130368 | 10 | 4 | chr22 | 29130367 | rs4820846 | A/G |
| chr22 | 29946301 | 1 | 3 | chr22 | 29946300 | rs1034588 | C/T |
| chr22 | 31434348 | 1 | 9 | chr22 | 31434347 | rs5754226 | A/G |
| chr22 | 31499117 | 4 | 2 | chr22 | 31499114 | rs5998634 | C/T |
| chr22 | 32345991 | 3 | 3 | chr22 | 32345990 | rs1029297 | A/G |
| chr22 | 33477445 | 2 | 6 | chr22 | 33477444 | rs5755269 | A/G |
| chr22 | 33867472 | 1 | 4 | chr22 | 33867471 | rs134230 | A/G |
| chr22 | 34223482 | 3 | 1 | chr22 | 34223481 | rs16995748 | A/G |
| chr22 | 34281220 | 1 | 3 | chr22 | 34281218 | rs5999865 | C/T |
| chr22 | 34458396 | 3 | 2 | chr22 | 34458395 | rs5995166 | A/G |
| chr22 | 34904713 | 1 | 3 | chr22 | 34904712 | rs132681 | A/G |
| chr22 | 34915473 | 0 | 0 | chr22 | 34915471 | rs1053983 | A/G |
| chr22 | 35043700 | 2 | 2 | chr22 | 35043699 | rs6000238 | A/G |
| chr22 | 35663740 | 1 | 5 | chr22 | 35663739 | rs131840 | C/T |
| chr22 | 35881553 | 3 | 1 | chr22 | 35881552 | rs743777 | A/G |
| chr22 | 36146883 | 3 | 2 | chr22 | 36146882 | rs133731 | C/T |
| chr22 | 36286788 | 1 | 3 | chr22 | 36286785 | rs8140986 | A/C |
| chr22 | 36482876 | 5 | 1 | chr22 | 36482874 | rs4821705 | C/T |
| chr22 | 36511325 | 2 | 4 | chr22 | 36511324 | rs2413484 | A/G |
| chr22 | 36888405 | 6 | 2 | chr22 | 36888404 | rs6001034 | A/G |
| chr22 | 36968544 | 3 | 4 | chr22 | 36968542 | rs2413510 | C/T |
| chr22 | 37558441 | 5 | 1 | chr22 | 37558439 | rs5757302 | C/T |
| chr22 | 37561485 | 2 | 4 | chr22 | 37561483 | rs11703122 | C/T |
| chr22 | 37815071 | 3 | 6 | chr22 | 37815071 | rs5757473 | A/G |
| chr22 | 39411508 | 1 | 3 | chr22 | 39411506 | rs133076 | C/T |
| chr22 | 39493055 | 3 | 6 | chr22 | 39493053 | rs5758069 | A/C |
| chr22 | 39845690 | 11 | 6 | chr22 | 39845689 | rs5758237 | G/T |
| chr22 | 40338014 | 3 | 2 | chr22 | 40338013 | rs132765 | A/G |
| chr22 | 40526413 | 10 | 1 | chr22 | 40526412 | rs738248 | A/G |

| | | | | | | | |
|---|---|---|---|---|---|---|---|
| chr22 | 40742879 | 1 | 4 | chr22 | 40742878 | rs5751202 | A/G |
| chr22 | 41012974 | 5 | 3 | chr22 | 41012973 | rs134899 | G/T |
| chr22 | 41419000 | 7 | 3 | chr22 | 41418998 | rs6002904 | C/G |
| chr22 | 41495811 | 0 | 0 | chr22 | 41495809 | rs6002950 | C/T |
| chr22 | 41953339 | 3 | 3 | chr22 | 41953338 | rs129415 | C/G |
| chr22 | 41972853 | 1 | 3 | chr22 | 41972852 | rs4822267 | G/T |
| chr22 | 41983569 | 8 | 2 | chr22 | 41983568 | rs139030 | A/G |
| chr22 | 42000460 | 2 | 2 | chr22 | 42000459 | rs139044 | A/G |
| chr22 | 42085089 | 3 | 5 | chr22 | 42085087 | rs3091398 | C/T |
| chr22 | 42091812 | 4 | 2 | chr22 | 42091811 | rs761743 | A/G |
| chr22 | 42499314 | 1 | 8 | chr22 | 42499313 | rs739234 | A/G |
| chr22 | 42607439 | 4 | 1 | chr22 | 42607437 | rs138129 | C/T |
| chr22 | 42613609 | 4 | 3 | chr22 | 42613608 | rs739231 | A/G |
| chr22 | 42925468 | 3 | 2 | chr22 | 42925467 | rs139149 | A/G |
| chr22 | 42927419 | 3 | 5 | chr22 | 42927417 | rs139156 | A/C |
| chr22 | 43038359 | 2 | 3 | chr22 | 43038358 | rs7510759 | A/G |
| chr22 | 43186249 | 2 | 11 | chr22 | 43186248 | rs129543 | A/G |
| chr22 | 43352002 | 12 | 3 | chr22 | 43352000 | rs2349631 | A/C |
| chr22 | 43352403 | 1 | 10 | chr22 | 43352402 | rs2349634 | A/G |
| chr22 | 43555667 | 1 | 6 | chr22 | 43555666 | rs1557557 | A/G |
| chr22 | 43870294 | 2 | 5 | chr22 | 43870293 | rs5766414 | A/G |
| chr22 | 44643909 | 0 | 0 | chr22 | 44643907 | rs12167093 | C/T |
| chr22 | 44643909 | 0 | 0 | chr22 | 44643908 | rs136049 | A/G |
| chr22 | 45152256 | 1 | 3 | chr22 | 45152255 | rs9615969 | C/G |
| chr22 | 45300436 | 8 | 8 | chr22 | 45300435 | rs4823844 | A/G |
| chr22 | 45307853 | 11 | 3 | chr22 | 45307852 | rs5767247 | G/T |
| chr22 | 45349224 | 4 | 3 | chr22 | 45349223 | rs2142663 | C/T |
| chr22 | 45381295 | 6 | 10 | chr22 | 45381293 | rs73888577 | C/T |
| chr22 | 45381295 | 6 | 10 | chr22 | 45381294 | rs909557 | C/T |
| chr22 | 45732285 | 3 | 2 | chr22 | 45732284 | rs136086 | A/G |
| chr22 | 45895772 | 2 | 3 | chr22 | 45895772 | rs5766681 | C/G |
| chr22 | 45941588 | 6 | 1 | chr22 | 45941587 | rs4823607 | A/G |

| | | | | | | | |
|---|---|---|---|---|---|---|---|
| chr22 | 45945346 | 3 | 4 | chr22 | 45945345 | rs7291893 | A/G |
| chr22 | 46119432 | 12 | 1 | chr22 | 46119430 | rs3890164 | A/G |
| chr22 | 46636894 | 1 | 6 | chr22 | 46636893 | rs761916 | A/G |
| chr22 | 46685900 | 3 | 1 | chr22 | 46685900 | rs5768145 | C/G |
| chr22 | 46954641 | 1 | 3 | chr22 | 46954640 | rs8135441 | A/G |
| chr22 | 47091140 | 3 | 3 | chr22 | 47091138 | rs2235166 | C/G/T |
| chr22 | 47146311 | 4 | 5 | chr22 | 47146309 | rs133686 | C/T |
| chr22 | 47200518 | 5 | 2 | chr22 | 47200517 | rs5767143 | A/G |
| chr22 | 47263261 | 4 | 5 | chr22 | 47263260 | rs6008730 | A/G |
| chr22 | 47305800 | 3 | 2 | chr22 | 47305798 | rs133476 | A/G |
| chr22 | 47376414 | 5 | 5 | chr22 | 47376413 | rs28385606 | A/G |
| chr22 | 47411349 | 3 | 1 | chr22 | 47411348 | rs910579 | A/G |
| chr22 | 47442475 | 1 | 3 | chr22 | 47442473 | rs5771693 | C/T |
| chr22 | 47453489 | 4 | 6 | chr22 | 47453488 | rs2057204 | A/G |
| chr22 | 47458167 | 3 | 2 | chr22 | 47458166 | rs132216 | A/G |
| chr22 | 47492790 | 3 | 5 | chr22 | 47492789 | rs132243 | A/G |
| chr22 | 47517036 | 1 | 3 | chr22 | 47517034 | rs1015512 | C/T |
| chr22 | 47651226 | 8 | 4 | chr22 | 47651223 | rs28703021 | A/C |
| chr22 | 47668787 | 5 | 1 | chr22 | 47668786 | rs28744458 | G/T |
| chr22 | 47734131 | 1 | 3 | chr22 | 47734130 | rs6009261 | A/G |
| chr22 | 47933968 | 1 | 4 | chr22 | 47933967 | rs12329931 | C/G |
| chr22 | 47934317 | 3 | 5 | chr22 | 47934316 | rs13057532 | A/G |
| chr22 | 47998598 | 2 | 3 | chr22 | 47998596 | rs2688168 | C/G |
| chr22 | 48058916 | 5 | 2 | chr22 | 48058915 | rs2858520 | A/G |
| chr22 | 48553657 | 3 | 2 | chr22 | 48553655 | rs4468 | C/T |
| chr22 | 48668607 | 12 | 8 | chr22 | 48668606 | rs738711 | A/G |
| chr22 | 48782009 | 9 | 1 | chr22 | 48782007 | rs137847 | C/T |
| chr22 | 48816089 | 0 | 0 | chr22 | 48816087 | rs5771313 | C/T |
| chr22 | 48979320 | 6 | 1 | chr22 | 48979319 | rs3747941 | A/G |
| chr22 | 48997225 | 3 | 4 | chr22 | 48997224 | rs2272851 | A/G |
| chr22 | 49223102 | 6 | 6 | chr22 | 49223100 | rs5770779 | C/T |
| chr22 | 49226517 | 2 | 4 | chr22 | 49226516 | rs4824133 | A/G |

| | | | | | | | | |
|---|---|---|---|---|---|---|---|---|
| chr22 | 49497034 | 3 | 3 | chr22 | 49497032 | rs5770819 | | C/T |
| chrX | 127340 | 0 | 0 | chrX | 127341 | rs28380056 | | A/G |
| chrX | 316437 | 2 | 6 | chrX | 316434 | rs5950841 | | A/C |
| chrX | 316437 | 2 | 6 | chrX | 316434 | rs73617760 | | A/C |
| chrX | 916143 | 0 | 0 | chrX | 916141 | rs34394253 | | -/A/G |
| chrX | 916143 | 0 | 0 | chrX | 916141 | rs62604681 | | C/T |
| chrX | 916143 | 0 | 0 | chrX | 916141 | rs67222979 | | C/T |
| chrX | 1160654 | 2 | 3 | chrX | 1160653 | rs67070289 | | C/G |
| chrX | 2625523 | 2 | 2 | chrX | 2625522 | rs28734886 | | A/G |
| chrX | 2625523 | 2 | 2 | chrX | 2625522 | rs73188834 | | A/G |
| chrX | 9702043 | 7 | 1 | chrX | 9702042 | rs2521655 | | A/G |
| chrX | 16729886 | 1 | 3 | chrX | 16729885 | rs6527728 | | A/G |
| chrX | 17729300 | 4 | 1 | chrX | 17729297 | rs6527818 | | C/T |
| chrX | 18734194 | 3 | 3 | chrX | 18734194 | rs2239439 | | C/T |
| chrX | 18992478 | 3 | 1 | chrX | 18992476 | rs11094761 | | C/T |
| chrX | 23628018 | 6 | 2 | chrX | 23628018 | rs11797996 | | A/G |
| chrX | 24610962 | 5 | 3 | chrX | 24610961 | rs1209395 | | A/G |
| chrX | 32852195 | 6 | 1 | chrX | 32852192 | rs1590199 | | A/C |
| chrX | 33820996 | 1 | 7 | chrX | 33820995 | rs4829306 | | A/G |
| chrX | 39078995 | 6 | 1 | chrX | 39078994 | rs3843786 | | C/T |
| chrX | 40058538 | 5 | 1 | chrX | 40058537 | rs3008970 | | C/T |
| chrX | 45532885 | 4 | 1 | chrX | 45532885 | rs7050010 | | A/G |
| chrX | 45722611 | 7 | 4 | chrX | 45722609 | rs1008309 | | A/G |
| chrX | 50364288 | 4 | 3 | chrX | 50364286 | rs4826641 | | C/T |
| chrX | 50839016 | 3 | 1 | chrX | 50839016 | rs2382923 | | A/G |
| chrX | 56804416 | 1 | 5 | chrX | 56804414 | rs5914785 | | C/T |
| chrX | 57636373 | 0 | 0 | chrX | 57636372 | rs1057328 | | C/G/T |
| chrX | 69282122 | 1 | 5 | chrX | 69282121 | rs5936542 | | A/G |
| chrX | 69306866 | 2 | 2 | chrX | 69306864 | rs5936545 | | C/T |
| chrX | 70283771 | 3 | 2 | chrX | 70283770 | rs7051629 | | A/G |
| chrX | 70805209 | 0 | 0 | chrX | 70805208 | rs6418435 | | A/G |
| chrX | 78594547 | 1 | 4 | chrX | 78594546 | rs62606304 | | A/G |

| | | | | | | | |
|---|---|---|---|---|---|---|---|
| chrX | 79126218 | 2 | 6 | chrX | 79126217 | rs6616375 | A/G |
| chrX | 85639178 | 4 | 1 | chrX | 85639176 | rs5923540 | C/T |
| chrX | 86603291 | 0 | 0 | chrX | 86603289 | rs4828461 | C/T |
| chrX | 92598971 | 3 | 2 | chrX | 92598970 | rs5940216 | A/G |
| chrX | 106820292 | 6 | 3 | chrX | 106820290 | rs5916788 | C/T |
| chrX | 107125564 | 7 | 4 | chrX | 107125562 | rs73251761 | A/C |
| chrX | 108184358 | 8 | 22 | chrX | 108184357 | rs10871901 | A/G |
| chrX | 109184548 | 5 | 2 | chrX | 109184547 | rs5985440 | A/G |
| chrX | 124797732 | 4 | 2 | chrX | 124797730 | rs5932802 | C/T |
| chrX | 128661669 | 1 | 3 | chrX | 128661667 | rs5932660 | C/T |
| chrX | 129097460 | 0 | 0 | chrX | 129097460 | rs209997 | C/G |
| chrX | 139417031 | 2 | 2 | chrX | 139417029 | rs911093 | C/T |
| chrX | 141999744 | 2 | 4 | chrX | 141999743 | rs6636902 | A/G |
| chrX | 142234990 | 3 | 5 | chrX | 142234989 | rs73231753 | A/G |
| chrX | 148774364 | 0 | 0 | chrX | 148774363 | rs45518135 | G/T |
| chrX | 151012952 | 2 | 2 | chrX | 151012950 | rs1844893 | A/G |
| chrX | 151923695 | 6 | 2 | chrX | 151923693 | rs5924791 | C/T |
| chrX | 152461314 | 0 | 0 | chrX | 152461313 | rs3020947 | A/G |
| chrX | 152525255 | 0 | 0 | chrX | 152525254 | rs35504651 | A/G |
| chrX | 152724546 | 3 | 3 | chrX | 152724544 | rs3747310 | A/G |
| chrX | 152739567 | 1 | 3 | chrX | 152739565 | rs6643647 | A/C |
| chrY | 127340 | 0 | 0 | chrY | 127341 | rs28380056 | A/G |
| chrY | 316437 | 2 | 6 | chrY | 316434 | rs5950841 | A/C |
| chrY | 316437 | 2 | 6 | chrY | 316434 | rs73617760 | A/C |
| chrY | 916143 | 0 | 0 | chrY | 916141 | rs34394253 | -/A/G |
| chrY | 916143 | 0 | 0 | chrY | 916141 | rs62604681 | C/T |
| chrY | 916143 | 0 | 0 | chrY | 916141 | rs67222979 | C/T |
| chrY | 1160654 | 2 | 3 | chrY | 1160653 | rs67070289 | C/G |
| chrY | 2625523 | 2 | 2 | chrY | 2625522 | rs28734886 | A/G |
| chrY | 2625523 | 2 | 2 | chrY | 2625522 | rs73188834 | A/G |
| chrY | 10622169 | 28 | 2 | chrY | 10622168 | rs406205 | C/T |
| chrY | 10626629 | 3 | 2 | chrY | 10626628 | rs4034401 | G/T |

| | | | | | | | |
|---|---|---|---|---|---|---|---|
| chrY | 11670265 | 3 | 1 | chrY | 11670263 | rs73618415 | C/T |
| chrY | 11921836 | 4 | 157 | chrY | 11921835 | rs71288150 | -/G |
| chrY | 11928894 | 5 | 9 | chrY | 11928892 | rs36008153 | A/G |
| chrY | 11932536 | 12 | 2 | chrY | 11932534 | rs7089199 | C/T |
| chrY | 11939116 | 73 | 36 | chrY | 11939114 | rs71181483 | C/T |
| chrY | 11948654 | 13 | 1 | chrY | 11948654 | rs71245077 | C/T |
| chrY | 11948654 | 13 | 1 | chrY | 11948654 | rs36153424 | -/G |
| chrY | 11948871 | 8 | 2 | chrY | 11948870 | rs36153345 | C/T |
| chrY | 11948880 | 6 | 6 | chrY | 11948877 | rs36177540 | A/G |
| chrY | 11949731 | 80 | 1 | chrY | 11949728 | rs71250273 | CGG/TGA |
| chrY | 17070436 | 9 | 1 | chrY | 17070435 | rs9786479 | G/T |
| chrY | 27103875 | 3 | 2 | chrY | 27103873 | rs72621787 | A/G |
| chrY | 57394705 | 10 | 3 | chrY | 57394704 | rs36162130 | C/G |
| chrY | 57406250 | 4 | 3 | chrY | 57406248 | rs28873754 | C/T |
| chrY | 57406250 | 4 | 3 | chrY | 57406250 | rs2527439 | C/T |
| chrY | 57406250 | 4 | 3 | chrY | 57406250 | rs56139041 | A/G |
| chrY | 57426594 | 24 | 25 | chrY | 57426592 | rs2641135 | A/G |
| chrY | 57426594 | 24 | 25 | chrY | 57426593 | rs2527411 | C/T |
| chrY | 57432096 | 10 | 15 | chrY | 57432093 | rs3914987 | C/G |
| chrY | 57432096 | 10 | 15 | chrY | 57432095 | rs4104965 | C/T |
| chrY | 57441846 | 16 | 4 | chrY | 57441844 | rs2334082 | A/G |

*Weighted count: unique reads plus proportional distribution of <10 multiple alignments

¶ number was normalised to total read number per lane

**TableS 9. HELP-tagging adapter/primer sequences**

| Adapter/primer | Oligo Name | Sequence |
|---|---|---|
| Adapter AS | AS_1 | 5′-ACACTCTTTCCCTACACGACGCTCTTCCGATC-x-T |
| | AS_2 | 5′-p-GATCGGAAGAGCGTCGTGTAGGGAAAGAGTGT |
| Adapter AE | AE_1 | 5′-AcagtaatacgactcactatagggagaaggctCAAGCAGAAGACGGCATACGA**CAGCAG** |
| | AE_2 | 5′-p-CG**CTGCTG**TCGTATGCCGTCTTCTGCTTGagccttctccctatagtgagtcgtattactg |
| PCR primer | PS | 5′-AATGATACGGCGACCACCGAGATCTACACTCTTTCCCTACACGACGCTCTTCCGATCT |
| | PE | 5′-CAAGCAGAAGACGGCATACGACAGCAG |
| Sequencing primer | PSeq | 5′-ACACTCTTTCCCTACACGACGCTCTTCCGATCT |

x= phosphorothioate bond
p=phosphoryation
Lower case=T7 promoter sequence
**Bold**=EcoP15I site
